# Cryo-EM reveals multiple mechanisms of ribosome inhibition by doxycycline

**DOI:** 10.1038/s41467-026-73421-5

**Published:** 2026-06-01

**Authors:** William S. Stuart, Michail N. Isupov, Mathew McLaren, Christopher H. Jenkins, Adam Monier, Bertram Daum, Isobel H. Norville, Vicki A. M. Gold, Nicholas J. Harmer

**Affiliations:** 1 Living Systems Institute, University of Exeter, Stocker Road, Exeter, UK; 2 Department of Biosciences, University of Exeter, Stocker Road, Exeter, UK; 3 Dstl Porton Down, Salisbury, Wiltshire UK

**Keywords:** Cryoelectron microscopy, Antibiotics, Bacterial structural biology, Ribosome

## Abstract

Antimicrobial resistance is driving the search for new antibiotics and a greater understanding of their mechanism of action. Doxycycline is amongst the most-prescribed antimicrobials. It demonstrates a particularly low minimum inhibitory concentration against the zoonotic pathogen *Coxiella burnetii*. Doxycycline canonically targets the bacterial ribosome by blocking tRNA binding at the decoding centre (A site) of the small subunit. Using cryo-electron microscopy, we analysed doxycycline binding to *C. burnetii* and *Escherichia coli* ribosomes. Both structures reveal doxycycline binding at the exit tunnel in the large subunit. In *C. burnetii* three doxycycline molecules stack to block the tunnel. In *E. coli* one doxycycline molecule triggers a major change in the conformation of the ribosome. This rearrangement of the peptidyl transferase centre blocks tRNA binding and nascent chain accommodation, abolishing interactions that are fundamental to ribosome function. We identify a distinct ribosomal protein in the *C. burnetii* large subunit and characterise an additional member of the prokaryotic ribosome hibernation-promoting factor family. These insights into ribosome function and antibiotic action may aid the development of new ribosome inhibitor antibiotics.

## Abstract (web summary)

The common antibiotic doxycycline inhibits the ribosome. Here, using cryo-EM, the authors identify additional binding sites for doxycycline in the *Coxiella burnetii* and *E. coli* ribosomes.

## Introduction

The obligate pathogen *Coxiella burnetii* infects a wide range of mammals and other species^1,2^. With an infectious dose of approximately one bacterium, *C. burnetii* is highly contagious, putting veterinary and farm workers at risk^3–5^. *C. burnetii* has a biphasic lifecycle with large and small cell variants (LCV and SCV, respectively)^6,7^. The LCV is metabolically active, whereas the quiescent SCV has a spore-like morphology, enabling airborne spread^8^. Over 4,000 human cases were identified during a three-year outbreak in the Netherlands (2007 to 2010)^9^, with associated costs estimated at up to 600 million Euros^10^. Q-fever, the symptomatic *C. burnetii* infection in humans, initially presents with fever-like symptoms, with up to 5% of symptomatic infections developing into long-term chronic illness, although it can be many years before chronic infections are identified^11,12^. *C. burnetii* has been named as one of the 24 pathogens posing the greatest risk to public health^13^.

**Fig. 1:**
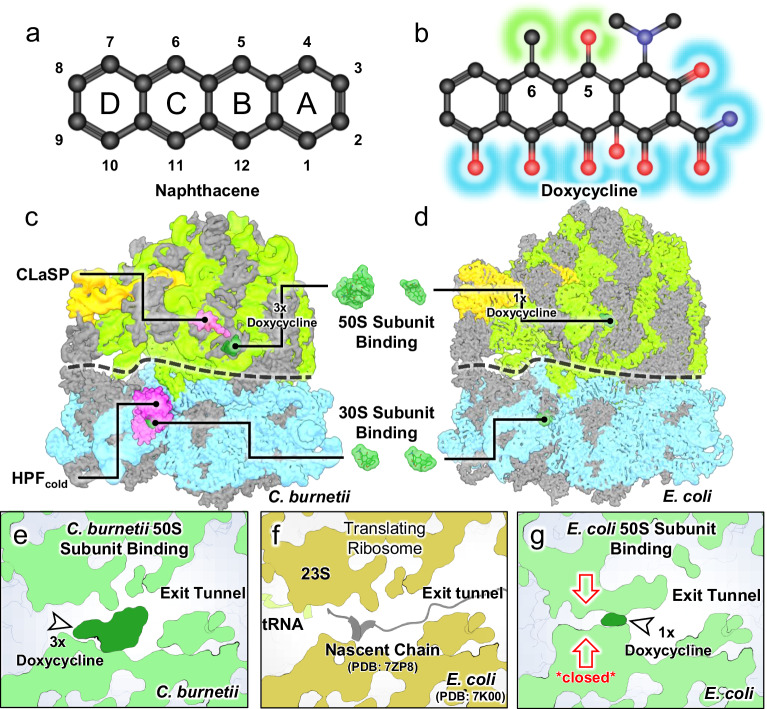
Cryo-EM reveals multiple doxycycline binding sites on the prokaryotic ribosome, a ribosome hibernation factor and a *Coxiellaceae* specific ribosomal protein. **a** Tetracyclines share a naphthacene core consisting of four fused carbon rings. **b** This is decorated by common modifications on carbons 1-3 and 10-12 (highlighted blue). Unique modifications at other positions (typically, carbons 4-8), such as a C5 hydroxyl group and a C6 methyl group for doxycycline (highlighted green), define the different family members. Later generation synthetic tetracyclines focused on additions around the D-ring (C7-9), expanding the physical footprint of the drugs to overcome tetracycline resistance mechanisms^91^. Overview of the structures of the *Coxiella burnetii* (**c**) and *Escherichia coli* (**d**) ribosomes in complex with 50 µM doxycycline. Proteins are coloured grey, 16S and 23S rRNA are coloured blue and green respectively and 5S is coloured pale orange. Bound doxycycline is indicated and coloured dark green; bound HPF_cold_ is shown in dark blue (lower left); CLaSP (*Coxiellaceae* Large Subunit Peptide) is shown in magenta. Doxycycline blocks the translating ribosome, each shows a cross section through the nascent chain exit tunnel region. The *C. burnetii* ribosome (**e**) is blocked by three molecules of doxycycline that block the tunnel that the nascent chain exits through. For comparison, an active ribosome (**f**), showing the canonical structure (dark yellow, PDB: 7k00^75^,) and nascent chain (grey cartoon, PDB: 7zp8^92^,) in an unimpeded exit tunnel. Doxycycline remodels the 23S rRNA chain at the peptidyl transfer centre (**g**), constricting this location and the exit tunnel.

Clearance of *C. burnetii* is highly challenging due to its slow growth rate and unique intracellular location within the *Coxiella* containing vacuole (CCV)^14^. Doxycycline (Fig. 1) and hydroxychloroquine are given in a regimen lasting for at least 18 months, consisting of two doses of doxycycline and three doses of hydroxychloroquine (respectively 200 and 300 mg daily)^15,16^. Hydroxychloroquine likely alkalises the acidic CCV, improving doxycycline performance^17^. *C. burnetii* is exquisitely sensitive to doxycycline (minimum inhibitory concentration (MIC) of between 0.01–0.04 µg/ml and a bactericidal concentration of 8 µg/ml)^18^. In comparison, the MIC of doxycycline against *E. coli* is around 1.5 µg/ml^19^.

Here, we present the structure of the *C. burnetii* ribosome and compare the doxycycline-bound forms of the *C. burnetii* and *E. coli* ribosomes to identify conformational differences associated with antibiotic binding. Ribosomes have three binding sites for tRNA molecules: the A, P and E sites. In prokaryotes, the translation initiation site is determined by the mRNA Shine-Dalgarno sequence pairing with the 16S rRNA anti-Shine-Dalgarno sequence in the 30S subunit. After formation of the translationally competent 70S ribosome, at the A-site decoding centre, aminoacylated tRNA is decoded by codon-anticodon base pairing with the mRNA. Peptide bond formation is catalysed by the peptidyl transferase centre (50S subunit), where the growing nascent polypeptide chain is transferred from the P-site tRNA to the amino group of the aminoacylated A-site tRNA. The extending polypeptide proceeds through the nascent chain exit tunnel in the large subunit. Translocation occurs, moving the mRNA and tRNAs to the next site (from A-site to P-site, or P-site to E-site).

The canonical tetracycline binding site overlaps the A-site tRNA, and we observe doxycycline bound here in each ribosome. However, in both organisms, additional doxycycline binding sites were identified around the nascent chain exit tunnel. Exit tunnel binding differs between both species and suggests an explanation for *C. burnetii’s* sensitivity to doxycycline. In the non-doxycycline-inhibited forms, paused, hibernating ribosomes predominated, induced by bound hibernation factor proteins. Hibernation factors, found in all forms of life, provide a way of pausing and protecting ribosomes when not engaged in protein synthesis^20,21^. Since cells eventually transition back to the SCV state within the infected host, where hibernating ribosomes likely predominate, this form is of clinical interest^22,23^. We observe a distinct *C. burnetii* large subunit peptide and identify a subpopulation of *C. burnetii* ribosomes complexed with a protein containing a cold shock domain, which we propose as an additional member of the hibernation-promoting factor (HPF) family, named HPF_cold_.

## Results

### Doxycycline binds to both large and small ribosomal subunits

To characterise species-specific features and contrast antibiotic behaviour, we determined the doxycycline (Fig. 1a, b) bound cryo-EM structures of the *C. burnetii* and *E. coli* ribosomes at 50 µM. Both datasets showed doxycycline bound in the expected 30S subunit location common to other tetracyclines^24–26^ (Fig. 1c, d, Supplementary Fig. 1). Focused classification was used to further interrogate doxycycline binding behaviour beyond the canonical site. Surprisingly, both populations also showed doxycycline bound to the 50S subunit, with distinct modes of binding for the two Gram-negative bacteria (Fig. 1c–g). The *C. burnetii* specific binding is similar to doxycycline binding to the *E. coli* ribosome at higher concentrations, recently observed by Devarkar et al.^27^.

### The Coxiella burnetii hibernating ribosome

The overall structure of the *C. burnetii* ribosome is similar to other prokaryotic ribosomes (Fig. 1c, Supplementary Movie 1). Forty-eight proteins identified in the 70S ribosome are listed in Supplementary Table 1 (28 in the large subunit, 20 in the small subunit, including a distinct large subunit peptide and a small subunit hibernation factor, both discussed below). Any remaining elements of the *C. burnetii* intervening sequence (IVS), found within the 23S ribosomal RNA (rRNA) were not present in the ribosome structure, nor the hypothetical S23 protein (encoded within the precursor 23S rRNA)^28^, correlating with previous studies^29^. There are notably fewer rRNA post-transcriptional modifications (PTMs) identified for the *C. burnetii* ribosome compared to *E. coli*, with those that remain being mostly in the small subunit (Supplementary Fig. 2).

### A prokaryotic hibernation factor protects the monomeric ribosome

**Fig. 2:**
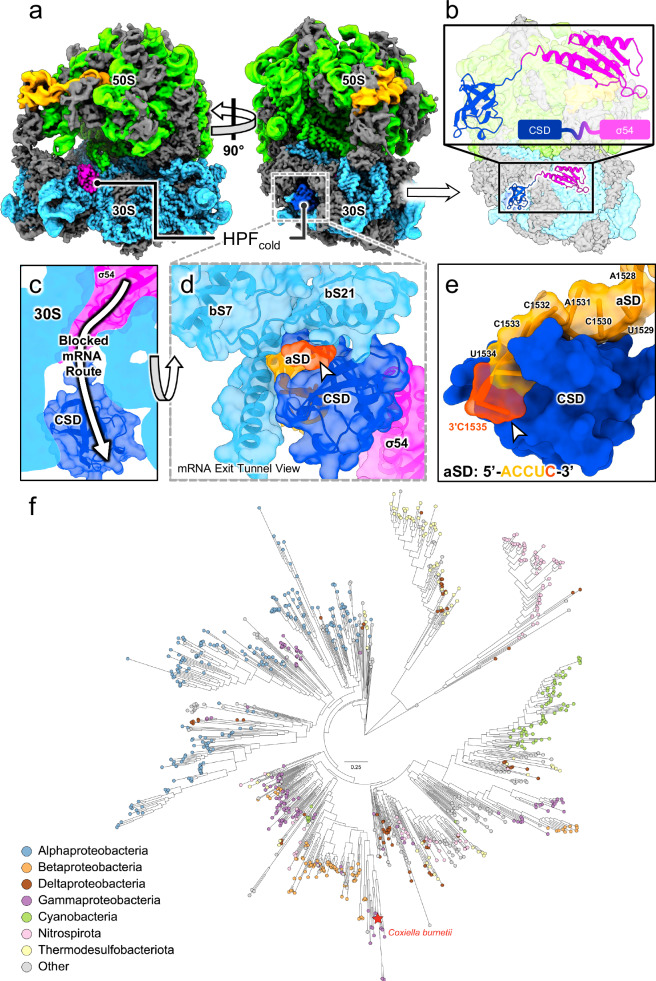
HPF_cold_, a distinct member of the hibernation factor family. **a** A hibernation promoting factor (HPF_cold_; magenta and dark blue) was observed bound within the 30S decoding centre of the *C. burnetii* ribosome. Ribosomal proteins are coloured grey, whilst 5S, 16S and 23S rRNA are coloured light orange, blue and green respectively. **b** HPF_cold_ (*CBU_0020*) consists of two domains, an N-terminal sigma-54 modulation factor domain (PF02482, σ54; magenta) and a C-terminal cold-shock domain (PF00313, CSD; dark blue). **c** HPF_cold_ occupies the mRNA route through the ribosome, preventing translation. **d** The cold shock domain binds the anti-Shine Dalgarno sequence on the 16S rRNA (orange/red). Ribosomal proteins uS7 and bS21 (pale blue) coordinate the CSD. Proteins shown with a semi-transparent surface and a cartoon representation. **e** The anti-Shine-Dalgarno (aSD) sequence (ACCUC) makes extensive interactions with the CSD. The aSD is at the 3’ end of the 16S rRNA and so is susceptible to RNase activity if not protected. CSD is shown as a surface, 16S rRNA as a semi-transparent surface and a cartoon representation. **f** Maximum-likelihood phylogeny of fused PF02482–PF00313 two-domain proteins across bacterial lineages. Unrooted ML tree of proteins containing both Pfam domains PF02482 (σ54) and PF00313 (CSD). Tips are coloured by major lineages (Alphaproteobacteria, Betaproteobacteria, Gammaproteobacteria, Deltaproteobacteria, Cyanobacteria, Nitrospirota, Thermodesulfobacteriota, Other). *Coxiella* sequences are highlighted. The alignment was built with MAFFT (G-INS-i)^86^ and trimmed with trimAl (gappyout)^87^; the best-fit model was selected with ModelFinder^89^, and branch support was estimated with 10,000 ultrafast bootstrap replicates in IQ-TREE^88^. The scale bar denotes 0.25 substitutions/site. Tree visualisation was done in iTOL^93^. Source data for panel f are provided as a Source Data file.

The *C. burnetii* 70S structure revealed a protein bound to the 30S tRNA cleft (Fig. 2a-b, Supplementary Movie 1). This was identified as *CBU_0020*, one of two hibernation factors present in the *C. burnetii* genome. *CBU_0020* is a ~20 kDa protein consisting of two domains joined by a six-residue linker. The N-terminal domain has a sigma-54 fold (Pfam PF02482) and the C-terminal domain a cold shock domain fold (CSD, Pfam PF00313) (Fig. 2b). *CBU_0020* will subsequently be referred to as HPF_cold_. A recent pan-genome analysis of HPFs also identified HPF_cold_ as a prokaryotic hibernation factor^30^.

The HPF_cold_ CSD domain occupies the mRNA exit tunnel (Fig. 2c), binding and protecting the 16S anti-Shine Dalgarno (aSD) sequence (Fig. 2d, e, Supplementary Fig. 3a, b). The aSD wraps around the CSD, making a combination of stacking interactions, hydrogen bonds and charge interactions (Supplementary Fig. 3b, c). The CSD is also pincered by the α-helical arms of bS21 and contacted by bS7 (Fig. 2d, Supplementary Fig. 3d).

We have identified HPF_cold_ in all major proteobacterial classes, including Cyanobacteria, and several non-proteobacterial phyla (Fig. 2f, Supplementary Discussion). A Protein Data Bank (PDB) BLAST^31,32^ search of the HPF_cold_ CSD revealed no substantial sequence similarity to any existing CSD structure. Strong structural similarity to bacterial cold shock proteins is retained, evidenced with a structural alignment RMSD of 1.8 Å against the full *E. coli* CspA, despite low sequence similarity overall (Supplementary Figs. 4a, d, 5a)^33^. A Foldseek search^34^ of *E. coli* CspA against the *C. burnetii* genome returned HPF_cold_ with the highest E-value and no dedicated cold shock protein family members, indicating these have been lost. Bacterial hibernation factor sequence alignments show conserved positively charged residues in the sigma-54 domain are retained between HPF_cold_ and existing members of the family (Supplementary Fig. 5b). Sequence conservation analysis of HPF_cold_ revealed that the CSD shows higher average conservation than the sigma-54 domain (Supplementary Fig. 6a). Conservation was strongly clustered to the aSD interacting residues (Supplementary Fig. 6b, 7a–c) while the sigma-54 domain shows surface conservation around a coordinated magnesium ion (Supplementary Fig. 7d, e).

### A C. burnetii-specific ribosome-binding protein

**Fig. 3:**
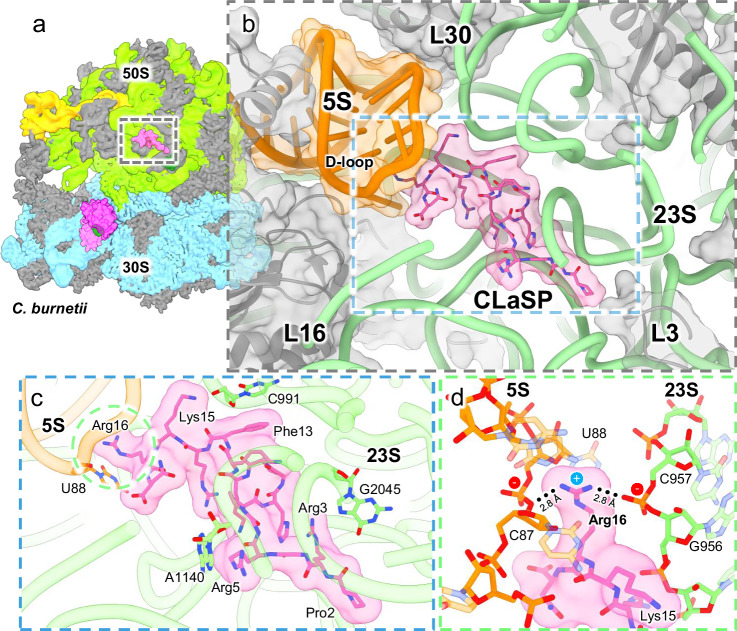
CLaSP: A ribosomal protein unique to *Coxiellaceae.* **a** Unidentified density in the large subunit was modelled *de-novo* and identified as an undescribed gene, termed CLaSP (*Coxiellaceae* large subunit peptide). Proteins are coloured grey, whilst 16S, 23S and 5S rRNA are coloured blue, green and orange respectively. CLaSP and HPF_cold_ (Fig. 2) are shown in magenta. **b** CLaSP shows an extended conformation and occupies a cleft between the 5S rRNA D-loop and the 23S rRNA. 5S rRNA and ribosomal proteins are shown as a transparent surface and cartoon representation in orange and grey, respectively, CLaSP as sticks with a transparent surface in pink, and 23S rRNA as a green tube. **c** CLaSP is a basic peptide, making extensive charge-charge interactions to the surrounding phosphate backbone. Specific stacking interactions are shown with rRNA bases as sticks. **d** Arg16 acts as a key coordinating residue, making interactions with the 5S and 23S rRNA backbones. rRNA shown as sticks.

During model building, we observed additional density that could not be attributed to any known ribosomal protein in the PDB. This density located to the cleft formed by domains II and V of the 23S rRNA, together with the D loop of 5S rRNA (Fig. 3a,b; Supplementary Fig. 8a,b, Supplementary Movie 1) and was of sufficient quality to assign a 16 amino acid peptide sequence *de-novo* from the cryo-EM map. A BLAST search^32^ of this peptide identified a unique hit within the *C. burnetii* genome located one base 3’ downstream from the ribosomal RNA transcription regulator *nusB* (Supplementary Fig. 8c). This open reading frame encodes a 22-residue peptide (residues 2-17 are visible in the cryo-EM map) unique to *Coxiellaceae*, which we therefore name CLaSP (*Coxiellaceae* Large Subunit Peptide). CLaSP connects the shorter *C. burnetii* 5S rRNA to the 23S rRNA, providing a direct contact between the rRNA molecules (Fig. 3c, d, Supplementary Fig. 8).

### Doxycycline binds the canonical small subunit site in both E. coli and C. burnetii

As expected, we observe doxycycline bound in the classical 30S binding site in the tRNA decoding region for both *C. burnetii* and *E. coli* ribosomes (Supplementary Fig. 1a, b). This binding site is identical to previous 30S tetracycline-bound structures, where two coordinating magnesium ions and ring D of doxycycline stacks upon 16S C1054^24^. This prevents tRNA A site binding, a mechanism shared with ribosomal hibernation promoting factors. Indeed, in our models, doxycycline overlapped with bound hibernation factors in both species: HPF_cold_ in *C. burnetii* and RaiA in the *E. coli* ribosome. We therefore separated antibiotic-bound particles from hibernation factor-bound particles for both species using focused classification methods. This improved the density for the bound antibiotic (Supplementary Fig. 9 and 10) and agrees with the observation in *E. coli* that 30S-bound tetracyclines compete directly with tRNA-binding site hibernation factors^20^.

### Doxycycline blocks the C. burnetii nascent peptide tunnel

**Fig. 4:**
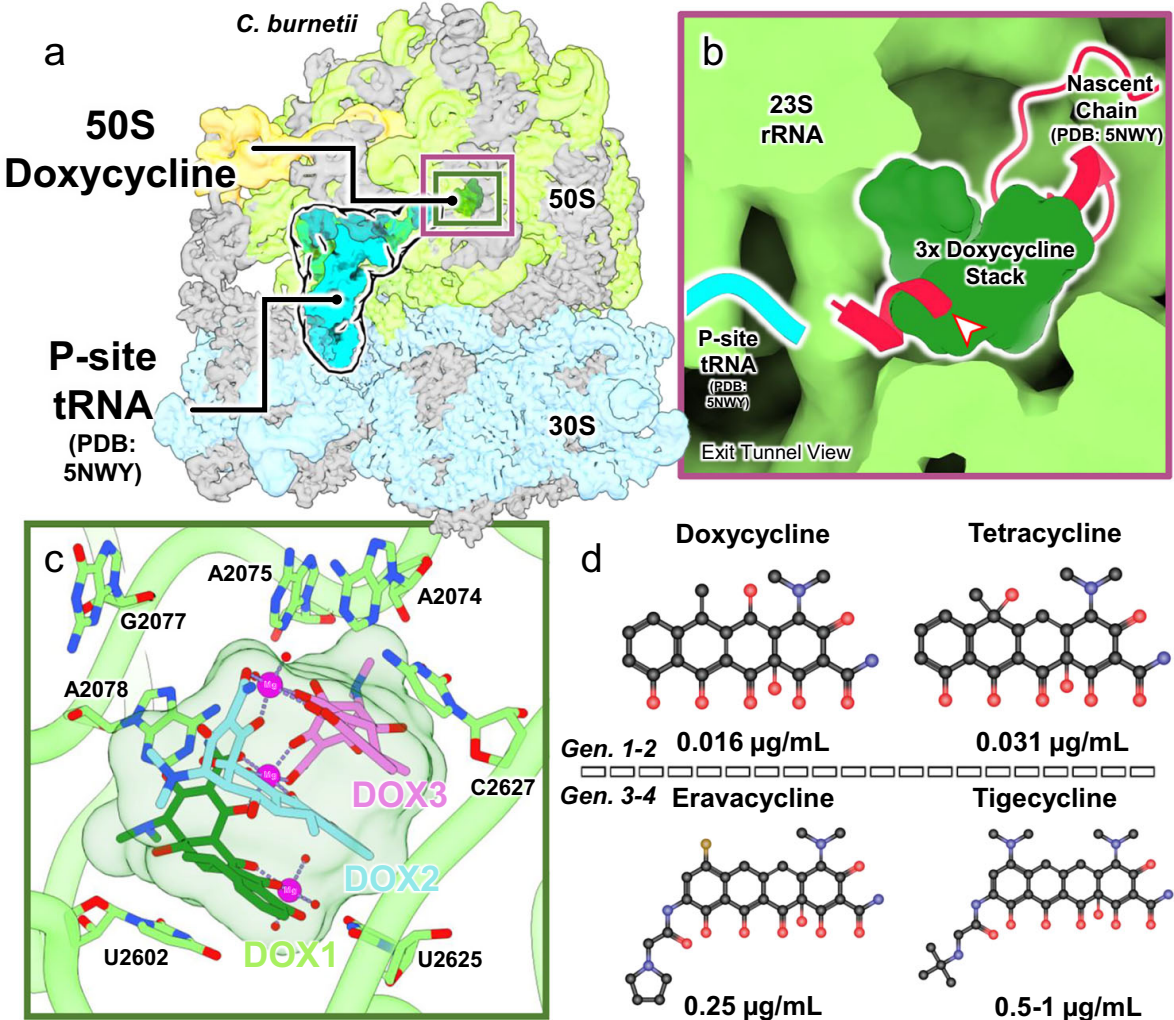
Three doxycycline molecules stack within the *C. burnetii* exit tunnel. **a** Overview of the doxycycline-bound *C. burnetii* ribosome. All molecules shown as a surface. Proteins are coloured grey, whilst 16S and 23S rRNA are coloured blue and green, respectively. P-site tRNA (PDB: 5nwy^94^,) is shown in cyan blue and the stack of three doxycycline molecules in dark green (sphere atom representation). **b** Superposition of the two structures shows that doxycycline completely blocks the nascent chain channel. Cut-through image of the ribosome shown, with a nascent chain and P-site tRNA (both PDB: 5nwy^94^) as cartoon representations in crimson and cyan, respectively. (**c**) Three molecules of doxycycline form a stack between bases U2602 and C2627. rRNA shown as a cartoon with interacting bases as sticks; doxycycline molecules shown as sticks coloured green, cyan, and magenta for molecules 1, 2, and 3, respectively. The doxycycline stack is coordinated around three magnesium ions (magenta spheres) that interact with doxycycline oxygen atoms and waters (not shown). **d** Later generation tetracyclines, which display higher inhibitory values in other species, perform worse than older, more compact chemicals. These may be unable to fit within the early exit tunnel in the same manner as doxycycline, suggesting the additional exit tunnel blocking mode of action might contribute towards the unusual potency of doxycycline against *C. burnetii*.

We observed a remarkable stack of three doxycycline molecules bound in the exit tunnel of the *C. burnetii* ribosome, which we number by increasing distance from the peptidyl transferase centre (PTC; Fig. 4a–c, Supplementary Fig. 11a, Supplementary Movie 1). Similar doxycycline stacks were recently observed in the exit tunnel of the *E. coli* and *Cutibacterium acnes* ribosomes at higher concentrations by Devarkar et al.^27^ This three-molecule *C. burnetii* binding is an elaboration of a similar 50S binding mode first observed for a single sarecycline molecule against *C. acnes*^35^ (Supplementary Fig. 12c–e). Doxycycline molecule one (DOX1) stacks upon the U1796:U2602 base pair (1782:2586, *E. coli* numbering in parentheses; Supplementary Fig. 11a). The two other doxycycline molecules stack consecutively on top of the first, with their ‘C’ rings stacked upon the ‘D’ ring of the preceding molecule (Fig. 4c). DOX2 is rotated by approximately 90° around the stack axis with respect to DOX1 and DOX3. This arrangement facilitates interactions with three magnesium ions that are key to the three-molecule binding (Supplementary Fig. 12a). The central magnesium ion is coordinated by two oxygen atoms from each of the three doxycycline molecules. DOX1 coordinates a second magnesium ion together with U2625 (U2609), while DOX2 and DOX3 coordinate a third magnesium (Fig. 4c, Supplementary Fig. 12a). The amide group of DOX1 holds in position the flexible ribosome stall sensor A2078 (A2062) in the tunnel protruding conformation (Fig. 4c, Supplementary Fig. 11e,12a). DOX3 is also closely associated with the edge of the tunnel (Fig. 4c). Here, a magnesium coordinated water forms an H-bond with A2074 (A2058; Supplementary Fig. 12g). Comparison between bound and unbound ribosomes (Supplementary Fig. 11a, b) revealed that DOX2 stabilises U2522 (U2506; Supplementary Fig. 11c-d) and G2073-A2075 (G2057-A2059) have additional density in the empty state, likely corresponding to bound waters that are displaced on doxycycline binding (Supplementary Fig. 11f-g). The interaction between DOX2 and U2522 stabilises the base in a luminal, translationally stalled-like position (Supplementary Fig. 11c). The 23S rRNA nucleotides that contact doxycycline are absolutely conserved in all available *C. burnetii* genomes and are strongly conserved across a wide range of bacteria (Supplementary Fig. 13). The development of later generation tetracyclines has elaborated the underlying four-ring structure with various chemical decorations (Fig. 4d). We reasoned that the lack of additional chemical elaboration on the doxycycline rings, compared to the bulkier later generation tetracyclines, may facilitate this stacked binding mode (Fig. 4d, Supplementary Fig. 12b). Minimum inhibitory concentration (MIC) experiments were carried out against *C. burnetii* (strain NMII) with four tetracycline derivatives: tetracycline, doxycycline, tigecycline and eravacycline. Tigecycline and eravacycline are third and fourth-generation tetracyclines, respectively, with chemical additions on C9 (Fig. 4d, Supplementary Fig. 12c–f). Doxycycline retained the lowest MIC at 0.016 µg/ml and, together with the similarly minimal tetracycline molecule (0.03 µg/ml), substantially outperformed both eravacycline (0.25 µg/ml) and tigecycline (0.5–1 µg/ml) (Supplementary Fig. 14). As MIC results are influenced by a complex network of factors, a direct causation cannot be drawn between our structural and inhibitory data.

### A rearranged peptidyl transferase centre in the E. coli ribosome

**Fig. 5:**
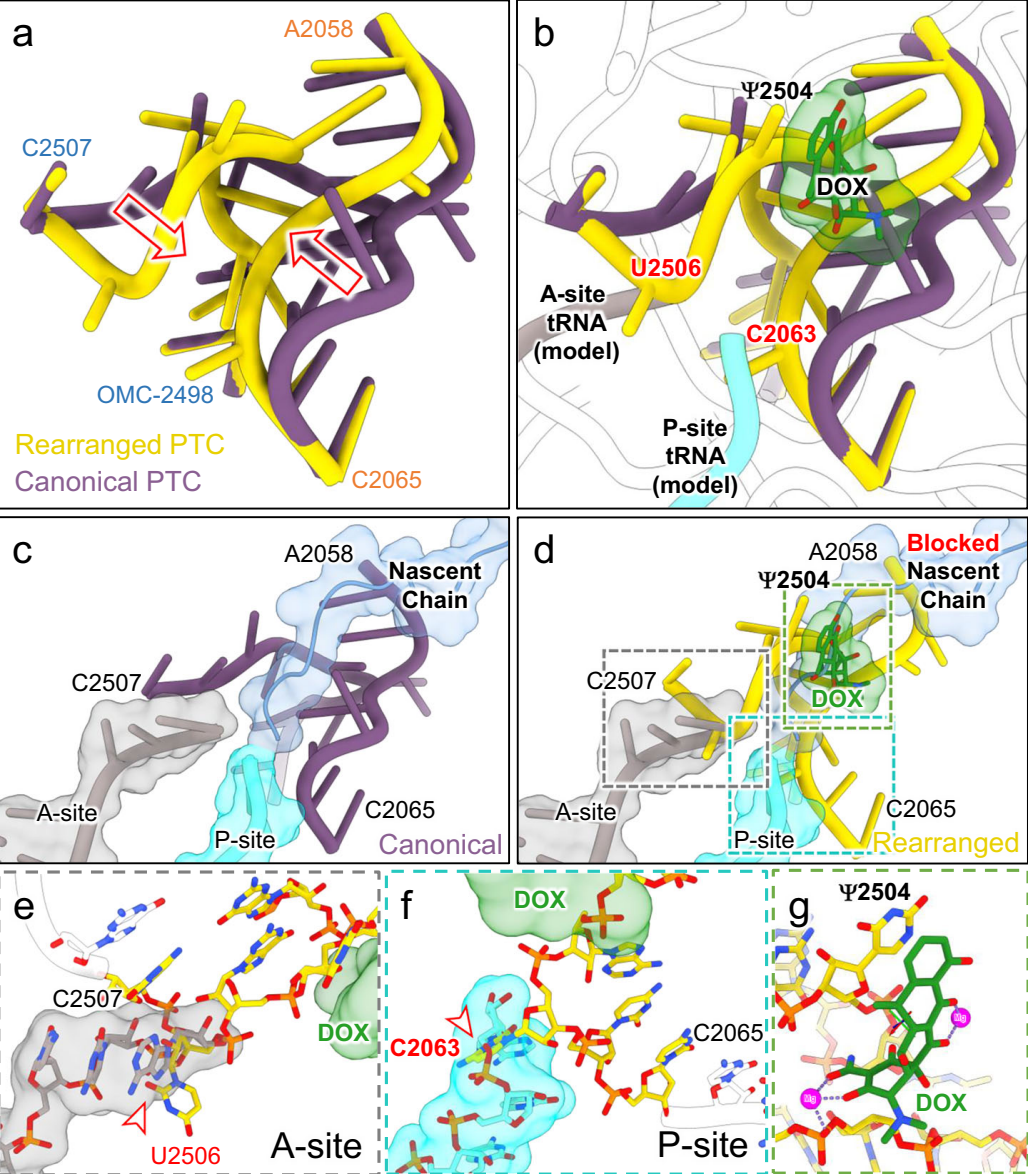
A rearranged peptidyl transferase centre. A subset of our *E. coli* data revealed a remarkable conformation of the prokaryotic ribosome bound to doxycycline. **a** Doxycycline drives a fundamental reorganisation of the 23S rRNA between bases 2058-2065 and 2498-2507 at the peptidyl transfer centre (PTC). rRNA is shown in cartoon representation, with the canonical structure in purple and the rearranged structure in yellow. The direction of movement is indicated by red arrows. **b** Doxycycline interacts with the rearranged PTC, stabilising this conformation that may be sampled at low occupancy in normal *E. coli* ribosomes. Doxycycline is shown as sticks with transparent surface, carbon atoms, green; oxygen, red; nitrogen, blue. The position of the P-site tRNA from the active *E. coli* ribosome (PDB: 7k00^75^) is shown as a cyan cartoon. OMC: O2’-methylcytidine. **c** Bound doxycycline occupies the location typically held by the nascent chain (blue cartoon and transparent surface) in the canonical, translating structure (PDB: 7k00^75^). **d** The rearranged PTC rRNA clashes with both A-site (mauve cartoon and transparent surface) and P-site tRNAs. This rearrangement will therefore prevent tRNA binding, peptide transfer, and the exit of a nascent chain. **e** The A-site tRNA is blocked by base U2506, which kinks outward from the exit tunnel in the rearranged PTC. rRNA and A-site tRNA shown as sticks; rRNA carbon, yellow, A-site tRNA carbon, mauve; phosphate, orange. **f** The P-site tRNA is blocked by base C2063 flipping outward from the exit tunnel. tRNA shown as sticks with transparent surface, carbon and surface cyan. **g** Detail of doxycycline interaction with rRNA. Doxycycline coordinates two magnesium ions with O11-O12 and O3-O21, respectively. Ring ‘D’ stacks upon pseudouridine Ψ2504.

Next, we attempted to identify whether doxycycline occupied the *E. coli* exit tunnel in a comparable way. 3D classification was carried out on the *E. coli* cryo-EM dataset, focusing on the *C. burnetii* doxycycline 50S binding site. The majority of particles showed the expected large subunit structure and in contrast to our *C. burnetii* data, only a weak density of one molecule (corresponding to DOX1) was observed in the *E. coli* ribosome (Supplementary Fig 1d). However, ~12% of particles showed a markedly different structure. Refinement of these yielded a 2.16 Å structure showing a single bound doxycycline and major rearrangement of the tertiary structure of the PTC and early nascent chain exit tunnel between C2498-G2508 and G2056-C2064 (Fig. 5a, b, Supplementary Fig. 15a). This rearrangement resembled a substantial zippering of these regions of rRNA (Supplementary Movie 1). The early exit tunnel is typically lined with bases, however in this zippered structure, these nucleotides are inverted so that the tunnel lumen is instead lined by phosphate backbone (Supplementary Fig. 15). Doxycycline stacks on Ψ2504, with O3 and O21 anchoring the antibiotic via a magnesium to the phosphate of A2062, pinching both backbones together (Fig. 5a, b, g, Supplementary Movie 1). The PTC is also now closed, preventing accommodation of tRNA in either the P or A site (Fig. 5a–e) or nascent peptide. Notably, both U2506 (Fig. 5e) and C2063 (Fig. 5f) are splayed outwards, forming new interactions (Supplementary Fig. 15c) and clashing with incoming P-site and A-site tRNAs. Other key rearrangements include A2062 flipping in, away from the PTC to base pair with dihydrouridine 2449 and, via a non-canonical base pair, with A2450 (Supplementary Fig. 16g). The A2450–C2063 base pair essential for translation is abolished^36^. Many additional new interactions are present, which we describe further in the Supplementary Discussion (Supplementary Fig. 16). Density for ribosomal protein uL4 is also notably poorer in the rearranged form than the canonical structure, due to a movement of the backbone around A2060, preventing the extended loop from anchoring against the phosphate backbone (Supplementary Fig. 15b).

## Discussion

HPF_cold_ provides the structure of a hibernation factor with a cold shock domain, revealing an additional member of the prokaryotic ribosome hibernation factor family^37^. Homologous cold shock domains are present across a wide range of bacterial phyla, particularly the proteobacteria, suggesting either an ancient origin with subsequent losses or horizontal gene transfer. Indeed, a very recent detailed phylogenetic analysis of prokaryotic hibernation factor genes identified HPF_cold_ and HPF-SigE, both products of fusion events^30^. Since it is structurally distinct from existing HPF family members (HPF_long_, HPF_short_, ribosome-associated inhibitor A, RaiA; Supplementary Fig. 4e) and the recently discovered Balon hibernation factor^37,38^ we propose to name this class HPF_cold_. HPF_cold_, like other members of the HPF family, occupies the A site, blocking tRNA entry. HPF members differ in their effect on the ribosome, either stabilising a single ribosome (70S hibernation) or recruiting a partner ribosome to achieve ribosome dimers (100S hibernation)^37^. HPF_short_ functions in combination with ribosome modulation factor (RMF). Both RMF^39^ and HPF_long_^40^ drive the formation of 100S ribosome dimers that protect the mRNA exit tunnel from RNases, particularly the 3’ end of the 16S rRNA, aiding survival and increasing virulence^41^. The 3’ end of the 16S rRNA contains the anti-Shine-Dalgarno sequence. This is particularly sensitive to RNases as a surface-exposed terminal sequence, and loss of the aSD sequence will obviate ribosome function^21^. *C. burnetii* lacks HPF_long_, RaiA, and RMF orthologues (its other hibernation factor (*CBU_0745*) having an HPF short structure, retained for shorter-term hibernation)^39^. However, HPF_cold_ protects the 3’ end of the 16S rRNA by binding and stabilising the anti-Shine Dalgarno sequence through the CSD, blocking RNase access to the hibernating ribosome. This likely confers a high enough degree of protection from RNases to keep 70S ribosomes viable during the extended hibernation periods required of the *C. burnetii* SCV form^42^.

We discovered a distinct peptide (CLaSP) closely associated with both the 23S and 5S rRNA. The 5S rRNA of *C. burnetii* shows a more compact conformation and therefore reduced protrusion into this cleft compared to *E. coli* (Supplementary Fig. 8a). The direct contact of CLaSP therefore, may suggest a role in ribosome assembly and 23S and 5S rRNA stability^43,44^. The position of CLaSP overlaps with that of the mycobacterial specific ribosomal protein bL37 (Supplementary Fig. 8b)^45^, suggesting a possible case of convergent evolution. Given its proximity to the PTC, bL37 has been hypothesised to contribute to ribosome stability, and we speculate that CLaSP may perform a similar function^32^. This is further supported by the location of the CLaSP open reading frame, located close to *nusB*, required for ribosome maturation^44^ (Supplementary Fig. 8c). CLaSP does not share substantial sequence identity or structural characteristics with bL37. Indeed, superposing the structures indicates that a CLaSP-like peptide would not bind to the 5S rRNA of *Mycobacteria*. CLaSP is structurally dissimilar to the uL30 extension from *Borrelia burgdorferi*, which occupies the same cleft^46^. Finally, it is possible that CLaSP may mitigate the general reduction in modified ribosomal nucleotides we observe in the *C. burnetii* ribosome, compared to *E. coli* (Supplementary Fig. 2)^47,48^, increasing the efficiency of this reduced ribosome. The hypothesis that there has been pressure to eliminate unnecessary modifications is supported by the remaining *C. burnetii* rRNA modification genes mapping precisely to those previously identified as providing the most substantial growth rescue in an *E. coli* multiple gene knockout^49^.

The *C. burnetii* ribosomal exit tunnel in the 50S subunit is filled by a remarkable stack of three doxycycline molecules (Fig. 4a–c), blocking nascent peptide exit (as observed for other antibiotic classes^50^). This stack stabilises U2522 (U2506) (Supplementary Fig. 11c) and A2078 (A2062) (Supplementary Fig. 11e), which sense ribosome stalling^51^ and translation arrest^52,53^. The tetracycline-like antimicrobials sarecycline and tetracenomycin X also bind to the exit tunnel^35,50^, in a similar area to DOX1 (Supplementary Fig. 12c-e). However, they are rotated with respect to DOX1 and so could not accommodate the three-molecule stack or magnesium coordination. This doxycycline triple stack therefore, represents an additional mode of antibiotic action that has greater similarity to the macrolide mode of action (Supplementary Discussion). During the preparation of this work, Devarkar et al. observed doxycycline complexes binding in the *C. acnes* and *E. coli* ribosomal exit tunnels at higher doxycycline concentrations^27^. This was not observed for either minocycline or sarecycline against the same organisms. We and Devarkar et al. could not resolve a doxycycline stack in *E. coli* at concentrations below 100 µM.

A major driver for the development of later-generation tetracyclines was maintaining or improving binding to the small subunit. Eravacycline has a ~14 times greater binding affinity for the 30S subunit than tetracycline in *E. coli*^54^. The 30S binding site is present in the *C. burnetii* ribosome; the smaller early generation tetracyclines show much greater potency against *C. burnetii*. This suggests that the binding in the large subunit, which can only accommodate the smaller tetracyclines, may contribute towards the efficacy of doxycycline against this pathogen. However, further work is required to elucidate the relative importance of the two sites. The ability to bind both large and small subunits presents the opportunity for complementarity and synergy, increasing the barrier to resistance mutations. However, it should be noted that some strains reporting reduced sensitivity to doxycycline have been reported^55^. Our results suggest that the traditional focus on optimising tetracycline binding to the small subunit may not improve antimicrobial activity universally.

We observed doxycycline bound to the *E. coli* peptidyl transferase centre, the latter in a distinct conformation (Fig. 5). The peptidyl transferase centre shows only limited backbone conformational variation between ribosomal states or between the domains of life^46,56–59^. The structure we have captured reveals that the prokaryotic ribosome is capable of a substantial rearrangement within this highly conserved, ancient region. Of particular note is the abolition of the essential A2450-C2063 interaction. Although we observed this conformation in only a subset of purified ribosome particles, it is possible that it is sampled more frequently in the cell than in purified ribosomes. The altered conformation will block the tip of the tRNA acceptor stem from the P and A sites and would be unable to accommodate the nascent chain (Fig. 5c-f, Supplementary Movie 1). The other major change is disordering of residues 60-65 of the uL4 loop (Supplementary Fig. 15b). Deletion of this loop has previously been shown to slow translation and disrupt ribosome formation^60^. Alterations of the uL4 loop negatively impact translation fidelity and confer erythromycin resistance^61,62^. Evidently, this ribosome conformation will be severely impaired and is likely inactive. This suggests an explanation for the bactericidal properties of doxycycline observed against *E. coli* strain ED1a, which shows particular bactericidal sensitivity to doxycycline^63^. In another recent study, ED1a did not show a lowered MIC value against tetracycline compared to other *E. coli* strains, but bactericidal activity remained, appearing to arise from an effect against the ribosome itself^64^. Thermal proteomic profiling results comparing *E. coli* ED1a (bactericidal) and *E. coli* K12 (bacteriostatic) showed the main divergence arising from the large subunit of ED1a ribosomes. While large subunit binding was not identified in that study, the classification required of our data suggests it could occur in a subset of ribosomes, making it difficult to identify. This inactive conformation of the ribosome presents an attractive avenue for future antibiotic development. Modification of doxycycline to exploit the properties of this site could lead to a potent inhibitor, potentially with bactericidal properties.

In conclusion, we have identified two distinct binding modes of doxycycline to the ribosome large subunit in distinct bacterial species. One suggests an explanation for the proclivity of doxycycline for the *C. burnetii* ribosome, while the other reveals an inactive ribosome conformation in *E. coli*. The modes of inhibition are mutually exclusive – one fills the nascent chain exit tunnel and the other remodels it. In addition, we describe a *C. burnetii* gene encoding a ribosomal peptide and a distinct member of the prokaryotic hibernation factor family, HPF_cold._ In a climate of increasing antimicrobial resistance, these offer new routes for antibiotic development.

## Methods

### E. coli ribosome isolation

400 ml BL21 DE3 (Merck #69450-3) *Escherichia coli* cells were grown to OD_600_ 0.7 and isolated through centrifugation at 4000 × g for 15 minutes at 20 °C. Pelleted cells were resuspended in 7 ml filtered and autoclaved BS100 buffer containing: 25 mM HEPES pH 7.5, 100 mM KOAc, 15 mM Mg(OAc)_2_, 1 mM DTT (added prior to each use) and frozen at −20 °C.

Thawed cells were lysed in a Precellys 24 homogeniser, utilising VK01 2 ml tubes with 0.1 mm zircon beads. Samples were lysed with 8 × 20 s beating at 6500 rpm and 5 minutes of cooling on ice between each run. Supernatant was collected and beads were washed with buffer to elute additional lysate. Cell debris were subsequently clarified by centrifugation at 9000 × g for 15 minutes at 4 °C.

Ribosome purification followed previously described methods^65^. Supernatant was layered over a 30% (w/v) sucrose in BS100 cushion and centrifuged at 206,000 × *g* for 3 hours at 4 °C. The ribosome pellet was resuspended in 500 µL BS100 buffer through shaking at 450 rpm at 4 °C for 90 minutes using a Stuart Scientific SA8 vortex mixer (Merck #Z648531). Suspension was layered over a 10–40% (w/v) BS100 sucrose gradient and centrifuged at 125,000 × g for 80 min at 4 °C. Cushion was manually aspirated into 500 µL fractions, and 70S fractions were pooled and concentrated in a 100 kDa Vivaspin concentrator (Sartorius VS0142). Purified samples were snap frozen in liquid nitrogen and stored at −80 °C.

### E. coli ribosome grid preparation

Purified ribosome samples were diluted to approximately 0.3 mg/ml (112.5 nM) and incubated with a final concentration of 50 µM doxycycline hyclate (Merck #D9891) for one hour on ice. 3 µl ribosome antibiotic complex was added to a Quantifoil R2/2 2 nm continuous carbon coated grid (Agar Scientific, AGS173-2-2CL), blotted for 4 s at 4 °C, 100% humidity before plunge freezing with a Vitrobot Mark IV (ThermoFisher Scientific).

Electron micrographs were collected at the electron biological imaging centre (eBIC) using Krios I (FEI Titan Krios, ThermoFisher) with a K3 detector (Gatan). 34,720 micrographs were collected at 130 k magnification with a pixel size of 0.615 Å and a total exposure of 45 e^-^/Å^2^.

### E. coli doxycycline-bound structure data processing

Raw data were imported into cryoSPARC (v4) for patch motion correction and CTF estimation. 2556 low-quality micrographs (CTF fit greater than 5 Å) were removed. Data were initially processed in two batches in parallel. 773,916 particles were picked from Batch 1 (17,587 micrographs) and subject to two rounds of 2D classification (4x binning, 2.6 Å/pix). The resulting 416,629 particles were subject to ab-initio classification. 391,620 particles were re-extracted with 2x binning (1.3 Å/pix) yielding a reconstruction at the Nyquist limit (2.6 Å). Batch 2 (14,523 micrographs) blob picking yielded 1,462,230 particles, reducing to 967,764 following one round of 2D classification. All blob-picked particles were then pooled and combined with crYOLO general model particle picks, subject to duplicate removal and 2,257,467 particles were subject to further 2D classification. Ab-initio and heterogeneous refinement were used to assess particle quality and select for high-quality 70S ribosomes. This yielded 1,747,354 million particles, which were re-extracted at 0.8 Å/pix. Homogenous refinement yielded a 70S ribosome at 2.11 Å, improving to 1.95 Å using non-uniform refinement. Reference-based motion correction followed by non-uniform refinement with global and local CTF-refinement resulted in a 1.77 Å 70S reconstruction. Local refinement of the 30S subunit with the same particle set yielded a 1.94 Å reconstruction.

Classification was carried out on the hibernation factor binding site to separate doxycycline and YfiA-bound ribosomes. A mask around YfiA was created and 3D classification without alignment was used to classify based on occupancy. 1,068,443 particles were in the YfiA-bound class and 678,911 in the doxycycline/tRNA-bound class. A local refinement of the YfiA class gave a map refined to 1.94 Å. A repeated classification (same parameters) on the 678,911 doxycycline/tRNA class selected 306,491 particles bound to doxycycline without tRNA and 372,420 with both present. Local refinements resolved to 2.13 Å without tRNA and 2.08 Å with tRNA; however, antibiotic density was more easily interpretable in the doxycycline with tRNA class, so this refinement was utilised for model building.

Following identification of the 50S doxycycline binding in *C. burnetii* focused classification was performed on the peptidyl transferase centre in *E. coli*. This utilised a mask around the three antibiotic molecules and 10 classes with a high-resolution filter (3 Å). A processing flowchart (Supplementary Fig. 10) and all Fourier shell correlation (FSC) curves, particle angular distribution plots and local resolution maps (Supplementary Fig. 17-20) are provided.

### C. burnetii cell lysis

mCherry-NMII *C. burnetii* cells^66^ (inoculated with 100 µl 1×10^7^ stock) were grown in 4×50 ml 1x ACCM-2 (Sunrise Bioscience #4700-300) in Nunc 50 ml flasks (Thermo Scientific, #156367) under 2.5 % O_2_, 5% CO_2_ (referred to subsequently as low oxygen) at 37 °C^67^. After 7 days of growth, cells were harvested into 50 ml Falcon tubes and pelleted by centrifugation at 8,000 x*g* for 20 minutes at room temperature. Pellets were resuspended in BS100 and pooled to a final volume of ~8 ml before storage at −80 °C. Cell lysis was carried out using bead beating (Precellys 24, 8 × 1 ml added to VK01 beads, Bertin Corp.) to eliminate the risk of aerosolisation that can arise with other lysis methods. Lysis protocol carried out as described above with *E. coli* except each 1 ml lysate sample was kept separate for a subsequent sterility check (below). Lysate was subject to centrifugation at 18,000 x* g* for one hour to pellet any remaining cells and filtered through a 0.22 µM filter. Sample not used for sterility test was split into 100 microlitre aliquots in 0.5 ml Eppendorf tubes, snap frozen in liquid nitrogen and stored at −80 °C.

### C. burnetii sterility check

100 microlitres of each lysate fraction was inoculated into 5 ml ACCM-2 and incubated statically for 7 days at 37 °C under low oxygen. A positive (20 microlitres at 2 × 10^10^ cfu/ml, −80 °C stock) and negative control (100 microlitres ACCM-2 alone) were used. After 7 days, ACCM-2 0.25% (w/v) agar plates were inoculated with 250 microlitres of culture, incubated for 10 days at 37 °C under low oxygen and assessed for growth.

### C. burnetii ribosome isolation

Post-sterility check pooled lysate was layered onto a 30% (w/v) sucrose BS100 cushion and centrifuged at 206,000 × g for 3 hours at 4 °C. The ribosome pellet was resuspended in 500 µL BS100 buffer supplemented with 300 nM doxycycline, shaking at 450 rpm at 4 °C for 90 minutes as for *E. coli*. Ribosomes were concentrated to 0.05 mg/ml using a 100 kDa Vivaspin concentrator (Sartorius VS0142) and snap frozen in liquid nitrogen for −80 °C storage.

### C. burnetii ribosome grid preparation and data collection

Ribosomes were incubated with doxycycline hyclate (Merck #D9891) at a final concentration of 50 µM for one hour on ice. Grids were frozen as for *E. coli*.

Electron micrographs were collected at the electron biological imaging centre (eBIC) using Krios IV (FEI Titan Krios) with a K3 detector (Gatan). 22,295 micrographs were collected at 81 k magnification with a pixel size of 1.06 Å and a total exposure of 45 e^-^/Å^2^.

### C. burnetii doxycycline-bound structure data processing

Raw data were imported into cryoSPARC (v4) for patch motion correction and CTF estimation^68–70^. A crYOLO convolutional neural network model was trained with blob-picked particles cleaned using 2D classification^71^. 5,543,101 crYOLO picked particles were extracted with 4x binning and subject to four rounds of 2D classification, resulting in 304,935 particles. One round of ab-initio 3D classification and a final 2D classification yielded 221,552 final particles for refinement. Particles were un-binned and refined to 2.71 Å with homogenous refinement, improving to 2.40 Å with non-uniform refinement^72^. Particles were separated by exposure group and subject to global CTF refinement followed by reference-based motion correction. Non-uniform refinement gave a final 50S reconstruction to 2.15 Å.

Focused classification on the 50S doxycycline binding site was carried out as above for *E. coli*. This resulted in 91,218 50S antibiotic-bound and 129,328 empty ribosomes. These were refined to 2.22 Å and 2.19 Å respectively, using non-uniform refinement. Despite further classification on the 50S, an *E. coli-*like rearranged ribosome class could not be identified.

Focused 3D classification without alignment isolated 44,196 70S ribosome particles, giving a 2.48 Å 70S non-uniform reconstruction and a 2.83 Å local refinement of the 30S subunit. Focused classification without alignment was carried out with a mask around HPF_cold_, as for *E. coli*. This identified 24,143 HPFcold-bound ribosomes and 20,053 30S doxycycline and tRNA-bound ribosomes. The HPF_cold-_bound ribosome refined to 2.87 Å with local refinement. As for *E. coli*, a further round of classification of the doxycycline-bound particles removed non-tRNA-bound ribosomes, which displayed poorer quality density. This resulted in a 3.02 Å local refinement of doxycycline bound to the 30S from 11k particles. A processing flowchart (Supplementary Fig. 9), all Fourier shell correlation curves, particle angular distribution plots and local resolution maps are provided (Supplementary Figs. 17–20).

### Atomic model building

*E. coli* model building was carried out in both Coot and Isolde^73,74^ using the published 2 Å *E. coli* structure (PDB 7k00) as a starting model^75^. Waters and magnesium ions were manually checked and built where necessary. Final refinements were carried out with Refmac v5.8^76^ and Phenix v1.21^77^.

*C. burnetii* AlphaFold v2.2^78^ ribosomal protein predictions were rigid body fitted into electron density using ChimeraX v1.7^79^ and rebuilt using Coot and Isolde^73,74^. ModelAngelo^80^ was used to provide an initial template for rRNA, which was rebuilt and sequence corrected as necessary. 5S rRNA and L1 stalk AlphaFold 3^81^ predictions were incorporated into the structure and rebuilt. Where it was not possible to confidently identify an rRNA post-transcriptional modification from the cryo-EM map, we included it only if the genome encoded a corresponding post-transcriptional modification protein. The resolution of the maps enabled the partial sequencing of the bound hibernation factor. The *C. burnetii* genome has two annotated candidate hibernation factors, CBU_0020 and CBU_0745. Modelling of the CBU_0745 AlphaFold v2.2 prediction showed sidechain disagreement against the map. Unlike CBU_0020, CBU_0745 lacks a C-terminal domain and a loop insertion between strands ß2 and ß3 of the N-terminal domain. Similarly, we were able to assign an initial sequence to the large subunit peptide (CLaSP) from the map and identify an open reading frame. Subsequent attempts were made to identify the full length of CLaSP present in the *C. burnetii* ribosome. Chymotryptic digests of SDS-page samples of purified *C. burnetii* ribosomes did not return successful hits from mass spectrometry, likely a combination of the low concentration of ribosome samples that could be purified and the small size of CLaSP. Despite this, the high resolution of the reconstruction enables the identification from the cryo-EM to be of high confidence. The 70S refinement showed density for both 50S triple stack doxycycline and 30S HPF_cold_, so these models were combined for the full 70S model. Final models were refined using Refmac and Phenix^76,77^.

### C. burnetii minimum inhibitory concentration

Doxycycline and tetracycline stocks were both reconstituted in dH_2_O at 10 mg/ml. Eravacycline (CAS 1334714-66-7) and tigecycline (CAS 220620-09-7) were purchased from Cambridge Bioscience. Eravacycline was reconstituted in distilled H_2_O to 1 mg/ml and tigecycline reconstituted in dimethyl sulfoxide (DMSO) to 10 mg/ml (a DMSO serial dilution control was included). All antibiotics were stored at −20 °C. Antibiotics were serially diluted in ACCM-2 and inoculated with final 1 × 10^6 ^*C. burnetii* mCherry-NMII cells. After 10 days or with clear growth in positive controls, MIC values were determined by visual inspection (as described previously^82^). All MIC assays were conducted with a minimum of three repeats.

### HPFcold phylogenetic analysis

Protein sequences containing both PF02482 and PF00313 Pfam domains were retrieved from UniProtKB1^83^. Domains were identified with hmmsearch, part of HMMER v3.3.2 software suite2^84^, against the corresponding Pfam HMMs, using Pfam gathering thresholds3. Only sequences with substantial matches to both target domains were retained. To remove exact duplicates, sequences were clustered with CD-HIT v4.8.14 at 100% identity^85^. Multiple sequence alignment was generated with MAFFT v7.4905 using the G-INS-i algorithm (global-homology strategy)^86^. The alignment was trimmed with trimAl v1.4.rev156 using the gappyout procedure to remove poorly aligned and gap-rich positions while preserving informative sites^87^. Maximum-likelihood trees were reconstructed with IQ-TREE v2.2.07^88^ with the best-fit substitution model selected by ModelFinder8 under the Bayesian Information Criterion (BIC)^89^. Branch support was assessed using ultrafast bootstrap9 (10,000 replicates). Trees were visualised and annotated in iTOL v6^90^.

### Reporting summary

Further information on research design is available in the Nature Portfolio Reporting Summary linked to this article.

## Supplementary information

Supplementary Information

Description of Additional Supplementary Files

Supplementary Movie 1

Reporting Summary

Transparent Peer Review file

## Source data

Source Data

## Data Availability

The structures generated in this study have been submitted to the Protein Data Bank with the codes: 9SLG (*E. coli* 50S doxycycline), 9SX2 (*E. coli* 30S doxycycline), 9T0Z (*C. burnetii* 30S HPF_cold_), 9T61 (*C. burnetii* 30S doxycycline), 9TZ5 (*C. burnetii* 50S doxycycline), 9TZ9 (*C. burnetii* 50S empty), and 9TZE (C. burnetii 70S with 50S doxycycline and 30S HPF_cold_). The cryo-EM data have been submitted to the Electron Microscopy Data Bank (EMDB) with the codes EMD-55001, EMD-55330, EMD-55416, EMD-55601, EMD-56459, EMD-56461, and EMD-56466 (order respective of the PDB accession code ordering). The alignment used to generate Fig. 2f and Supplementary Fig. 6 is provided as Source Data (files 1 and 2). The accession numbers used to generate Supplementary Fig. 5 are provided as Source Data (file 3). The full alignments underpinning Supplementary Fig. 13a are provided as Source Data (files 4 and 5).
