# Supplementary material for: Cryo-EM reveals multiple mechanisms of ribosome inhibition by doxycycline
Source: Nat Commun. 2026 Jun 1;17:7049. doi: 10.1038/s41467-026-73421-5 (PMC13392368; doi:10.1038/s41467-026-73421-5)
Supplement: Supplementary file 1 — Supplementary Information [file 41467_2026_73421_MOESM1_ESM.pdf]

Cryo-EM reveals multiple mechanisms of ribosome inhibition by doxycycline

Stuart et al.

Supplementary information

Contents:

Supplementary Tables

Supplementary Table 1: List of ribosomal proteins modelled for the *C. burnetii ribosome* **3**

Supplementary Table 2: Data collection and refinement parameters **4**

Supplementary Figures

Supplementary Figure 1: Electron density demonstrates a distinct doxycycline binding site in *C. burnetii*. **6**

Supplementary Figure 2: rRNA modifications observed in the *C. burnetii* ribosome **7**

Supplementary Figure 3: HPF_{cold} binds the 30S subunit of the ribosome, protecting the anti-Shine Dalgarno sequence **9**

Supplementary Figure 4: HPF_{cold} is an additional hibernation promoting factor **10**

Supplementary Figure 5: Alignment of HPF_{cold} with paralogues of the two domains **12**

Supplementary Figure 6: Evolutionary conservation of HPF_{cold} **14**

Supplementary Figure 7: Conservation of HPF_{cold} in structural context **16**

Supplementary Figure 8: CLaSP binding in context **18**

Supplementary Figure 9: *C. burnetii* data processing pipeline **20**

Supplementary Figure 10: *E. coli* data processing pipeline **22**

Supplementary Figure 11: Electron density for doxycycline bound in the exit tunnel, comparison to the empty exit tunnel **24**

Supplementary Figure 12: Structural details of doxycycline within the *C. burnetii* exit tunnel **26**

Supplementary Figure 13: rRNA bases contacting doxycycline are highly conserved **28**

Supplementary Figure 14: Tetracycline minimum inhibitory concentrations against *C. burnetii* **30**

Supplementary Figure 15: Inactive *E. coli* ribosome identified through doxycycline driven serendipity **31**

Supplementary Figure 16: Distinct rRNA interactions are made in the doxycycline bound *E. coli* ribosome **33**

Supplementary Figure 17: Cryo-EM map and model validation **35**

Supplementary Figure 18: Cryo-EM particle angular distribution plots **37**

Supplementary Figure 19: Cryo-EM local resolution estimates **39**

Supplementary Figure 20: Cryo-EM map density at doxycycline binding sites **41**

Cryo-EM reveals multiple mechanisms of ribosome inhibition by doxycycline

Stuart et al.

Supplementary discussion 43

Supplementary References 48

Cryo-EM reveals multiple mechanisms of ribosome inhibition by doxycycline

Stuart et al.

Supplementary Table 1: List of ribosomal proteins modelled for the *C. burnetii* ribosome. Ribosomal proteins are given in standard ribosomal protein nomenclature [1]. Gene names are from the *C. burnetii* RSA493 (Nine Mile) genome.

Large Subunit Proteins	Gene	Small Subunit Proteins	Gene
uL2	*CBU_0241*	uS3	*CBU_0244*
uL3	*CBU_0238*	uS4	*CBU_0262*
uL4	*CBU_0239*	uS5	*CBU_0255*
uL5	*CBU_0250*	bS6	*CBU_0864*
uL6	*CBU_0253*	uS7	*CBU_0234*
bL9	*CBU_0867*	uS8	*CBU_0252*
uL13	*CBU_1749*	uS9	*CBU_1748*
uL14	*CBU_0248*	uS10	*CBU_0237*
uL15	*CBU_0257*	uS11	*CBU_0261*
uL16	*CBU_0245*	uS12	*CBU_0233*
bL17	*CBU_0264*	uS13	*CBU_0260*
uL18	*CBU_0254*	uS14	*CBU_0251*
bL19	*CBU_0442*	uS15	*CBU_0851*
bL20	*CBU_1323*	bS16	*CBU_0445*
bL21	*CBU_0385*	uS17	*CBU_0247*
uL22	*CBU_0243*	bS18	*CBU_0865*
uL23	*CBU_0240*	uS19	*CBU_0242*
uL24	*CBU_0249*	bS20	*CBU_0389*
bL25	*CBU_1840*	bS21	*CBU_1593*
bL27	*CBU_0386*	HPF_{cold}	*CBU_0020*
bL28	*CBU_0291*		
uL29	*CBU_0246*		
uL30	*CBU_0256*		
bL32	*CBU_0491*		
bL34	*CBU_1917*		
bL35	*CBU_1324*		
bL36	*CBU_2097*		
CLaSP	-		

Cryo-EM reveals multiple mechanisms of ribosome inhibition by doxycycline

Stuart et al.

Supplementary Table 2: Data collection and refinement parameters

	***E. coli* 70S Ribosome (50S Bound Doxycycline)**	***E. coli* 30S Ribosome Subunit (30S Bound Doxycycline**	***C. burnetii* 30S Ribosome Subunit (HPFcold Bound)**	***C. burnetii* 30S Ribosome Subunit (30S Bound Doxycycline)**	***C. burnetii* 70S Ribosome (50S Doxycycline & 30S HPFcold)**	***C. burnetii* 50S Ribosome Subunit (50S Bound Doxycycline)**	***C. burnetii* 50S Ribosome Subunit (Empty)**
	(EMDB-55001)	(EMDB-55330)	(EMDB-55416)	(EMDB-55601)	(EMDB-56466)	(EMDB-56459)	(EMDB-56461)
	(PDB 9SLG)	(PDB 9SX2)	(PDB 9T0Z)	(PDB 9T61)	(PDB 9TZE)	(PDB 9TZ5)	(PDB 9TZ9)
**Data collection and processing**							
Magnification	130,000	130,000	81,000	81,000	81,000	81,000	81,000
Voltage (kV)	300	300	300	300	300	300	300
Electron exposure (e–/Å^2)	45	45	45	45	45	45	45
Defocus range (μm)	-0.4 to -2.5 (0.3 steps)	-0.4 to -2.5 (0.3 steps)	-0.8 to -2.0 (0.3 steps)	-0.8 to -2.0 (0.3 steps)	-0.8 to -2.0 (0.3 steps)	-0.8 to -2.0 (0.3 steps)	-0.8 to -2.0 (0.3 steps)
Pixel size (Å)	0.645	0.645	1.06	1.06	1.06	1.06	1.06
Symmetry imposed	C1	C1	C1	C1	C1	C1	C1
Initial particle images (no.)	2,257,467	2,257,467	5,866,605	5,866,605	5,866,605	5,866,605	5,866,605
Final particle images (no.)	215,222	372,420	24,143	10,803	44,196	91,218	129,328
Map resolution (Å)	2.16	2.08	2.87	3.06	2.48	2.22	2.19
FSC threshold	0.143	0.143	0.143	0.143	0.143	0.143	0.143
**Refinement**							
Initial model used (PDB code)	7K00	7K00	N/A	N/A	N/A	N/A	N/A
Map model FSC (masked, Å)	2.63	2.45	3.04	3.37	2.88	2.63	2.53
FSC threshold	0.5	0.5	0.5	0.5	0.5	0.5	0.5
Map sharpening *B* factor (Å^2)	N/A	N/A	N/A	N/A	N/A	N/A	N/A
Model composition							
Non-hydrogen atoms	150,218	54,044	52,223	51,204	142,356	90,136	89,972
Residues	Protein: 5946<br>Nucleotide: 4561	Protein: 2492<br>Nucleotide: 1534	Protein: 2414<br>Nucleotide: 1535	Protein: 2239<br>Nucleotide: 1552	Protein: 5670<br>Nucleotide: 4525	Protein: 3256<br>Nucleotide: 2990	Protein: 3256<br>Nucleotide: 2990
Ligands	K: 77<br>ZN: 2<br>MG: 447<br>MS6: 1<br>DXT: 1<br>IAS: 1	K: 4<br>ZN: 1<br>MG: 142<br>IAS: 1<br>DXT: 1	K: 34<br>ZN: 2<br>MG: 53	K: 34<br>ZN: 2<br>MG: 54<br>DXT: 1	K: 167<br>ZN: 3<br>MG: 220<br>DXT: 3	K: 134<br>ZN: 1<br>MG: 167<br>DXT: 3	K: 136<br>ZN: 1<br>MG: 162
B factors (min,max, mean; Å^2)							
Protein	30.00/157.46/62.71	30.00/157.46/60.46	45.15/89.11/64.45	52.46/107.76/74.56	28.09/80.07/59.53	28.09/66.45/45.10	28.09/67.55/48.66
Nucleotide	44.82/94.85/59.74	34.29/68.86/53.18	43.69/84.26/59.91	50.28/102.49/69.38	38.78/76.47/51.41	38.78/54.05/44.64	35.18/66.51/46.77
Ligand	30.00/157.46/63.52	30.00/115.41/57.08	7.46/167.39/64.12	26.67/200.00/78.23	11.21/222.37/58.78	11.21/122.88/49.06	12.57/148.70/51.02

Water	30.00/109.28/63.65	30.00/109.28/52.61	0.5/81.28/35.96	0.65/60.94/39.21	20.96/127.95/59.64	20.96/53.99/33.53	24.99/52.48/34.97
R.m.s. deviations							
Bond lengths (Å) (# > 4 σ)	0.008 (108)	0.007 (23)	0.014 (32)	0.007 (39)	0.006 (54)	0.006 (23)	0.006 (21)
Bond angles (°) (# > 4 σ)	1.181 (468)	1.120 (38)	1.875 (559)	1.220 (122)	1.225 (386)	1.223 (262)	1.180 (176)
Validation							
MolProbity score	1.53	1.17	1.1	1.06	1.07	1.04	1.03
Clashscore	8.25	2.11	1.97	1.97	1.75	1.57	1.56
Poor rotamers (%)	0.90	0.73	1.33	1.33	1.27	1.22	1.18
Ramachandran plot							
Favored (%)	97.60	96.85	97.80	98.04	97.74	97.69	97.65
Allowed (%)	2.40	3.15	2.20	1.96	2.23	2.31	2.35
Disallowed (%)	0.00	0.00	0.00	0.00	0.04	0.00	0.00

Cryo-EM reveals multiple mechanisms of ribosome inhibition by doxycycline

Stuart et al.

Supplementary Figure 1: Electron density demonstrates a distinct doxycycline binding site in *C. burnetii*.

(**a,b**) Following classification, clear density could be observed for doxycycline in the A-site of the ribosomal 30S subunit of *C. burnetii* and *E. coli* respectively (rRNA and doxycycline shown as sticks. Colours: oxygen, red; nitrogen, blue; phosphorus, orange; rRNA carbon, cyan; doxycycline carbon, green; electron density, grey). The coordinating rRNA base C1054 is highlighted in both structures. O11 and O12 of doxycycline coordinate a magnesium bound by the phosphate groups of C1054 and G1198 while ring D stacks upon the base of C1054. Another magnesium bound to the phosphate of A966 coordinates O3. (**c**) A second doxycycline binding site is observed in the *C. burnetii* 50S ribosome. Three doxycycline molecules form a stack with three coordinated magnesium atoms. Electron density shown as a blue net. (**d**) The respective *E. coli* region following classification shows little evidence of an equivalent state, with limited evidence for only the lower molecule in the image shown. Colours: doxycycline carbon, white; electron density, grey net.

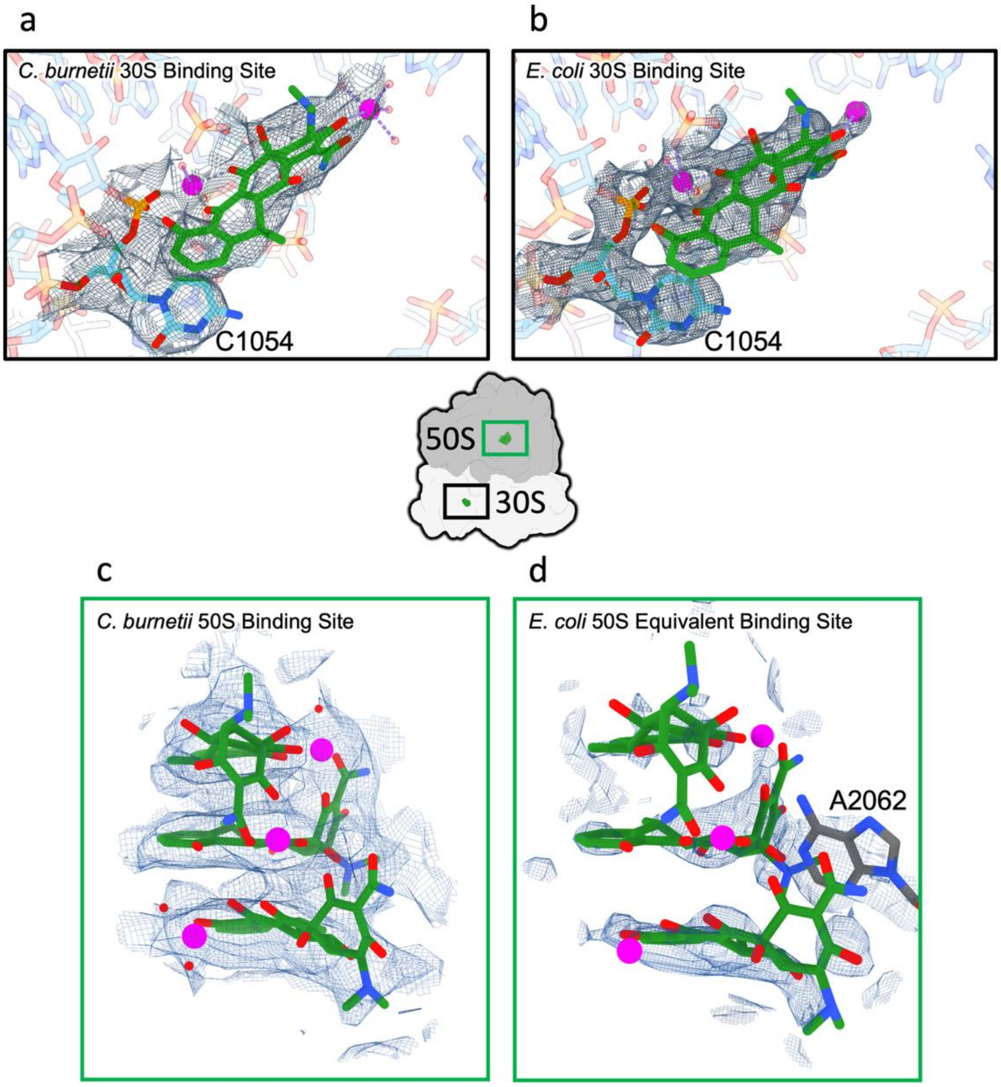

Cryo-EM reveals multiple mechanisms of ribosome inhibition by doxycycline

Stuart et al.

Supplementary Figure 2: rRNA modifications observed in the *C. burnetii* ribosome.

C. burnetii shows fewer rRNA post-transcriptional modifications than *E. coli*. Modifications were included in the structure where they were clearly indicated by the density. Modifications were only included if an orthologue of an *E. coli* modification gene is present in *C. burnetii* and supported by map density (e.g. pseudo uridylation have been excluded, even if a corresponding *C. burnetii* gene is present). Modifications are numbered in the *C. burnetii* ribosome, with *E. coli* numbering in parentheses. Modification abbreviations: OMU: 2'-*O*-methyluridine; 2MA: 2-methyladenosine; 7MG: 7-methylguanosine; 5MC, 5-methylcytosine; 4,2MC: 4-*N*-methyl, 2'-*O*-methylcytosine; 3MU, 3-*N*-methyluridine; 2MG, 2-*N*-methylguanosine; 6M (2)A, 6-*N*,*N*-dimethyladenine. Bases shown as sticks. Colours: yellow, carbon; red, oxygen; blue, nitrogen; light blue, *E. coli* density; grey, *C. burnetii* density.

C. burnetii Modification (*E. coli*)	*E. coli*	*C. burnetii*	*C. burnetii* Gene	
OMU-2568 (2552)	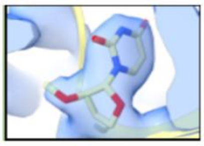	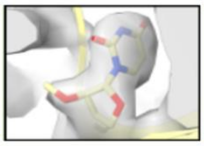	*rlmE, CBU_1353*	**23S**
2MA-2519 (2503)	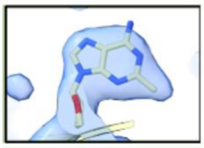	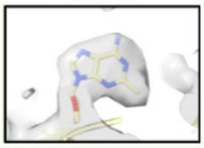	*rlmN, CBU_1252*	
OMG-2267 (2251)	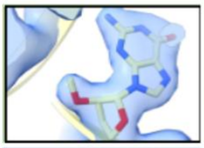	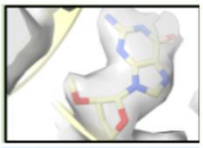	*rlmB, CBU_0986*	
7MG-524 (527)	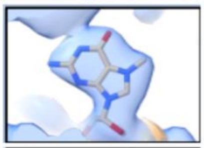	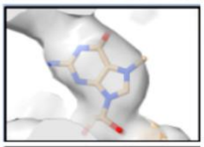	*rsmG, CBU_1925*	**16S**
2MG-962 (966)	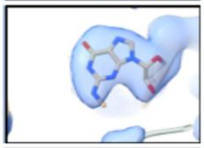	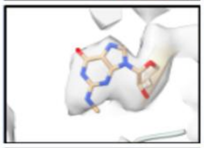	*rsmD, CBU_1899*	
5MC-963 (967)	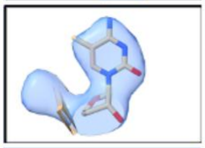	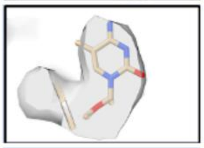	*rsmB, CBU_1915*	
4,2MC-1399 (1402)	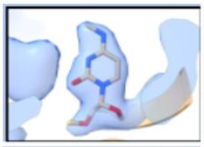	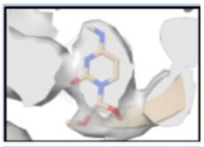	*rsmH & rsmI, CBU_0116 & CBU_1739*	
3MU-1495 (1498)	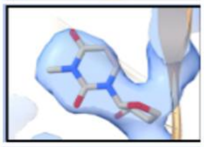	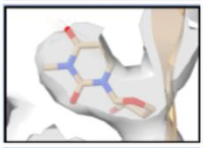	*rsmE, CBU_1960*	
2MG-1513 (1516)	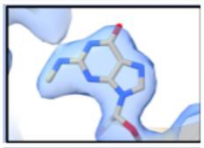	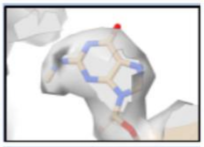	*rsmJ, CBU_1876*	
6M(2)A-1515 (1518)	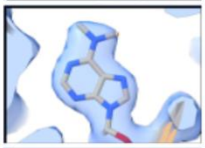	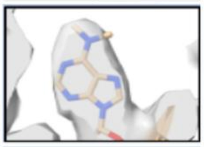	*rsmA, CBU_1982*	
6M(2)A-1516 (1519)	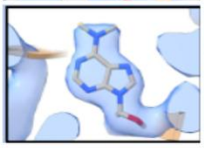	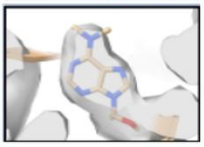	*rsmA, CBU_1982*	

Cryo-EM reveals multiple mechanisms of ribosome inhibition by doxycycline

Stuart et al.

Supplementary Figure 3: HPF$_{cold}$ binds the 30S subunit of the ribosome, protecting the anti-Shine Dalgarno sequence.

Structural characterisation of HPF$_{cold}$ (hibernation promoting factor) bound to the *C. burnetii* ribosome. (**a**) The cold shock domain (CSD) interacts intimately with the 3' region of the 16S rRNA containing the anti-Shine Dalgarno (aSD) sequence at the mRNA exit channel. rRNA shown as ribbon and sticks with the aSD sequence in rainbow colours; CSD shown as cartoon and semi-transparent surface in blue. (**b**) The aSD wraps around the CSD, with U1534 stacking upon Y142 while C1535 stacks with F132 and forms a hydrogen bond pseudo-pair to R123 with its N3 and O2 atoms. rRNA and interacting residues of CSD shown as sticks. Colours: oxygen, red; nitrogen/CSD carbon, blue; phosphate/rRNA carbon, orange. (**c**) R177 acts as a bridge between two rRNA segments, forming salt bridges with a cleft of the rRNA phosphate backbone formed by G924-925 and C1530. rRNA shown as ribbon and sticks (carbons and ribbon in cyan). aSD not differentiated with colour but C1535 indicated with open black arrowhead. (**d**) HPF$_{cold}$ and bS21 pincer the aSD. Further interactions are made on the other face of the CSD with rRNA (yellow) and bS7. Ribosomal proteins shown as pink cartoon.

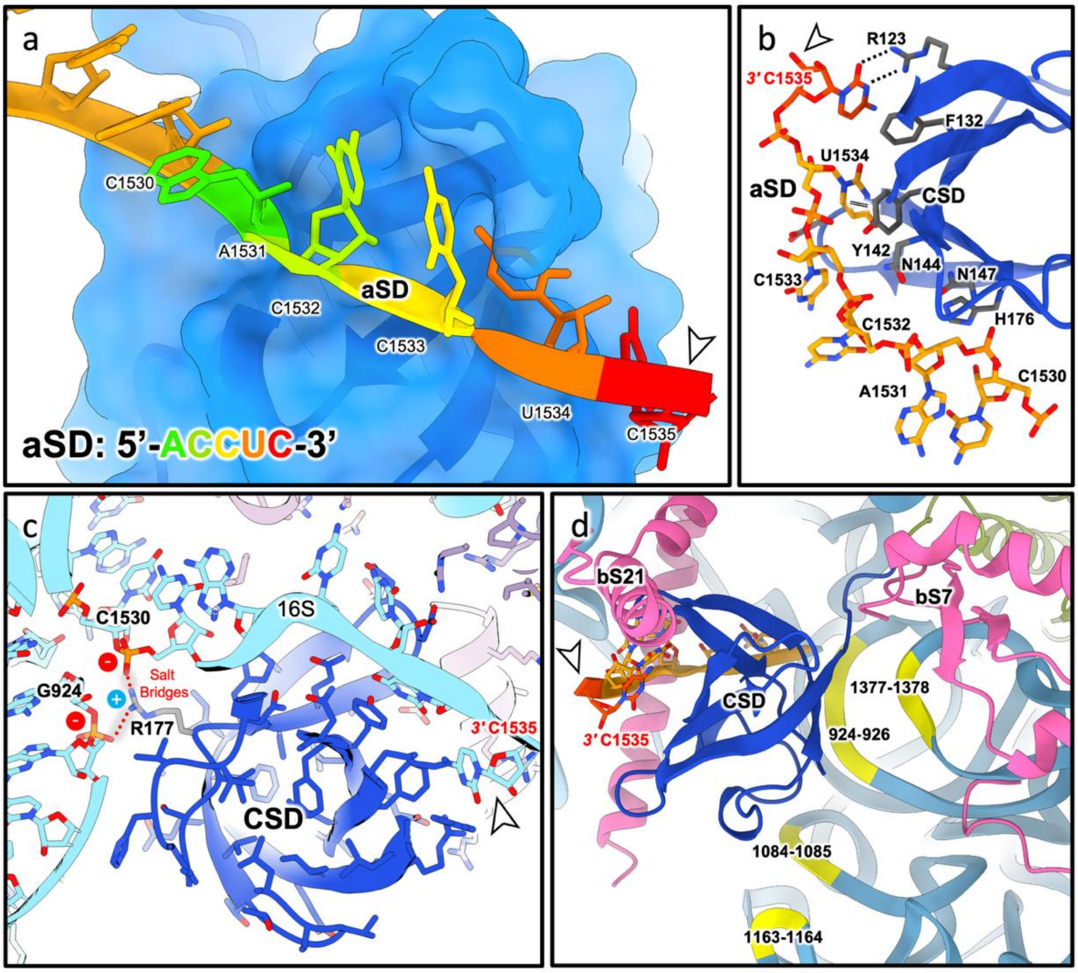

Cryo-EM reveals multiple mechanisms of ribosome inhibition by doxycycline

Stuart et al.

Supplementary Figure 4: HPF_{cold} is an additional hibernation promoting factor

While no structural data for sequence homologues could be identified in the protein data bank, the CSD (cold shock domain) displays a classical cold shock protein fold. (**a**) Sequence alignment (Supplementary Fig. 5a) against the *E. coli* (K12) cold shock proteins reveals generally low sequence identity, indicating evolutionary divergence. Accession numbers are provided as a Source Data file. (**b**) AlphaFold 3 [2] predicts that *Legionella pneumophila* HPF_{cold} is structurally highly comparable to $CbHPF_{cold}$. Proteins shown as cartoon; green, *C. burnetii*, blue, *L. pneumophila*. (**c**) A KLEVG motif (magenta) is conserved between *C. burnetii* and *L. pneumophila* HPF_{cold} CSDs (blue) and not present in standalone cold shock proteins. This motif falls on the CSD interface contacting helix 40 of the 16S rRNA, opposite to the aSD interface (orange). The KLEVG Lys155 inserts into the backbone cleft between A1164 and G1084. Proteins and rRNA shown as cartoon with semi-transparent surface, with sticks for the KLEVG motif. (**d**) Structural alignment between HPF_{cold} CSD and CspA from *E. coli* (PDB: 1mjc [3]). RMSD (root mean square deviation) values (calculated in ChimeraX 1.9 [4]) sequence alignment score = 107.2, RMSD between 56 pruned residues is 0.799 Å; and 1.756 Å across all 66 aligned residues. (**e**) Structural comparison of known ribosomal hibernation factors (alignment: Supplementary Fig. 5b). Structures are superposed on the common domain and shown as cartoon. Colours: HPF_{short}, orange (PDB: 4v8i [5]); YfiA, blue (PDB: 4v8h [5]); HPF_{long}, magenta (PDB: 5myj [6]); HPF_{cold}, green and blue. Both the N-terminal (NTD) and C-terminal (CTD) domains of HPF_{long} are indicated. Both HPF_{cold} and HPF_{long} C-terminal domains likely function to preserve the ribosome during hibernation, albeit in 70S and 100S ribosomal forms respectively.

a

E.coli (K12 Csp)	Max Score	Total Score	Query Cover	E value	Per. Ident	Acc. Len
CspA	25.0	25.0	88%	1e-05	31%	70
CspB	20.0	20.0	85%	0.001	28%	71
CspC	24.6	24.6	94%	2e-05	29%	69
CspD	22.7	22.7	88%	1e-04	26%	74
CspE	23.1	23.1	94%	6e-05	29%	69
CspF	-	-	-	-	-	-
CspG	19.6	19.6	85%	0.001	30%	70
CspH	16.9	16.9	85%	0.013	32%	70
CspI	20.8	20.8	34%	5e-04	46%	70

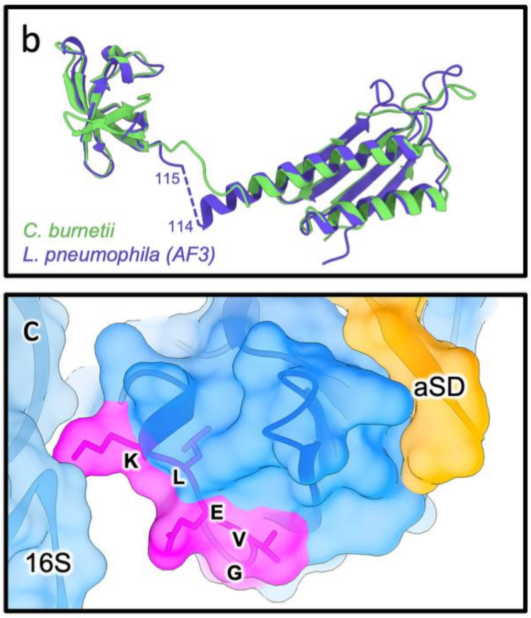

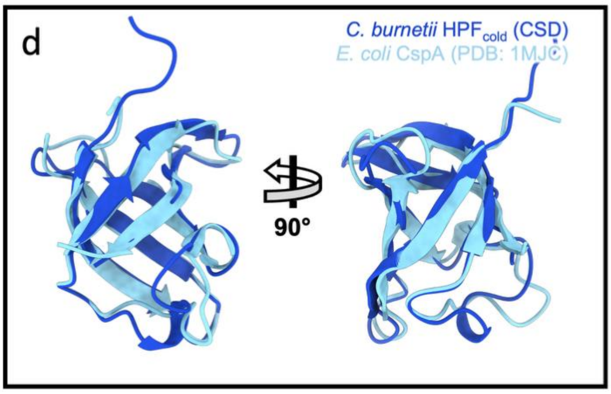

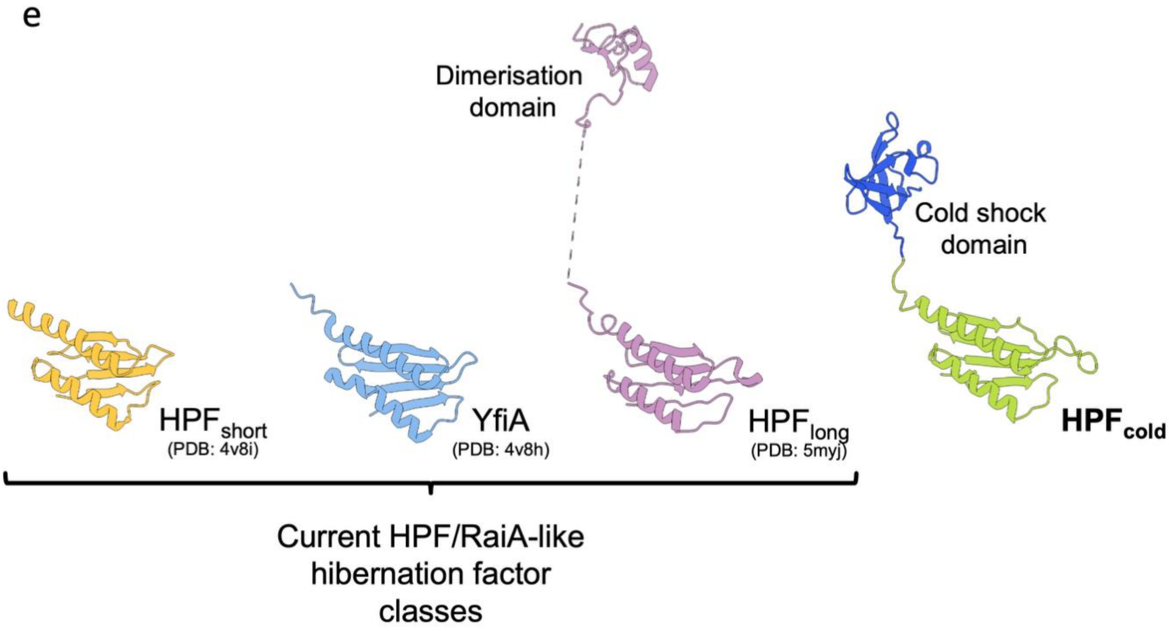

Cryo-EM reveals multiple mechanisms of ribosome inhibition by doxycycline

Stuart et al.

Supplementary Figure 5: Alignment of HPF_{cold} with paralogues of the two domains

(**a**) The cold shock domain (CSD, Glu115 onwards) of HPF_{cold} (hibernation promoting factor) from *Coxiella burnetii* and *Legionella pneumophila* was aligned to the nine cold shock proteins of *E. coli* K12. (**b**) The hibernation factor domain of HPF_{cold} from *C. burnetii* and *L. pneumophila* was aligned to example sequences of the three established hibernation factors (HPF_{long} from *Staphylococcus aureus*, YfiA from *E. coli* K12, and HPF_{short} from *E. coli* K12. Alignments were performed using Clustal Omega [7] and visualised using Espript 3 [8]. Accession numbers are provided as a Source Data file.

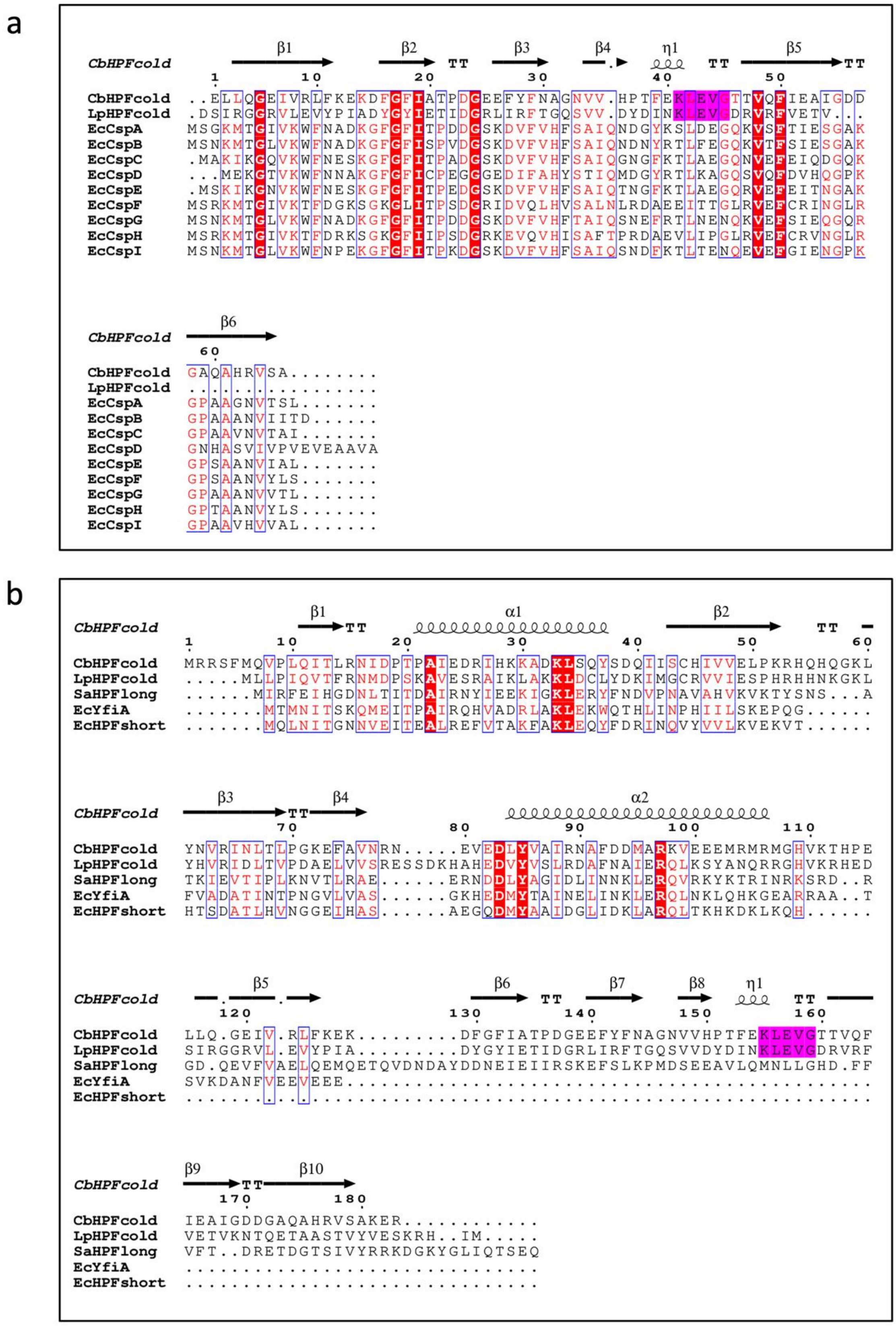
a
CbHPFcold
β1 β2 β3 β4 η1 β5
CbHPFcold ..ELLQGEIVRLFKEKDFGFIATPDGEEFYFNAGNVV.HPTFEKLEVGTTVQFIEAIGDD
LpHPFcold .DSIRGGRVLEVYPIADYGYIETIDGRLIRFTGQSVV.DYDINKLEVGDRVRFVETV...
EcCspA MSGKMTGIVKWFNADKGFGFITPDDGSKDVFVHFSAIQNDGYKSLDEGQKVSFTIESGAK
EcCspB MSNKMTGLVKWFNADKGFGFISPVDGSKDVFVHFSAIQNDNYRTLFEGQKVTFSIESGAK
EcCspC .MAKIKGQVKWFNESKGFGFITPADGSKDVFVHFSAIQGNGFKTLAEGQNVEFEIQDGQK
EcCspD ...MEKGTVKWFNNAKGFGFICPEGGGEDIFAHYSTIQMDGYRTLKAGQSVQFDVHQGPK
EcCspE .MSKIKGNVKWFNESKGFGFITPEDGSKDVFVHFSAIQTNGFKTLAEGQRVEFEITNGAK
EcCspF MSRKMTGIVKTFDGKSGKGLITPSDGRIDVQLHVSALNLRDAEEITTGLRVEFCRINGLR
EcCspG MSNKMTGLVKWFNADKGFGFITPDDGSKDVFVHFTAIQSNEFRTLNENQKVEFSIEQGQR
EcCspH MSRKMTGIVKTFDRKSGKGFIIPSDGRKEVQVHISAFTPRDAEVLIPGLRVEFCRVNGLR
EcCspI MSNKMTGLVKWFNPEKGFGFITPKDGSKDVFVHFSAIQSNDFKTLTENQEVEFGIENGPK
CbHPFcold
β6
CbHPFcold GAQAHRVSA........
LpHPFcold ....
EcCspA GPAAGNVTSL.......
EcCspB GPAAANVIITD......
EcCspC GPAAVNVTAI.......
EcCspD GNHASVIVPVEVEAAVA
EcCspE GPSAANVIAL.......
EcCspF GPSAANVYLS.......
EcCspG GPAAANVVTL.......
EcCspH GPTAANVYLS.......
EcCspI GPAAVHVVAL.......
b
CbHPFcold
β1 α1 β2
CbHPFcold MRRSFMQVPLQITLRNIDPTPAIEDRIHKKADKLSQYSDQIISCHIVVELPKRHQHQGKL
LpHPFcold .....MLLPIQVTFRNMDPSKAVESRAIKLAKKLDCLYDKIMGCRVVIESPHRHHNKGKL
SaHPFlong ......MIRFEIHGDNLTITDAIRNYIEEKIGKLERYFNDVPNAVAHVKVKTYSNS...A
EcYfiA .......MTMNITSKQMEITPAIRQHVADRLAKLEKWQTHLINPHIILSKEPQG......
EcHPFshort .......MQLNITGNNVEITEALREFVTAKFAKLEQYFDRINQVYVVLKVEKVT......
CbHPFcold
β3 β4 α2
CbHPFcold YNVRINLTLPGKEFAVNRN.....EVEDLYVAIRNAFDDMARKVEEEMRMRMGHVKTHPE
LpHPFcold YHVRIDLTVPDAELVVSRESSDKHAHEDVYVSLRDAFNAIERQLKSYANQRRGHVKRHED
SaHPFlong TKIEVTIPLKNVTLRAE......ERNDDLYAGIDLINNKLERQVRKYKTRINRKSRD..R
EcYfiA FVADATINTPNGVLVAS......GKHEDMYTAINELINKLERQLNKLQHKGEARRAA..T
EcHPFshort HTSDATLHVNGGEIHAS......AEGQDMYAAIDGLIDKLARQLTKHKDKLKQH......
CbHPFcold
β5 β6 β7 β8 η1
CbHPFcold LLQ.GEIV.RLFKEK.........DFGFIATPDGEEFYFNAGNVVHPTFEKLEVGTTVQF
LpHPFcold SIRGGRVL.EVYPIA.........DYGYIETIDGRLIRFTGQSVVDYDINKLEVGDRVRF
SaHPFlong GD.QEVFVAELQEMQETQVDNDAYDDNEIEIIRSKEFSLKPMDSEEAVLQMNLLGHD.FF
EcYfiA SVKDANFVEEVEEE..
EcHPFshort ....
CbHPFcold
β9 β10
CbHPFcold IEAIGDDGAQAHRVSAKER............
LpHPFcold VETVKNTQETAASTVYVESKRH..IM.....
SaHPFlong VFT..DRETDGTSIVYRRKDGKYGLIQTSEQ
EcYfiA ....
EcHPFshort ....

Cryo-EM reveals multiple mechanisms of ribosome inhibition by doxycycline

Stuart et al.

Supplementary Figure 6: Evolutionary conservation of HPF_{cold}

100 representative sequences from across the HPF_{cold} (hibernation promoting factor) alignment were analysed using ConSurf 2010 [9] with the results mapped to the *C. burnetii* reference sequence (Uniprot: Q83FC0). (**a**) Summary of the similarity values obtained from ConSurf across the protein. The HPF domain is highlighted in yellow, and the CSD (cold shock domain) domain in green. The conservation is notably higher in the CSD. (**b**) Residue-by-residue identification of the conserved amino acids in HPF_{cold}. The similarity scores were converted to a heatmap and plotted onto the HPF_{cold} structure (PDB: 9T0Z). This alignment is provided as a Source Data file.

a

b

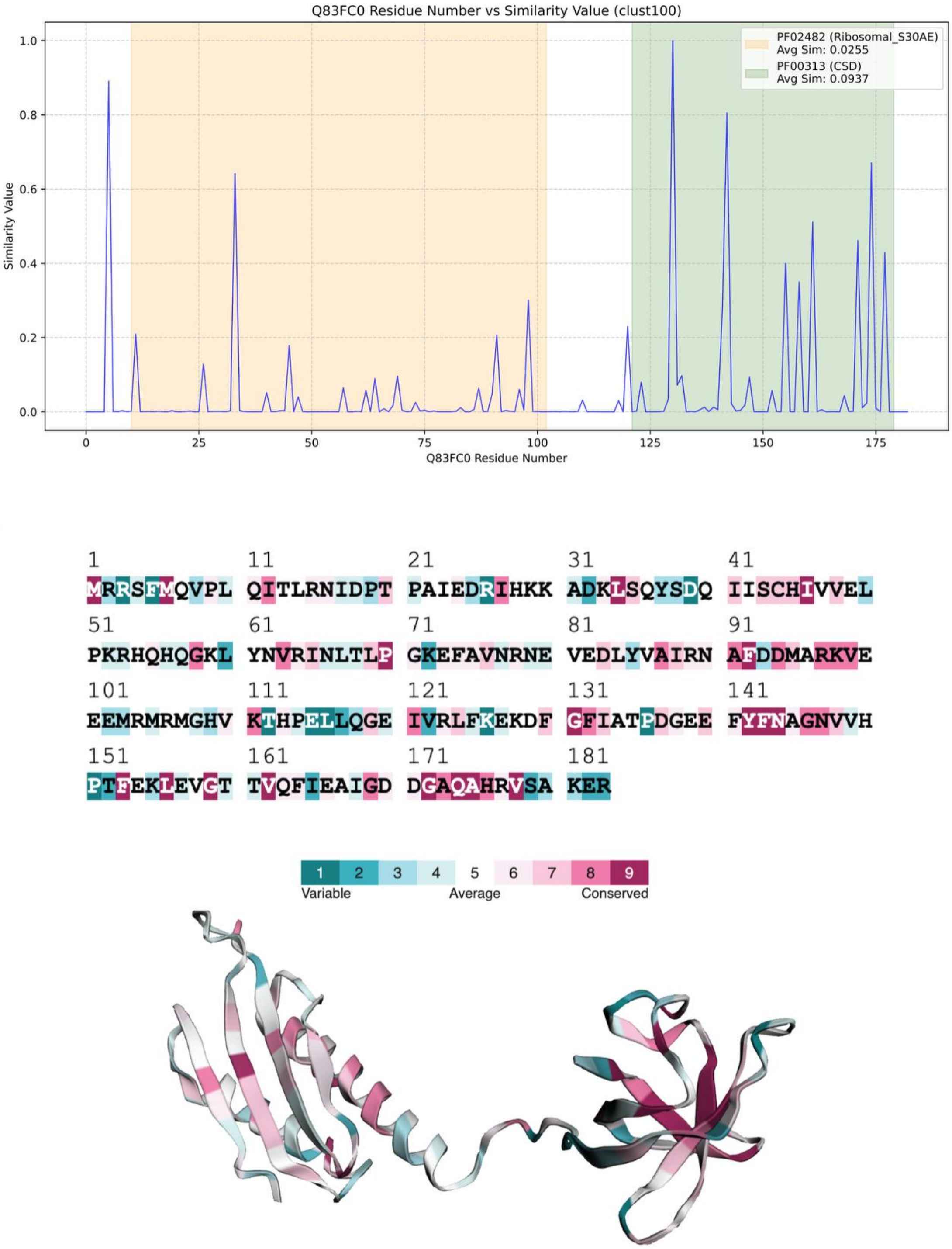
Q83FC0 Residue Number vs Similarity Value (clust100)
PF02482 (Ribosomal_S30AE)
Avg Sim: 0.0255
PF00313 (CSD)
Avg Sim: 0.0937
Similarity Value
Q83FC0 Residue Number
1 MRRSFMQVPL 11 QITLRNIDPT 21 PAIEDRIHKK 31 ADKLSQYSDQ 41 IISCHIVVEL
51 PKRHQHQGKL 61 YNVRINLTLP 71 GKEFAVNRNE 81 VEDLYVAIRN 91 AFDDMARKVE
101 EEMRMRMGHV 111 KTHPELLQGE 121 IVRLFKEKDF 131 GFIATPDGEE 141 FYFNAGNVVH
151 PTFEKLEVGT 161 TVQFIEAIGD 171 DGAQAHRVSA 181 KER
1 2 3 4 5 6 7 8 9
Variable
Average
Conserved

Cryo-EM reveals multiple mechanisms of ribosome inhibition by doxycycline

Stuart et al.

Supplementary Figure 7: Conservation of HPF_{cold} in structural context

The conservation heatmap colouring from Supplementary Fig. 6 were mapped to the HPF_{cold} (hibernation promoting factor) surface using Pymol v. 3.1.6.1 (Schrödinger) and the structure of the 30S ribosome with HPF_{cold} (PDB: 9T0Z). RNA and other ribosomal proteins are shown as orange and green cartoon respectively, potassium ions are shown as purple spheres, and magnesium ions are shown as green spheres. (**a,b,c**) Cold shock domain (CSD). The most significant patch of surface conservation occurs where HPF_{cold} binds to the anti-Shine-Dalgarno rRNA sequence (**b**, yellow cartoon). This includes the only strongly conserved residues in HPF_{cold} with side chains facing the exterior of the protein (as opposed to conserved structural residues). In contrast, the other faces of HPF_{cold} (**a**, **c**) show limited surface conservation. (**d**,**e**) HPF domain. The HPF domain similarly shows limited surface conservation. The only significant patch is clustered around a conserved magnesium binding site (**d**, open arrowhead). Other moderately conserved surface residues are limited to individual positively charged residues binding to the phosphate backbone. The striking conservation of the anti-Shine-Dalgarno sequence binding region suggests that this is an important function of HPF_{cold} in *C. burnetii.*

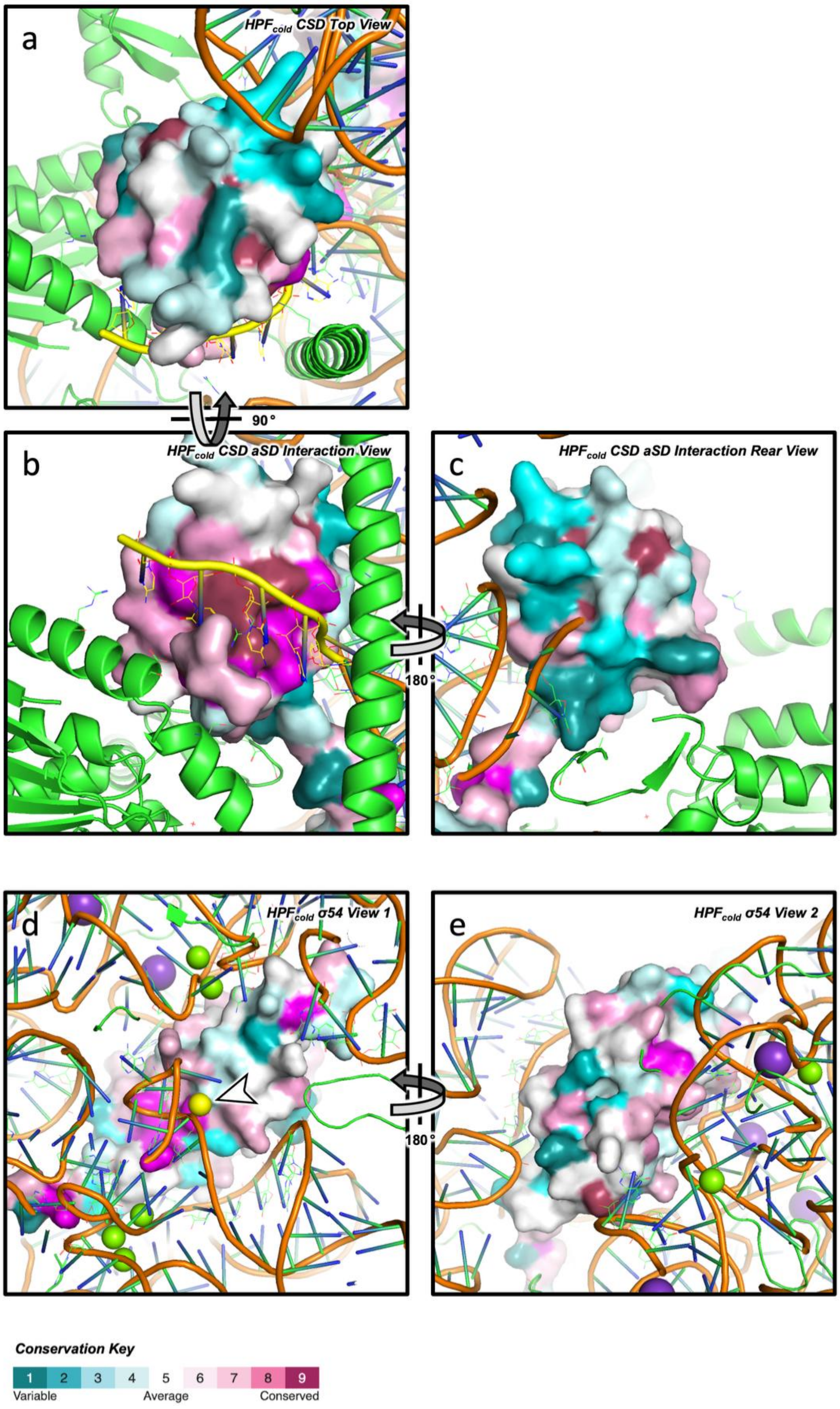
a
HPFcold CSD Top View
90°
b
HPFcold CSD aSD Interaction View
c
HPFcold CSD aSD Interaction Rear View
180°
d
HPFcold σ54 View 1
e
HPFcold σ54 View 2
180°
Conservation Key
1 2 3 4 5 6 7 8 9
Variable
Average
Conserved

Cryo-EM reveals multiple mechanisms of ribosome inhibition by doxycycline

Stuart et al.

Supplementary Figure 8: CLaSP binding in context

(**a**) *Coxiella burnetii* displays a smaller loop D 5S rRNA conformation in comparison to *Escherichia coli*, more similar to the 5S found in *Mycobacterium tuberculosis*. Like *C. burnetii*, *M. tuberculosis* also occupies this cleft with a ribosomal protein, named bL37 (PDB: 5xym [10]). (**b**) bL37 displays a more compact, helical structure than CLaSP (*Coxiellaceae* large subunit peptide). 5S rRNA shown as purple ribbon and sticks with semi-transparent surface. 23S rRNA shown as light green ribbon. CLaSP (pink) and bL37 (green) shown as cartoon and sticks with semi-transparent surface. Other ribosomal proteins shown as grey cartoon and semi-transparent surface. The C-terminal residue (A24) of bL37 is at the rear of the protein as shown (black arrow). (**c**) CLaSP is located adjacent to *nusB*, one base out of frame from the 3' end. Orange – *nusB*; pink – *CLaSP*.

Cryo-EM reveals multiple mechanisms of ribosome inhibition by doxycycline

Stuart et al.

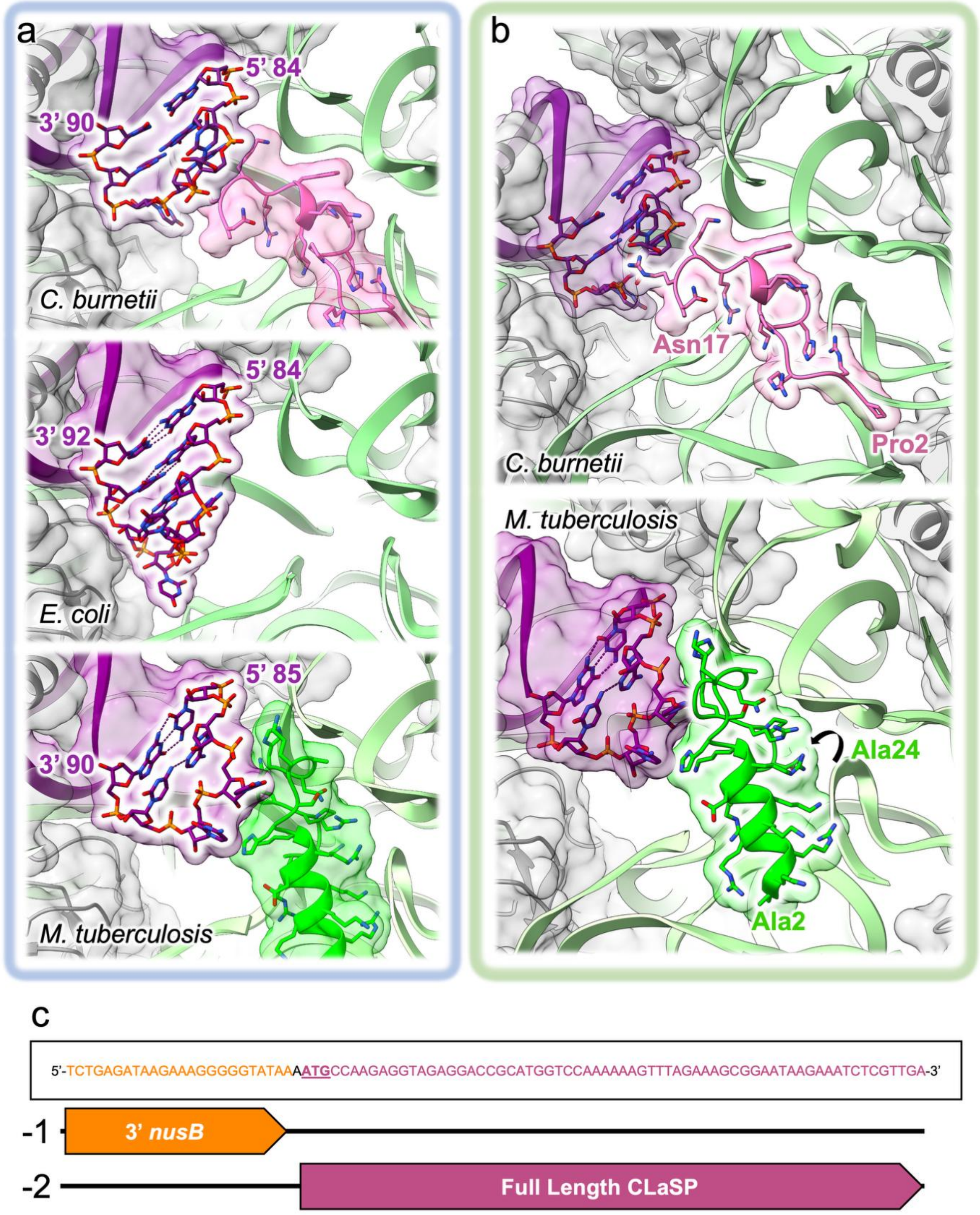

Cryo-EM reveals multiple mechanisms of ribosome inhibition by doxycycline

Stuart et al.

Supplementary Figure 9: *C. burnetii* data processing pipeline

Overview of the data processing strategy. Images comparing electron density following classification are at the same sigma values. Left: a mask was applied at the site of the doxycycline triple stack (purple area shown). This separated the particles into five classes, with two classes (41.5% of particles) representing the doxycycline bound 50S ribosome and 58.5% representing an empty 50S ribosome. Right: a mask was applied at the site of HPF_{cold} (hibernation promoting factor; cyan area shown). Two classes were separated, with 55% bound to HPF_{cold} (purple) and 45% showing doxycycline (green) and a tRNA (blue).

Cryo-EM reveals multiple mechanisms of ribosome inhibition by doxycycline

Stuart et al.

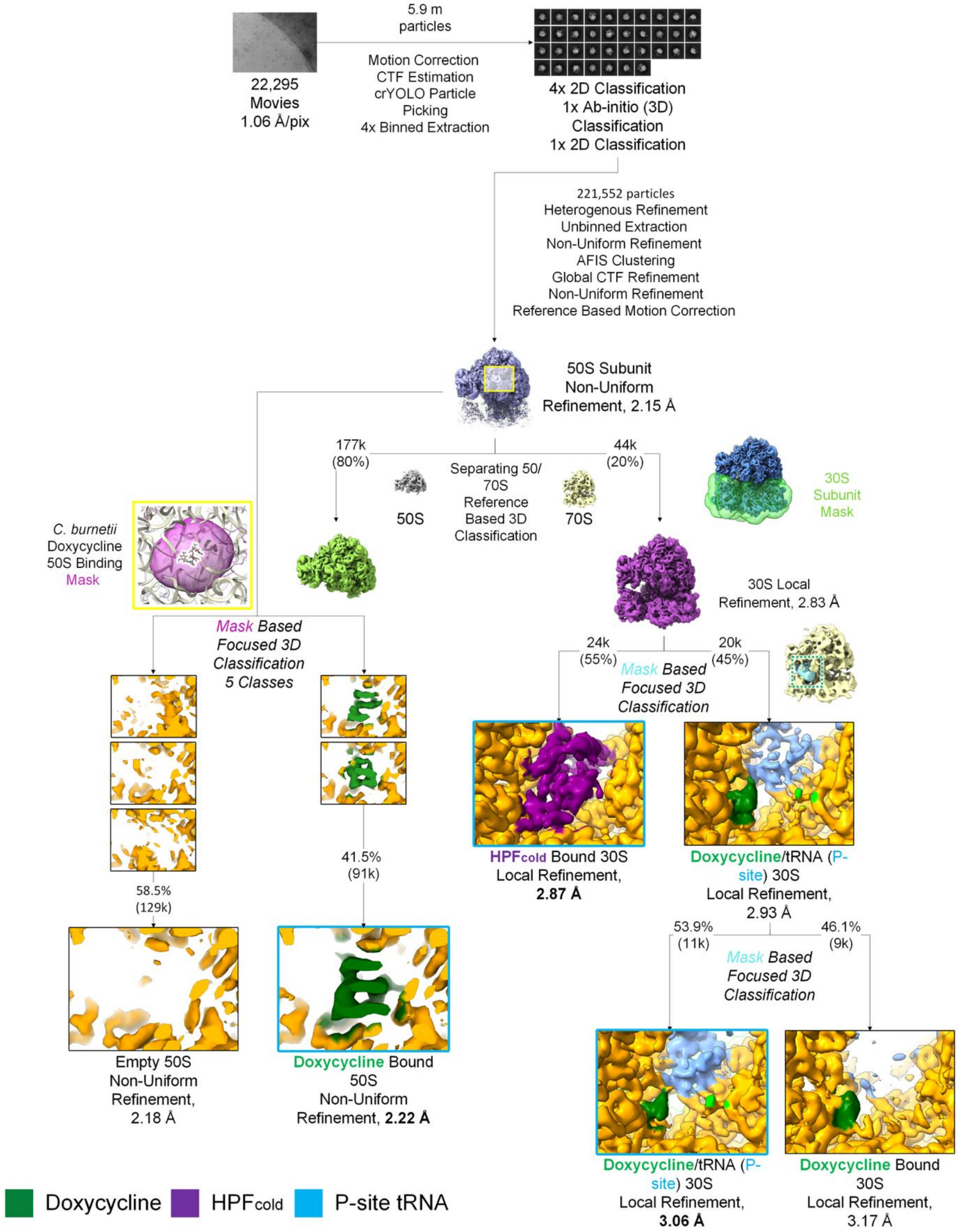

Cryo-EM reveals multiple mechanisms of ribosome inhibition by doxycycline

Stuart et al.

Supplementary Figure 10: *E. coli* data processing pipeline

Overview of the data processing strategy. Images comparing electron density following classification are at the same sigma values. Left: a mask was applied at the site of the doxycycline binding to the 50S ribosome (purple area shown). This separated the particles into ten classes, with one class (12.3% of particles) representing the doxycycline bound 50S ribosome. Right: a mask was applied at the site of YfiA (hibernation factor) (cyan area shown). Two classes were separated, with 61% bound to YfiA (purple) and 39% showing doxycycline (green) and a tRNA (blue).

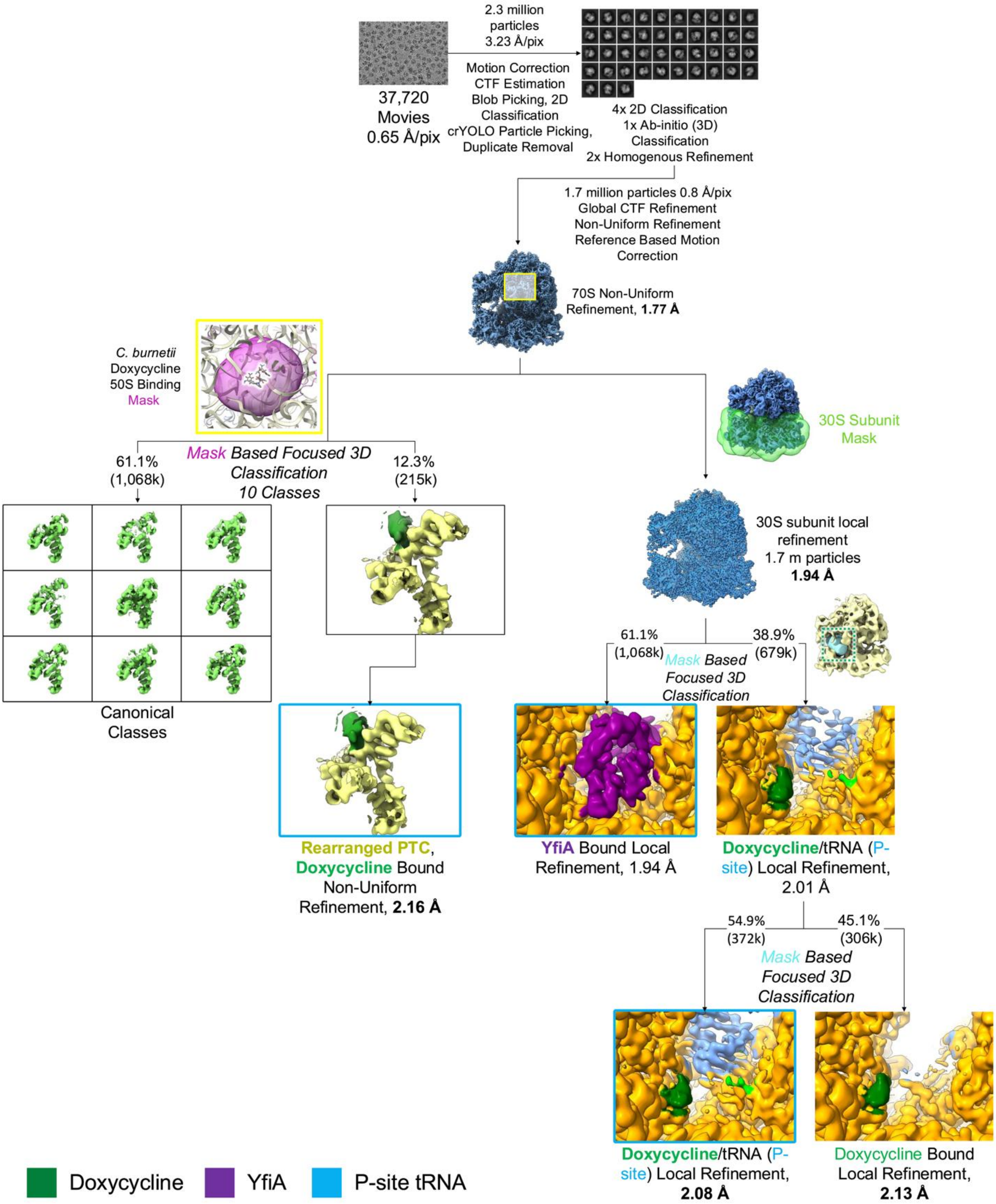
2.3 million particles 3.23 Å/pix
37,720 Movies 0.65 Å/pix
Motion Correction
CTF Estimation
Blob Picking, 2D Classification
crYOLO Particle Picking, Duplicate Removal
4x 2D Classification
1x Ab-initio (3D) Classification
2x Homogenous Refinement
1.7 million particles 0.8 Å/pix
Global CTF Refinement
Non-Uniform Refinement
Reference Based Motion Correction
70S Non-Uniform Refinement, 1.77 Å
C. burnetii Doxycycline 50S Binding Mask
30S Subunit Mask
Mask Based Focused 3D Classification 10 Classes
61.1% (1,068k)
12.3% (215k)
Canonical Classes
Rearranged PTC, Doxycycline Bound Non-Uniform Refinement, 2.16 Å
30S subunit local refinement 1.7 m particles 1.94 Å
61.1% (1,068k)
38.9% (679k)
Mask Based Focused 3D Classification
YfiA Bound Local Refinement, 1.94 Å
Doxycycline/tRNA (P-site) Local Refinement, 2.01 Å
54.9% (372k)
45.1% (306k)
Mask Based Focused 3D Classification
Doxycycline/tRNA (P-site) Local Refinement, 2.08 Å
Doxycycline Bound Local Refinement, 2.13 Å
Doxycycline
YfiA
P-site tRNA

Cryo-EM reveals multiple mechanisms of ribosome inhibition by doxycycline

Stuart et al.

Supplementary Figure 11: Electron density for doxycycline bound in the exit tunnel, comparison to the empty exit tunnel

(**a, b**) Focused classification of the *Coxiella burnetii* 50S ribosome (Supplementary Fig. 9) separated the particles into those containing a three-molecule doxycycline stack (**a**) and doxycycline-free 50S ribosomes (**b**) reconstructions. (**c**) An interaction is between DOX2 and U2526, stabilising the conformation of the base in a luminal, translationally stalled-like position. (**d**) Equivalent density for U2526 in the unbound ribosome is weak, suggesting that U2526 samples multiple conformations. (**e**) Aligning the macrolide resistance conferring m2m8A2503 modified ribosome reveals that the C8 methylation is distant from the antibiotic binding (**c**). This suggests that the m2m8A2503 (2,8-dimethyladenosine) modification may not confer resistance to this doxycycline binding mode. (**f,g**) The doxycycline bound (**f**) and unbound (**g**) exit tunnels additionally differ through an absence in waters coordinated to A2077 and A2078 in the bound tunnel, which are readily observed in the unbound exit tunnel (open arrowheads). These may be displaced upon antibiotic binding. 23S rRNA and doxycycline shown as sticks; electron density shown as blue mesh. Colours: nitrogen, blue; oxygen, red; phosphorus, orange; rRNA carbon, light green; doxycycline carbon, dark green.

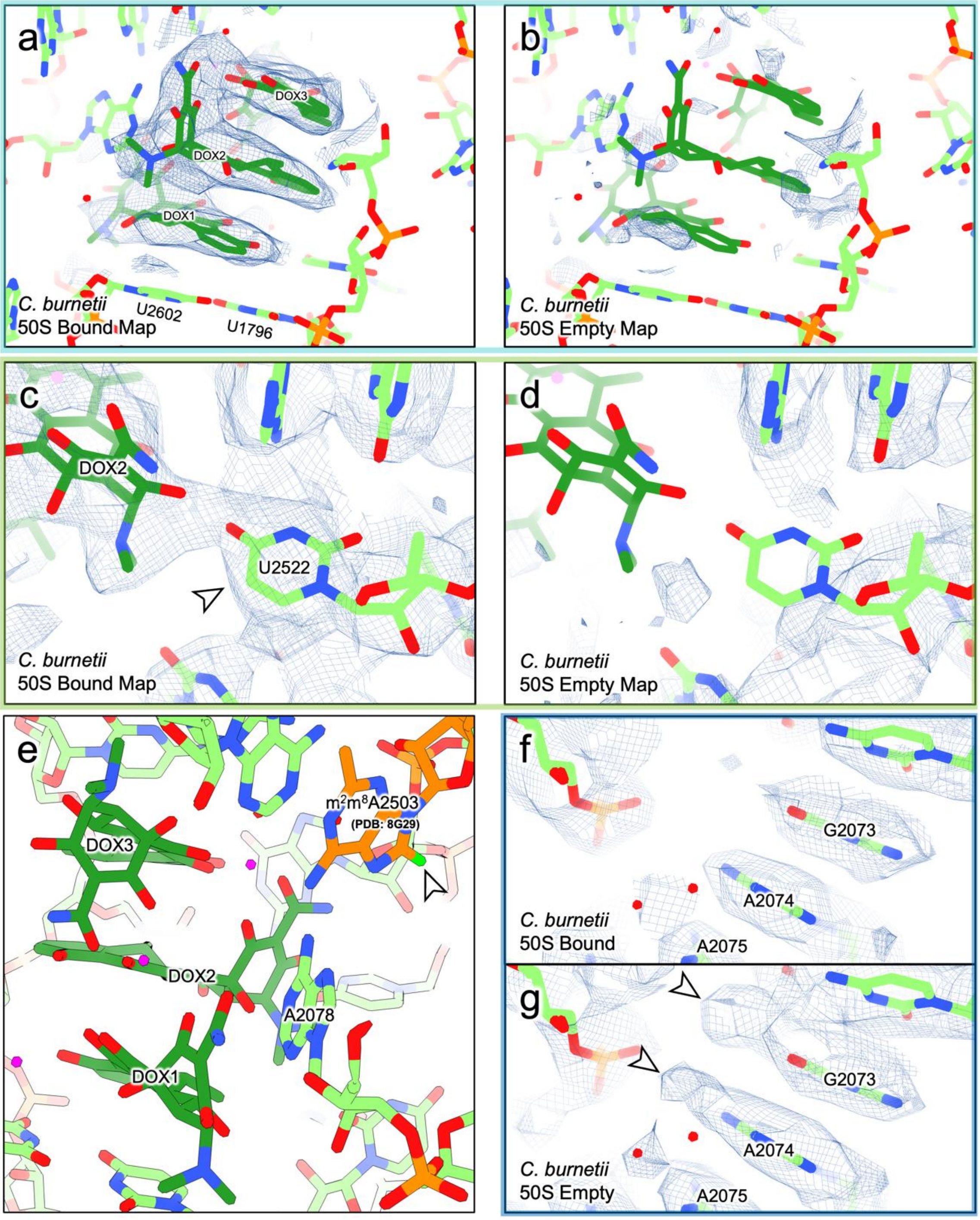
a
DOX3
DOX2
DOX1
C. burnetii
50S Bound Map
U2602
U1796
b
C. burnetii
50S Empty Map
c
DOX2
U2522
C. burnetii
50S Bound Map
d
C. burnetii
50S Empty Map
e
m2m8A2503
(PDB: 8G29)
DOX3
DOX2
A2078
DOX1
f
G2073
A2074
C. burnetii
50S Bound
A2075
g
G2073
A2074
C. burnetii
50S Empty
A2075

Supplementary Figure 12: Structural details of doxycycline within the *C. burnetii* exit tunnel

(**a**) Doxycycline stacking is reliant upon three magnesium ions. The "top" ion (dashed red box, top left) is coordinated by two oxygen atoms each from DOX2 and DOX3 and two water molecules; the central magnesium ion (blue dashed box, bottom left) is coordinated by all three molecules, with each doxycycline donating two oxygen atoms to complete the octahedral magnesium coordination. The third magnesium is coordinated only by DOX1 (bottom right). DOX1 shows a strong π-interaction with A2082 (green dashed box, top right). 23S rRNA and doxycycline shown as sticks; magnesium and water shown as spheres. Colours: nitrogen, blue; oxygen, red; phosphorus, orange; rRNA carbon, light green; doxycycline carbon, dark green; magnesium, pink.
(**b**) Modelling the triple stacking with later generation tetracyclines indicates the stacking we observe for doxycycline is unlikely to be feasible due to clashes with the walls of the exit tunnel. (**c-e**) Previous investigations have displayed tetracyclines and tetracycline-like molecule binding somewhat in similar locations to DOX1, such as sarecycline and tetracenomycin-X. Sarecycline bound to the *Cutibacterium acnes* ribosome (**c**; PDB: 8cvm [11]) shows the 23S rRNA backbone encroaching into the doxycycline stacking space (arrowhead), with sarecycline in a different pose to DOX1 (**d**). Tetracenomycin X bound to the *E. coli* ribosome (**e**; PDB: 6y69 [12]) binds in a similar location to DOX1 but in a very different pose that would not support the magnesium coordination that characterises the doxycycline triple stack. Antibiotics and selected rRNA bases shown as sticks, rRNA as ribbon; sarecycline carbons, salmon; *C. acnes* rRNA, blue; tetracenomycin X, light blue; *E. coli* rRNA, cyan. (**f,g**) Doxycycline recapitulates the hydrogen bond to A2078 (A2058 in *E. coli*) that characterises macrolide binding to the ribosome exit tunnel (**f**; PDB: 6xhx [13]). Here, a water coordinated by the DOX2-DOX3 magnesium is taking the role of the hydroxyl group of the macrolide (**g**). Erythromycin and 23S rRNA shown as sticks; erythromycin carbons, grey; *E. coli* rRNA, light orange.

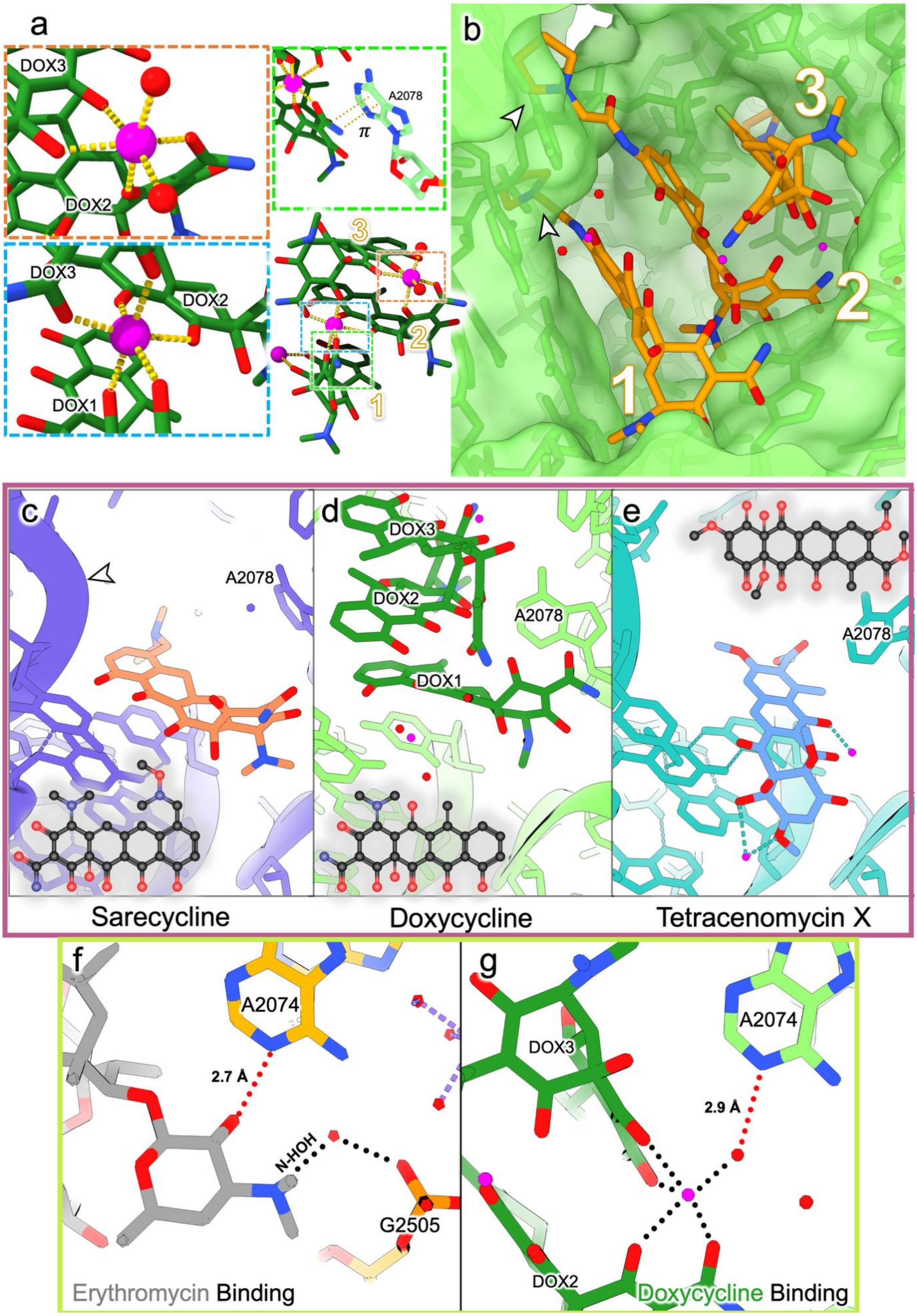
a
DOX3
DOX2
DOX3
DOX2
DOX1
A2078
π
3
2
1
b
3
2
1
c
A2078
Sarecycline
d
DOX3
DOX2
A2078
DOX1
Doxycycline
e
A2078
Tetracenomycin X
f
A2074
2.7 Å
N-HOH
G2505
Erythromycin Binding
g
DOX3
A2074
2.9 Å
DOX2
Doxycycline Binding

Cryo-EM reveals multiple mechanisms of ribosome inhibition by doxycycline

Stuart et al.

Supplementary Figure 13: rRNA bases contacting doxycycline are highly conserved.

(**a**) The consensus of the alignment of *Coxiella* 23S rRNA sequences was aligned with representative rRNA sequences from species that have a ribosome structure in the PDB. Four other proteobacteria (*Acinetobacter baumannii*, *Escherichia coli*, *Pseudomonas aeruginosa*, and *Vibrio natriegens*) and two Hydrobacteria (*Porphyromonas gingivalis* and *Borrelia burgdorferi*) have such structures. Where possible, the sequence of the same strain used for the structure was used; where the genome of this strain is not available in NCBI, an alternative of the same species was chosen. These seven sequences were aligned together using the MUSCLE algorithm [14] in Geneious v2026.0.2. All interactions occur within a 900 base section of the 23S rRNA, with the relevant section of the alignment shown here. Bases within 4 Å of a doxycycline molecule in the 50S ribosome structure in complex with doxycycline are indicated with an arrow. In the structure, these are bases U1796, A2074, A2075, A2078, U2457, G2521, U2522, U2601, U2602, U2625, C2626, and C2627. These are indicated in the alignment with arrows, coloured red for those conserved in all species. Three bases show alterations in one or both of the Hydrobacteria (blue arrows); U1796 and U2602 are both altered to cytosine. These two bases form a highly conserved non-Watson-Crick base pair, which forms with both a U=U or C=C pair. In *P. gingivalis*, C2627 is altered to uracil. This base makes a Watson-Crick base pair (to G2073 in *C. burnetii*). In *P. gingivalis*, the equivalent base alters to adenine to maintain the Watson-Crick base pair. (**b**) Comparison of the *P. gingivalis* (PDB: 9I5T [15]), *B. burgdorferi* (PDB: 8FN2 [16]), and *C. burnetii* ribosomes shows that there is no alteration in the location of these bases (coloured with carbon atoms in cyan, yellow, and green respectively; doxycycline shown as sticks with carbon atoms in white; nitrogen, oxygen, and phosphorus atoms shown in blue, red, and orange respectively; magnesium shown as green spheres). U1796, U2602 (blue arrows), and C2627 (dashed magenta arrow) all contact the doxycycline molecules through base stacking (especially with the phenyl rings of two doxycycline molecules), with no interactions with the hydrogen bond donors or acceptors on the bases. Consequently, these alterations are conservative and are likely to support a doxycycline triple stack in each of these organisms. The alignments are provided as Source Data files.

Cryo-EM reveals multiple mechanisms of ribosome inhibition by doxycycline

Stuart et al.

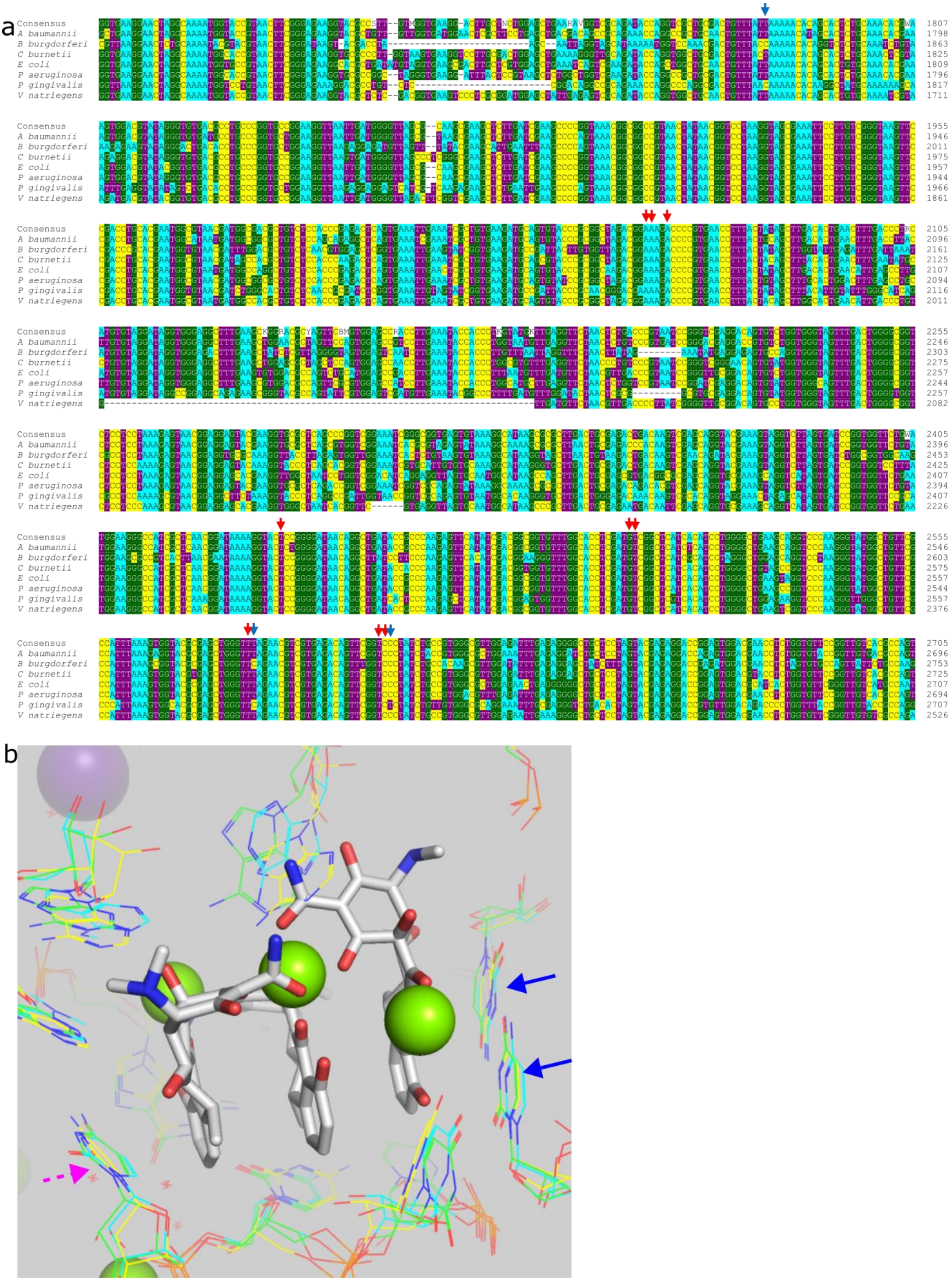

Supplementary Figure 14: Tetracycline minimum inhibitory concentrations against *C. burnetii*

All MIC experiments carried out with a minimum of three repeats and determined as previously described [17]. In line with previously used protocols, cultures were assessed by eye as showing no observable growth, borderline growth, or growth as observed in control wells. The MIC was determined as the lowest concentration that prevented growth. In all cases tested, the replicates gave identical results. Red; antibiotic inhibition of bacterial growth, orange; growth inhibition borderline, green; bacterial growth.

Concentration (µg/mL)	Doxycycline	Tetracycline	Eravacycline	Tigecycline
4.00	N/D	N/D	N/D	
2.00	N/D	N/D	N/D	
1.00	N/D	N/D		
0.50	N/D	N/D		
0.25	N/D	N/D		
0.13	N/D			
0.063				
0.031			N/D	N/D
0.016			N/D	N/D
0.0078			N/D	N/D
0.0039		N/D	N/D	N/D

No growth

Borderline growth

Growth

Cryo-EM reveals multiple mechanisms of ribosome inhibition by doxycycline

Stuart et al.

Supplementary Figure 15: Inactive *E. coli* ribosome identified through doxycycline driven serendipity

In depth classification of our *E. coli* data to identify *C. burnetii*-like binding revealed a subset displaying a substantially rearranged, inactive ribosome. (**a**) Comparison of the unbound (upper) and doxycycline bound (lower) *E. coli* ribosome conformations, showing only nucleotides that display motion in response to doxycycline binding. rRNA shown as ribbon with riboses and bases as sticks. Doxycycline shown as sticks with semi-transparent green surface. Colours: rRNA carbon and ribbon, orange; doxycycline carbon, green; oxygen, red; nitrogen, blue. (**b**) The inactive conformation is incompatible with the typical uL4 extended loop structure (upper). In the doxycycline bound ribosomes this region is unstructured (lower; open arrowhead). (**c**) tRNA blocking nucleotides are stabilised by stacking interactions. U2585 stacks upon C2063 and U2506 stacks upon A2602. To achieve this, both undergo a rotation from their canonical tRNA bound conformation (blue dotted outline) upon doxycycline binding (yellow dotted outline). Green arrows indicate movement of U2585 and A2602 while yellow arrows indicate movement of C2063 and U2506. Translucent neighbouring bases show that doxycycline conformational change is limited only to U2585 and A2602.

The doxycycline bound population had only a minor contribution (~12%, Supplementary Fig. 10) to the bulk 1.77 Å reconstruction, with occasional weak density which would have been ignored were it not for the particular attention paid to this area, driven by the *C. burnetii* ribosome. This is a specific strength of cryo-EM as a technique and demonstrates that there may be more biological phenomena hidden in data already collected and deposited.

Cryo-EM reveals multiple mechanisms of ribosome inhibition by doxycycline

Stuart et al.

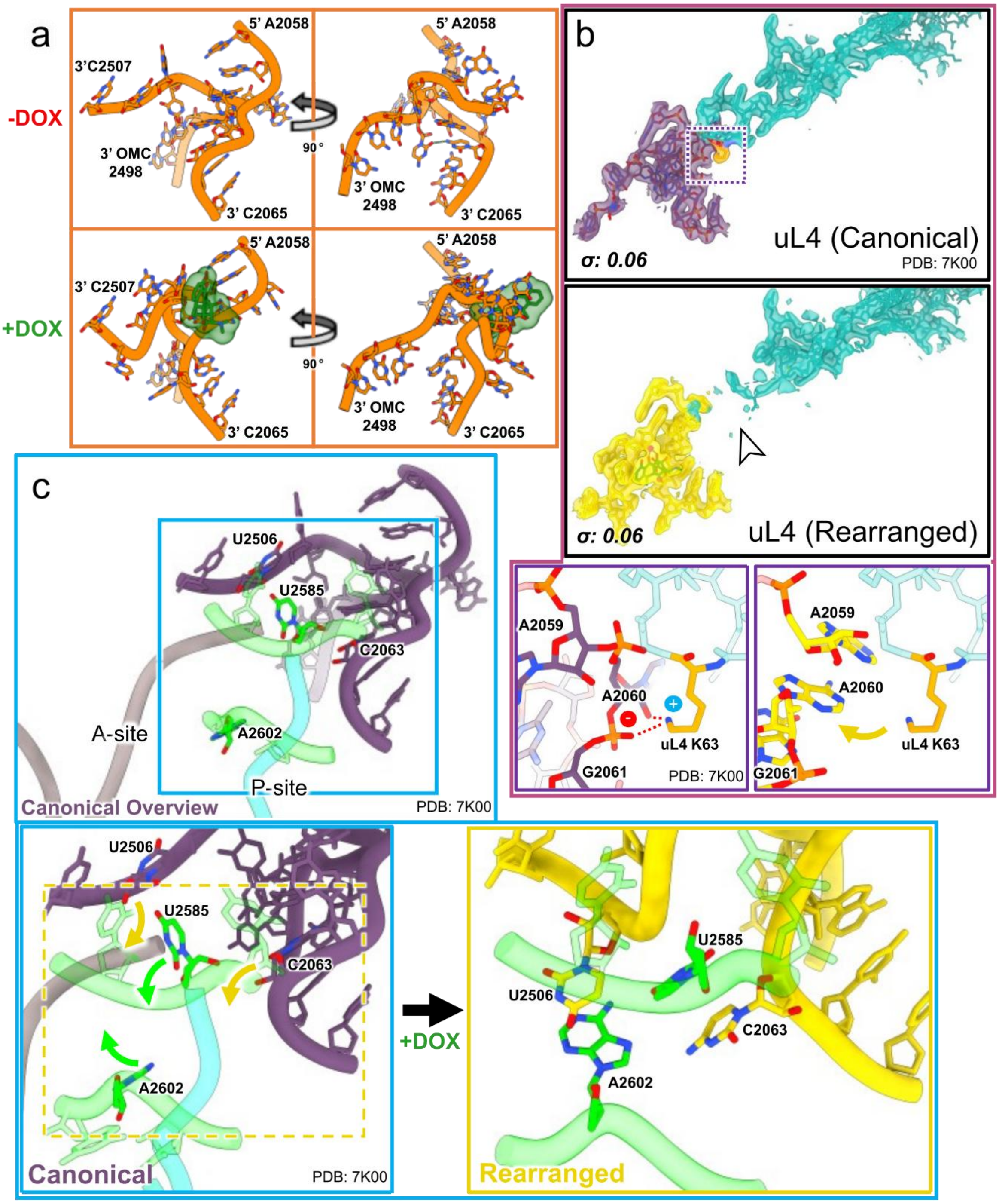

Cryo-EM reveals multiple mechanisms of ribosome inhibition by doxycycline

Stuart et al.

Supplementary Figure 16: Distinct rRNA interactions are made in the doxycycline bound *E. coli* ribosome

(**a,b**) Detailed view of the intra-rRNA interactions made. Only nucleotides that show rearrangement on doxycycline binding are shown (as in Supplementary Fig. 15a). G2502 and G2505 form a stacking interaction (open arrowheads; highlighted in panel **c**). G2502 moves almost 30 Å (28.4 Å) when compared to the canonical ribosome (measuring N1-N1). Similarly, A2059, A2060, G2061 and C2501 are also forming a series of stacking interactions (black arrowhead; highlighted in panels **d** and **e**). 2MA2503 forms a stacking interaction with A2058 (grey arrowhead; highlighted in **f**). With C2063 flipped outwards towards incoming tRNAs, there is a cascade of stacking interactions between A2062, C2064 and C2065 (black arrows). (**g**) View of the interactions made by the 'flipped in' A2062. A2062 now interacts via a canonical base pair with dihydrouridine 2449 and through non-canonical bonding with A2450. Nucleotides that did not undergo rearrangement are made distinct from others shown in this figure through blue colouring. rRNA shown as ribbon with bases and riboses as sticks. Colours: carbon and ribbon (blue for non-rearranged in panel **g**), yellow; oxygen, red; nitrogen, blue. 2MA: 2-methyladenosine; Ψ, pseudouridine; OMC, 2'-*O*-methylcytosine.

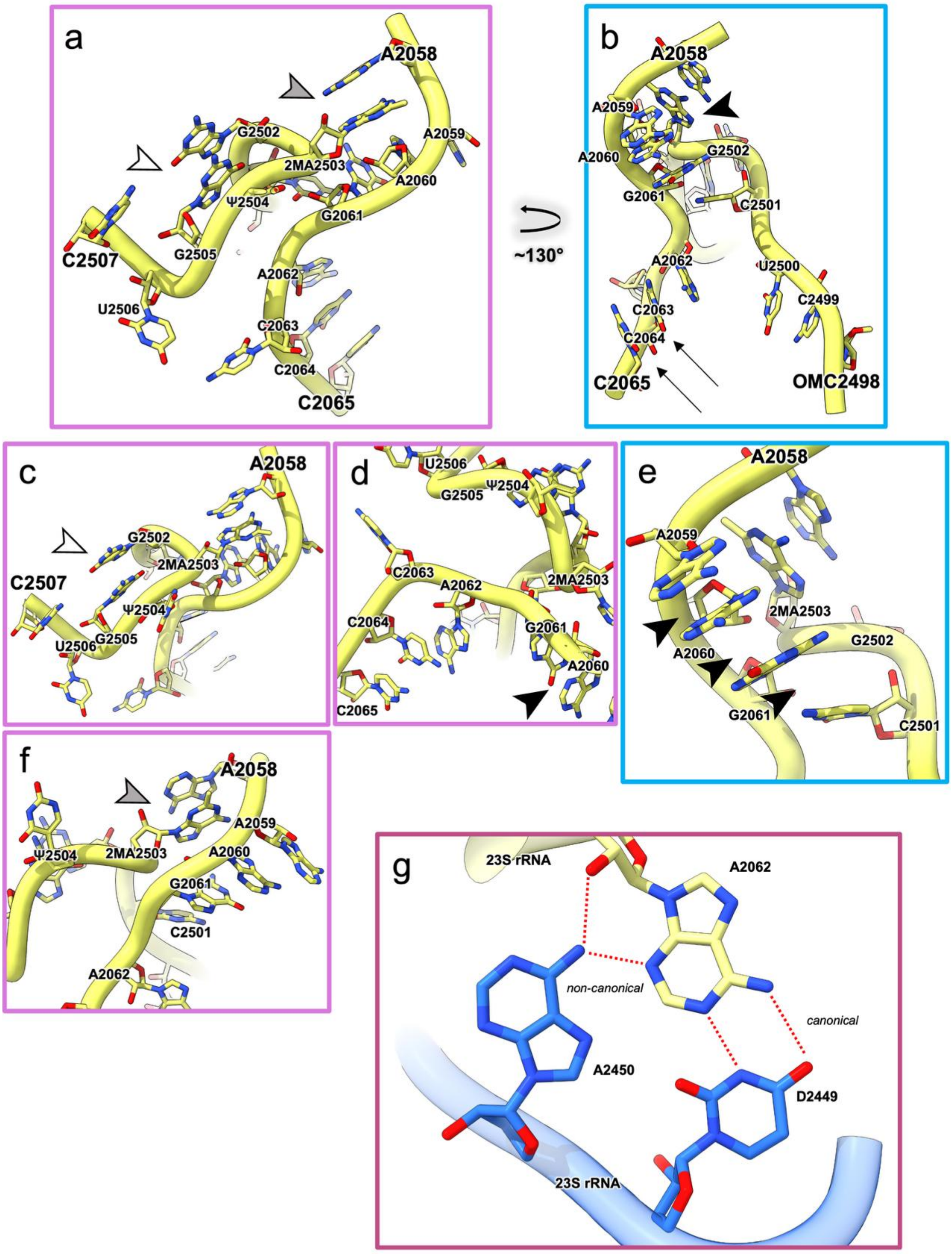
a
A2058
G2502
A2059
2MA2503
A2060
Ψ2504
G2061
C2507
G2505
A2062
U2506
C2063
C2064
C2065
~130°
b
A2058
A2059
A2060
G2502
G2061
C2501
A2062
U2500
C2063
C2499
C2064
C2065
OMC2498
c
A2058
G2502
2MA2503
C2507
Ψ2504
U2506
G2505
d
U2506
G2505
Ψ2504
C2063
2MA2503
A2062
C2064
G2061
A2060
C2065
e
A2058
A2059
2MA2503
G2502
A2060
G2061
C2501
f
A2058
A2059
Ψ2504
2MA2503
A2060
G2061
C2501
A2062
g
23S rRNA
A2062
non-canonical
canonical
A2450
D2449
23S rRNA

Cryo-EM reveals multiple mechanisms of ribosome inhibition by doxycycline

Stuart et al.

Supplementary Figure 17: Cryo-EM map and model validation

Gold standard Fourier shell correlation (FSC) curves for key refined maps from cryoSPARC [18]. Green box corresponds to *Coxiella burnetii* data (**a-e**) and turquoise to *Escherichia coli* (**f-g**). All resolutions called using the 0.143 FSC cut-off.

(**a**) Non-uniform refinement of the doxycycline bound 50S subunit. (**b**) Local refinement of the HPF_{cold} (hibernation promoting factor) bound 30S subunit. (**c**) Local refinement of the doxycycline bound 30S subunit. (**d**) Non-uniform refinement of the apo 50S subunit. (**g**) Non-uniform refinement of the 70S subunit. (**f**) Non-uniform refinement of the doxycycline bound rearranged *E. coli* ribosome. (**g**) Local refinement of the doxycycline bound 30S subunit.

Cryo-EM reveals multiple mechanisms of ribosome inhibition by doxycycline

Stuart et al.

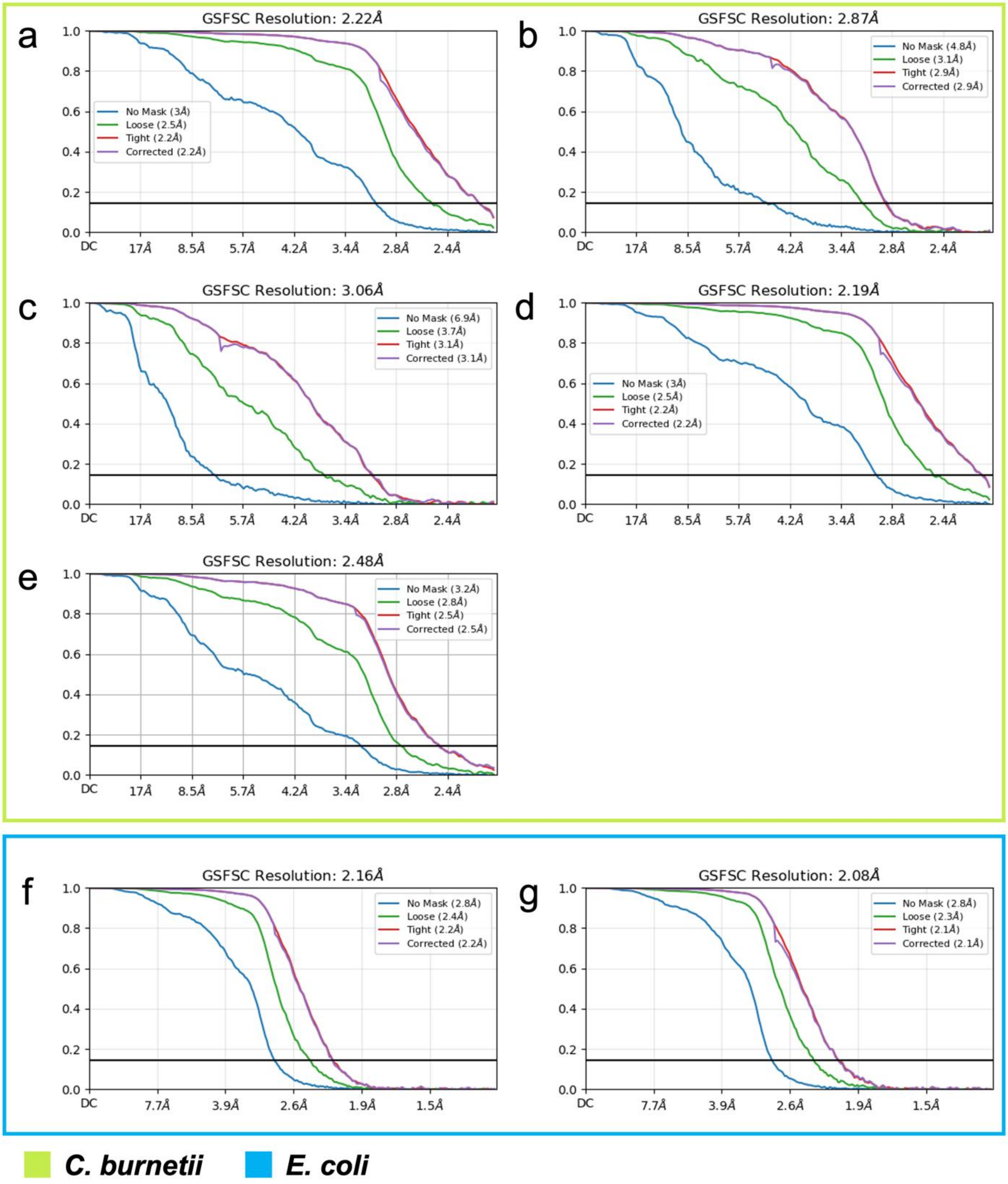

Cryo-EM reveals multiple mechanisms of ribosome inhibition by doxycycline

Stuart et al.

Supplementary Figure 18: Cryo-EM particle angular distribution plots

Euler angle distribution plots of particles from key refined maps from cryoSPARC [18]. Green box corresponds to *Coxiella burnetii* data (**a-e**) and turquoise to *Escherichia coli* (**f-g**).

(**a**) Non-uniform refinement of the doxycycline bound 50S subunit. (**b**) Local refinement of the HPF_{cold} (hibernation promoting factor) bound 30S subunit. (**c**) Local refinement of the doxycycline bound 30S subunit. (**d**) Non-uniform refinement of the apo 50S subunit. (**g**) Non-uniform refinement of the 70S subunit. (**f**) Non-uniform refinement of the doxycycline bound rearranged *E. coli* ribosome. (**g**) Local refinement of the doxycycline bound 30S subunit.

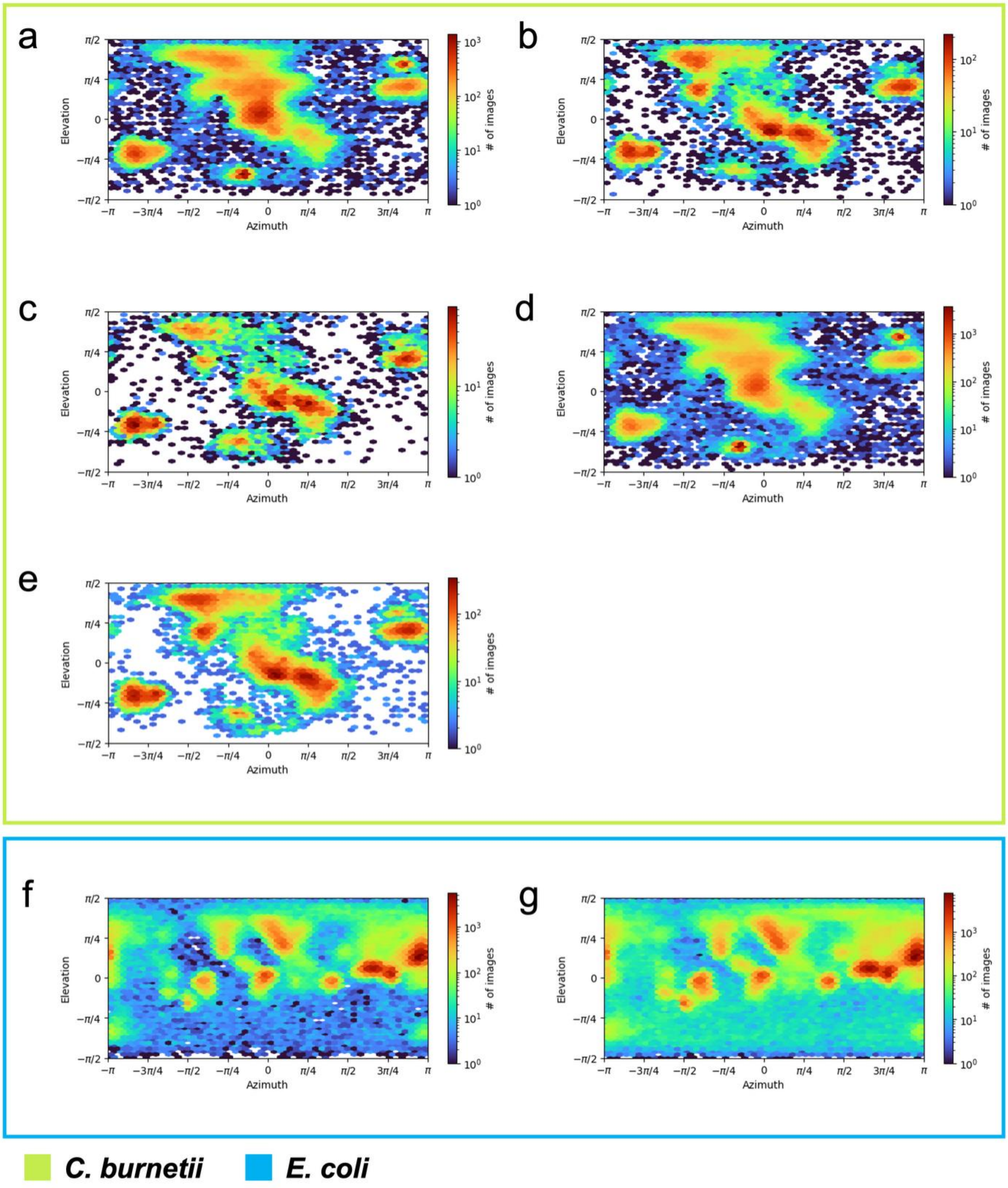
a
b
c
d
e
f
g
Elevation
Azimuth
# of images
π/2
π/4
0
−π/4
−π/2
−π
−3π/4
π/2
3π/4
π
C. burnetii
E. coli

Cryo-EM reveals multiple mechanisms of ribosome inhibition by doxycycline

Stuart et al.

Supplementary Figure 19: Cryo-EM local resolution estimates

Key refined maps from with voxels coloured by estimated local resolution. Local resolution estimates were calculated in cryoSPARC [18] using an FSC cutoff of 0.143. Green box corresponds to *Coxiella burnetii* data (**a-e**) and turquoise to *Escherichia coli* (**f-g**).

(**a**) Non-uniform refinement of the doxycycline bound 50S subunit. (**b**) Local refinement of the HPF_{cold} (hibernation promoting factor) bound 30S subunit. (**c**) Local refinement of the doxycycline bound 30S subunit. (**d**) Non-uniform refinement of the apo 50S subunit. (**g**) Non-uniform refinement of the 70S subunit. (**f**) Non-uniform refinement of the doxycycline bound rearranged *E. coli* ribosome. (**g**) Local refinement of the doxycycline bound 30S subunit.

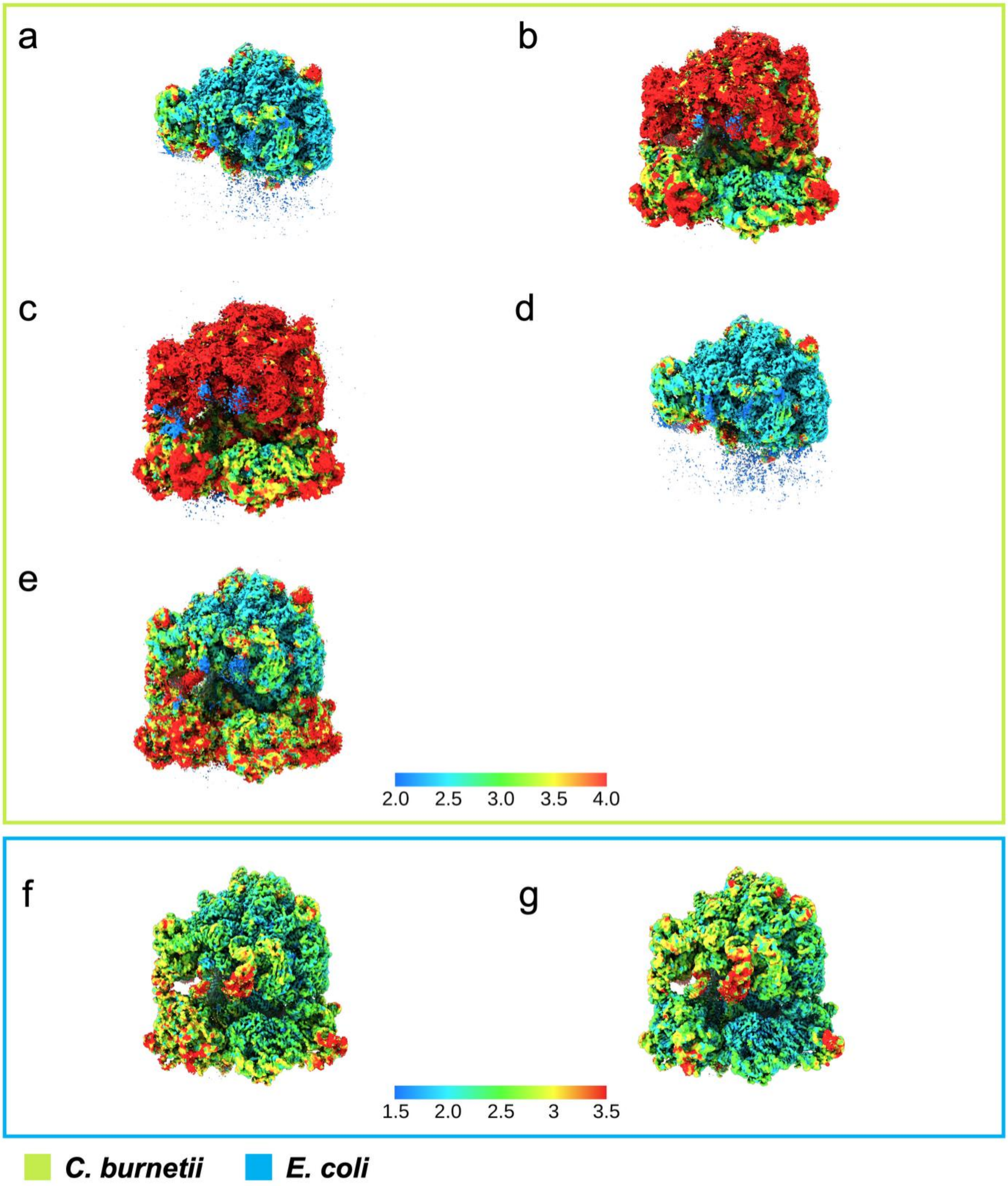
a
b
c
d
e
2.0 2.5 3.0 3.5 4.0
f
g
1.5 2.0 2.5 3 3.5
C. burnetii
E. coli

Cryo-EM reveals multiple mechanisms of ribosome inhibition by doxycycline

Stuart et al.

Supplementary Figure 20: Cryo-EM map density at doxycycline binding sites

Representative images of cryo-EM map density for doxycycline bound to *C. burnetii* and *E. coli* ribosomes. Density is shown in transparent grey surface and mesh representation.

Images are outlined by species, green for *C. burnetii* and blue for *E. coli*. Top two rows show density for doxycycline bound in the respective 50S binding sites and the bottom two rows for the canonical 30S binding sites. Images between the columns are related by a 90° rotation about the antibiotic and display a consistent contour level. Colours: doxycycline carbon atoms are shown in dark green, oxygen in red and nitrogen in blue. Coordinating magnesium atoms are shown in bright green in *C. burnetii* images and magenta in *E. coli* images. Remaining bulk ribosomal atoms are displayed in a uniform colour; *C. burnetii* 50S pale green, *C. burnetii* 30S pale yellow, *E. coli* 50S light blue and *E. coli* 30S pale green-grey.

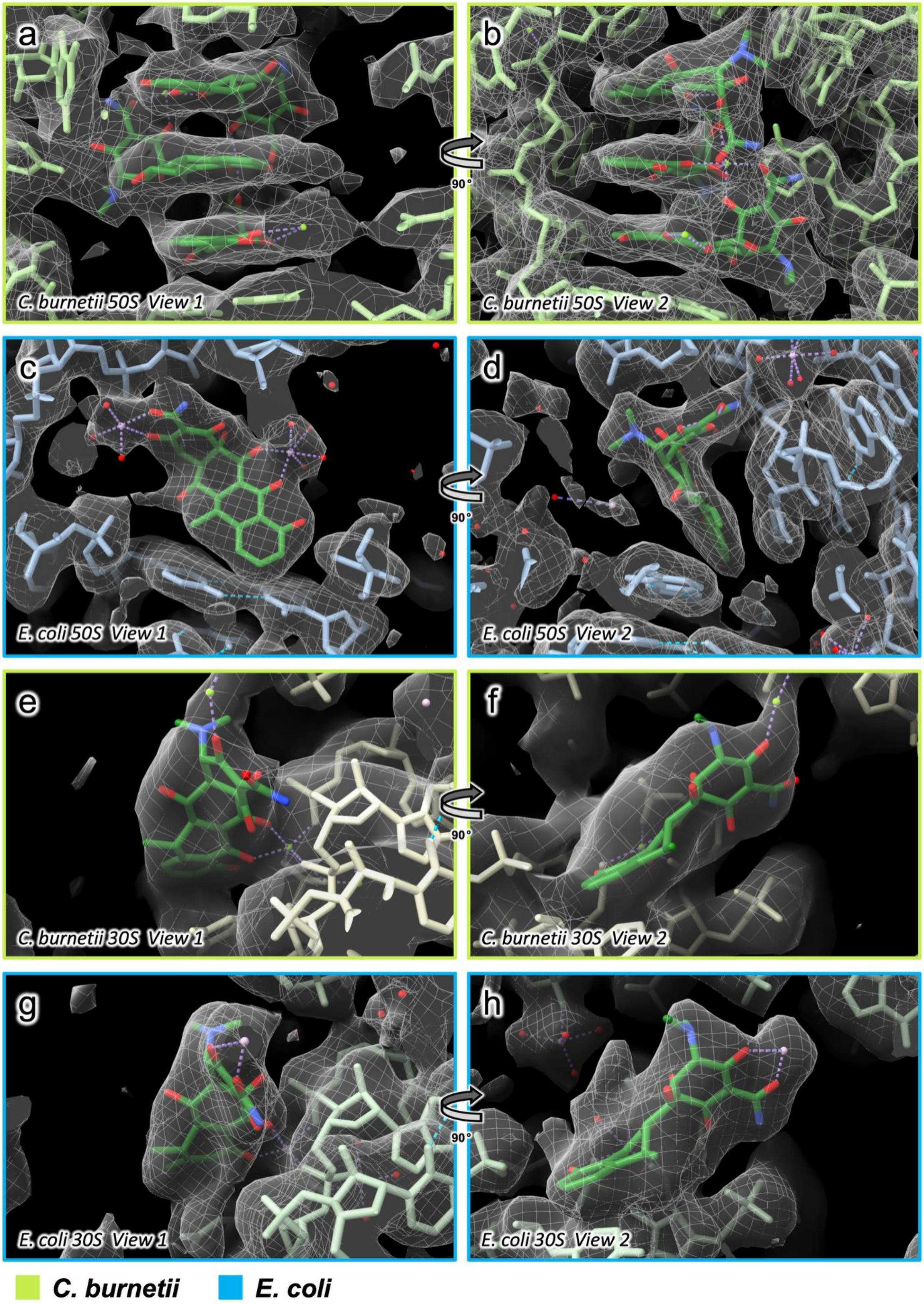
a
b
c
d
e
f
g
h
90°
C. burnetii 50S View 1
C. burnetii 50S View 2
E. coli 50S View 1
E. coli 50S View 2
C. burnetii 30S View 1
C. burnetii 30S View 2
E. coli 30S View 1
E. coli 30S View 2
C. burnetii
E. coli

Supplementary discussion

HPF_{cold} is a distinct class of ribosome hibernation factor

HPF_{cold} provides the structure of a hibernation factor with a cold shock domain, representing a distinct class of ribosome hibernation factor family [19]. As such, we propose to name this class HPF_{cold}. It is structurally distinct from existing HPF family members; HPF_{long}, HPF_{short} or ribosome associated inhibitor A (RaiA) (Supplementary Fig. 4e). These are the primary hibernation factors in bacteria and dictate the specific form of ribosome hibernation that occurs. In gram-negatives, 100S ribosome dimers are formed through a combination of HPF_{short} and ribosome modulation factor (RMF). HPF_{short} occupies the tRNA binding cleft with RMF driving dimerising through a copy on the partner ribosome [5]. In other species, both roles are carried out by HPF_{long}, dimerising through its C-terminal domain (CTD) via an elongated linker [6]. 70S hibernation occurs through RaiA, with a C-terminal extension preventing 100S formation by clashing with RMF [5]. *C. burnetii* lacks a gene for both HPF_{long} and RMF, with its only other known hibernation factor (*CBU_0745*) annotated as RaiA. However, *CBU_0745* lacks the characteristic C-terminal extension of RaiA, instead appearing to classify as HPF_{short}. The prokaryotic 100S dimerization interface falls upon the mRNA exit tunnel region, conferring protection not provided by HPF_{short}. Loss of 100S hibernation is therefore associated with lower virulence and survival rates, with increased susceptibility to RNAses [20]. RMF confers protection to the 3' 16S [21]; it appears that HPF_{cold} provides similar protection since HPF_{cold} CSD binds and stabilises the anti-Shine Dalgarno sequence, blocking RNAse access. HPF_{cold} may confer a high enough degree of protection from RNAses to keep ribosomes viable over a long time period, presumably a crucial

capability for *C. burnetii* during the extended hibernation periods required of the SCV form [22]. We were unable to identify ribosome dimers in our single particle data (although it is feasible any dimerised ribosomes would be lost during sample preparation). While an as yet unidentified factor (or even HPF_{cold} CSD dimerization) could induce 100S assemblies, HPF_{cold} may render this need obsolete by providing 100S-like capabilities in a 70S form. A transposon mutagenesis screen suggests HPF_{cold} is not essential for *C. burnetii* growth in media at 37 °C [23]. Future experiments investigating the effects of a HPF_{cold} knockout would be valuable for delineating its role in the bacterial hibernation and stress response, in *C. burnetii* and other prokaryotes. HPF_{cold} overlaps with the 30S tetracycline binding site. To our knowledge, the effect of hibernation factor mutants on tetracycline efficacy have not been tested. However, *Legionella pneumophila* encodes an HPF_{cold} orthologue and does not appear to display particular sensitivity to doxycycline [24].

Across the phylogenetic tree (Fig. 2f), the PF02482 (sigma-54) and PF00313 (cold shock domain) fusion is present in all major proteobacterial classes but with uneven distribution, consistent with either lineage-specific expansions or sampling biases. Fused-domain homologs are also present in Cyanobacteria and several non-proteobacterial phyla, producing a polyphyletic distribution. Mapping metabolic annotations onto the tree shows the fusion recurring in specialist guilds such as nitrifiers, anammox bacteria and multiple phototrophic lineages suggesting repeated recruitment into redox-intensive lifestyles across oxic–anoxic gradients and, therefore, ecological niche expansion rather than a single constrained function. Taken together, this pattern is consistent with a mix of processes: repeated, independent PF02482–PF00313 fusion events, horizontal transfers of an existing

fusion, and lineage-specific losses, with retention where the fused domain architecture is advantageous. Within Gammaproteobacteria, *Coxiella* sequences form a distinct, well-supported subclade (*Coxiellaceae/Legionellales*), clearly separated from other gammaproteobacterial homologs. This placement points to lineage-specific functional constraints under an intracellular lifestyle rather than a recent cross-phylum acquisition. Notably, Alphaproteobacteria display the broadest radiation, with multiple sub-clusters that could reflect diversification of the fusion within this class or simply denser genome sampling. Conversely, the absence of the fused architecture in several major bacterial groups in our dataset points to niche-specific utility and possible functional constraints on maintaining this two-domain fusion. Together, these patterns place HPF_{cold} in a clear phylogenetic context and support a scenario where the fusion has a long evolutionary history punctuated by clade-specific expansions and losses.

A doxycycline triple stack blocks the *C. burnetii* ribosome exit tunnel

The *C. burnetii* ribosomal exit tunnel in the 50S subunit is filled by a stack of three doxycycline molecules (Fig. 4a-c). This stack relies upon a magnesium induced rotated amide conformation (Supplementary Fig. 12a), as has been observed in certain small molecule crystallisation conditions [25]. In comparison to the unbound structure, DOX2 stabilises 23S rRNA U2522 (U2506) (Supplementary Fig. 11c-d), involved in sensing ribosomal stalling [26]. U2522 takes a ‘luminal’, stalled-like conformation showing a rotation which has broken the hydrogen bond pair to G2599 (G2583). Additionally, A2078 (A2062), which is vital for sensing translational arrest [27] is stabilised by DOX1 through a Pi stack of the amide group (Supplementary Fig. 11e, 12a).

Sarecycline bound to the *C. acnes* ribosome most closely resembles the DOX1 binding mode, however the *C. acnes* phosphate backbone conformation between U2625-C2636 (2609-2610) would abrogate binding of doxycycline molecule 2 (Supplementary Fig. 12c). Sarecycline is also too distant to interact with U2522 (U2506) [11]. Like DOX1, tetracenomycin X stacks on U2602 but is instead rotated, moving out of range of the magnesium ion DOX1 utilises (Supplementary Fig. 12e) [12]. Interestingly, some elements of macrolide binding are re-capitulated by the doxycycline stack, notably, the single conserved hydrogen bond required for macrolide binding [13]. In macrolides, the desosamine 2-OH group hydrogen bonds to A2074 (A2058). This role is taken by a water coordinated by the DOX2-3 magnesium, H-bonding to the N1 of A2074 (A2058) (Supplementary Fig. 12f-g). Dimethylation of *E. coli* A2058 confers macrolide resistance through the disruption of a water mediated interaction. This same interaction is not imitated in the doxycycline binding, which may enable doxycycline to bind even in the presence of a dimethylated A2074 [13].

Erythromycin resistance is conferred by the ErmCL peptide, which interacts with U2506 (*E. coli*) to trigger translational arrest [26]. The tunnel blockage by doxycycline may preclude a similar peptide-based resistance mechanism. Whilst resistance to many antibiotics targeting the PTC (chloramphenicol, linezolid etc) is conferred by methylation of 2503 (*E. coli*) C8 [28, 29], given the distance from the bound doxycycline molecules, this would be unlikely to obstruct *C. burnetii* doxycycline binding (Supplementary Fig. 11e). We note that *C. burnetii* appears to lack a gene encoding a tetracycline destructase, a recently identified family of bacterial resistance enzymes [30, 31].

We carried out our single particle analysis at a concentration of 50 μM (~22 mg/L), the higher end of physiological conditions in the blood serum of patients, which can reach 8 mg/L [32]. We also note that doxycycline is highly lipophilic and likely achieves higher concentrations in cell compared to in serum. While human data was not available and so a direct comparison is impossible, doxycycline displayed up to 17x higher concentration into polymorphonuclear leukocytes compared to interstitial fluid in horses [33]. Fundamentally, the concentration of doxycycline within the bacterium at MIC is not known and so experimentation to observe antibiotic activity against the *in-situ* ribosome using cryo electron-tomography would be a useful follow up study.

We also note that while our results in *C. burnetii* were observed with three doxycycline molecules, we see no reason to constrain this to a single antibiotic. In the first case, combinatorial therapy with existing, licensed tetracycline family members may further optimise the binding within the stack. This could concomitantly achieve higher affinities and most crucially, would sidestep extensive development and clinical trial processes. It may be that different combinations would be optimal for different organisms. Subsequent structure guided development of new molecules, fully exploiting each binding site within the stack could yield a species-specific gold standard, most effective treatment.

Cryo-EM reveals multiple mechanisms of ribosome inhibition by doxycycline

Stuart et al.

Supplementary References

1. Ban, N., R. Beckmann, J.H.D. Cate, J.D. Dinman, F. Dragon, S.R. Ellis, D.L.J. Lafontaine, L. Lindahl, A. Liljas, J.M. Lipton, M.A. McAlear, P.B. Moore, H.F. Noller, J. Ortega, V.G. Panse, V. Ramakrishnan, C.M.T. Spahn, T.A. Steitz, M. Tchorzewski, D. Tollervey, A.J. Warren, J.R. Williamson, D. Wilson, A. Yonath, and M. Yusupov, *A new system for naming ribosomal proteins.* Current Opinion in Structural Biology, 2014. **24**: p. 165-169.
2. Abramson, J., J. Adler, J. Dunger, R. Evans, T. Green, A. Pritzel, O. Ronneberger, L. Willmore, A.J. Ballard, J. Bambrick, S.W. Bodenstein, D.A. Evans, C.C. Hung, M. O'Neill, D. Reiman, K. Tunyasuvunakool, Z. Wu, A. Zemgulyte, E. Arvaniti, C. Beattie, O. Bertolli, A. Bridgland, A. Cherepanov, M. Congreve, A.I. Cowen-Rivers, A. Cowie, M. Figurnov, F.B. Fuchs, H. Gladman, R. Jain, Y.A. Khan, C.M.R. Low, K. Perlin, A. Potapenko, P. Savy, S. Singh, A. Stecula, A. Thillaisundaram, C. Tong, S. Yakneen, E.D. Zhong, M. Zielinski, A. Zidek, V. Bapst, P. Kohli, M. Jaderberg, D. Hassabis, and J.M. Jumper, *Accurate structure prediction of biomolecular interactions with AlphaFold 3.* Nature, 2024. **630**(8016): p. 493-500.
3. Schindelin, H., W. Jiang, M. Inouye, and U. Heinemann, *Crystal structure of CspA, the major cold shock protein of Escherichia coli.* Proc Natl Acad Sci U S A, 1994. **91**(11): p. 5119-23.
4. Pettersen, E.F., T.D. Goddard, C.C. Huang, E.C. Meng, G.S. Couch, T.I. Croll, J.H. Morris, and T.E. Ferrin, *UCSF ChimeraX: Structure visualization for researchers, educators, and developers.* Protein Sci, 2021. **30**(1): p. 70-82.
5. Polikanov, Y.S., G.M. Blaha, and T.A. Steitz, *How hibernation factors RMF, HPF, and YfiA turn off protein synthesis.* Science, 2012. **336**(6083): p. 915-8.
6. Franken, L.E., G.T. Oostergetel, T. Pijning, P. Puri, V. Arkhipova, E.J. Boekema, B. Poolman, and A. Guskov, *A general mechanism of ribosome dimerization revealed by single-particle cryo-electron microscopy.* Nat Commun, 2017. **8**(1): p. 722.
7. Sievers, F., A. Wilm, D. Dineen, T.J. Gibson, K. Karplus, W. Li, R. Lopez, H. McWilliam, M. Remmert, J. Soding, J.D. Thompson, and D.G. Higgins, *Fast, scalable generation of high-quality protein multiple sequence alignments using Clustal Omega.* Mol Syst Biol, 2011. **7**: p. 539.
8. Robert, X. and P. Gouet, *Deciphering key features in protein structures with the new ENDscript server.* Nucleic Acids Res, 2014. **42**(Web Server issue): p. W320-4.
9. Ashkenazy, H., E. Erez, E. Martz, T. Pupko, and N. Ben-Tal, *ConSurf 2010: calculating evolutionary conservation in sequence and structure of proteins and nucleic acids.* Nucleic Acids Res, 2010. **38**(Web Server issue): p. W529-33.
10. Li, Z., X. Ge, Y. Zhang, L. Zheng, S. Sanyal, and N. Gao, *Cryo-EM structure of Mycobacterium smegmatis ribosome reveals two unidentified ribosomal proteins close to the functional centers.* Protein & Cell, 2017. **9**(4): p. 384-388.
11. Lomakin, I.B., S.C. Devarkar, S. Patel, A. Grada, and C.G. Bunick, *Sarecycline inhibits protein translation in Cutibacterium acnes 70S ribosome using a two-site mechanism.* Nucleic Acids Res, 2023. **51**(6): p. 2915-2930.
12. Osterman, I.A., M. Wieland, T.P. Maviza, K.A. Lashkevich, D.A. Lukianov, E.S. Komarova, Y.V. Zakalyukina, R. Buschauer, D.I. Shiriaev, S.A. Leyn, J.E. Zlamal,

M.V. Biryukov, D.A. Skvortsov, V.N. Tashlitsky, V.I. Polshakov, J. Cheng, Y.S. Polikanov, A.A. Bogdanov, A.L. Osterman, S.E. Dmitriev, R. Beckmann, O.A. Dontsova, D.N. Wilson, and P.V. Sergiev, *Tetracenomycin X inhibits translation by binding within the ribosomal exit tunnel.* Nat Chem Biol, 2020. **16**(10): p. 1071-1077.

13. Svetlov, M.S., E.A. Syroegin, E.V. Aleksandrova, G.C. Atkinson, S.T. Gregory, A.S. Mankin, and Y.S. Polikanov, *Structure of Erm-modified 70S ribosome reveals the mechanism of macrolide resistance.* Nat Chem Biol, 2021. **17**(4): p. 412-420.
14. Edgar, R.C., *MUSCLE: multiple sequence alignment with high accuracy and high throughput.* Nucleic Acids Research, 2004. **32**(5): p. 1792-1797.
15. Hiregange, D.-G., S. Samiya, D. Mizgalska, E. Ben-Zeev, M. Waghalter, A. Rivalta, K S. Rajan, Y. Halfon, E. Breiner-Goldstein, I. Kaczmarczyk, A. Sroka, M. Taoka, Y. Nobe, T. Isobe, S. Paukner, E. Zimmerman, A. Bashan, J. Potempa, and A. Yonath, *Structural studies of ribosome from an anaerobic Bacteroidetes human pathogen Porphyromonas gingivalis.* Nucleic Acids Research, 2025. **53**(10).
16. Sharma, M.R., S.R. Manjari, E.K. Agrawal, P. Keshavan, R.K. Koripella, S. Majumdar, A.L. Marcinkiewicz, Y.P. Lin, R.K. Agrawal, and N.K. Banavali, *The structure of a hibernating ribosome in a Lyme disease pathogen.* Nat Commun, 2023. **14**(1): p. 6961.
17. Clay, K.A., M.G. Hartley, P. Russell, and I.H. Norville, *Use of axenic media to determine antibiotic efficacy against coxiella burnetii.* Int J Antimicrob Agents, 2018. **51**(5): p. 806-808.
18. Punjani, A., J.L. Rubinstein, D.J. Fleet, and M.A. Brubaker, *cryoSPARC: algorithms for rapid unsupervised cryo-EM structure determination.* Nat Methods, 2017. **14**(3): p. 290-296.
19. Prossliner, T., K. Skovbo Winther, M.A. Sorensen, and K. Gerdes, *Ribosome Hibernation.* Annu Rev Genet, 2018. **52**: p. 321-348.
20. Gohara, D.W. and M.F. Yap, *Survival of the drowsiest: the hibernating 100S ribosome in bacterial stress management.* Curr Genet, 2018. **64**(4): p. 753-760.
21. Prossliner, T., K. Gerdes, M.A. Sorensen, and K.S. Winther, *Hibernation factors directly block ribonucleases from entering the ribosome in response to starvation.* Nucleic Acids Res, 2021. **49**(4): p. 2226-2239.
22. Kersh, G.J., K.A. Fitzpatrick, J.S. Self, R.A. Priestley, A.J. Kelly, R.R. Lash, N. Marsden-Haug, R.J. Nett, A. Bjork, R.F. Massung, and A.D. Anderson, *Presence and persistence of Coxiella burnetii in the environments of goat farms associated with a Q fever outbreak.* Appl Environ Microbiol, 2013. **79**(5): p. 1697-703.
23. Metters, G., C. Hemsley, I. Norville, and R. Titball, *Identification of essential genes in Coxiella burnetii.* Microb Genom, 2023. **9**(2): p. 944.
24. Bruin, J.P., E.P.F. Ijzerman, J.W. den Boer, J.W. Mouton, and B.M.W. Diederen, *Wild-type MIC distribution and epidemiological cut-off values in clinical Legionella pneumophila serogroup 1 isolates.* Diagnostic Microbiology and Infectious Disease, 2012. **72**(1): p. 103-108.
25. Santos, O.M., D.M. Silva, F.T. Martins, A.O. Legendre, L.C. Azarias, I.M. Rosa, P.P. Neves, M.B. de Araujo, and A.C. Doriguetto, *Protonation pattern, tautomerism, conformerism, and physicochemical analysis in new crystal forms of the antibiotic doxycycline.* Crystal growth & design, 2014. **14**(8): p. 3711-3726.

26. Koch, M., J. Willi, U. Pradere, J. Hall, and N. Polacek, *Critical 23S rRNA interactions for macrolide-dependent ribosome stalling on the ErmCL nascent peptide chain.* Nucleic Acids Res, 2017. **45**(11): p. 6717-6728.
27. Vazquez-Laslop, N., H. Ramu, D. Klepacki, K. Kannan, and A.S. Mankin, *The key function of a conserved and modified rRNA residue in the ribosomal response to the nascent peptide.* EMBO J, 2010. **29**(18): p. 3108-17.
28. Kehrenberg, C., S. Schwarz, L. Jacobsen, L.H. Hansen, and B. Vester, *A new mechanism for chloramphenicol, florfenicol and clindamycin resistance: methylation of 23S ribosomal RNA at A2503.* Mol Microbiol, 2005. **57**(4): p. 1064-73.
29. Aleksandrova, E.V., K.J.Y. Wu, B.I.C. Tresco, E.A. Syroegin, E.E. Killeavy, S.M. Balasanyants, M.S. Svetlov, S.T. Gregory, G.C. Atkinson, A.G. Myers, and Y.S. Polikanov, *Structural basis of Cfr-mediated antimicrobial resistance and mechanisms to evade it.* Nat Chem Biol, 2024. **20**(7): p. 867-876.
30. Blake, K.S., H. Kumar, A. Loganathan, E.E. Williford, L. Diorio-Toth, Y.P. Xue, W.K. Tang, T.P. Campbell, D.D. Chong, S. Angtuaco, T.A. Wencewicz, N.H. Tolia, and G. Dantas, *Sequence-structure-function characterization of the emerging tetracycline destructase family of antibiotic resistance enzymes.* Commun Biol, 2024. **7**(1): p. 336.
31. Forsberg, K.J., S. Patel, T.A. Wencewicz, and G. Dantas, *The Tetracycline Destructases: A Novel Family of Tetracycline-Inactivating Enzymes.* Chem Biol, 2015. **22**(7): p. 888-97.
32. Hopkins, A.M., J. Wojciechowski, A.Y. Abuhelwa, S. Mudge, R.N. Upton, and D.J.R. Foster, *Population Pharmacokinetic Model of Doxycycline Plasma Concentrations Using Pooled Study Data.* Antimicrobial Agents and Chemotherapy, 2017. **61**(3): p. 10.1128/aac.02401-16.
33. Davis, J.L., J.H. Salmon, and M.G. Papich, *Pharmacokinetics and tissue distribution of doxycycline after oral administration of single and multiple doses in horses.* Am J Vet Res, 2006. **67**(2): p. 310-6.
