# Supplementary material for: Cryo-EM reveals multiple mechanisms of ribosome inhibition by doxycycline
Source: Nat Commun. 2026 Jun 1;17:7049. doi: 10.1038/s41467-026-73421-5 (PMC13392368; doi:10.1038/s41467-026-73421-5)
Supplement: Supplementary file 2 — Description of Additional Supplementary Files [file 41467_2026_73421_MOESM2_ESM.pdf]

Description of Additional Supplementary Files

File Name: Supplementary Movie 1
Description: Summary of key results. The movie illustrates the structures of the Coxiella burnetii and Escherichia coli ribosomes in complex with doxycycline. The Coxiella hibernation promoting factor (HPFcold), Coxiellaceae Large Subunit Peptide (CLaSP), doxycycline triple stack in the C. burnetii ribosome nascent peptide exist tunnel, and doxycycline rearrangement of the E. coli ribosome peptidyl transferase centre are illustrated. Electron density of key elements is illustrated.
