# Supplementary material for: Cryo-EM reveals multiple mechanisms of ribosome inhibition by doxycycline
Source: Nat Commun. 2026 Jun 1;17:7049. doi: 10.1038/s41467-026-73421-5 (PMC13392368; doi:10.1038/s41467-026-73421-5)
Supplement: Supplementary file 4 — Reporting Summary [file 41467_2026_73421_MOESM4_ESM.pdf]

nature portfolio

Corresponding author(s): DAPR NCOMMS-25-90179A

Last updated by author(s): Apr 22, 2026

Reporting Summary

Nature Portfolio wishes to improve the reproducibility of the work that we publish. This form provides structure for consistency and transparency in reporting. For further information on Nature Portfolio policies, see our Editorial Policies and the Editorial Policy Checklist.

Statistics

For all statistical analyses, confirm that the following items are present in the figure legend, table legend, main text, or Methods section.

n/a	Confirmed	
☐	☒	The exact sample size (*n*) for each experimental group/condition, given as a discrete number and unit of measurement
☐	☒	A statement on whether measurements were taken from distinct samples or whether the same sample was measured repeatedly
☒	☐	The statistical test(s) used AND whether they are one- or two-sided<br>*Only common tests should be described solely by name; describe more complex techniques in the Methods section.*
☒	☐	A description of all covariates tested
☒	☐	A description of any assumptions or corrections, such as tests of normality and adjustment for multiple comparisons
☐	☒	A full description of the statistical parameters including central tendency (e.g. means) or other basic estimates (e.g. regression coefficient) AND variation (e.g. standard deviation) or associated estimates of uncertainty (e.g. confidence intervals)
☒	☐	For null hypothesis testing, the test statistic (e.g. *F*, *t*, *r*) with confidence intervals, effect sizes, degrees of freedom and *P* value noted<br>*Give P values as exact values whenever suitable.*
☒	☐	For Bayesian analysis, information on the choice of priors and Markov chain Monte Carlo settings
☒	☐	For hierarchical and complex designs, identification of the appropriate level for tests and full reporting of outcomes
☒	☐	Estimates of effect sizes (e.g. Cohen's *d*, Pearson's *r*), indicating how they were calculated

Our web collection on statistics for biologists contains articles on many of the points above.

Software and code

Policy information about availability of computer code

Data collection	Single particle Cryo-EM data were collected on a ThermoFisher Titan Krios electron microscope using the Smart EPU (v3.4.0.5) software.
Data analysis	Electron micrographs were processed with CryoSPARC v4.6, particles were picked with crYOLO 1.9.3.<br>Maps were visualised using ChimeraX v1.9.<br>Initial models were built either manually in Coot v0.9.8.8, predicted with AlphaFold 2.2 or 3 or interpreted with ModelAngelo 1.0.1. Post-processing to aid map interpretation was carried out with EMReady 2.0.<br>Models were built with Coot v0.9.8.8 or Isolde v1.9<br>Models were refined using Refmac 5.8.0258

For manuscripts utilizing custom algorithms or software that are central to the research but not yet described in published literature, software must be made available to editors and reviewers. We strongly encourage code deposition in a community repository (e.g. GitHub). See the Nature Portfolio guidelines for submitting code & software for further information.

Data

Policy information about availability of data

All manuscripts must include a data availability statement. This statement should provide the following information, where applicable:

- Accession codes, unique identifiers, or web links for publicly available datasets
- A description of any restrictions on data availability
- For clinical datasets or third party data, please ensure that the statement adheres to our policy

DATA AVAILABILITY
PDB accession codes and cryo-EM maps have been deposited in the Protein Data Bank and the Electron Microscopy Data Bank repositories respectively. These are set to become available publicly upon publication of this work. All raw micrograph data will be made available at the Electron Microscopy Public Image Archive (EMPIAR) following publication of this work.

PDB code, Structure title, PDB Accession, EMDB Accession
9SLG: Doxycycline Bound 50S E. coli Ribosome with Rearranged Peptidyl Transferase Centre. PDB entry: https://doi.org/10.2210/pdb9slg/pdb EMDB entry: https://www.ebi.ac.uk/emdb/EMD-55001
9SX2: 30S Doxycycline Bound E. coli Ribosome. PDB entry: https://doi.org/10.2210/pdb9sx2/pdb EMDB entry: https://www.ebi.ac.uk/emdb/EMD-55330
9T0Z: HPFcold Bound Hibernating C. burnetii 30S Ribosome. PDB entry: https://doi.org/10.2210/pdb9t0z/pdb EMDB entry: https://www.ebi.ac.uk/emdb/EMD-55416
9T61: Doxycycline Bound C. burnetii 30S Ribosome. PDB entry: https://doi.org/10.2210/pdb9t61/pdb EMDB entry: https://www.ebi.ac.uk/emdb/EMD-55601
9TZ5: Triple Stack Doxycycline Bound 50S Subunit of the Coxiella burnetii Ribosome. PDB entry: https://doi.org/10.2210/pdb9tz5/pdb EMDB entry: https://www.ebi.ac.uk/emdb/EMD-56459
9TZ9: Empty 50S Subunit of the Coxiella burnetii Ribosome. PDB entry: https://doi.org/10.2210/pdb9tz9/pdb EMDB entry: https://www.ebi.ac.uk/emdb/EMD-56461
9TZE: 70S Coxiella burnetii Ribosome with Doxycycline and HPFcold. PDB entry: https://doi.org/10.2210/pdb9tze/pdb EMDB entry: https://www.ebi.ac.uk/emdb/EMD-56466

Existing structures used for illustration or comparison are available at the Protein Data Bank as follows: stalled nascent chain E. coli ribosome (7ZP8), high resolution E. coli ribosome (7K00), VemP stalled E. coli ribosome (5NWY), YfiA bound T. thermophilus ribosome (4V8I), HPFshort bound T. thermophilus ribosome (4V8H), HPFlong bound L. lactis ribosome (5MYJ), E. coli CspA (1MJC), M. smegmatis ribosome large subunit (5XYM), Sarecycline bound C. acnes ribosome (8CVM), tetracenomycin-X bound E. coli ribosome (6Y69), erythromycin bound T. thermophilus ribosome (6XHX), P. gingivalis ribosome large subunit (9I5T), B. burgdorferi ribosome large subunit (8FN2).

Research involving human participants, their data, or biological material

Policy information about studies with human participants or human data. See also policy information about sex, gender (identity/presentation), and sexual orientation and race, ethnicity and racism.

Reporting on sex and gender	Not applicable
Reporting on race, ethnicity, or other socially relevant groupings	Not applicable
Population characteristics	Not applicable
Recruitment	Not applicable
Ethics oversight	Not applicable

Note that full information on the approval of the study protocol must also be provided in the manuscript.

Field-specific reporting

Please select the one below that is the best fit for your research. If you are not sure, read the appropriate sections before making your selection.

☒ Life sciences ☐ Behavioural & social sciences ☐ Ecological, evolutionary & environmental sciences

For a reference copy of the document with all sections, see nature.com/documents/nr-reporting-summary-flat.pdf

Life sciences study design

All studies must disclose on these points even when the disclosure is negative.

Sample size	22,195 micrographs were collected for C. burnetii, with the fewest ribosome particles contributing to a 3-D reconstruction being 10,803. 37,720 micrographs were collected for E. coli, the fewest ribosome particles contributing to a 3-D reconstruction being 215,222. Minimum inhibitory concentration experiments were conducted in triplicate (N=3).
Data exclusions	Ribosome particles were excluded based upon classification of signal as described in the Materials and Methods.

Replication	Minimum inhibitory concentration experiments were repeated on multiple different days and interpretations were made by multiple individuals, all repeats carried out confirmed the same result.
Randomization	Not applicable
Blinding	Not applicable

Reporting for specific materials, systems and methods

We require information from authors about some types of materials, experimental systems and methods used in many studies. Here, indicate whether each material, system or method listed is relevant to your study. If you are not sure if a list item applies to your research, read the appropriate section before selecting a response.

Materials & experimental systems

n/a	Involved in the study
☒	☐ Antibodies
☒	☐ Eukaryotic cell lines
☒	☐ Palaeontology and archaeology
☐	☒ Animals and other organisms
☒	☐ Clinical data
☒	☐ Dual use research of concern
☒	☐ Plants

Methods

n/a	Involved in the study
☒	☐ ChIP-seq
☒	☐ Flow cytometry
☒	☐ MRI-based neuroimaging

Animals and other research organisms

Policy information about studies involving animals; ARRIVE guidelines recommended for reporting animal research, and Sex and Gender in Research

Laboratory animals	Study did not involve laboratory animals
Wild animals	Study did not involve laboratory animals
Reporting on sex	Study did not involve laboratory animals
Field-collected samples	Study did not involve laboratory animals
Ethics oversight	No ethical approval or guidance was required for work on these bacteria.

Note that full information on the approval of the study protocol must also be provided in the manuscript.

Plants

Seed stocks	Not applicable
Novel plant genotypes	Not applicable
Authentication	Not applicable
