# Supplementary material for: Cryo-EM reveals multiple mechanisms of ribosome inhibition by doxycycline
Source: Nat Commun. 2026 Jun 1;17:7049. doi: 10.1038/s41467-026-73421-5 (PMC13392368; doi:10.1038/s41467-026-73421-5)
Supplement: Supplementary file 6 — Source Data [file 41467_2026_73421_MOESM6_ESM.zip › File 4.docx]

**Source data file 4: alignment of *Coxiella burnetii* 23S sequences.**

All 231 available *Coxiella burnetii* genomes (NCBI TaxID: 777) were downloaded from the NCBI database. The 23S rRNA sequences were extracted from these genomes by text searching for ‘product=”23S ribosomal RNA”’, ‘product=”large subunit ribosomal RNA”’ or ‘gene=”23S”’. This returned 259 23S rRNA sequences (in some, these were sequences split from the same genome where the rRNA had been assembled from shotgun sequencing). These sequences were pruned for identical sequences, returning 66 unique sequences. Five of these contained significant numbers of poor base calls and were excluded. The remaining 61 sequences were aligned together using the MUSCLE algorithm in Geneious v.2026.0.2. Bases within 4 Å of a doxycycline molecule in the 50S ribosome structure in complex with doxycycline are indicated with an arrow. In the structure, these are bases U1796, A2074, A2075, A2078, U2457, G2521, U2522, U2601, U2602, U2625, C2626, and C2627. These are indicated in the alignment with arrows, coloured red for those conserved to *Porphyromonas gingivalis*, whilst those that have U-C or C-U alterations (but are conserved in space) with *P. gingivalis* are indicated in blue. All contacting bases are conserved throughout extant *C. burnetii* genomes.

Consensus AGGGTTGCATGGTCAAGTGAATAAGCGCATATGGTGGATGCCTTGGCAGTAAGAGGCGATGAAGGACGTAGTAGCCTGCGATAAGCTTCGGGGAGCTGGCAAACAAGCGATGATCCGGAGATTTCCGAATGGGGAAACCCAACCTTTCGA 150

JAKFBC010000002.1:34496-38842(-) (45) ----------GGTCAAGTGAATAAGCGCATATGGTGGATGCCTTGGCAGTAAGAGGCGATGAAGGACGTAGTAGCCTGCGATAAGCTTCGGGGAGCTGGCAAACAAGCGATGATCCGGAGATTTCCGAATGGGGAAACCCAACCTTTCGA 140

NZ_AP019757.1:1739264-1743610(-) (31) ----------GGTCAAGTGAATAAGCGCATATGGTGGATGCCTTGGCAGTAAGAGGCGATGAAGGACGTAGTAGCCTGCGATAAGCTTCGGGGAGCTGGCAAACAAGCGATGATCCGGAGATTTCCGAATGGGGAAACCCAACCTTTCGA 140

JBKOHN010000001.1:81530-85875(+) (25) ----------GGTCAAGTGAATAAGCGCATATGGTGGATGCCTTGGCAGTAAGAGGCGATGAAGGACGTAGTAGCCTGCGATAAGCTTCGGGGAGCTGGCAAACAAGCGATGATCCGGAGATTTCCGAATGGGGAAACCCAACCTTTCGA 140

NC_011528.1:339307-343646(+) (14) ----------GGTCAAGTGAATAAGCGCATATGGTGGATGCCTTGGCAGTAAGAGGCGATGAAGGACGTAGTAGCCTGCGATAAGCTTCGGGGAGCTGGCAAACAAGCTATGATCCGGAGATTTCCGAATGGGGAAACCCAACCTTTCGA 140

CP018005.1:167566-171940(+) (14) AGGGTTGCATGGTCAAGTGAATAAGCGCATATGGTGGATGCCTTGGCAGTAAGAGGCGATGAAGGACGTAGTAGCCTGCGATAAGCTTCGGGGAGCTGGCAAACAAGCGATGATCCGGAGATTTCCGAATGGGGAAACCCAACCTTTCGA 150

NZ_CCAM010000005.1:71418-75764(+) (13) ----------GGTCAAGTGAATAAGCGCATATGGTGGATGCCTTGGCAGTAAGAGGCGATGAAGGACGTAGTAGCCTGCGATAAGCTTCGGGGAGCTGGCAAACAAGCGATGATCCGGAGATTTCCGAATGGGGAAACCCAACCTTTCGA 140

NZ_CCXO01000001.1:317226-321572(+) (11) ----------GGTCAAGTGAATAAGCGCATATGGTGGATGCCTTGGCAGTAAGAGGCGATGAAGGACGTAGTAGCCTGCGATAAGCTTCGGGGAGCTGGCAAACAAGCGATGATCCGGAGATTTCCGAATGGGGAAACCCAACCTTTCGA 140

NC_011527.1:1751498-1755844(-) (8) ----------GGTCAAGTGAATAAGCGCATATGGTGGATGCCTTGGCAGTAAGAGGCGATGAAGGACGTAGTAGCCTGCGATAAGCTTCGGGGAGCTGGCAAACAAGCGATGATCCGGAGATTTCCGAATGGGGAAACCCAACCTTTCGA 140

NZ_CP032542.1:88036-92382(-) (8) ----------GGTCAAGTGAATAAGCGCATATGGTGGATGCCTTGGCAGTAAGAGGCGATGAAGGACGTAGTAGCCTGCGATAAGCTTCGGGGAGCTGGCAAACAAGCGATGATCCGGAGATTTCCGAATGGGGAAACCCAACCTTTCGA 140

CP014551.1:161236-165610(+) (8) AGGGTTGCATGGTCAAGTGAATAAGCGCATATGGTGGATGCCTTGGCAGTAAGAGGCGATGAAGGACGTAGTAGCCTGCGATAAGCTTCGGGGAGCTGGCAAACAAGCGATGATCCGGAGATTTCCGAATGGGGAAACCCAACCTTTCGA 150

CP013667.1:88011-92385(-) (8) AGGGTTGCATGGTCAAGTGAATAAGCGCATATGGTGGATGCCTTGGCAGTAAGAGGCGATGAAGGACGTAGTAGCCTGCGATAAGCTTCGGGGAGCTGGCAAACAAGCGATGATCCGGAGATTTCCGAATGGGGAAACCCAACCTTTCGA 150

CP107247.1:164350-168689(+) (7) ----------GGTCAAGTGAATAAGCGCATATGGTGGATGCCTTGGCAGTAAGAGGCGATGAAGGACGTAGTAGCCTGCGATAAGCTTCGGGGAGCTGGCAAACAAGCGATGATCCGGAGATTTCCGAATGGGGAAACCCAACCTTTCGA 140

NZ_CP103435.1:167581-171920(+) (6) ----------GGTCAAGTGAATAAGCGCATATGGTGGATGCCTTGGCAGTAAGAGGCGATGAAGGACGTAGTAGCCTGCGATAAGCTTCGGGGAGCTGGCAAACAAGCGATGATCCGGAGATTTCCGAATGGGGAAACCCAACCTTTCGA 140

CP014565.1:1711747-1716121(-) (3) AGGGTTGCATGGTCAAGTGAATAAGCGCATATGGTGGATGCCTTGGCAGTAAGAGGCGATGAAGGACGTAGTAGCCTGCGATAAGCTTCGGGGAGCTGGCAAACAAGCGATGATCCGGAGATTTCCGAATGGGGAAACCCAACCTTTCGA 150

AAYJ01000007.1:0-1404(+) (3) ------------------------------------------------------------------------------------------------------------------------------------------------------

NZ_JAOXDR010000002.1:16672-21025(-) (3) ----------GGTCAAGTGAATAAGCGCATATGGTGGATGCCTTGGCAGTAAGAGGCGATGAAGGACGTAGTAGCCTGCGATAAGCTTCGGGGAGCTGGCAAACAAGCGATGATCCGGAGATTTCCGAATGGGGAAACCCAACCTTTCGA 140

NZ_CP103432.1:167699-172038(+) (3) ----------GGTCAAGTGAATAAGCGCATATGGTGGATGCCTTGGCAGTAAGAGGCGATGAAGGACGTAGTAGCCTGCGATAAGCTTCGGGGAGCTGGCAAACAAGCGATGATCCGGAGATTTCCGAATGGGGAAACCCAACCTTTCGA 140

NZ_CP103431.1:167697-172036(+) (3) ----------GGTCAAGTGAATAAGCGCATATGGTGGATGCCTTGGCAGTAAGAGGCGATGAAGGACGTAGTAGCCTGCGATAAGCTTCGGGGAGCTGGCAAACAAGCGATGATCCGGAGATTTCCGAATGGGGAAACCCAACCTTTCGA 140

NZ_CP103428.1:167591-171929(+) (2) ----------GGTCAAGTGAATAAGCGCATATGGTGGATGCCTTGGCAGTAAGAGGCGATGAAGGACGTAGTAGCCTGCGATAAGCTTCGGGGAGCTGGCAAACAAGCGATGATCCGGAGATTTCCGAATGGGGAAACCCAACCTTTCGA 140

NZ_JANTNR010000002.1:115350-119696(+) (2) ----------GGTCAAGTGAATAAGCGCATATGGTGGATGCCTTGGCAGTAAGAGGCGATGAAGGACGTAGTAGCCTGCGATAAGCTTCGGGGAGCTGGCAAACAAGCGATGATCCGGAGATTTCCGAATGGGGAAACCCAACCTTTCGA 140

JARBIR010004564.1:3782-8128(-) (2) ----------GGTCAAGTGAATAAGCGCATATGGTGGATGCCTTGGCAGTAAGAGGCGATGAAGGACGTAGTAGCCTGCGATAAGCTTCGGGGAGCTGGCAAACAAGCGATGATCCGGAGATTTCCGAATGGGGAAACCCAACCTTTCGA 140

NC_009727.1:1888349-1892695(-) (2) ----------GGTCAAGTGAATAAGCGCATATGGTGGATGCCTTGGCAGTAAGAGGCGATGAAGGACGTAGTAGCCTGCGATAAGCTTCGGGGAGCTGGCAAACAAGCGATGATCCGGAGATTTCCGAATGGGGAAACCCAACCTTTCGA 140

AAYJ01000139.1:0-660(+) (2) ------------------------------------------------------------------------------------------------------------------------------------------------------

CP032542.1:88018-92392(-) (2) AGGGTTGCATGGTCAAGTGAATAAGCGCATATGGTGGATGCCTTGGCAGTAAGAGGCGATGAAGGACGTAGTAGCCTGCGATAAGCTTCGGGGAGCTGGCAAACAAGCGATGATCCGGAGATTTCCGAATGGGGAAACCCAACCTTTCGA 150

CP014559.1:232744-237117(+) (2) AGGGTTGCATGGTCAAGTGAATAAGCGCATATGGTGGATGCCTTGGCAGTAAGAGGCGATGAAGGACGTAGTAGCCTGCGATAAGCTTCGGGGAGCTGGCAAACAAGCGATGATCCGGAGATTTCCGAATGGGGAAACCCAACCTTTCGA 150

CP007555.1:337408-341752(+) ------------TCAAGTGAATAAGCGCATATGGTGGATGCCTTGGCAGTAAGAGGCGATGAAGGACGTAGTAGCCTGCGATAAGCTTCGGGGAGCTGGCAAACAAGCGATGATCCGGAGATTTCCGAATGGGGAAACCCAACCTTTCGA 138

NZ_CP014354.1:76710-81178(-) ----------GGTCAAGTGAATAAGCGCATATGGTGGATGCCTTGGCAGTAAGAGGCGATGAAGGACGTAGTAGCCTGCGATAAGCTTCGGGGAGCTGGCAAACAAGCGATGATCCGGAGATTTCCGAATGGGGAAACCCAACCTTTCGA 140

CP000890.1:243129-244433(+) ------------------------------------------------------------------------------------------------------------------------------------------------------

NZ_CP115461.1:167576-171922(+) ----------GGTCAAGTGAATAAGCGCATATGGTGGATGCCTTGGCAGTAAGAGGCGATGAAGGACGTAGTAGCCTGCGATAAGCTTCGGGGAGCTGGCAAACAAGCGATGATCCGGAGATTTCCGAATGGGGAAACCCAACCTTTCGA 140

AP019759.1:167578-171919(+) ------------TCAAGTGAATAAGCGCATATGGTGGATGCCTTGGCAGTAAGAGGCGATGAAGGACGTAGTAGCCTGCGATAAGCTTCGGGGAGCTGGCAAACAAGCGATGATCCGGAGATTTCCGAATGGGGAAACCCAACCTTTCGA 138

AP019757.1:1739267-1743608(-) ------------TCAAGTGAATAAGCGCATATGGTGGATGCCTTGGCAGTAAGAGGCGATGAAGGACGTAGTAGCCTGCGATAAGCTTCGGGGAGCTGGCAAACAAGCGATGATCCGGAGATTTCCGAATGGGGAAACCCAACCTTTCGA 138

NOLN01000021.1:90861-95235(-) AGGGTTGCATGGTCAAGTGAATAAGCGCATATGGTGGATGCCTTGGCAGTAAGAGGCGATGAAGGACGTAGTAGCCTGCGATAAGCTTCGGGGAGCTGGCAAACAAGCGATGATCCGGAGATTTCCGAATGGGGAAACCCAACCTTTCGA 150

NOLM01000022.1:91202-95576(-) AGGGTTGCATGGTCAAGTGAATAAGCGCATATGGTGGATGCCTTGGCAGTAAGAGGCGATGAAGGACGTAGTAGCCTGCGATAAGCTTCGGGGAGCTGGCAAACAAGCGATGATCCGGAGATTTCCGAATGGGGAAACCCAACCTTTCGA 150

PDLP01000011.1:14431-18805(+) AGGGTTGCATGGTCAAGTGAATAAGCGCATATGGTGGATGCCTTGGCAGTAAGAGGCGATGAAGGACGTAGTAGCCTGCGATAAGCTTCGGGGAGCTGGCAAACAAGCGATGATCCGGAGATTTCCGAATGGGGAAACCCAACCTTTCGA 150

CP014563.1:522940-527314(+) AGGGTTGCATGGTCAAGTGAATAAGCGCATATGGTGGATGCCTTGGCAGTAAGAGGCGATGAAGGACGTAGTAGCCTGCGATAAGCTTCGGGGAGCTGGCAAACAAGCGATGATCCGGAGATTTCCGAATGGGGAAACCCAACCTTTCGA 150

CP000890.1:244922-245905(+) ------------------------------------------------------------------------------------------------------------------------------------------------------

NZ_NOLR01000002.1:131843-136189(+) ----------GGTCAAGTGAATAAGCGCATATGGTGGATGCCTTGGCAGTAAGAGGCGATGAAGGACGTAGTAGCCTGCGATAAGCTTCGGGGAGCTGGCAAACAAGCGATGATCCGGAGATTTCCGAATGGGGAAACCCAACCTTTCGA 140

NZ_JAOXDP010000002.1:15794-20139(-) ----------GGTCAAGTGAATAAGCGCATATGGTGGATGCCTTGGCAGTAAGAGGCGATGAAGGACGTAGTAGCCTGCGATAAGCTTCGGGGAGCTGGCAAACAAGCGATGATCCGGAGATTTCCGAATGGGGAAACCCAACCTTTCGA 140

NZ_PPFR01000008.1:34378-38724(-) ----------GGTCAAGTGAATAAGCGCATATGGTGGATGCCTTGGCAGTAAGAGGCGATGAAGGACGTAGTAGCCTGCGATAAGCTTCGGGGAGCTGGCAAACAAGCGATGATCCGGAGATTTCCGAATGGGGAAACCCAACCTTTCGA 140

NZ_PPFQ01000003.1:131093-135446(+) ----------GGTCAAGTGAATAAGCGCATATGGTGGATGCCTTGGCAGTAAGAGGCGATGAAGGACGTAGTAGCCTGCGATAAGCTTCGGGGAGCTGGCAAACAAGCGATGATCCGGAGATTTCCGAATGGGGAAACCCAACCTTTCGA 140

NZ_LK937696.1:167613-171953(+) ----------GGTCAAGTGAATAAGCGCATATGGTGGATGCCTTGGCAGTAAGAGGCGATGAAGGACGTAGTAGCCTGCGATAAGCTTCGGGGAGCTGGCAAACAAGCGATGATCCGGAGATTTCCGAATGGGGAAACCCAACCTTTCGA 140

NZ_NOVI01000003.1:105965-110310(+) ----------GGTCAAGTGAATAAGCGCATATGGTGGATGCCTTGGCAGTAAGAGGCGATGAAGGACGTAGTAGCCTGCGATAAGCTTCGGGGAGCTGGCAAACAAGCGATGATCCGGAGATTTCCGAATGGGGAAACCCAACCTTTCGA 140

NZ_CP103426.1:167642-171981(+) ----------GGTCAAGTGAATAAGCGCATATGGTGGATGCCTTGGCAGTAAGAGGCGATGAAGGACGTAGTAGCCTGCGATAAGCTTCGGGGAGCTGGCAAACAAGCGATGATCCGGAGATTTCCGAATGGGGAAACCCAACCTTTCGA 140

NZ_JAOXDN010000007.1:36985-41324(-) ----------GGTCAAGTGAATAAGCGCATATGGTGGATGCCTTGGCAGTAAGAGGCGATGAAGGACGTAGTAGCCTGCGATAAGCTTCGGGGAGCTGGCAAACAAGCGATGATCCGGAGATTTCCGAATGGGGAAACCCAACCTTTCGA 140

NZ_JPVV01000013.1:13888-18051(+) ----------GGTCAAGTGAATAAGCGCATATGGTGGATGCCTTGGCAGTAAGAGGCGATGAAGGACGTAGTAGCCTGCGATAAGCTTCGGGGAGCTGGCAAACAAGCGATGATCCGGAGATTTCCGAATGGGGAAACCCAACCTTTCGA 140

NZ_CP007555.1:337406-341752(+) ----------GGTCAAGTGAATAAGCGCATATGGTGGATGCCTTGGCAGTAAGAGGCGATGAAGGACGTAGTAGCCTGCGATAAGCTTCGGGGAGCTGGCAAACAAGCGATGATCCGGAGATTTCCGAATGGGGAAACCCAACCTTTCGA 140

NZ_JASNNV010000010.1:39284-43631(-) ----------GGTCAAGTGAATAAGCGCATATGGTGGATGCCTTGGCAGTAAGAGGCGATGAAGGACGTAGTAGCCTGCGATAAGCTTCGGGGAGCTGGCAAACAAGCGATGATCCGGAGATTTCCGAATGGGGAAACCCAACCTTTCGA 140

NZ_JAOXFC010000012.1:14231-18577(+) ----------GGTCAAGTGAATAAGCGCATATGGTGGATGCCTTGGCAGTAAGAGGCGATGAAGGACGTAGTAGCCTGCGATAAGCTTCGGGGAGCTGGCAAACAAGCGATGATCCGGAGATTTCCGAATGGGGAAACCCAACCTTTCGA 140

NZ_CP103430.1:167658-171997(+) ----------GGTCAAGTGAATAAGCGCATATGGTGGATGCCTTGGCAGTAAGAGGCGATGAAGGACGTAGTAGCCTGCGATAAGCTTCGGGGAGCTGGCAAACAAGCGATGATCCGGAGATTTCCGAATGGGGAAACCCAACCTTTCGA 140

NZ_PDLP01000011.1:14441-18787(+) ----------GGTCAAGTGAATAAGCGCATATGGTGGATGCCTTGGCAGTAAGAGGCGATGAAGGACGTAGTAGCCTGCGATAAGCTTCGGGGAGCTGGCAAACAAGCGATGATCCGGAGATTTCCGAATGGGGAAACCCAACCTTTCGA 140

NZ_NOLM01000022.1:91220-95566(-) ----------GGTCAAGTGAATAAGCGCATATGGTGGATGCCTTGGCAGTAAGAGGCGATGAAGGACGTAGTAGCCTGCGATAAGCTTCGGGGAGCTGGCAAACAAGCGATGATCCGGAGATTTCCGAATGGGGAAACCCAACCTTTCGA 140

JBJCIS010000056.1:0-2780(-) ----------GGTCAAGTGAATAAGCGCATATGGTGGATGCCTTGGCAGTAAGAGGCGATGAAGGACGTAGTAGCCTGCGATAAGCTTCGGGGAGCTGGCAAACAAGCGATGATCCGGAGATTTCCGAATGGGGAAACCTAACCTTTCGA 140

NZ_CDBG01000001.1:179744-184090(+) ----------GGTCAAGTGAATAAGCGCATATGGTGGATGCCTTGGCAGTAAGAGGCGATGAAGGACGTAGTAGCCTGCGATAAGCTTCGGGGAGCTGGCAAACAAGCGATGATCCGGAGATTTCCGAATGGGGAAACCCAACCTTTCGA 140

NZ_CP103434.1:167622-171961(+) ----------GGTCAAGTGAATAAGGGCATATGGTGGATGCCTTGGCAGTAAGAGGCGATGAAGGACGTAGTAGCCTGCGATAAGCTTCGGGGAGCTGGCAAACAAGCGATGATCCGGAGATTTCCGAATGGGGAAACCCAACCTTTCGA 140

CP014354.1:76692-80603(-) ------------------------------------------------------------------------------------------------------------------------------------------------------

NOLR01000002.1:131833-136207(+) AGGGTTGCATGGTCAAGTGAATAAGCGCATATGGTGGATGCCTTGGCAGTAAGAGGCGATGAAGGACGTAGTAGCCTGCGATAAGCTTCGGGGAGCTGGCAAACAAGCGATGATCCGGAGATTTCCGAATGGGGAAACCCAACCTTTCGA 150

PPFR01000008.1:34360-38734(-) AGGGTTGCATGGTCAAGTGAATAAGCGCATATGGTGGATGCCTTGGCAGTAAGAGGCGATGAAGGACGTAGTAGCCTGCGATAAGCTTCGGGGAGCTGGCAAACAAGCGATGATCCGGAGATTTCCGAATGGGGAAACCCAACCTTTCGA 150

PPFQ01000003.1:131083-135464(+) AGGGTTGCATGGTCAAGTGAATAAGCGCATATGGTGGATGCCTTGGCAGTAAGAGGCGATGAAGGACGTAGTAGCCTGCGATAAGCTTCGGGGAGCTGGCAAACAAGCGATGATCCGGAGATTTCCGAATGGGGAAACCCAACCTTTCGA 150

NZ_AKYP01000181.1:1647-6020(-) AGGGTTGCATGGTCAAGTGAATAAGCGCATATGGTGGATGCCTTGGCAGTAAGAGGCGATGAAGGACGTAGTAGCCTGCGATAAGCTTCGGGGAGCTGGCAAACAAGCGATGATCCGGAGATTTCCGAATGGGGAAACCCAACCTTTCGA 150

NOVI01000003.1:105955-110328(+) AGGGTTGCATGGTCAAGTGAATAAGCGCATATGGTGGATGCCTTGGCAGTAAGAGGCGATGAAGGACGTAGTAGCCTGCGATAAGCTTCGGGGAGCTGGCAAACAAGCGATGATCCGGAGATTTCCGAATGGGGAAACCCAACCTTTCGA 150

CP018150.1:339294-343661(+) AGGGTTGCATGGTCAAGTGAATAAGCGCATATGGTGGATGCCTTGGCAGTAAGAGGCGATGAAGGACGTAGTAGCCTGCGATAAGCTTCGGGGAGCTGGCAAACAAGCTATGATCCGGAGATTTCCGAATGGGGAAACCCAACCTTTCGA 150

Consensus GGTTATCGTATACTGAATTCATAGGTATACGAGGCGAACCTGGGGAACTGAAACATCTAAGTACCCAGAGGAAAAGAAATCAACCGAGATTCCGTCAGTAGCGGCGAGCGAAAGCGGAACAGCCCAGTTACTAAATCATTATTTGTTCTA 300

JAKFBC010000002.1:34496-38842(-) (45) GGTTATCGTATACTGAATTCATAGGTATACGAGGCGAACCTGGGGAACTGAAACATCTAAGTACCCAGAGGAAAAGAAATCAACCGAGATTCCGTCAGTAGCGGCGAGCGAAAGCGGAACAGCCCAGTTACTAAATCATTATTTGTTCTA 290

NZ_AP019757.1:1739264-1743610(-) (31) GGTTATCGTATACTGAATTCATAGGTATACGAGGCGAACCTGGGGAACTGAAACATCTAAGTACCCAGAGGAAAAGAAATCAACCGAGATTCCGTCAGTAGCGGCGAGCGAAAGCGGAACAGCCCAGTTACTAAATCATTATTTGTTCTA 290

JBKOHN010000001.1:81530-85875(+) (25) GGTTATCGTATACTGAATTCATAGGTATACGAGGCGAACCTGGGGAACTGAAACATCTAAGTACCCAGAGGAAAAGAAATCAACCGAGATTCCGTCAGTAGCGGCGAGCGAAAGCGGAACAGCCCAGTTACTAAATCATTATTTGTTCTA 290

NC_011528.1:339307-343646(+) (14) GGTTATCGTATACTGAATTCATAGGTATACGAGGCGAACCTGGGGAACTGAAACATCTAAGTACCCAGAGGAAAAGAAATCAACCGAGATTCCGTCAGTAGCGGCGAGCGAAAGCGGAACAGCCCAGTTACTAAATCATTATTTGTTCTA 290

CP018005.1:167566-171940(+) (14) GGTTATCGTATACTGAATTCATAGGTATACGAGGCGAACCTGGGGAACTGAAACATCTAAGTACCCAGAGGAAAAGAAATCAACCGAGATTCCGTCAGTAGCGGCGAGCGAAAGCGGAACAGCCCAGTTACTAAATCATTATTTGTTCTA 300

NZ_CCAM010000005.1:71418-75764(+) (13) GGTTATCGTATACTGAATTCATAGGTATACGAGGCGAACCTGGGGAACTGAAACATCTAAGTACCCAGAGGAAAAGAAATCAACCGAGATTCCGTCAGTAGCGGCGAGCGAAAGCGGAACAGCCCAGTTACTAAATCATTATTTGTTCTA 290

NZ_CCXO01000001.1:317226-321572(+) (11) GGTTATCGTATACTGAATTCATAGGTATACGAGGCGAACCTGGGGAACTGAAACATCTAAGTACCCAGAGGAAAAGAAATCAACCGAGATTCCGTCAGTAGCGGCGAGCGAAAGCGGAACAGCCCAGTTACTAAATCATTATTTGTTCTA 290

NC_011527.1:1751498-1755844(-) (8) GGTTATCGAATACTGAATTCATAGGTATACGAGGCGAACCTGGGGAACTGAAACATCTAAGTACCCAGAGGAAAAGAAATCAACCGAGATTCCGTCAGTAGCGGCGAGCGAAAGCGGAATAGCCCAGTTACTAAATCATTATTTGTTCTA 290

NZ_CP032542.1:88036-92382(-) (8) GGTTATCGTATACTGAATTCATAGGTATACGAGGCGAACCTGGGGAACTGAAACATCTAAGTACCCAGAGGAAAAGAAATCAACCGAGATTCCGTCAGTAGCGGCGAGCGAAAGCGGAACAGCCCAGTTACTAAATCATTATTTGTTCTA 290

CP014551.1:161236-165610(+) (8) GGTTATCGTATACTGAATTCATAGGTATACGAGGCGAACCTGGGGAACTGAAACATCTAAGTACCCAGAGGAAAAGAAATCAACCGAGATTCCGTCAGTAGCGGCGAGCGAAAGCGGAACAGCCCAGTTACTAAATCATTATTTGTTCTA 300

CP013667.1:88011-92385(-) (8) GGTTATCGTATACTGAATTCATAGGTATACGAGGCGAACCTGGGGAACTGAAACATCTAAGTACCCAGAGGAAAAGAAATCAACCGAGATTCCGTCAGTAGCGGCGAGCGAAAGCGGAACAGCCCAGTTACTAAATCATTATTTGTTCTA 300

CP107247.1:164350-168689(+) (7) GGTTATCGTATACTGAATTCATAGGTATACGAGGCGAACCTGGGGAACTGAAACATCTAAGTACCCAGAGGAAAAGAAATCAACCGAGATTCCGTCAGTAGCGGCGAGCGAAAGCGGAACAGCCCAGTTACTAAATCATTATTTGTTCTA 290

NZ_CP103435.1:167581-171920(+) (6) GGTTATCGTATACTGAATTCATAGGTATACGAGGCGAACCTGGGGAACTGAAACATCTAAGTACCCAGAGGAAAAGAAATCAACCGAGATTCCGTCAGTAGCGGCGAGCGAAAGCGGAACAGCCCAGTTACTAAATCATTATTTGTTCTA 290

CP014565.1:1711747-1716121(-) (3) GGTTATCGAATACTGAATTCATAGGTATACGAGGCGAACCTGGGGAACTGAAACATCTAAGTACCCAGAGGAAAAGAAATCAACCGAGATTCCGTCAGTAGCGGCGAGCGAAAGCGGAATAGCCCAGTTACTAAATCATTATTTGTTCTA 300

AAYJ01000007.1:0-1404(+) (3) ------------------------------------------------------------------------------------------------------------------------------------------------------

NZ_JAOXDR010000002.1:16672-21025(-) (3) GGTTATCGTATACTGAATTCATAGGTATACGAGGCGAACCTGGGGAACTGAAACATCTAAGTACCCAGAGGAAAAGAAATCAACCGAGATTCCGTCAGTAGCGGCGAGCGAAAGCGGAACAGCCCAGTTACTAAATCATTATTTGTTCTA 290

NZ_CP103432.1:167699-172038(+) (3) GGTTATCGTATACTGAATTCATAGGTATACGAGGCGAACCTGGGGAACTGAAACATCTAAGTACCCAGAGGAAAAGAAATCAACCGAGATTCCGTCAGTAGCGGCGAGCGAAAGCGGAACAGCCCAGTTACTAAATCATTATTTGTTCTA 290

NZ_CP103431.1:167697-172036(+) (3) GGTTATCGTATACTGAATTCATAGGTATACGAGGCGAACCTGGGGAACTGAAACATCTAAGTACCCAGAGGAAAAGAAATCAACCGAGATTCCGTCAGTAGCGGCGAGCGAAAGCGGAACAGCCCAGTTACTAAATCATTATTTGTTCTA 290

NZ_CP103428.1:167591-171929(+) (2) GGTTATCGTATACTGAATTCATAGGTATACGAGGCGAACCTGGGGAACTGAAACATCTAAGTACCCAGAGGAAAAGAAATCAACCGAGATTCCGTCAGTAGCGGCGAGCGAAAGCGGAACAGCCCAGTTACTAAATCATTATTTGTTCTA 290

NZ_JANTNR010000002.1:115350-119696(+) (2) GGTTATCGTATACTGAATTCATAGGTATACGAGGCGAACCTGGGGAACTGAAACATCTAAGTACCCAGAGGAAAAGAAATCAACCGAGATTCCGTCAGTAGCGGCGAGCGAAAGCGGAACAGCCCAGTTACTAAATCATTATTTGTTCTA 290

JARBIR010004564.1:3782-8128(-) (2) GGTTATCGTATACTGAATTCATAGGTATACGAGGCGAACCTGGGGAACTGAAACATCTAAGTACCCAGAGGAAAAGAAATCAACCGAGATTCCGTCAGTAGCGGCGAGCGAAAGCGGAACAGCCCAGTTACTAAATCATTATTTGTTCTA 290

NC_009727.1:1888349-1892695(-) (2) GGTTATCGTATACTGAATTCATAGGTATACGAGGCGAACCTGGGGAACTGAAACATCTAAGTACCCAGAGGAAAAGAAATCAACCGAGATTCCGTCAGTAGCGGCGAGCGAAAGCGGAACAGCCCAGTTACTAAATCATTATTTGTTCTA 290

AAYJ01000139.1:0-660(+) (2) ------------------------------------------------------------------------------------------------------------------------------------------------------

CP032542.1:88018-92392(-) (2) GGTTATCGTATACTGAATTCATAGGTATACGAGGCGAACCTGGGGAACTGAAACATCTAAGTACCCAGAGGAAAAGAAATCAACCGAGATTCCGTCAGTAGCGGCGAGCGAAAGCGGAACAGCCCAGTTACTAAATCATTATTTGTTCTA 300

CP014559.1:232744-237117(+) (2) GGTTATCGTATACTGAATTCATAGGTATACGAGGCGAACCTGGGGAACTGAAACATCTAAGTACCCAGAGGAAAAGAAATCAACCGAGATTCCGTCAGTAGCGGCGAGCGAAAGCGGAACAGCCCAGTTACTAAATCATTATTTGTTCTA 300

CP007555.1:337408-341752(+) GGTTATCGTATACTGAATTCATAGGTATACGAGGCGAACCTGGGGAACTGAAACATCTAAGTACCCAGAGGAAAAGAAATCAACCGAGATTCCGTCAGTAGCGGCGAGCGAAAGCGGAACAGCCCAGTTACTAAATCATTATTTGTTCTA 288

NZ_CP014354.1:76710-81178(-) GGTTATCGTATACTGAATTCATAGGTATACGAGGCGAACCTGGGGAACTGAAACATCTAAGTACCCAGAGGAAAAGAAATCAACCGAGATTCCGTCAGTAGCGGCGAGCGAAAGCGGAACAGCCCAGTTACTAAATCATTATTTGTTCTA 290

CP000890.1:243129-244433(+) ------------------------------------------------------------------------------------------------------------------------------------------------------

NZ_CP115461.1:167576-171922(+) GGTTATCGTATACTGAATTCATAGGTATACGAGGCGAACCTGGGGAACTGAAACATCTAAGTACCCAGAGGAAAAGAAATCAACCGAGATTCCGTCAGTAGCGGCGAGCGAAAGCGGAACAGCCCAGTTACTAAATCATTATTTGTTCTA 290

AP019759.1:167578-171919(+) GGTTATCGTATACTGAATTCATAGGTATACGAGGCGAACCTGGGGAACTGAAACATCTAAGTACCCAGAGGAAAAGAAATCAACCGAGATTCCGTCAGTAGCGGCGAGCGAAAGCGGAACAGCCCAGTTACTAAATCATTATTTGTTCTA 288

AP019757.1:1739267-1743608(-) GGTTATCGTATACTGAATTCATAGGTATACGAGGCGAACCTGGGGAACTGAAACATCTAAGTACCCAGAGGAAAAGAAATCAACCGAGATTCCGTCAGTAGCGGCGAGCGAAAGCGGAACAGCCCAGTTACTAAATCATTATTTGTTCTA 288

NOLN01000021.1:90861-95235(-) GGTTATCGTATACTGAATTCATAGGTATACGAGGCGAACCTGGGGAACTGAAACATCTAAGTACCCAGAGGAAAAGAAATCAACCGAGATTCCGTCAGTAGCGGCGAGCGAAAGCGGAACAGCCCAGTTACTAAATCATTATTTGTTCTA 300

NOLM01000022.1:91202-95576(-) GGTTATCGTATACTGAATTCATAGGTATACGAGGCGAACCTGGGGAACTGAAACATCTAAGTACCCAGAGGAAAAGAAATCAACCGAGATTCCGTCAGTAGCGGCGAGCGAAAGCGGAACAGCCCAGTTACTAAATCATTATTTGTTCTA 300

PDLP01000011.1:14431-18805(+) GGTTATCGTATACTGAATTCATAGGTATACGAGGCGAACCTGGGGAACTGAAACATCTAAGTACCCAGAGGAAAAGAAATCAACCGAGATTCCGTCAGTAGCGGCGAGCGAAAGCGGAACAGCCCAGTTACTAAATCATTATTTGTTCTA 300

CP014563.1:522940-527314(+) GGTTATCGTATACTGAATTCATAGGTATACGAGGCGAACCTGGGGAACTGAAACATCTAAGTACCCAGAGGAAAAGAAATCAACCGAGATTCCGTCAGTAGCGGCGAGCGAAAGCGGAACAGCCCAGTTACTAAATCATTATTTGTTCTA 300

CP000890.1:244922-245905(+) ------------------------------------------------------------------------------------------------------------------------------------------------------

NZ_NOLR01000002.1:131843-136189(+) GGTTATCGTATACTGAATTCATAGGTATACGAGGCGAACCTGGGGAACTGAAACATCTAAGTACCCAGAGGAAAAGAAATCAACCGAGATTCCGTCAGTAGCGGCGAGCGAAAGCGGAACAGCCCAGTTACTAAATCATTATTTGTTCTA 290

NZ_JAOXDP010000002.1:15794-20139(-) GGTTATCGTATACTGAATTCATAGGTATACGAGGCGAACCTGGGGAACTGAAACATCTAAGTACCCAGAGGAAAAGAAATCAACCGAGATTCCGTCAGTAGCGGCGAGCGAAAGCGGAACAGCCCAGTTACTAAATCATTATTTGTTCTA 290

NZ_PPFR01000008.1:34378-38724(-) GGTTATCGTATACTGAATTCATAGGTATACGAGGCGAACCTGGGGAACTGAAACATCTAAGTACCCAGAGGAAAAGAAATCAACCGAGATTCCGTCAGTAGCGGCGAGCGAAAGCGGAACAGCCCAGTTACTAAATCATTATTTGTTCTA 290

NZ_PPFQ01000003.1:131093-135446(+) GGTTATCGTATACTGAATTCATAGGTATACGAGGCGAACCTGGGGAACTGAAACATCTAAGTACCCAGAGGAAAAGAAATCAACCGAGATTCCGTCAGTAGCGGCGAGCGAAAGCGGAACAGCCCAGTTACTAAATCATTATTTGTTCTA 290

NZ_LK937696.1:167613-171953(+) GGTTATCGTATACTGAATTCATAGGTATACGAGGCGAACCTGGGGAACTGAAACATCTAAGTACCCAGAGGAAAAGAAATCAACCGAGATTCCGTCAGTAGCGGCGAGCGAAAGCGGAACAGCCCAGTTACTAAATCATTATTTGTTCTA 290

NZ_NOVI01000003.1:105965-110310(+) GGTTATCGTATACTGAATTCATAGGTATACGAGGCGAACCTGGGGAACTGAAACATCTAAGTACCCAGAGGAAAAGAAATCAACCGAGATTCCGTCAGTAGCGGCGAGCGAAAGCGGAACAGCCCAGTTACTAAATCATTATTTGTTCTA 290

NZ_CP103426.1:167642-171981(+) GGTTATCGTATACTGAATTCATAGGTATACGAGGCGAACCTGGGGAACTGAAACATCTAAGTACCCAGAGGAAAAGAAATCAACCGAGATTCCGTCAGTAGCGGCGAGCGAAAGCGGAACAGCCCAGTTACTAAATCATTATTTGTTCTA 290

NZ_JAOXDN010000007.1:36985-41324(-) GGTTATCGTATACTGAATTCATAGGTATACGAGGCGAACCTGGGGAACTGAAACATCTAAGTACCCAGAGGAAAAGAAATCAACCGAGATTCCGTCAGTAGCGGCGAGCGAAAGCGGAACAGCCCAGTTACTAAATCATTATTTGTTCTA 290

NZ_JPVV01000013.1:13888-18051(+) GGTTATCGTATACTGAATTCATAGGTATACGAGGCGAACCTGGGGAACTGAAACATCTAAGTACCCAGAGGAAAAGAAATCAACCGAGATTCCGTCAGTAGCGGCGAGCGAAAGCGGAACAGCCCAGTTACTAAATCATTATTTGTTCTA 290

NZ_CP007555.1:337406-341752(+) GGTTATCGTATACTGAATTCATAGGTATACGAGGCGAACCTGGGGAACTGAAACATCTAAGTACCCAGAGGAAAAGAAATCAACCGAGATTCCGTCAGTAGCGGCGAGCGAAAGCGGAACAGCCCAGTTACTAAATCATTATTTGTTCTA 290

NZ_JASNNV010000010.1:39284-43631(-) GGTTATCGTATACTGAATTCATAGGTATACGAGGCGAACCTGGGGAACTGAAACATCTAAGTACCCAGAGGAAAAGAAATCAACCGAGATTCCGTCAGTAGCGGCGAGCGAAAGCGGAACAGCCCAGTTACTAAATCATTATTTGTTCTA 290

NZ_JAOXFC010000012.1:14231-18577(+) GGTTATCGTATACTGAATTCATAGGTATACGAGGCGAACCTGGGGAACTGAAACATCTAAGTACCCAGAGGAAAAGAAATCAACCGAGATTCCGTCAGTAGCGGCGAGCGAAAGCGGAACAGCCCAGTTACTAAATCATTATTTGTTCTA 290

NZ_CP103430.1:167658-171997(+) GGTTATCGTATACTGAATTCATAGGTATACGAGGCGAACCTGGGGAACTGAAACATCTAAGTACCCAGAGGAAAAGAAATCAACCGAGATTCCGTCAGTAGCGGCGAGCGAAAGCGGAACAGCCCAGTTACTAAATCATTATTTGTTCTA 290

NZ_PDLP01000011.1:14441-18787(+) GGTTATCGTATACTGAATTCATAGGTATACGAGGCGAACCTGGGGAACTGAAACATCTAAGTACCCAGAGGAAAAGAAATCAACCGAGATTCCGTCAGTAGCGGCGAGCGAAAGCGGAACAGCCCAGTTACTAAATCATTATTTGTTCTA 290

NZ_NOLM01000022.1:91220-95566(-) GGTTATCGTATACTGAATTCATAGGTATACGAGGCGAACCTGGGGAACTGAAACATCTAAGTACCCAGAGGAAAAGAAATCAACCGAGATTCCGTCAGTAGCGGCGAGCGAAAGCGGAACAGCCCAGTTACTAAATCATTATTTGTTCTA 290

JBJCIS010000056.1:0-2780(-) GGTTATCGTATACTGAATTCATAGGTATACGAGGCGAACCTGGGGAACTGAAACATCTAAGTACCCAGAGGAAAAGAAATCAACCGAGATTCCGTCAGTAGCGGCGAGCGAAAGCGGAACAGCCCAGTTACTAAATCATTATTTGTTCTA 290

NZ_CDBG01000001.1:179744-184090(+) GGTTATCGTATACTGAATTCATAGGTATACGAGGCGAACCTGGGGAACTGAAACATCTAAGTACCCAGAGGAAAAGAAATCAACCGAGATTCCGTCAGTAGCGGCGAGCGAAAGCGGAACAGCCCAGTTACTAAATCATTATTTGTTCTA 290

NZ_CP103434.1:167622-171961(+) GGTTATCGTATACTGAATTCATAGGTATACGAGGCGAACCTGGGGAACTGAAACATCTAAGTACCCAGAGGAAAAGAAATCAACCGAGATTCCGTCAGTAGCGGCGAGCGAAAGCGGAACAGCCCAGTTACTAAATCATTATTTGTTCTA 290

CP014354.1:76692-80603(-) ------------------------------------------------------------------------------------------------------------------------------------------------------

NOLR01000002.1:131833-136207(+) GGTTATCGTATACTGAATTCATAGGTATACGAGGCGAACCTGGGGAACTGAAACATCTAAGTACCCAGAGGAAAAGAAATCAACCGAGATTCCGTCAGTAGCGGCGAGCGAAAGCGGAACAGCCCAGTTACTAAATCATTATTTGTTCTA 300

PPFR01000008.1:34360-38734(-) GGTTATCGTATACTGAATTCATAGGTATACGAGGCGAACCTGGGGAACTGAAACATCTAAGTACCCAGAGGAAAAGAAATCAACCGAGATTCCGTCAGTAGCGGCGAGCGAAAGCGGAACAGCCCAGTTACTAAATCATTATTTGTTCTA 300

PPFQ01000003.1:131083-135464(+) GGTTATCGTATACTGAATTCATAGGTATACGAGGCGAACCTGGGGAACTGAAACATCTAAGTACCCAGAGGAAAAGAAATCAACCGAGATTCCGTCAGTAGCGGCGAGCGAAAGCGGAACAGCCCAGTTACTAAATCATTATTTGTTCTA 300

NZ_AKYP01000181.1:1647-6020(-) GGTTATCGTATACTGAATTCATAGGTATACGAGGCGAACCTGGGGAACTGAAACATCTAAGTACCCAGAGGAAAAGAAATCAACCGAGATTCCGTCAGTAGCGGCGAGCGAAAGCGGAACAGCCCAGTTACTAAATCATTATTTGTTCTA 300

NOVI01000003.1:105955-110328(+) GGTTATCGTATACTGAATTCATAGGTATACGAGGCGAACCTGGGGAACTGAAACATCTAAGTACCCAGAGGAAAAGAAATCAACCGAGATTCCGTCAGTAGCGGCGAGCGAAAGCGGAACAGCCCAGTTACTAAATCATTATTTGTTCTA 300

CP018150.1:339294-343661(+) GGTTATCGTATACTGAATTCATAGGTATACGAGGCGAACCTGGGGAACTGAAACATCTAAGTACCCAGAGGAAAAGAAATCAACCGAGATTCCGTCAGTAGCGGCGAGCGAAAGCGGAACAGCCCAGTTACTAAATCATTATTTGTTCTA 300

Consensus GCAGAATGTTCTGGAAAGTTCAGCCATAGCGGGTGATAGCCCCGTACGCGAAAGAGTAAATAATGTGGGTAACGATGAGTAGGTCGGGACACGTGGTATCTTGACTGAACATGGGGGGACCATCCTCCAAGGCTAAATACTCCTTACTGA 450

JAKFBC010000002.1:34496-38842(-) (45) GCAGAATGTTCTGGAAAGTTCAGCCATAGCGGGTGATAGCCCCGTACGCGAAAGAGTAAATAATGTGGGTAACGATGAGTAGGTCGGGACACGTGGTATCTTGACTGAACATGGGGGGACCATCCTCCAAGGCTAAATACTCCTTACTGA 440

NZ_AP019757.1:1739264-1743610(-) (31) GCAGAATGTTCTGGAAAGTTCAGCCATAGCGGGTGATAGCCCCGTACGCGAAAGAGTAAATAATGTGGGTAACGATGAGTAGGTCGGGACACGTGGTATCTTGACTGAACATGGGGGGACCATCCTCCAAGGCTAAATACTCCTTACTGA 440

JBKOHN010000001.1:81530-85875(+) (25) GCAGAATGTTCTGGAAAGTTCAGCCATAGCGGGTGATAGCCCCGTACGCGAAAGAGTAAATAATGTGGGTAACGATGAGTAGGTCGGGACACGTGGTATCTTGACTGAACATGGGGGGACCATCCTCCAAGGCTAAATACTCCTTACTGA 440

NC_011528.1:339307-343646(+) (14) GCAGAATGTTCTGGAAAGTTCAGCCATAGCGGGTGATAGCCCCGTACGCGAAAGAGTAAATAATGTGGGTAACGATGAGTAGGTCGGGACACGTGGTATCTTGACTGAACATGGGGGGACCATCCTCCAAGGCTAAATACTCCTTACTGA 440

CP018005.1:167566-171940(+) (14) GCAGAATGTTCTGGAAAGTTCAGCCATAGCGGGTGATAGCCCCGTACGCGAAAGAGTAAATAATGTGGGTAACGATGAGTAGGTCGGGACACGTGGTATCTTGACTGAACATGGGGGGACCATCCTCCAAGGCTAAATACTCCTTACTGA 450

NZ_CCAM010000005.1:71418-75764(+) (13) GCAGAATGTTCTGGAAAGTTCAGCCATAGCGGGTGATAGCCCCGTACGCGAAAGAGTAAATAATGTGGGTAACGATGAGTAGGTCGGGACACGTGGTATCTTGACTGAACATGGGGGGACCATCCTCCAAGGCTAAATACTCCTTACTGA 440

NZ_CCXO01000001.1:317226-321572(+) (11) GCAGAATGTTCTGGAAAGTTCAGCCATAGCGGGTGATAGCCCCGTACGCGAAAGAGTAAATAATGTGGGTAACGATGAGTAGGTCGGGACACGTGGTATCTTGACTGAACATGGGGGGACCATCCTCCAAGGCTAAATACTCCTTACTGA 440

NC_011527.1:1751498-1755844(-) (8) GCAGAATGTTCTGGAAAGTTCAGCCATAGCGGGTGATAGCCCCGTACGCGAAAGAGTAAATAATGTGGGTAACGATGAGTAGGTCGGGACACGTGGTATCTTGACTGAACATGGGGGGACCATCCTCCAAGGCTAAATACTCCTTACTGA 440

NZ_CP032542.1:88036-92382(-) (8) GCAGAATGTTCTGGAAAGTTCAGCCATAGCGGGTGATAGCCCCGTACGCGAAAGAGTAAATAATGTGGGTAACGATGAGTAGGTCGGGACACGTGGTATCTTGACTGAACATGGGGGGACCATCCTCCAAGGCTAAATACTCCTTACTGA 440

CP014551.1:161236-165610(+) (8) GCAGAATGTTCTGGAAAGTTCAGCCATAGCGGGTGATAGCCCCGTACGCGAAAGAGTAAATAATGTGGGTAACGATGAGTAGGTCGGGACACGTGGTATCTTGACTGAACATGGGGGGACCATCCTCCAAGGCTAAATACTCCTTACTGA 450

CP013667.1:88011-92385(-) (8) GCAGAATGTTCTGGAAAGTTCAGCCATAGCGGGTGATAGCCCCGTACGCGAAAGAGTAAATAATGTGGGTAACGATGAGTAGGTCGGGACACGTGGTATCTTGACTGAACATGGGGGGACCATCCTCCAAGGCTAAATACTCCTTACTGA 450

CP107247.1:164350-168689(+) (7) GCAGAATGTTCTGGAAAGTTCAGCCATAGCGGGTGATAGCCCCGTACGCGAAAGAGTAAATAATGTGGGTAACGATGAGTAGGTCGGGACACGTGGTATCTTGACTGAACATGGGGGGACCATCCTCCAAGGCTAAATACTCCTTACTGA 440

NZ_CP103435.1:167581-171920(+) (6) GCAGAATGTTCTGGAAAGTTCAGCCATAGCGGGTGATAGCCCCGTACGCGAAAGAGTAAATAATGTGGGTAACGATGAGTAGGTCGGGACACGTGGTATCTTGACTGAACATGGGGGGACCATCCTCCAAGGCTAAATACTCCTTACTGA 440

CP014565.1:1711747-1716121(-) (3) GCAGAATGTTCTGGAAAGTTCAGCCATAGCGGGTGATAGCCCCGTACGCGAAAGAGTAAATAATGTGGGTAACGATGAGTAGGTCGGGACACGTGGTATCTTGACTGAACATGGGGGGACCATCCTCCAAGGCTAAATACTCCTTACTGA 450

AAYJ01000007.1:0-1404(+) (3) ------------------------------------------------------------------------------------------------------------------------------------------------------

NZ_JAOXDR010000002.1:16672-21025(-) (3) GCAGAATGTTCTGGAAAGTTCAGCCATAGCGGGTGATAGCCCCGTACGCGAAAGAGTAAATAATGTGGGTAACGATGAGTAGGTCGGGACACGTGGTATCTTGACTGAACATGGGGGGACCATCCTCCAAGGCTAAATACTCCTTACTGA 440

NZ_CP103432.1:167699-172038(+) (3) GCAGAATGTTCTGGAAAGTTCAGCCATAGCGGGTGATAGCCCCGTACGCGAAAGAGTAAATAATGTGGGTAACGATGAGTAGGTCGGGACACGTGGTATCTTGACTGAACATGGGGGGACCATCCTCCAAGGCTAAATACTCCTTACTGA 440

NZ_CP103431.1:167697-172036(+) (3) GCAGAATGTTCTGGAAAGTTCAGCCATAGCGGGTGATAGCCCCGTACGCGAAAGAGTAAATAATGTGGGTAACGATGAGTAGGTCGGGACACGTGGTATCTTGACTGAACATGGGGGGACCATCCTCCAAGGCTAAATACTCCTTACTGA 440

NZ_CP103428.1:167591-171929(+) (2) GCAGAATGTTCTGGAAAGTTCAGCCATAGCGGGTGATAGCCCCGTACGCGAAAGAGTAAATAATGTGGGTAACGATGAGTAGGTCGGGACACGTGGTATCTTGACTGAACATGGGGGGACCATCCTCCAAGGCTAAATACTCCTTACTGA 440

NZ_JANTNR010000002.1:115350-119696(+) (2) GCAGAATGTTCTGGAAAGTTCAGCCATAGCGGGTGATAGCCCCGTACGCGAAAGAGTAAATAATGTGGGTAACGATGAGTAGGTCGGGACACGTGGTATCTTGACTGAACATGGGGGGACCATCCTCCAAGGCTAAATACTCCTTACTGA 440

JARBIR010004564.1:3782-8128(-) (2) GCAGAATGTTCTGGAAAGTTCAGCCATAGCGGGTGATAGCCCCGTACGCGAAAGAGTAAATAATGTGGGTAACGATGAGTAGGTCGGGACACGTGGTATCTTGACTGAACATGGGGGGACCATCCTCCAAGGCTAAATACTCCTTACTGA 440

NC_009727.1:1888349-1892695(-) (2) GCAGAATGTTCTGGAAAGTTCAGCCATAGCGGGTGATAGCCCCGTACGCGAAAGAGTAAATAATGTGGGTAACGATGAGTAGGTCGGGACACGTGGTATCTTGACTGAACATGGGGGGACCATCCTCCAAGGCTAAATACTCCTTACTGA 440

AAYJ01000139.1:0-660(+) (2) ------------------------------------------------------------------------------------------------------------------------------------------------------

CP032542.1:88018-92392(-) (2) GCAGAATGTTCTGGAAAGTTCAGCCATAGCGGGTGATAGCCCCGTACGCGAAAGAGTAAATAATGTGGGTAACGATGAGTAGGTCGGGACACGTGGTATCTTGACTGAACATGGGGGGACCATCCTCCAAGGCTAAATACTCCTTACTGA 450

CP014559.1:232744-237117(+) (2) GCAGAATGTTCTGGAAAGTTCAGCCATAGCGGGTGATAGCCCCGTACGCGAAAGAGTAAATAATGTGGGTAACGATGAGTAGGTCGGGACACGTGGTATCTTGACTGAACATGGGGGGACCATCCTCCAAGGCTAAATACTCCTTACTGA 450

CP007555.1:337408-341752(+) GCAGAATGTTCTGGAAAGTTCAGCCATAGCGGGTGATAGCCCCGTACGCGAAAGAGTAAATAATGTGGGTAACGATGAGTAGGTCGGGACACGTGGTATCTTGACTGAACATGGGGGGACCATCCTCCAAGGCTAAATACTCCTTACTGA 438

NZ_CP014354.1:76710-81178(-) GCAGAATGTTCTGGAAAGTTCAGCCATAGCGGGTGATAGCCCCGTACGCGAAAGAGTAAATAATGTGGGTAACGATGAGTAGGTCGGGACACGTGGTATCTTGACTGAACATGGGGGGACCATCCTCCAAGGCTAAATACTCCTTACTGA 440

CP000890.1:243129-244433(+) ------------------------------------------------------------------------------------------------------------------------------------------------------

NZ_CP115461.1:167576-171922(+) GCAGAATGTTCTGGAAAGTTCAGCCATAGCGGGTGATAGCCCCGTACGCGAAAGAGTAAATAATGTGGGTAACGATGAGTAGGTCGGGACACGTGGTATCTTGACTGAAGAAGGGGGGACCATCCTCCAAGGCTAAATACTCCTGACTGA 440

AP019759.1:167578-171919(+) GCAGAATGTTCTGGAAAGTTCAGCCATAGCGGGTGATAGCCCCGTACGCGAAAGAGTAAATAATGTGGGTAACGATGAGTAGGTCGGGACACGTGGTATCTTGACTGAACATGGGGGGACCATCCTCCAAGGCTAAATACTCCTTACTGA 438

AP019757.1:1739267-1743608(-) GCAGAATGTTCTGGAAAGTTCAGCCATAGCGGGTGATAGCCCCGTACGCGAAAGAGTAAATAATGTGGGTAACGATGAGTAGGTCGGGACACGTGGTATCTTGACTGAACATGGGGGGACCATCCTCCAAGGCTAAATACTCCTTACTGA 438

NOLN01000021.1:90861-95235(-) GCAGAATGTTCTGGAAAGTTCAGCCATAGCGGGTGATAGCCCCGTACGCGAAAGAGTAAATAATGTGGGTAACGATGAGTAGGTCGGGACACGTGGTATCTTGACTGAACATGGGGGGACCATCCTCCAAGGCTAAATACTCCTTACTGA 450

NOLM01000022.1:91202-95576(-) GCAGAATGTTCTGGAAAGTTCAGCCATAGCGGGTGATAGCCCCGTACGCGAAAGAGTAAATAATGTGGGTAACGATGAGTAGGTCGGGACACGTGGTATCTTGACTGAACATGGGGGGACCATCCTCCAAGGCTAAATACTCCTTACTGA 450

PDLP01000011.1:14431-18805(+) GCAGAATGTTCTGGAAAGTTCAGCCATAGCGGGTGATAGCCCCGTACGCGAAAGAGTAAATAATGTGGGTAACGATGAGTAGGTCGGGACACGTGGTATCTTGACTGAACATGGGGGGACCATCCTCCAAGGCTAAATACTCCTTACTGA 450

CP014563.1:522940-527314(+) GCAGAATGTTCTGGAAAGTTCAGCCATAGCGGGTGATAGCCCCGTACGCGAAAGAGTAAATAATGTGGGTAACGATGAGTAGGTCGGGACACGTGGTATCTTGACTGAACATGGGGGGACCATCCTCCAAGGCTAAATACTCCTTACTGA 450

CP000890.1:244922-245905(+) ------------------------------------------------------------------------------------------------------------------------------------------------------

NZ_NOLR01000002.1:131843-136189(+) GCAGAATGTTCTGGAAAGTTCAGCCATAGCGGGTGATAGCCCCGTACGCGAAAGAGTAAATAATGTGGGTAACGATGAGTAGGTCGGGACACGTGGTATCTTGACTGAACATGGGGGGACCATCCTCCAAGGCTAAATACTCCTTACTGA 440

NZ_JAOXDP010000002.1:15794-20139(-) GCAGAATGTTCTGGAAAGTTCAGCCATAGCGGGTGATAGCCCCGTACGCGAAAGAGTAAATAATGTGGGTAACGATGAGTAGGTCGGGACACGTGGTATCTTGACTGAACATGGGGGGACCATCCTCCAAGGCTAAATACTCCTTACTGA 440

NZ_PPFR01000008.1:34378-38724(-) GCAGAATGTTCTGGAAAGTTCAGCCATAGCGGGTGATAGCCCCGTACGCGAAAGAGTAAATAATGTGGGTAACGATGAGTAGGTCGGGACACGTGGTATCTTGACTGAACATGGGGGGACCATCCTCCAAGGCTAAATACTCCTTACTGA 440

NZ_PPFQ01000003.1:131093-135446(+) GCAGAATGTTCTGGAAAGTTCAGCCATAGCGGGTGATAGCCCCGTACGCGAAAGAGTAAATAATGTGGGTAACGATGAGTAGGTCGGGACACGTGGTATCTTGACTGAACATGGGGGGACCATCCTCCAAGGCTAAATACTCCTTACTGA 440

NZ_LK937696.1:167613-171953(+) GCAGAATGTTCTGGAAAGTTCAGCCATAGCGGGTGATAGCCCCGTACGCGAAAGAGTAAATAATGTGGGTAACGATGAGTAGGTCGGGACACGTGGTATCTTGACTGAACATGGGGGGACCATCCTCCAAGGCTAAATACTCCTTACTGA 440

NZ_NOVI01000003.1:105965-110310(+) GCAGAATGTTCTGGAAAGTTCAGCCATAGCGGGTGATAGCCCCGTACGCGAAAGAGTAAATAATGTGGGTAACGATGAGTAGGTCGGGACACGTGGTATCTTGACTGAACATGGGGGGACCATCCTCCAAGGCTAAATACTCCTTACTGA 440

NZ_CP103426.1:167642-171981(+) GCAGAATGTTCTGGAAAGTTCAGCCATAGCGGGTGATAGCCCCGTACGCGAAAGAGTAAATAATGTGGGTAACGATGAGTAGGTCGGGACACGTGGTATCTTGACTGAACATGGGGGGACCATCCTCCAAGGCTAAATACTCCTTACTGA 440

NZ_JAOXDN010000007.1:36985-41324(-) GCAGAATGTTCTGGAAAGTTCAGCCATAGCGGGTGATAGCCCCGTACGCGAAAGAGTAAATAATGTGGGTAACGATGAGTAGGTCGGGACACGTGGTATCTTGACTGAACATGGGGGGACCATCCTCCAAGGCTAAATACTCCTTACTGA 440

NZ_JPVV01000013.1:13888-18051(+) GCAGAATGTTCTGGAAAGTTCAGCCATAGCGGGTGATAGCCCCGTACGCGAAAGAGTAAATAATGTGGGTAACGATGAGTAGGTCGGGACACGTGGTATCTTGACTGAACATGGGGGGACCATCCTCCAAGGCTAAATACTCCTTACTGA 440

NZ_CP007555.1:337406-341752(+) GCAGAATGTTCTGGAAAGTTCAGCCATAGCGGGTGATAGCCCCGTACGCGAAAGAGTAAATAATGTGGGTAACGATGAGTAGGTCGGGACACGTGGTATCTTGACTGAACATGGGGGGACCATCCTCCAAGGCTAAATACTCCTTACTGA 440

NZ_JASNNV010000010.1:39284-43631(-) GCAGAATGTTCTGGAAAGTTCAGCCATAGCGGGTGATAGCCCCGTACGCGAAAGAGTAAATAATGTGGGTAACGATGAGTAGGTCGGGACACGTGGTATCTTGACTGAACATGGGGGGACCATCCTCCAAGGCTAAATACTCCTTACTGA 440

NZ_JAOXFC010000012.1:14231-18577(+) GCAGAATGTTCTGGAAAGTTCAGCCATAGCGGGTGATAGCCCCGTACGCGAAAGAGTAAATAATGTGGGTAACGATGAGTAGGTCGGGACACGTGGTATCTTGACTGAACATGGGGGGACCATCCTCCAAGGCTAAATACTCCTTACTGA 440

NZ_CP103430.1:167658-171997(+) GCAGAATGTTCTGGAAAGTTCAGCCATAGCGGGTGATAGCCCCGTACGCGAAAGAGTAAATAATGTGGGTAACGATGAGTAGGTCGGGACACGTGGTATCTTGACTGAACATGGGGGGACCATCCTCCAAGGCTAAATACTCCTTACTGA 440

NZ_PDLP01000011.1:14441-18787(+) GCAGAATGTTCTGGAAAGTTCAGCCATAGCGGGTGATAGCCCCGTACGCGAAAGAGTAAATAATGTGGGTAACGATGAGTAGGTCGGGACACGTGGTATCTTGACTGAACATGGGGGGACCATCCTCCAAGGCTAAATACTCCTTACTGA 440

NZ_NOLM01000022.1:91220-95566(-) GCAGAATGTTCTGGAAAGTTCAGCCATAGCGGGTGATAGCCCCGTACGCGAAAGAGTAAATAATGTGGGTAACGATGAGTAGGTCGGGACACGTGGTATCTTGACTGAACATGGGGGGACCATCCTCCAAGGCTAAATACTCCTTACTGA 440

JBJCIS010000056.1:0-2780(-) GCAGAATGTTCTGGAAAGTTCAGCCATAGCGGGTGATAGCCCCGTACGCGAAAGAGTAAATAATGTGGGTAACGATGAGTAGGTCGGGACACGTGGTATCTTGACTGAACATGGGGGGACCATCCTCCAAGGCTAAATACTCCTTACTGA 440

NZ_CDBG01000001.1:179744-184090(+) GCAGAATGTTCTGGAAAGTTCAGCCATAGCGGGTGATAGCCCCGTACGCGAAAGAGTAAATAATGTGGGTAACGATGAGTAGGTCGGGACACGTGGTATCTTGACTGAACATGGGGGGACCATCCTCCAAGGCTAAATACTCCTTACTGA 440

NZ_CP103434.1:167622-171961(+) GCAGAATGTTCTGGAAAGTTCAGCCATAGCGGGTGATAGCCCCGTACGCGAAAGAGTAAATAATGTGGGTAACGATGAGTAGGTCGGGACACGTGGTATCTTGACTGAACATGGGGGGACCATCCTCCAAGGCTAAATACTCCTTACTGA 440

CP014354.1:76692-80603(-) ------------------------------------------------------------------------------------------------------------------------------------------------------

NOLR01000002.1:131833-136207(+) GCAGAATGTTCTGGAAAGTTCAGCCATAGCGGGTGATAGCCCCGTACGCGAAAGAGTAAATAATGTGGGTAACGATGAGTAGGTCGGGACACGTGGTATCTTGACTGAACATGGGGGGACCATCCTCCAAGGCTAAATACTCCTTACTGA 450

PPFR01000008.1:34360-38734(-) GCAGAATGTTCTGGAAAGTTCAGCCATAGCGGGTGATAGCCCCGTACGCGAAAGAGTAAATAATGTGGGTAACGATGAGTAGGTCGGGACACGTGGTATCTTGACTGAACATGGGGGGACCATCCTCCAAGGCTAAATACTCCTTACTGA 450

PPFQ01000003.1:131083-135464(+) GCAGAATGTTCTGGAAAGTTCAGCCATAGCGGGTGATAGCCCCGTACGCGAAAGAGTAAATAATGTGGGTAACGATGAGTAGGTCGGGACACGTGGTATCTTGACTGAACATGGGGGGACCATCCTCCAAGGCTAAATACTCCTTACTGA 450

NZ_AKYP01000181.1:1647-6020(-) GCAGAATGTTCTGGAAAGTTCAGCCATAGCGGGTGATAGCCCCGTACGCGAAAGAGTAAATAATGTGGGTAACGATGAGTAGGTCGGGACACGTGGTATCTTGACTGAACATGGGGGGACCATCCTCCAAGGCTAAATACTCCTTACTGA 450

NOVI01000003.1:105955-110328(+) GCAGAATGTTCTGGAAAGTTCAGCCATAGCGGGTGATAGCCCCGTACGCGAAAGAGTAAATAATGTGGGTAACGATGAGTAGGTCGGGACACGTGGTATCTTGACTGAACATGGGGGGACCATCCTCCAAGGCTAAATACTCCTTACTGA 450

CP018150.1:339294-343661(+) GCAGAATGTTCTGGAAAGTTCAGCCATAGCGGGTGATAGCCCCGTACGCGAAAGAGTAAATAATGTGGGTAACGATGAGTAGGTCGGGACACGTGGTATCTTGACTGAACATGGGGGGACCATCCTCCAAGGCTAAATACTCCTTACTGA 450

Consensus CCGATAGCGAACCAGTACCGTGAGGGAAAG------GTGAAAAGAACCCCGGCGAGGGG-AGTGAAATAGAACCTGAAACCGTATGCGTACAAGCAGTAGGAGCATTTCTTCGGAAATGTGACTGCGTACCTTTTGTATAATGGGTCAGC 593

JAKFBC010000002.1:34496-38842(-) (45) CCGATAGCGAACCAGTACCGTGAGGGAAAG------GTGAAAAGAACCCCGGCGAGGGG-AGTGAAATAGAACCTGAAACCGTATGCGTACAAGCAGTAGGAGCATTTCTTCGGAAATGTGACTGCGTACCTTTTGTATAATGGGTCAGC 583

NZ_AP019757.1:1739264-1743610(-) (31) CCGATAGCGAACCAGTACCGTGAGGGAAAG------GTGAAAAGAACCCCGGCGAGGGG-AGTGAAATAGAACCTGAAACCGTATGCGTACAAGCAGTAGGAGCATTTCTTCGGAAATGTGACTGCGTACCTTTTGTATAATGGGTCAGC 583

JBKOHN010000001.1:81530-85875(+) (25) CCGATAGCGAACCAGTACCGTGAGGGAAAG------GTGAAAAGAACCCCGGCGAGGGG-AGTGAAATAGAACCTGAAACCGTATGCGTACAAGCAGTAGGAGCATTTCTTCGGAAATGTGACTGCGTACCTTTTGTATAATGGGTCAGC 583

NC_011528.1:339307-343646(+) (14) CCGATAGCGAACCAGTACCGTGAGGGAAAG------GTGAAAAGAACCCCGGCGAGGGG-AGTGAAATAGAACCTGAAACCGTATGCGTACAAGCAGTAGGAGCATTTCTTCGGAAGTGTGACTGCGTACCTTTTGTATAATGGGTCAGC 583

CP018005.1:167566-171940(+) (14) CCGATAGCGAACCAGTACCGTGAGGGAAAG------GTGAAAAGAACCCCGGCGAGGGG-AGTGAAATAGAACCTGAAACCGTATGCGTACAAGCAGTAGGAGCATTTCTTCGGAAATGTGACTGCGTACCTTTTGTATAATGGGTCAGC 593

NZ_CCAM010000005.1:71418-75764(+) (13) CCGATAGCGAACCAGTACCGTGAGGGAAAG------GTGAAAAGAACCCCGGCGAGGGG-AGTGAAATAGAACCTGAAACCGTATGCGTACAAGCAGTAGGAGCATTTCTTCGGAAATGTGACTGCGTACCTTTTGTATAATGGGTCAGC 583

NZ_CCXO01000001.1:317226-321572(+) (11) CCGATAGCGAACCAGTACCGTGAGGGAAAG------GTGAAAAGAACCCCGGCGAGGGG-AGTGAAATAGAACCTGAAACCGTATGCGTACAAGCAGTAGGAGCATTTCTTCGGAAGTGTGACTGCGTACCTTTTGTATAATGGGTCAGC 583

NC_011527.1:1751498-1755844(-) (8) CCGATAGCGAACCAGTACCGTGAGGGAAAG------GTGAAAAGAACCCCGGCGAGGGG-AGTGAAATAGAACCTGAAACCGTATGCGTACAAGCAGTAGGAGCATTTCTTCGGAAATGTGACTGCGTACCTTTTGTATAATGGGTCAGC 583

NZ_CP032542.1:88036-92382(-) (8) CCGATAGCGAACCAGTACCGTGAGGGAAAG------GTGAAAAGAACCCCGGCGAGGGG-AGTGAAATAGAACCTGAAACCGTATGCGTACAAGCAGTAGGAGCATTTCTTCGGAAATGTGACTGCGTACCTTTTGTATAATGGGTCAGC 583

CP014551.1:161236-165610(+) (8) CCGATAGCGAACCAGTACCGTGAGGGAAAG------GTGAAAAGAACCCCGGCGAGGGG-AGTGAAATAGAACCTGAAACCGTATGCGTACAAGCAGTAGGAGCATTTCTTCGGAAATGTGACTGCGTACCTTTTGTATAATGGGTCAGC 593

CP013667.1:88011-92385(-) (8) CCGATAGCGAACCAGTACCGTGAGGGAAAG------GTGAAAAGAACCCCGGCGAGGGG-AGTGAAATAGAACCTGAAACCGTATGCGTACAAGCAGTAGGAGCATTTCTTCGGAAATGTGACTGCGTACCTTTTGTATAATGGGTCAGC 593

CP107247.1:164350-168689(+) (7) CCGATAGCGAACCAGTACCGTGAGGGAAAG------GTGAAAAGAACCCCGGCGAGGGG-AGTGAAATAGAACCTGAAACCGTATGCGTACAAGCAGTAGGAGCATTTCTTCGGAAGTGTGACTGCGTACCTTTTGTATAATGGGTCAGC 583

NZ_CP103435.1:167581-171920(+) (6) CCGATAGCGAACCAGTACCGTGAGGGAAAG------GTGAAAAGAACCCCGGCGAGGGG-AGTGAAATAGAACCTGAAACCGTATGCGTACAAGCAGTAGGAGCATTTCTTCGGAAATGTGACTGCGTACCTTTTGTATAATGGGTCAGC 583

CP014565.1:1711747-1716121(-) (3) CCGATAGCGAACCAGTACCGTGAGGGAAAG------GTGAAAAGAACCCCGGCGAGGGG-AGTGAAATAGAACCTGAAACCGTATGCGTACAAGCAGTAGGAGCATTTCTTCGGAAATGTGACTGCGTACCTTTTGTATAATGGGTCAGC 593

AAYJ01000007.1:0-1404(+) (3) ------------------------------------------------------------------------------------------------------------------------------------------------------

NZ_JAOXDR010000002.1:16672-21025(-) (3) CCGATAGCGAACCAGTACCGTGAGGGAAAG------GTGAAAAGAACCCCGGCGAGGGG-AGTGAAATAGAACCTGAAACCGTATGCGTACAAGCAGTAGGAGCATTTCTTCGGAAATGTGACTGCGTACCTTTTGTATAATGGGTCAGC 583

NZ_CP103432.1:167699-172038(+) (3) CCGATAGCGAACCAGTACCGTGAGGGAAAG------GTGAAAAGAACCCCGGCGAGGGG-AGTGAAATAGAACCTGAAACCGTATGCGTACAAGCAGTAGGAGCATTTCTTCGGAAATGTGACTGCGTACCTTTTGTATAATGGGTCAGC 583

NZ_CP103431.1:167697-172036(+) (3) CCGATAGCGAACCAGTACCGTGAGGGAAAG------GTGAAAAGAACCCCGGCGAGGGG-AGTGAAATAGAACCTGAAACCGTATGCGTACAAGCAGTAGGAGCATTTCTTCGGAAGTGTGACTGCGTACCTTTTGTATAATGGGTCAGC 583

NZ_CP103428.1:167591-171929(+) (2) CCGATAGCGAACCAGTACCGTGAGGGAAAG------GTGAAAAGAACCCCGGCGAGGGG-AGTGAAATAGAACCTGAAACCGTATGCGTACAAGCAGTAGGAGCATTTCTTCGGAAATGTGACTGCGTACCTTTTGTATAATGGGTCAGC 583

NZ_JANTNR010000002.1:115350-119696(+) (2) CCGATAGCGAACCAGTACCGTGAGGGAAAG------GTGAAAAGAACCCCGGCGAGGGG-AGTGAAATAGAACCTGAAACCGTATGCGTACAAGCAGTAGGAGCATTTCTTCGGAAATGTGACTGCGTACCTTTTGTATAATGGGTCAGC 583

JARBIR010004564.1:3782-8128(-) (2) CCGATAGCGAACCAGTACCGTGAGGGAAAG------GTGAAAAGAACCCCGGCGAGGGG-AGTGAAATAGAACCTGAAACCGTATGCGTACAAGCAGTAGGAGCATTTCTTCGGAAATGTGACTGCGTACCTTTTGTATAATGGGTCAGC 583

NC_009727.1:1888349-1892695(-) (2) CCGATAGCGAACCAGTACCGTGAGGGAAAG------GTGAAAAGAACCCCGGCGAGGGG-AGTGAAATAGAACCTGAAACCGTATGCGTACAAGCAGTAGGAGCATTTCTTCGGAAGTGTGACTGCGTACCTTTTGTATAATGGGTCAGC 583

AAYJ01000139.1:0-660(+) (2) ------------------------------------------------------------------------------------------------------------------------------------------------------

CP032542.1:88018-92392(-) (2) CCGATAGCGAACCAGTACCGTGAGGGAAAG------GTGAAAAGAACCCCGGCGAGGGG-AGTGAAATAGAACCTGAAACCGTATGCGTACAAGCAGTAGGAGCATTTCTTCGGAAATGTGACTGCGTACCTTTTGTATAATGGGTCAGC 593

CP014559.1:232744-237117(+) (2) CCGATAGCGAACCAGTACCGTGAGGGAAAG------GTGAAAAGAACCCCGGCGAGGGG-AGTGAAATAGAACCTGAAACCGTATGCGTACAAGCAGTAGGAGCATTTCTTCGGAAATGTGACTGCGTACCTTTTGTATAATGGGTCAGC 593

CP007555.1:337408-341752(+) CCGATAGCGAACCAGTACCGTGAGGGAAAG------GTGAAAAGAACCCCGGCGAGGGG-AGTGAAATAGAACCTGAAACCGTATGCGTACAAGCAGTAGGAGCATTTCTTCGGAAGTGTGACTGCGTACCTTTTGTATAATGGGTCAGC 581

NZ_CP014354.1:76710-81178(-) CCGATAGCGAACCAGTACCGTGAGGGAAAGGGGAAGGTGAAAAGAACCCCGGCGAGGGG-AGTGAAATAGAACCTGAAACCGTATGCGTACAAGCAGTAGGAGCATTTCTTCGGAAATGTGACTGCGTACCTTTTGTATAATGGGTCAGC 589

CP000890.1:243129-244433(+) ------------------------------------------------------------------------------------------------------------------------------------------------------

NZ_CP115461.1:167576-171922(+) CCGATAGTGAACCAGTACCGTGAGGGAAAG------GCGAAAAGAACCCCGGCGAGGGG-AGTGAAATAGAACCTGAAACCGTATGCGTACAAGCAGTAGGAGCATTTCTTCGGAAATGTGACTGCGTACCTTTTGTATAATGGGTCAGC 583

AP019759.1:167578-171919(+) CCGATAGCGAACCAGTACCGTGAGGGAAAG------GTGAAAAGAACCCCGGCGAGGGG-AGTGAAATAGAACCTGAAACCGTATGCGTACAAGCAGTAGGAGCATTTCTTCGGAAATGTGACTGCGTACCTTTTGTATAATGGGTCAGC 581

AP019757.1:1739267-1743608(-) CCGATAGCGAACCAGTACCGTGAGGGAAAG------GTGAAAAGAACCCCGGCGAGGGG-AGTGAAATAGAACCTGAAACCGTATGCGTACAAGCAGTAGGAGCATTTCTTCGGAAATGTGACTGCGTACCTTTTGTATAATGGGTCAGC 581

NOLN01000021.1:90861-95235(-) CCGATAGCGAACCAGTACCGTGAGGGAAAG------GTGAAAAGAACCCCGGCGAGGGG-AGTGAAATAGAACCTGAAACCGTATGCGTACAAGCAGTAGGAGCATTTCTTCGGAAGTGTGACTGCGTACCTTTTGTATAATGGGTCAGC 593

NOLM01000022.1:91202-95576(-) CCGATAGCGAACCAGTACCGTGAGGGAAAG------GTGAAAAGAACCCCGGCGAGGGG-AGTGAAATAGAACCTGAAACCGTATGCGTACAAGCAGTAGGAGCATTTCTTCGGAAGTGTGACTGCGTACCTTTTGTATAATGGGTCAGC 593

PDLP01000011.1:14431-18805(+) CCGATAGCGAACCAGTACCGTGAGGGAAAG------GTGAAAAGAACCCCGGCGAGGGG-AGTGAAATAGAACCTGAAACCGTATGCGTACAAGCAGTAGGAGCATTTCTTCGGAAGTGTGACTGCGTACCTTTTGTATAATGGGTCAGC 593

CP014563.1:522940-527314(+) CCGATAGCGAACCAGTACCGTGAGGGAAAG------GTGAAAAGAACCCCGGCGAGGGG-AGTGAAATAGAACCTGAAACCGTATGCGTACAAGCAGTAGGAGCATTTCTTCGGAAGTGTGACTGCGTACCTTTTGTATAATGGGTCAGC 593

CP000890.1:244922-245905(+) ------------------------------------------------------------------------------------------------------------------------------------------------------

NZ_NOLR01000002.1:131843-136189(+) CCGATAGCGAACCAGTACCGTGAGGGAAAG------GTGAAAAGAACCCCGGCGAGGGG-AGTGAAATAGAACCTGAAACCGTATGCGTACAAGCAGTAGGAGCATTTCTTCGGAAATGTGACTGCGTACCTTTTGTATAATGGGTCAGC 583

NZ_JAOXDP010000002.1:15794-20139(-) CCGATAGCGAACCAGTACCGTGAGGGAAAG------GTGAAAAGAACCCCGGCGAGGGG-AGTGAAATAGAACCTGAAACCGTATGCGTACAAGCAGTAGGAGCATTTCTTCGGAAATGTGACTGCGTACCTTTTGTATAATGGGTCAGC 583

NZ_PPFR01000008.1:34378-38724(-) CCGATAGCGAACCAGTACCGTGAGGGAAAG------GTGAAAAGAACCCCGGCGAGGGG-AGTGAAATAGAACCTGAAACCGTATGCGTACAAGCAGTAGGAGCATTTCTTCGGAAATGTGACTGCGTACCTTTTGTATAATGGGTCAGC 583

NZ_PPFQ01000003.1:131093-135446(+) CCGATAGCGAACCAGTACCGTGAGGGAAAG------GTGAAAAGAACCCCGGCGAGGGG-AGTGAAATAGAACCTGAAACCGTATGCGTACAAGCAGTAGGAGCATTTCTTCGGAAATGTGACTGCGTACCTTTTGTATAATGGGTCAGC 583

NZ_LK937696.1:167613-171953(+) CCGATAGCGAACCAGTACCGTGAGGGAAAG------GTGAAAAGAACCCCGGCGAGGGG-AGTGAAATAGAACCTGAAACCGTATGCGTACAAGCAGTAGGAGCATTTCTTCGGAAATGTGACTGCGTACCTTTTGTATAATGGGTCAGC 583

NZ_NOVI01000003.1:105965-110310(+) CCGATAGCGAACCAGTACCGTGAGGGAAAG------GTGAAAAGAACCCCGGCGAGGGG-AGTGAAATAGAACCTGAAACCGTATGCGTACAAGCAGTAGGAGCATTTCTTCGGAAATGTGACTGCGTACCTTTTGTATAATGGGTCAGC 583

NZ_CP103426.1:167642-171981(+) CCGATAGCGAACCAGTACCGTGAGGGAAAG------GTGAAAAGAACCCCGGCGAGGGG-AGTGAAATAGAACCTGAAACCGTATGCGTACAAGCAGTAGGAGCATTTCTTCGGAAATGTGACTGCGTACCTTTTGTATAATGGGTCAGC 583

NZ_JAOXDN010000007.1:36985-41324(-) CCGATAGCGAACCAGTACCGTGAGGGAAAG------GTGAAAAGAACCCCGGCGAGGGG-AGTGAAATAGAACCTGAAACCGTATGCGTACAAGCAGTAGGAGCATTTCTTCGGAAATGTGACTGCGTACCTTTTGTATAATGGGTCAGC 583

NZ_JPVV01000013.1:13888-18051(+) CCGATAGCGAACCAGTACCGTGAGGGAAAG------GTGAAAAGAACCCCGGCGAGGGG-AGTGAAATAGAACCTGAAACCGTATGCGTACAAGCAGTAGGAGCATTTCTTCGGAAGTGTGACTGCGTACCTTTTGTATAATGGGTCAGC 583

NZ_CP007555.1:337406-341752(+) CCGATAGCGAACCAGTACCGTGAGGGAAAG------GTGAAAAGAACCCCGGCGAGGGG-AGTGAAATAGAACCTGAAACCGTATGCGTACAAGCAGTAGGAGCATTTCTTCGGAAGTGTGACTGCGTACCTTTTGTATAATGGGTCAGC 583

NZ_JASNNV010000010.1:39284-43631(-) CCGATAGCGAACCAGTACCGTGAGGGAAAG------GTGAAAAGAACCCCGGCGAGGGG-AGTGAAATAGAACCTGAAACCGTATGCGTACAAGCAGTAGGAGCATTTCTTCGGAAGTGTGACTGCGTACCTTTTGTATAATGGGTCAGC 583

NZ_JAOXFC010000012.1:14231-18577(+) CCGATAGCGAACCAGTACCGTGAGGGAAAG------GTGAAAAGAACCCCGGCGAGGGG-AGTGAAATAGAACCTGAAACCGTATGCGTACAAGCAGTAGGAGCATTTCTTCGGAAGTGTGACTGCGTACCTTTTGTATAATGGGTCAGC 583

NZ_CP103430.1:167658-171997(+) CCGATAGCGAACCAGTACCGTGAGGGAAAG------GTGAAAAGAACCCCGGCGAGGGG-AGTGAAATAGAACCTGAAACCGTATGCGTACAAGCAGTAGGAGCATTTCTTCGGAAGTGTGACTGCGTACCTTTTGTATAATGGGTCAGC 583

NZ_PDLP01000011.1:14441-18787(+) CCGATAGCGAACCAGTACCGTGAGGGAAAG------GTGAAAAGAACCCCGGCGAGGGG-AGTGAAATAGAACCTGAAACCGTATGCGTACAAGCAGTAGGAGCATTTCTTCGGAAGTGTGACTGCGTACCTTTTGTATAATGGGTCAGC 583

NZ_NOLM01000022.1:91220-95566(-) CCGATAGCGAACCAGTACCGTGAGGGAAAG------GTGAAAAGAACCCCGGCGAGGGG-AGTGAAATAGAACCTGAAACCGTATGCGTACAAGCAGTAGGAGCATTTCTTCGGAAGTGTGACTGCGTACCTTTTGTATAATGGGTCAGC 583

JBJCIS010000056.1:0-2780(-) CCGATAGCGAACCAGTACCGTGAGGGAAAG------GTGAAAAGAACCCCGGCGAGGGG-AGTGAAATAGAACCTGAAACCGTATGCGTACAAGCAGTAGGAGCATTTCTTCGGAAGTGTGACTGCGTACCTTTTGTATAATGGGTCAGC 583

NZ_CDBG01000001.1:179744-184090(+) CCGATAGCGAACCAGTACCGTGAGGGAAAG------GTGAAAAGAACCCCGGCGAGGGG-AGTGAAATAGAACCTGAAACCGTATGCGTACAAGCAGTAGGAGCATTTCTTCGGAARTGTGACTGCGTACCTTTTGTATAATGGGTCAGC 583

NZ_CP103434.1:167622-171961(+) CCGATAGCGAACCAGTACCGTGAGGGAAAG------GTGAAAAGAACCCCGGCGAGGGG-AGTGAAATAGAACCTGAAACCGTATGCGTACAAGCAGTAGGAGCATTTCTTCGGAAATGTGACTGCGTACCTTTTGTATAATGGGTCAGC 583

CP014354.1:76692-80603(-) -----------------------------G------GTGAAAAGAACCCCGGCGAGGGG-AGTGAAATAGAACCTGAAACCGTATGCGTACAAGCAGTAGGAGCATTTCTTCGGAAATGTGACTGCGTACCTTTTGTATAATGGGTCAGC 114

NOLR01000002.1:131833-136207(+) CCGATAGCGAACCAGTACCGTGAGGGAAAG------GTGAAAAGAACCCCGGCGAGGGG-AGTGAAATAGAACCTGAAACCGTATGCGTACAAGCAGTAGGAGCATTTCTTCGGAAATGTGACTGCGTACCTTTTGTATAATGGGTCAGC 593

PPFR01000008.1:34360-38734(-) CCGATAGCGAACCAGTACCGTGAGGGAAAG------GTGAAAAGAACCCCGGCGAGGGG-AGTGAAATAGAACCTGAAACCGTATGCGTACAAGCAGTAGGAGCATTTCTTCGGAAATGTGACTGCGTACCTTTTGTATAATGGGTCAGC 593

PPFQ01000003.1:131083-135464(+) CCGATAGCGAACCAGTACCGTGAGGGAAAG------GTGAAAAGAACCCCGGCGAGGGG-AGTGAAATAGAACCTGAAACCGTATGCGTACAAGCAGTAGGAGCATTTCTTCGGAAATGTGACTGCGTACCTTTTGTATAATGGGTCAGC 593

NZ_AKYP01000181.1:1647-6020(-) CCGATAGCGAACCAGTACCGTGAGGGAAAG------GTGAAAAGAACCCCGGCGAGGGG-AGTGAAATAGAACCTGAAACCGTATGCGTACAAGCAGTAGGAGCATTTCTTCGGAAATGTGACTGCGTACCTTTTGTATAATGGGTCAGC 593

NOVI01000003.1:105955-110328(+) CCGATAGCGAACCAGTACCGTGAGGGAAAG------GTGAAAAGAACCCCGGCGAGGGG-AGTGAAATAGAACCTGAAACCGTATGCGTACAAGCAGTAGGAGCATTTCTTCGGAAATGTGACTGCGTACCTTTTGTATAATGGGTCAGC 593

CP018150.1:339294-343661(+) CCGATAGCGAACCAGTACCGTGAGGGAAAG------GTGAAAAGAACCCCGGCGAGGGG-AGTGAAATAGAACCTGAAACCGTATGCGTACAAGCAGTAGGAGCATTTCTTCGGAAGTGTGACTGCGTACCTTTTGTATAATGGGTCAGC 593

Consensus GACTTACTTGTTGTAGCGAGCTTAACCGTCTAGGGGAGGCGTAGGGAAACCGAGTCCGAAATGGGCGTTTAGTTGCAACGAGTAGACCCGAAACCGAGCGATCTATCTATGGCCAGGGTGAAGGTCAGGTAACACTGACTGGAGGCCCGA 743

JAKFBC010000002.1:34496-38842(-) (45) GACTTACTTGTTGTAGCGAGCTTAACCGTCTAGGGGAGGCGTAGGGAAACCGAGTCCGAAATGGGCGTTTAGTTGCAACGAGTAGACCCGAAACCGAGCGATCTATCTATGGCCAGGGTGAAGGTCAGGTAACACTGACTGGAGGCCCGA 733

NZ_AP019757.1:1739264-1743610(-) (31) GACTTACTTGTTGTAGCGAGCTTAACCGTCTAGGGGAGGCGTAGGGAAACCGAGTCCGAAATGGGCGTTTAGTTGCAACGAGTAGACCCGAAACCGAGCGATCTATCTATGGCCAGGGTGAAGGTCAGGTAACACTGACTGGAGGCCCGA 733

JBKOHN010000001.1:81530-85875(+) (25) GACTTACTTGTTGTAGCGAGCTTAACCGTCTAGGGGAGGCGTAGGGAAACCGAGTCCGAAATGGGCGTTTAGTTGCAACGAGTAGACCCGAAACCGAGCGATCTATCTATGGCCAGGGTGAAGGTCAGGTAACACTGACTGGAGGCCCGA 733

NC_011528.1:339307-343646(+) (14) GACTTACTTGTTGTAGCGAGCTTAACCGTCTAGGGGAGGCGTAGGGAAACCGAGTCCGAAATGGGCGTTTAGTTGCAACGAGTAGACCCGAAACCGAACGATCTATCTATGGCCAGGGTGAAGGTCAGGTAACACTGACTGGAGGCCCGA 733

CP018005.1:167566-171940(+) (14) GACTTACTTGTTGTAGCGAGCTTAACCGTCTAGGGGAGGCGTAGGGAAACCGAGTCCGAAATGGGCGTTTAGTTGCAACGAGTAGACCCGAAACCGAGCGATCTATCTATGGCCAGGGTGAAGGTCAGGTAACACTGACTGGAGGCCCGA 743

NZ_CCAM010000005.1:71418-75764(+) (13) GACTTACTTGTTGTAGCGAGCTTAACCGTCTAGGGGAGGCGTAGGGAAACCGAGTCCGAAATGGGCGTTTAGTTGCAACGAGTAGACCCGAAACCGAGCGATCTATCTATGGCCAGGGTGAAGGTCAGGTAACACTGACTGGAGGCCCGA 733

NZ_CCXO01000001.1:317226-321572(+) (11) GACTTACTTGTTGTAGCGAGCTTAACCGTCTAGGGGAGGCGTAGGGAAACCGAGTCCGAAATGGGCGTTTAGTTGCAACGAGTAGACCCGAAACCGAACGATCTATCTATGGCCAGGGTGAAGGTCAGGTAACACTGACTGGAGGCCCGA 733

NC_011527.1:1751498-1755844(-) (8) GACTTACTTGTTGTAGCGAGCTTAACCGTCTAGGGGAGGCGTAGGGAAACCGAGTCCGAAATGGGCGTTTAGTTGCAACGAGTAGACCCGAAACCGAGCGATCTATCTATGGCCAGGGTGAAGGTCAGGTAACACTGACTGGAGGCCCGA 733

NZ_CP032542.1:88036-92382(-) (8) GACTTACTTGTTGTAGCGAGCTTAACCGTCTAGGGGAGGCGTAGGGAAACCGAGTCCGAAATGGGCGTTTAGTTGCAACGAGTAGACCCGAAACCGAGCGATCTATCTATGGCCAGGGTGAAGGTCAGGTAACACTGACTGGAGGCCCGA 733

CP014551.1:161236-165610(+) (8) GACTTACTTGTTGTAGCGAGCTTAACCGTCTAGGGGAGGCGTAGGGAAACCGAGTCCGAAATGGGCGTTTAGTTGCAACGAGTAGACCCGAAACCGAGCGATCTATCTATGGCCAGGGTGAAGGTCAGGTAACACTGACTGGAGGCCCGA 743

CP013667.1:88011-92385(-) (8) GACTTACTTGTTGTAGCGAGCTTAACCGTCTAGGGGAGGCGTAGGGAAACCGAGTCCGAAATGGGCGTTTAGTTGCAACGAGTAGACCCGAAACCGAGCGATCTATCTATGGCCAGGGTGAAGGTCAGGTAACACTGACTGGAGGCCCGA 743

CP107247.1:164350-168689(+) (7) GACTTACTTGTTGTAGCGAGCTTAACCGTCTAGGGGAGGCGTAGGGAAACCGAGTCCGAAATGGGCGTTTAGTTGCAACGAGTAGACCCGAAACCGAACGATCTATCTATGGCCAGGGTGAAGGTCAGGTAACACTGACTGGAGGCCCGA 733

NZ_CP103435.1:167581-171920(+) (6) GACTTACTTGTTGTAGCGAGCTTAACCGTCTAGGGGAGGCGTAGGGAAACCGAGTCCGAAATGGGCGTTTAGTTGCAACGAGTAGACCCGAAACCGAGCGATCTATCTATGGCCAGGGTGAAGGTCAGGTAACACTGACTGGAGGCCCGA 733

CP014565.1:1711747-1716121(-) (3) GACTTACTTGTTGTAGCGAGCTTAACCGTCTAGGGGAGGCGTAGGGAAACCGAGTCCGAAATGGGCGTTTAGTTGCAACGAGTAGACCCGAAACCGAGCGATCTATCTATGGCCAGGGTGAAGGTCAGGTAACACTGACTGGAGGCCCGA 743

AAYJ01000007.1:0-1404(+) (3) ------------------------------------------------------------------------------------------------------------------------------------------------------

NZ_JAOXDR010000002.1:16672-21025(-) (3) GACTTACTTGTTGTAGCGAGCTTAACCGTCTAGGGGAGGCGTAGGGAAACCGAGTCCGAAATGGGCGTTTAGTTGCAACGAGTAGACCCGAAACCGAGCGATCTATCTATGGCCAGGGTGAAGGTCAGGTAACACTGACTGGAGGCCCGA 733

NZ_CP103432.1:167699-172038(+) (3) GACTTACTTGTTGTAGCGAGCTTAACCGTCTAGGGGAGGCGTAGGGAAACCGAGTCCGAAATGGGCGTTTAGTTGCAACGAGTAGACCCGAAACCGAGCGATCTATCTATGGCCAGGGTGAAGGTCAGGTAACACTGACTGGAGGCCCGA 733

NZ_CP103431.1:167697-172036(+) (3) GACTTACTTGTTGTAGCGAGCTTAACCGTCTAGGGGAGGCGTAGGGAAACCGAGTCCGAAATGGGCGTTTAGTTGCAACGAGTAGACCCGAAACCGAACGATCTATCTATGGCCAGGGTGAAGGTCAGGTAACACTGACTGGAGGCCCGA 733

NZ_CP103428.1:167591-171929(+) (2) GACTTACTTGTTGTAGCGAGCTTAACCGTCTAGGGGAGGCGTAGGGAAACCGAGTCCGAAATGGGCGTTTAGTTGCAACGAGTAGACCCGAAACCGAGCGATCTATCTATGGCCAGGGTGAAGGTCAGGTAACACTGACTGGAGGCCCGA 733

NZ_JANTNR010000002.1:115350-119696(+) (2) GACTTACTTGTTGTAGCGAGCTTAACCGTCTAGGGGAGGCGTAGGGAAACCGAGTCCGAAATGGGCGTTTAGTTGCAACGAGTAGACCCGAAACCGAGCGATCTATCTATGGCCAGGGTGAAGGTCAGGTAACACTGACTGGAGGCCCGA 733

JARBIR010004564.1:3782-8128(-) (2) GACTTACTTGTTGTAGCGAGCTTAACCGTCTAGGGGAGGCGTAGGGAAACCGAGTCCGAAATGGGCGTTTAGTTGCAACGAGTAGACCCGAAACCGAGCGATCTATCTATGGCCAGGGTGAAGGTCAGGTAACACTGACTGGAGGCCCGA 733

NC_009727.1:1888349-1892695(-) (2) GACTTACTTGTTGTAGCGAGCTTAACCGTCTAGGGGAGGCGTAGGGAAACCGAGTCCGAAATGGGCGTTTAGTTGCAACGAGTAGACCCGAAACCGAACGATCTATCTATGGCCAGGGTGAAGGTCAGGTAACACTGACTGGAGGCCCGA 733

AAYJ01000139.1:0-660(+) (2) ------------------------------------------------------------------------------------------------------------------------------------------------------

CP032542.1:88018-92392(-) (2) GACTTACTTGTTGTAGCGAGCTTAACCGTCTAGGGGAGGCGTAGGGAAACCGAGTCCGAAATGGGCGTTTAGTTGCAACGAGTAGACCCGAAACCGAGCGATCTATCTATGGCCAGGGTGAAGGTCAGGTAACACTGACTGGAGGCCCGA 743

CP014559.1:232744-237117(+) (2) GACTTACTTGTTGTAGCGAGCTTAACCGTCTAGGGGAGGCGTAGGGAAACCGAGTCCGAAATGGGCGTTTAGTTGCAACGAGTAGACCCGAAACCGAGCGATCTATCTATGGCCAGGGTGAAGGTCAGGTAACACTGACTGGAGGCCCGA 743

CP007555.1:337408-341752(+) GACTTACTTGTTGTAGCGAGCTTAACCGTCTAGGGGAGGCGTAGGGAAACCGAGTCCGAAATGGGCGTTTAGTTGCAACGAGTAGACCCGAAACCGAACGATCTATCTATGGCCAGGGTGAAGGTCAGGTAACACTGACTGGAGGCCCGA 731

NZ_CP014354.1:76710-81178(-) GACTTACTTGTTGTAGCGAGCTTAACCGTCTAGGGGAGGCGTAGGGAAACCGAGTCCGAAATGGGCGTTTAGTTGCAACGAGTAGACCCGAAACCGAGCGATCTATCTATGGCCAGGGTGAAGGTCAGGTAACACTGACTGGAGGCCCGA 739

CP000890.1:243129-244433(+) ------------------------------------------------------------------------------------------------------------------------------------------------------

NZ_CP115461.1:167576-171922(+) GACTTACTTGTTGTAGCGAGCTTAACCGTCTAGGGGAGGCGTAGGGAAACCGAGTCCGAAATGGGCGTTTAGTTGCAACGAGTAGACCCGAAACCGAGCGATCTATCTATGGCCAGGGTGAAGGTCAGGTAACACTGACTGGAGGCCCGA 733

AP019759.1:167578-171919(+) GACTTACTTGTTGTAGCGAGCTTAACCGTCTAGGGGAGGCGTAGGGAAACCGAGTCCGAAATGGGCGTTTAGTTGCAACGAGTAGACCCGAAACCGAGCGATCTATCTATGGCCAGGGTGAAGGTCAGGTAACACTGACTGGAGGCCCGA 731

AP019757.1:1739267-1743608(-) GACTTACTTGTTGTAGCGAGCTTAACCGTCTAGGGGAGGCGTAGGGAAACCGAGTCCGAAATGGGCGTTTAGTTGCAACGAGTAGACCCGAAACCGAGCGATCTATCTATGGCCAGGGTGAAGGTCAGGTAACACTGACTGGAGGCCCGA 731

NOLN01000021.1:90861-95235(-) GACTTACTTGTTGTAGCGAGCTTAACCGTCTAGGGGAGGCGTAGGGAAACCGAGTCCGAAATGGGCGTTTAGTTGCAACGAGTAGACCCGAAACCGAACGATCTATCTATGGCCAGGGTGAAGGTCAGGTAACACTGACTGGAGGCCCGA 743

NOLM01000022.1:91202-95576(-) GACTTACTTGTTGTAGCGAGCTTAACCGTCTAGGGGAGGCGTAGGGAAACCGAGTCCGAAATGGGCGTTTAGTTGCAACGAGTAGACCCGAAACCGAACGATCTATCTATGGCCAGGGTGAAGGTCAGGTAACACTGACTGGAGGCCCGA 743

PDLP01000011.1:14431-18805(+) GACTTACTTGTTGTAGCGAGCTTAACCGTCTAGGGGAGGCGTAGGGAAACCGAGTCCGAAATGGGCGTTTAGTTGCAACGAGTAGACCCGAAACCGAACGATCTATCTATGGCCAGGGTGAAGGTCAGGTAACACTGACTGGAGGCCCGA 743

CP014563.1:522940-527314(+) GACTTACTTGTTGTAGCGAGCTTAACCGTCTAGGGGAGGCGTAGGGAAACCGAGTCCGAAATGGGCGTTTAGTTGCAACGAGTAGACCCGAAACCGAACGATCTATCTATGGCCAGGGTGAAGGTCAGGTAACACTGACTGGAGGCCCGA 743

CP000890.1:244922-245905(+) ------------------------------------------------------------------------------------------------------------------------------------------------------

NZ_NOLR01000002.1:131843-136189(+) GACTTACTTGTTGTAGCGAGCTTAACCGTCTAGGGGAGGCGTAGGGAAACCGAGTCCGAAATGGGCGTTTAGTTGCAACGAGTAGACCCGAAACCGAGCGATCTATCTATGGCCAGGGTGAAGGTCAGGTAACACTGACTGGAGGCCCGA 733

NZ_JAOXDP010000002.1:15794-20139(-) GACTTACTTGTTGTAGCGAGCTTAACCGTCTAGGGGAGGCGTAGGGAAACCGAGTCCGAAATGGGCGTTTAGTTGCAACGAGTAGACCCGAAACCGAGCGATCTATCTATGGCCAGGGTGAAGGTCAGGTAACACTGACTGGAGGCCCGA 733

NZ_PPFR01000008.1:34378-38724(-) GACTTACTTGTTGTAGCGAGCTTAACCGTCTAGGGGAGGCGTAGGGAAACCGAGTCCGAAATGGGCGTTTAGTTGCAACGAGTAGACCCGAAACCGAGCGATCTATCTATGGCCAGGGTGAAGGTCAGGTAACACTGACTGGAGGCCCGA 733

NZ_PPFQ01000003.1:131093-135446(+) GACTTACTTGTTGTAGCGAGCTTAACCGTCTAGGGGAGGCGTAGGGAAACCGAGTCCGAAATGGGCGTTTAGTTGCAACGAGTAGACCCGAAACCGAGCGATCTATCTATGGCCAGGGTGAAGGTCAGGTAACACTGACTGGAGGCCCGA 733

NZ_LK937696.1:167613-171953(+) GACTTACTTGTTGTAGCGAGCTTAACCGTCTAGGGGAGGCGTAGGGAAACCGAGTCCGAAATGGGCGTTTAGTTGCAACGAGTAGACCCGAAACCGAGCGATCTATCTATGGCCAGGGTGAAGGTCAGGTAACACTGACTGGAGGCCCGA 733

NZ_NOVI01000003.1:105965-110310(+) GACTTACTTGTTGTAGCGAGCTTAACCGTCTAGGGGAGGCGTAGGGAAACCGAGTCCGAAATGGGCGTTTAGTTGCAACGAGTAGACCCGAAACCGAGCGATCTATCTATGGCCAGGGTGAAGGTCAGGTAACACTGACTGGAGGCCCGA 733

NZ_CP103426.1:167642-171981(+) GACTTACTTGTTGTAGCGAGCTTAACCGTCTAGGGGAGGCGTAGGGAAACCGAGTCCGAAATGGGCGTTTAGTTGCAACGAGTAGACCCGAAACCGAGCGATCTATCTATGGCCAGGGTGAAGGTCAGGTAACACTGACTGGAGGCCCGA 733

NZ_JAOXDN010000007.1:36985-41324(-) GACTTACTTGTTGTAGCGAGCTTAACCGTCTAGGGGAGGCGTAGGGAAACCGAGTCCGAAATGGGCGTTTAGTTGCAACGAGTAGACCCGAAACCGAGCGATCTATCTATGGCCAGGGTGAAGGTCAGGTAACACTGACTGGAGGCCCGA 733

NZ_JPVV01000013.1:13888-18051(+) GACTTACTTGTTGTAGCGAGCTTAACCGTCTAGGGGAGGCGTAGGGAAACCGAGTCCGAAATGGGCGTTTAGTTGCAACGAGTAGACCCGAAACCGAACGATCTATCTATGGCCAGGGTGAAGGTCAGGTAACACTGACTGGAGGCCCGA 733

NZ_CP007555.1:337406-341752(+) GACTTACTTGTTGTAGCGAGCTTAACCGTCTAGGGGAGGCGTAGGGAAACCGAGTCCGAAATGGGCGTTTAGTTGCAACGAGTAGACCCGAAACCGAACGATCTATCTATGGCCAGGGTGAAGGTCAGGTAACACTGACTGGAGGCCCGA 733

NZ_JASNNV010000010.1:39284-43631(-) GACTTACTTGTTGTAGCGAGCTTAACCGTCTAGGGGAGGCGTAGGGAAACCGAGTCCGAAATGGGCGTTTAGTTGCAACGAGTAGACCCGAAACCGAACGATCTATCTATGGCCAGGGTGAAGGTCAGGTAACACTGACTGGAGGCCCGA 733

NZ_JAOXFC010000012.1:14231-18577(+) GACTTACTTGTTGTAGCGAGCTTAACCGTCTAGGGGAGGCGTAGGGAAACCGAGTCCGAAATGGGCGTTTAGTTGCAACGAGTAGACCCGAAACCGAACGATCTATCTATGGCCAGGGTGAAGGTCAGGTAACACTGACTGGAGGCCCGA 733

NZ_CP103430.1:167658-171997(+) GACTTACTTGTTGTAGCGAGCTTAACCGTCTAGGGGAGGCGTAGGGAAACCGAGTCCGAAATGGGCGTTTAGTTGCAACGAGTAGACCCGAAACCGAACGATCTATCTATGGCCAGGGTGAAGGTCAGGTAACACTGACTGGAGGCCCGA 733

NZ_PDLP01000011.1:14441-18787(+) GACTTACTTGTTGTAGCGAGCTTAACCGTCTAGGGGAGGCGTAGGGAAACCGAGTCCGAAATGGGCGTTTAGTTGCAACGAGTAGACCCGAAACCGAACGATCTATCTATGGCCAGGGTGAAGGTCAGGTAACACTGACTGGAGGCCCGA 733

NZ_NOLM01000022.1:91220-95566(-) GACTTACTTGTTGTAGCGAGCTTAACCGTCTAGGGGAGGCGTAGGGAAACCGAGTCCGAAATGGGCGTTTAGTTGCAACGAGTAGACCCGAAACCGAACGATCTATCTATGGCCAGGGTGAAGGTCAGGTAACACTGACTGGAGGCCCGA 733

JBJCIS010000056.1:0-2780(-) GACTTACTTGTTGTAGCGAGCTTAACCGTCTAGGGGAGGCGTAGGGAAACCGAGTCCGAAATGGGCGTTTAGTTGCAACGAGTAGACCCGAAACCGAACGATCTATCTATGGCCAGGGTGAAGGTCAGGTAACACTGACTGGAGGCCCGA 733

NZ_CDBG01000001.1:179744-184090(+) GACTTACTTGTTGTAGCGAGCTTAACCGTCTAGGGGAGGCGTAGGGAAACCGAGTCCGAAATGGGCGTTTAGTTGCAACGAGTAGACCCGAAACCGARCGATCTATCTATGGCCAGGGTGAAGGTCAGGTAACACTGACTGGAGGCCCGA 733

NZ_CP103434.1:167622-171961(+) GACTTACTTGTTGTAGCGAGCTTAACCGTCTAGGGGAGGCGTAGGGAAACCGAGTCCGAAATGGGCGTTTAGTTGCAACGAGTAGACCCGAAACCGAGCGATCTATCTATGGCCAGGGTGAAGGTCAGGTAACACTGACTGGAGGCCCGA 733

CP014354.1:76692-80603(-) GACTTACTTGTTGTAGCGAGCTTAACCGTCTAGGGGAGGCGTAGGGAAACCGAGTCCGAAATGGGCGTTTAGTTGCAACGAGTAGACCCGAAACCGAGCGATCTATCTATGGCCAGGGTGAAGGTCAGGTAACACTGACTGGAGGCCCGA 264

NOLR01000002.1:131833-136207(+) GACTTACTTGTTGTAGCGAGCTTAACCGTCTAGGGGAGGCGTAGGGAAACCGAGTCCGAAATGGGCGTTTAGTTGCAACGAGTAGACCCGAAACCGAGCGATCTATCTATGGCCAGGGTGAAGGTCAGGTAACACTGACTGGAGGCCCGA 743

PPFR01000008.1:34360-38734(-) GACTTACTTGTTGTAGCGAGCTTAACCGTCTAGGGGAGGCGTAGGGAAACCGAGTCCGAAATGGGCGTTTAGTTGCAACGAGTAGACCCGAAACCGAGCGATCTATCTATGGCCAGGGTGAAGGTCAGGTAACACTGACTGGAGGCCCGA 743

PPFQ01000003.1:131083-135464(+) GACTTACTTGTTGTAGCGAGCTTAACCGTCTAGGGGAGGCGTAGGGAAACCGAGTCCGAAATGGGCGTTTAGTTGCAACGAGTAGACCCGAAACCGAGCGATCTATCTATGGCCAGGGTGAAGGTCAGGTAACACTGACTGGAGGCCCGA 743

NZ_AKYP01000181.1:1647-6020(-) GACTTACTTGTTGTAGCGAGCTTAACCGTCTAGGGGAGGCGTAGGGAAACCGAGTCCGAAATGGGCGTTTAGTTGCAACGAGTAGACCCGAAACCGAGCGATCTATCTATGGCCAGGGTGAAGGTCAGGTAACACTGACTGGAGGCCCGA 743

NOVI01000003.1:105955-110328(+) GACTTACTTGTTGTAGCGAGCTTAACCGTCTAGGGGAGGCGTAGGGAAACCGAGTCCGAAATGGGCGTTTAGTTGCAACGAGTAGACCCGAAACCGAGCGATCTATCTATGGCCAGGGTGAAGGTCAGGTAACACTGACTGGAGGCCCGA 743

CP018150.1:339294-343661(+) GACTTACTTGTTGTAGCGAGCTTAACCGTCTAGGGGAGGCGTAGGGAAACCGAGTCCGAAATGGGCGTTTAGTTGCAACGAGTAGACCCGAAACCGAACGATCTATCTATGGCCAGGGTGAAGGTCAGGTAACACTGACTGGAGGCCCGA 743

Consensus ACCCACTAATGTTGAAAAATTAGGGGATGAGCTGTGGATAGGAGTGAAAGGCTAATCAAGCTCGGAGATAGCTGGTTCTCCTCGAAAGCTATTTAGGTAGCGCCTCGTGTATGACTCTTGGGGGTAGAGCACTGTTTCGGCTAGGGGGCC 893

JAKFBC010000002.1:34496-38842(-) (45) ACCCACTAATGTTGAAAAATTAGGGGATGAGCTGTGGATAGGAGTGAAAGGCTAATCAAGCTCGGAGATAGCTGGTTCTCCTCGAAAGCTATTTAGGTAGCGCCTCGTGTATGACTCTTGGGGGTAGAGCACTGTTTCGGCTAGGGGGCC 883

NZ_AP019757.1:1739264-1743610(-) (31) ACCCACTAATGTTGAAAAATTAGGGGATGAGCTGTGGATAGGAGTGAAAGGCTAATCAAGCTCGGAGATAGCTGGTTCTCCTCGAAAGCTATTTAGGTAGCGCCTCGTGTATGACTCTTGGGGGTAGAGCACTGTTTCGGCTAGGGGGCC 883

JBKOHN010000001.1:81530-85875(+) (25) ACCCACTAATGTTGAAAAATTAGGGGATGAGCTGTGGATAGGAGTGAAAGGCTAATCAAGCTCGGAGATAGCTGGTTCTCCTCGAAAGCTATTTAGGTAGCGCCTCGTGTATGACTCTTGGGGGTAGAGCACTGTTTCGGCTAGGGGGCC 883

NC_011528.1:339307-343646(+) (14) ACCCACTAATGTTGAAAAATTAGGGGATGAGCTGTGGATAGGAGTGAAAGGCTAATCAAGCTCGGAGATAGCTGGTTCTCCTCGAAAGCTATTTAGGTAGCGCCTCGTGTATGACTCTTGGGGGTAGAGCACTGTTTCGGCTAGGGGGCC 883

CP018005.1:167566-171940(+) (14) ACCCACTAATGTTGAAAAATTAGGGGATGAGCTGTGGATAGGAGTGAAAGGCTAATCAAGCTCGGAGATAGCTGGTTCTCCTCGAAAGCTATTTAGGTAGCGCCTCGTGTATGACTCTTGGGGGTAGAGCACTGTTTCGGCTAGGGGGCC 893

NZ_CCAM010000005.1:71418-75764(+) (13) ACCCACTAATGTTGAAAAATTAGGGGATGAGCTGTGGATAGGAGTGAAAGGCTAATCAAGCTCGGAGATAGCTGGTTCTCCTCGAAAGCTATTTAGGTAGCGCCTCGTGTATGACTCTTGGGGGTAGAGCACTGTTTCGGCTAGGGGGCC 883

NZ_CCXO01000001.1:317226-321572(+) (11) ACCCACTAATGTTGAAAAATTAGGGGATGAGCTGTGGATAGGAGTGAAAGGCTAATCAAGCTCGGAGATAGCTGGTTCTCCTCGAAAGCTATTTAGGTAGCGCCTCGTGTATGACTCTTGGGGGTAGAGCACTGTTTCGGCTAGGGGGCC 883

NC_011527.1:1751498-1755844(-) (8) ACCCACTAATGTTGAAAAATTAGGGGATGAGCTGTGGATAGGAGTGAAAGGCTAATCAAGCTCGGAGATAGCTGGTTCTCCTCGAAAGCTATTTAGGTAGCGCCTCGTGTATGACTCTTGGGGGTAGAGCACTGTTTCGGCTAGGGGGCC 883

NZ_CP032542.1:88036-92382(-) (8) ACCCACTAATGTTGAAAAATTAGGGGATGAGCTGTGGATAGGAGTGAAAGGCTAATCAAGCTCGGAGATAGCTGGTTCTCCTCGAAAGCTATTTAGGTAGCGCCTCGTGTATGACTCTTGGGGGTAGAGCACTGTTTCGGCTAGGGGGCC 883

CP014551.1:161236-165610(+) (8) ACCCACTAATGTTGAAAAATTAGGGGATGAGCTGTGGATAGGAGTGAAAGGCTAATCAAGCTCGGAGATAGCTGGTTCTCCTCGAAAGCTATTTAGGTAGCGCCTCGTGTATGACTCTTGGGGGTAGAGCACTGTTTCGGCTAGGGGGCC 893

CP013667.1:88011-92385(-) (8) ACCCACTAATGTTGAAAAATTAGGGGATGAGCTGTGGATAGGAGTGAAAGGCTAATCAAGCTCGGAGATAGCTGGTTCTCCTCGAAAGCTATTTAGGTAGCGCCTCGTGTATGACTCTTGGGGGTAGAGCACTGTTTCGGCTAGGGGGCC 893

CP107247.1:164350-168689(+) (7) ACCCACTAATGTTGAAAAATTAGGGGATGAGCTGTGGATAGGAGTGAAAGGCTAATCAAGCTCGGAGATAGCTGGTTCTCCTCGAAAGCTATTTAGGTAGCGCCTCGTGTATGACTCTTGGGGGTAGAGCACTGTTTCGGCTAGGGGGCC 883

NZ_CP103435.1:167581-171920(+) (6) ACCCACTAATGTTGAAAAATTAGGGGATGAGCTGTGGATAGGAGTGAAAGGCTAATCAAGCTCGGAGATAGCTGGTTCTCCTCGAAAGCTATTTAGGTAGCGCCTCGTGTATGACTCTTGGGGGTAGAGCACTGTTTCGGCTAGGGGGCC 883

CP014565.1:1711747-1716121(-) (3) ACCCACTAATGTTGAAAAATTAGGGGATGAGCTGTGGATAGGAGTGAAAGGCTAATCAAGCTCGGAGATAGCTGGTTCTCCTCGAAAGCTATTTAGGTAGCGCCTCGTGTATGACTCTTGGGGGTAGAGCACTGTTTCGGCTAGGGGGCC 893

AAYJ01000007.1:0-1404(+) (3) ------------------------------------------------------------------------------------------------------------------------------------------------------

NZ_JAOXDR010000002.1:16672-21025(-) (3) ACCCACTAATGTTGAAAAATTAGGGGATGAGCTGTGGATAGGAGTGAAAGGCTAATCAAGCTCGGAGATAGCTGGTTCTCCTCGAAAGCTATTTAGGTAGCGCCTCGTGTATGACTCTTGGGGGTAGAGCACTGTTTCGGCTAGGGGGCC 883

NZ_CP103432.1:167699-172038(+) (3) ACCCACTAATGTTGAAAAATTAGGGGATGAGCTGTGGATAGGAGTGAAAGGCTAATCAAGCTCGGAGATAGCTGGTTCTCCTCGAAAGCTATTTAGGTAGCGCCTCGTGTATGACTCTTGGGGGTAGAGCACTGTTTCGGCTAGGGGGCC 883

NZ_CP103431.1:167697-172036(+) (3) ACCCACTAATGTTGAAAAATTAGGGGATGAGCTGTGGATAGGAGTGAAAGGCTAATCAAGCTCGGAGATAGCTGGTTCTCCTCGAAAGCTATTTAGGTAGCGCCTCGTGTATGACTCTTGGGGGTAGAGCACTGTTTCGGCTAGGGGGCC 883

NZ_CP103428.1:167591-171929(+) (2) ACCCACTAATGTTGAAAAATTAGGGGATGAGCTGTGGATAGGAGTGAAAGGCTAATCAAGCTCGGAGATAGCTGGTTCTCCTCGAAAGCTATTTAGGTAGCGCCTCGTGTATGACTCTTGGGGGTAGAGCACTGTTTCGGCTAGGGGGCC 883

NZ_JANTNR010000002.1:115350-119696(+) (2) ACCCACTAATGTTGAAAAATTAGGGGATGAGCTGTGGATAGGAGTGAAAGGCTAATCAAGCTCGGAGATAGCTGGTTCTCCTCGAAAGCTATTTAGGTAGCGCCTCGTGTATGACTCTTGGGGGTAGAGCACTGTTTCGGCTAGGGGGCC 883

JARBIR010004564.1:3782-8128(-) (2) ACCCACTAATGTTGAAAAATTAGGGGATGAGCTGTGGATAGGAGTGAAAGGCTAATCAAGCTCGGAGATAGCTGGTTCTCCTCGAAAGCTATTTAGGTAGCGCCTCGTGTATGACTCTTGGGGGTAGAGCACTGTTTCGGCTAGGGGGCC 883

NC_009727.1:1888349-1892695(-) (2) ACCCACTAATGTTGAAAAATTAGGGGATGAGCTGTGGATAGGAGTGAAAGGCTAATCAAGCTCGGAGATAGCTGGTTCTCCTCGAAAGCTATTTAGGTAGCGCCTCGTGTATGACTCTTGGGGGTAGAGCACTGTTTCGGCTAGGGGGCC 883

AAYJ01000139.1:0-660(+) (2) ------------------------------------------------------------------------------------------------------------------------------------------------------

CP032542.1:88018-92392(-) (2) ACCCACTAATGTTGAAAAATTAGGGGATGAGCTGTGGATAGGAGTGAAAGGCTAATCAAGCTCGGAGATAGCTGGTTCTCCTCGAAAGCTATTTAGGTAGCGCCTCGTGTATGACTCTTGGGGGTAGAGCACTGTTTCGGCTAGGGGGCC 893

CP014559.1:232744-237117(+) (2) ACCCACTAATGTTGAAAAATTAGGGGATGAGCTGTGGATAGGAGTGAAAGGCTAATCAAGCTCGGAGATAGCTGGTTCTCCTCGAAAGCTATTTAGGTAGCGCCTCGTGTATGACTCTTGGGGGTAGAGCACTGTTTCGGCTAGGGGGCC 893

CP007555.1:337408-341752(+) ACCCACTAATGTTGAAAAATTAGGGGATGAGCTGTGGATAGGAGTGAAAGGCTAATCAAGCTCGGAGATAGCTGGTTCTCCTCGAAAGCTATTTAGGTAGCGCCTCGTGTATGACTCTTGGGGGTAGAGCACTGTTTCGGCTAGGGGGCC 881

NZ_CP014354.1:76710-81178(-) ACCCACTAATGTTGAAAAATTAGGGGATGAGCTGTGGATAGGAGTGAAAGGCTAATCAAGCTCGGAGATAGCTGGTTCTCCTCGAAAGCTATTTAGGTAGCGCCTCGTGTATGACTCTTGGGGGTAGAGCACTGTTTCGGCTAGGGGGCC 889

CP000890.1:243129-244433(+) ------------------------------------------------------------------------------------------------------------------------------------------------------

NZ_CP115461.1:167576-171922(+) ACCCACTAATGTTGAAAAATTAGGGGATGAGCTGTGGATAGGAGTGAAAGGCTAATCAAGCTCGGAGATAGCTGGTTCTCCTCGAAAGCTATTTAGGTAGCGCCTCGTGTATGACTCTTGGGGGTAGAGCACTGTTTCGGCTAGGGGGCC 883

AP019759.1:167578-171919(+) ACCCACTAATGTTGAAAAATTAGGGGATGAGCTGTGGATAGGAGTGAAAGGCTAATCAAGCTCGGAGATAGCTGGTTCTCCTCGAAAGCTATTTAGGTAGCGCCTCGTGTATGACTCTTGGGGGTAGAGCACTGTTTCGGCTAGGGGGCC 881

AP019757.1:1739267-1743608(-) ACCCACTAATGTTGAAAAATTAGGGGATGAGCTGTGGATAGGAGTGAAAGGCTAATCAAGCTCGGAGATAGCTGGTTCTCCTCGAAAGCTATTTAGGTAGCGCCTCGTGTATGACTCTTGGGGGTAGAGCACTGTTTCGGCTAGGGGGCC 881

NOLN01000021.1:90861-95235(-) ACCCACTAATGTTGAAAAATTAGGGGATGAGCTGTGGATAGGAGTGAAAGGCTAATCAAGCTCGGAGATAGCTGGTTCTCCTCGAAAGCTATTTAGGTAGCGCCTCGTGTATGACTCTTGGGGGTAGAGCACTGTTTCGGCTAGGGGGCC 893

NOLM01000022.1:91202-95576(-) ACCCACTAATGTTGAAAAATTAGGGGATGAGCTGTGGATAGGAGTGAAAGGCTAATCAAGCTCGGAGATAGCTGGTTCTCCTCGAAAGCTATTTAGGTAGCGCCTCGTGTATGACTCTTGGGGGTAGAGCACTGTTTCGGCTAGGGGGCC 893

PDLP01000011.1:14431-18805(+) ACCCACTAATGTTGAAAAATTAGGGGATGAGCTGTGGATAGGAGTGAAAGGCTAATCAAGCTCGGAGATAGCTGGTTCTCCTCGAAAGCTATTTAGGTAGCGCCTCGTGTATGACTCTTGGGGGTAGAGCACTGTTTCGGCTAGGGGGCC 893

CP014563.1:522940-527314(+) ACCCACTAATGTTGAAAAATTAGGGGATGAGCTGTGGATAGGAGTGAAAGGCTAATCAAGCTCGGAGATAGCTGGTTCTCCTCGAAAGCTATTTAGGTAGCGCCTCGTGTATGACTCTTGGGGGTAGAGCACTGTTTCGGCTAGGGGGCC 893

CP000890.1:244922-245905(+) ------------------------------------------------------------------------------------------------------------------------------------------------------

NZ_NOLR01000002.1:131843-136189(+) ACCCACTAATGTTGAAAAATTAGGGGATGAGCTGTGGATAGGAGTGAAAGGCTAATCAAGCTCGGAGATAGCTGGTTCTCCTCGAAAGCTATTTAGGTAGCGCCTCGTGTATGACTCTTGGGGGTAGAGCACTGTTTCGGCTAGGGGGCC 883

NZ_JAOXDP010000002.1:15794-20139(-) ACCCACTAATGTTGAAAAATTAGGGGATGAGCTGTGGATAGGAGTGAAAGGCTAATCAAGCTCGGAGATAGCTGGTTCTCCTCGAAAGCTATTTAGGTAGCGCCTCGTGTATGACTCTTGGGGGTAGAGCACTGTTTCGGCTAGGGGGCC 883

NZ_PPFR01000008.1:34378-38724(-) ACCCACTAATGTTGAAAAATTAGGGGATGAGCTGTGGATAGGAGTGAAAGGCTAATCAAGCTCGGAGATAGCTGGTTCTCCTCGAAAGCTATTTAGGTAGCGCCTCGTGTATGACTCTTGGGGGTAGAGCACTGTTTCGGCTAGGGGGCC 883

NZ_PPFQ01000003.1:131093-135446(+) ACCCACTAATGTTGAAAAATTAGGGGATGAGCTGTGGATAGGAGTGAAAGGCTAATCAAGCTCGGAGATAGCTGGTTCTCCTCGAAAGCTATTTAGGTAGCGCCTCGTGTATGACTCTTGGGGGTAGAGCACTGTTTCGGCTAGGGGGCC 883

NZ_LK937696.1:167613-171953(+) ACCCACTAATGTTGAAAAATTAGGGGATGAGCTGTGGATAGGAGTGAAAGGCTAATCAAGCTCGGAGATAGCTGGTTCTCCTCGAAAGCTATTTAGGTAGCGCCTCGTGTATGACTCTTGGGGGTAGAGCACTGTTTCGGCTAGGGGGCC 883

NZ_NOVI01000003.1:105965-110310(+) ACCCACTAATGTTGAAAAATTAGGGGATGAGCTGTGGATAGGAGTGAAAGGCTAATCAAGCTCGGAGATAGCTGGTTCTCCTCGAAAGCTATTTAGGTAGCGCCTCGTGTATGACTCTTGGGGGTAGAGCACTGTTTCGGCTAGGGGGCC 883

NZ_CP103426.1:167642-171981(+) ACCCACTAATGTTGAAAAATTAGGGGATGAGCTGTGGATAGGAGTGAAAGGCTAATCAAGCTCGGAGATAGCTGGTTCTCCTCGAAAGCTATTTAGGTAGCGCCTCGTGTATGACTCTTGGGGGTAGAGCACTGTTTCGGCTAGGGGGCC 883

NZ_JAOXDN010000007.1:36985-41324(-) ACCCACTAATGTTGAAAAATTAGGGGATGAGCTGTGGATAGGAGTGAAAGGCTAATCAAGCTCGGAGATAGCTGGTTCTCCTCGAAAGCTATTTAGGTAGCGCCTCGTGTATGACTCTTGGGGGTAGAGCACTGTTTCGGCTAGGGGGCC 883

NZ_JPVV01000013.1:13888-18051(+) ACCCACTAATGTTGAAAAATTAGGGGATGAGCTGTGGATAGGAGTGAAAGGCTAATCAAGCTCGGAGATAGCTGGTTCTCCTCGAAAGCTATTTAGGTAGCGCCTCGTGTATGACTCTTGGGGGTAGAGCACTGTTTCGGCTAGGGGGCC 883

NZ_CP007555.1:337406-341752(+) ACCCACTAATGTTGAAAAATTAGGGGATGAGCTGTGGATAGGAGTGAAAGGCTAATCAAGCTCGGAGATAGCTGGTTCTCCTCGAAAGCTATTTAGGTAGCGCCTCGTGTATGACTCTTGGGGGTAGAGCACTGTTTCGGCTAGGGGGCC 883

NZ_JASNNV010000010.1:39284-43631(-) ACCCACTAATGTTGAAAAATTAGGGGATGAGCTGTGGATAGGAGTGAAAGGCTAATCAAGCTCGGAGATAGCTGGTTCTCCTCGAAAGCTATTTAGGTAGCGCCTCGTGTATGACTCTTGGGGGTAGAGCACTGTTTCGGCTAGGGGGCC 883

NZ_JAOXFC010000012.1:14231-18577(+) ACCCACTAATGTTGAAAAATTAGGGGATGAGCTGTGGATAGGAGTGAAAGGCTAATCAAGCTCGGAGATAGCTGGTTCTCCTCGAAAGCTATTTAGGTAGCGCCTCGTGTATGACTCTTGGGGGTAGAGCACTGTTTCGGCTAGGGGGCC 883

NZ_CP103430.1:167658-171997(+) ACCCACTAATGTTGAAAAATTAGGGGATGAGCTGTGGATAGGAGTGAAAGGCTAATCAAGCTCGGAGATAGCTGGTTCTCCTCGAAAGCTATTTAGGTAGCGCCTCGTGTATGACTCTTGGGGGTAGAGCACTGTTTCGGCTAGGGGGCC 883

NZ_PDLP01000011.1:14441-18787(+) ACCCACTAATGTTGAAAAATTAGGGGATGAGCTGTGGATAGGAGTGAAAGGCTAATCAAGCTCGGAGATAGCTGGTTCTCCTCGAAAGCTATTTAGGTAGCGCCTCGTGTATGACTCTTGGGGGTAGAGCACTGTTTCGGCTAGGGGGCC 883

NZ_NOLM01000022.1:91220-95566(-) ACCCACTAATGTTGAAAAATTAGGGGATGAGCTGTGGATAGGAGTGAAAGGCTAATCAAGCTCGGAGATAGCTGGTTCTCCTCGAAAGCTATTTAGGTAGCGCCTCGTGTATGACTCTTGGGGGTAGAGCACTGTTTCGGCTAGGGGGCC 883

JBJCIS010000056.1:0-2780(-) ACCCACTAATGTTGAAAAATTAGGGGATGAGCTGTGGATAGGAGTGAAAGGCTAATCAAGCTCGGAGATAGCTGGTTCTCCTCGAAAGCTATTTAGGTAGCGCCTCGTGTATGACTCTTGGGGGTAGAGCACTGTTTCGGCTAGGGGGCC 883

NZ_CDBG01000001.1:179744-184090(+) ACCCACTAATGTTGAAAAATTAGGGGATGAGCTGTGGATAGGAGTGAAAGGCTAATCAAGCTCGGAGATAGCTGGTTCTCCTCGAAAGCTATTTAGGTAGCGCCTCGTGTATGACTCTTGGGGGTAGAGCACTGTTTCGGCTAGGGGGCC 883

NZ_CP103434.1:167622-171961(+) ACCCACTAATGTTGAAAAATTAGGGGATGAGCTGTGGATAGGAGTGAAAGGCTAATCAAGCTCGGAGATAGCTGGTTCTCCTCGAAAGCTATTTAGGTAGCGCCTCGTGTATGACTCTTGGGGGTAGAGCACTGTTTCGGCTAGGGGGCC 883

CP014354.1:76692-80603(-) ACCCACTAATGTTGAAAAATTAGGGGATGAGCTGTGGATAGGAGTGAAAGGCTAATCAAGCTCGGAGATAGCTGGTTCTCCTCGAAAGCTATTTAGGTAGCGCCTCGTGTATGACTCTTGGGGGTAGAGCACTGTTTCGGCTAGGGGGCC 414

NOLR01000002.1:131833-136207(+) ACCCACTAATGTTGAAAAATTAGGGGATGAGCTGTGGATAGGAGTGAAAGGCTAATCAAGCTCGGAGATAGCTGGTTCTCCTCGAAAGCTATTTAGGTAGCGCCTCGTGTATGACTCTTGGGGGTAGAGCACTGTTTCGGCTAGGGGGCC 893

PPFR01000008.1:34360-38734(-) ACCCACTAATGTTGAAAAATTAGGGGATGAGCTGTGGATAGGAGTGAAAGGCTAATCAAGCTCGGAGATAGCTGGTTCTCCTCGAAAGCTATTTAGGTAGCGCCTCGTGTATGACTCTTGGGGGTAGAGCACTGTTTCGGCTAGGGGGCC 893

PPFQ01000003.1:131083-135464(+) ACCCACTAATGTTGAAAAATTAGGGGATGAGCTGTGGATAGGAGTGAAAGGCTAATCAAGCTCGGAGATAGCTGGTTCTCCTCGAAAGCTATTTAGGTAGCGCCTCGTGTATGACTCTTGGGGGTAGAGCACTGTTTCGGCTAGGGGGCC 893

NZ_AKYP01000181.1:1647-6020(-) ACCCACTAATGTTGAAAAATTAGGGGATGAGCTGTGGATAGGAGTGAAAGGCTAATCAAGCTCGGAGATAGCTGGTTCTCCTCGAAAGCTATTTAGGTAGCGCCTCGTGTATGACTCTTGGGGGTAGAGCACTGTTTCGGCTAGGGGGCC 893

NOVI01000003.1:105955-110328(+) ACCCACTAATGTTGAAAAATTAGGGGATGAGCTGTGGATAGGAGTGAAAGGCTAATCAAGCTCGGAGATAGCTGGTTCTCCTCGAAAGCTATTTAGGTAGCGCCTCGTGTATGACTCTTGGGGGTAGAGCACTGTTTCGGCTAGGGGGCC 893

CP018150.1:339294-343661(+) ACCCACTAATGTTGAAAAATTAGGGGATGAGCTGTGGATAGGAGTGAAAGGCTAATCAAGCTCGGAGATAGCTGGTTCTCCTCGAAAGCTATTTAGGTAGCGCCTCGTGTATGACTCTTGGGGGTAGAGCACTGTTTCGGCTAGGGGGCC 893

Consensus ATCCCGGCCTACCAAACCGAGGCAAACTCCGAATACCAAGAAGTTTTAGCACGGGAGACACACTGCGGGTGATAAGGTCCGTGGTGGAAAGGGAAACAGCCCAGATCGCCAGCTAAGGTCCCAAAATCACAGTTAAGTGGAAAACGATGT 1043

JAKFBC010000002.1:34496-38842(-) (45) ATCCCGGCCTACCAAACCGAGGCAAACTCCGAATACCAAGAAGTTTTAGCACGGGAGACACACTGCGGGTGATAAGGTCCGTGGTGGAAAGGGAAACAGCCCAGATCGCCAGCTAAGGTCCCAAAATCACAGTTAAGTGGAAAACGATGT 1033

NZ_AP019757.1:1739264-1743610(-) (31) ATCCCGGCCTACCAAACCGAGGCAAACTCCGAATACCAAGAAGTTTTAGCACGGGAGACACACTGCGGGTGATAAGGTCCGTGGTGGAAAGGGAAACAGCCCAGATCGCCAGCTAAGGTCCCAAAATCACAGTTAAGTGGAAAACGATGT 1033

JBKOHN010000001.1:81530-85875(+) (25) ATCCCGGCCTACCAAACCGAGGCAAACTCCGAATACCAAGAAGTTTTAGCACGGGAGACACACTGCGGGTGATAAGGTCCGTGGTGGAAAGGGAAACAGCCCAGATCGCCAGCTAAGGTCCCAAAATCACAGTTAAGTGGAAAACGATGT 1033

NC_011528.1:339307-343646(+) (14) ATCCCGGCCTACCAAACCGAGGCAAACTCCGAATACCAAGAAGTTTTAGCACGGGAGACACACTGCGGGTGATAAGGTCCGTGGTGGAAAGGGAAACAGCCCAGATCGCCAGCTAAGGTCCCAAAATCACAGTTAAGTGGAAAACGATGT 1033

CP018005.1:167566-171940(+) (14) ATCCCGGCCTACCAAACCGAGGCAAACTCCGAATACCAAGAAGTTTTAGCACGGGAGACACACTGCGGGTGATAAGGTCCGTGGTGGAAAGGGAAACAGCCCAGATCGCCAGCTAAGGTCCCAAAATCACAGTTAAGTGGAAAACGATGT 1043

NZ_CCAM010000005.1:71418-75764(+) (13) ATCCCGGCCTACCAAACCGAGGCAAACTCCGAATACCAAGAAGTTTTAGCACGGGAGACACACTGCGGGTGATAAGGTCCGTGGTGGAAAGGGAAACAGCCCAGATCGCCAGCTAAGGTCCCAAAATCACAGTTAAGTGGAAAACGATGT 1033

NZ_CCXO01000001.1:317226-321572(+) (11) ATCCCGGCCTACCAAACCGAGGCAAACTCCGAATACCAAGAAGTTTTAGCACGGGAGACACACTGCGGGTGATAAGGTCCGTGGTGGAAAGGGAAACAGCCCAGATCGCCAGCTAAGGTCCCAAAATCACAGTTAAGTGGAAAACGATGT 1033

NC_011527.1:1751498-1755844(-) (8) ATCCCGGCCTACCAAACCGAGGCAAACTCCGAATACCAAGAAGTTTTAGCACGGGAGACACACTGCGGGTGATAAGGTCCGTGGTGGAAAGGGAAACAGCCCAGATCGCCAGCTAAGGTCCCAAAATCACAGTTAAGTGGAAAACGATGT 1033

NZ_CP032542.1:88036-92382(-) (8) ATCCCGGCCTACCAAACCGAGGCAAACTCCGAATACCAAGAAGTTTTAGCACGGGAGACACACTGCGGGTGATAAGGTCCGTGGTGGAAAGGGAAACAGCCCAGATCGCCAGCTAAGGTCCCAAAATCACAGTTAAGTGGAAAACGATGT 1033

CP014551.1:161236-165610(+) (8) ATCCCGGCCTACCAAACCGAGGCAAACTCCGAATACCAAGAAGTTTTAGCACGGGAGACACACTGCGGGTGATAAGGTCCGTGGTGGAAAGGGAAACAGCCCAGATCGCCAGCTAAGGTCCCAAAATCACAGTTAAGTGGAAAACGATGT 1043

CP013667.1:88011-92385(-) (8) ATCCCGGCCTACCAAACCGAGGCAAACTCCGAATACCAAGAAGTTTTAGCACGGGAGACACACTGCGGGTGATAAGGTCCGTGGTGGAAAGGGAAACAGCCCAGATCGCCAGCTAAGGTCCCAAAATCACAGTTAAGTGGAAAACGATGT 1043

CP107247.1:164350-168689(+) (7) ATCCCGGCCTACCAAACCGAGGCAAACTCCGAATACCAAGAAGTTTTAGCACGGGAGACACACTGCGGGTGATAAGGTCCGTGGTGGAAAGGGAAACAGCCCAGATCGCCAGCTAAGGTCCCAAAATCACAGTTAAGTGGAAAACGATGT 1033

NZ_CP103435.1:167581-171920(+) (6) ATCCCGGCCTACCAAACCGAGGCAAACTCCGAATACCAAGAAGTTTTAGCACGGGAGACACACTGCGGGTGATAAGGTCCGTGGTGGAAAGGGAAACAGCCCAGATCGCCAGCTAAGGTCCCAAAATCACAGTTAAGTGGAAAACGATGT 1033

CP014565.1:1711747-1716121(-) (3) ATCCCGGCCTACCAAACCGAGGCAAACTCCGAATACCAAGAAGTTTTAGCACGGGAGACACACTGCGGGTGATAAGGTCCGTGGTGGAAAGGGAAACAGCCCAGATCGCCAGCTAAGGTCCCAAAATCACAGTTAAGTGGAAAACGATGT 1043

AAYJ01000007.1:0-1404(+) (3) ------------------------------------------------------------------------------------------------------------------------------------------------------

NZ_JAOXDR010000002.1:16672-21025(-) (3) ATCCCGGCCTACCAAACCGAGGCAAACTCCGAATACCAAGAAGTTTTAGCACGGGAGACACACTGCGGGTGATAAGGTCCGTGGTGGAAAGGGAAACAGCCCAGATCGCCAGCTAAGGTCCCAAAATCACAGTTAAGTGGAAAACGATGT 1033

NZ_CP103432.1:167699-172038(+) (3) ATCCCGGCCTACCAAACCGAGGCAAACTCCGAATACCAAGAAGTTTTAGCACGGGAGACACACTGCGGGTGATAAGGTCCGTGGTGGAAAGGGAAACAGCCCAGATCGCCAGCTAAGGTCCCAAAATCACAGTTAAGTGGAAAACGATGT 1033

NZ_CP103431.1:167697-172036(+) (3) ATCCCGGCCTACCAAACCGAGGCAAACTCCGAATACCAAGAAGTTTTAGCACGGGAGACACACTGCGGGTGATAAGGTCCGTGGTGGAAAGGGAAACAGCCCAGATCGCCAGCTAAGGTCCCAAAATCACAGTTAAGTGGAAAACGATGT 1033

NZ_CP103428.1:167591-171929(+) (2) ATCCCGGCCTACCAAACCGAGGCAAACTCCGAATACCAAGAAGTTTTAGCACGGGAGACACACTGCGGGTGATAAGGTCCGTGGTGGAAAGGGAAACAGCCCAGATCGCCAGCTAAGGTCCCAAAATCACAGTTAAGTGGAAAACGATGT 1033

NZ_JANTNR010000002.1:115350-119696(+) (2) ATCCCGGCCTACCAAACCGAGGCAAACTCCGAATACCAAGAAGTTTTAGCACGGGAGACACACTGCGGGTGATAAGGTCCGTGGTGGAAAGGGAAACAGCCCAGATCGCCAGCTAAGGTCCCAAAATCACAGTTAAGTGGAAAACGATGT 1033

JARBIR010004564.1:3782-8128(-) (2) ATCCCGGCCTACCAAACCGAGGCAAACTCCGAATACCAAGAAGTTTTAGCACGGGAGACACACTGCGGGTGATAAGGTCCGTGGTGGAAAGGGAAACAGCCCAGATCGCCAGCTAAGGTCCCAAAATCACAGTTAAGTGGAAAACGATGT 1033

NC_009727.1:1888349-1892695(-) (2) ATCCCGGCCTACCAAACCGAGGCAAACTCCGAATACCAAGAAGTTTTAGCACGGGAGACACACTGCGGGTGATAAGGTCCGTGGTGGAAAGGGAAACAGCCCAGATCGCCAGCTAAGGTCCCAAAATCACAGTTAAGTGGAAAACGATGT 1033

AAYJ01000139.1:0-660(+) (2) ------------------------------------------------------------------------------------------------------------------------------------------------------

CP032542.1:88018-92392(-) (2) ATCCCGGCCTACCAAACCGAGGCAAACTCCGAATACCAAGAAGTTTTAGCACGGGAGACACACTGCGGGTGATAAGGTCCGTGGTGGAAAGGGAAACAGCCCAGATCGCCAGCTAAGGTCCCAAAATCACAGTTAAGTGGAAAACGATGT 1043

CP014559.1:232744-237117(+) (2) ATCCCGGCCTACCAAACCGAGGCAAACTCCGAATACCAAGAAGTTTTAGCACGGGAGACACACTGCGGGTGATAAGGTCCGTGGTGGAAAGGGAAACAGCCCAGATCGCCAGCTAAGGTCCCAAAATCACAGTTAAGTGGAAAACGATGT 1043

CP007555.1:337408-341752(+) ATCCCGGCCTACCAAACCGAGGCAAACTCCGAATACCAAGAAGTTTTAGCACGGGAGACACACTGCGGGTGATAAGGTCCGTGGTGGAAAGGGAAACAGCCCAGATCGCCAGCTAAGGTCCCAAAATCACAGTTAAGTGGAAAACGATGT 1031

NZ_CP014354.1:76710-81178(-) ATCCCGGCCTACCAAACCGAGGCAAACTCCGAATACCAAGAAGTTTTAGCACGGGAGACACACTGCGGGTGATAAGGTCCGTGGTGGAAAGGGAAACAGCCCAGATCGCCAGCTAAGGTCCCAAAATCACAGTTAAGTGGAAAACGATGT 1039

CP000890.1:243129-244433(+) ------------------------------------------------------------------------------------------------------------------------------------------------------

NZ_CP115461.1:167576-171922(+) ATCCCGGCCTACCAAACCGAGGCAAACTCCGAATACCAAGAAGTTTTAGCACGGGAGACACACTGCGGGTGATAAGGTCCGTGGTGGAAAGGGAAACAGCCCAGATCGCCAGCTAAGGTCCCAAAATCACAGTTAAGTGGAAAACGATGT 1033

AP019759.1:167578-171919(+) ATCCCGGCCTACCAAACCGAGGCAAACTCCGAATACCAAGAAGTTTTAGCACGGGAGACACACTGCGGGTGATAAGGTCCGTGGTGGAAAGGGAAACAGCCCAGATCGCCAGCTAAGGTCCCAAAATCACAGTTAAGTGGAAAACGATGT 1031

AP019757.1:1739267-1743608(-) ATCCCGGCCTACCAAACCGAGGCAAACTCCGAATACCAAGAAGTTTTAGCACGGGAGACACACTGCGGGTGATAAGGTCCGTGGTGGAAAGGGAAACAGCCCAGATCGCCAGCTAAGGTCCCAAAATCACAGTTAAGTGGAAAACGATGT 1031

NOLN01000021.1:90861-95235(-) ATCCCGGCCTACCAAACCGAGGCAAACTCCGAATACCAAGAAGTTTTAGCACGGGAGACACACTGCGGGTGATAAGGTCCGTGGTGGAAAGGGAAACAGCCCAGATCGCCAGCTAAGGTCCCAAAATCACAGTTAAGTGGAAAACGATGT 1043

NOLM01000022.1:91202-95576(-) ATCCCGGCCTACCAAACCGAGGCAAACTCCGAATACCAAGAAGTTTTAGCACGGGAGACACACTGCGGGTGATAAGGTCCGTGGTGGAAAGGGAAACAGCCCAGATCGCCAGCTAAGGTCCCAAAATCACAGTTAAGTGGAAAACGATGT 1043

PDLP01000011.1:14431-18805(+) ATCCCGGCCTACCAAACCGAGGCAAACTCCGAATACCAAGAAGTTTTAGCACGGGAGACACACTGCGGGTGATAAGGTCCGTGGTGGAAAGGGAAACAGCCCAGATCGCCAGCTAAGGTCCCAAAATCACAGTTAAGTGGAAAACGATGT 1043

CP014563.1:522940-527314(+) ATCCCGGCCTACCAAACCGAGGCAAACTCCGAATACCAAGAAGTTTTAGCACGGGAGACACACTGCGGGTGATAAGGTCCGTGGTGGAAAGGGAAACAGCCCAGATCGCCAGCTAAGGTCCCAAAATCACAGTTAAGTGGAAAACGATGT 1043

CP000890.1:244922-245905(+) ------------------------------------------------------------------------------------------------------------------------------------------------------

NZ_NOLR01000002.1:131843-136189(+) ATCCCGGCCTACCAAACCGAGGCAAACTCCGAATACCAAGAAGTTTTAGCACGGGAGACACACTGCGGGTGATAAGGTCCGTGGTGGAAAGGGAAACAGCCCAGATCGCCAGCTAAGGTCCCAAAATCACAGTTAAGTGGAAAACGATGT 1033

NZ_JAOXDP010000002.1:15794-20139(-) ATCCCGGCCTACCAAACCGAGGCAAACTCCGAATACCAAGAAGTTTTAGCACGGGAGACACACTGCGGGTGATAAGGTCCGTGGTGGAAAGGGAAACAGCCCAGATCGCCAGCTAAGGTCCCAAAATCACAGTTAAGTGGAAAACGATGT 1033

NZ_PPFR01000008.1:34378-38724(-) ATCCCGGCCTACCAAACCGAGGCAAACTCCGAATACCAAGAAGTTTTAGCACGGGAGACACACTGCGGGTGATAAGGTCCGTGGTGGAAAGGGAAACAGCCCAGATCGCCAGCTAAGGTCCCAAAATCACAGTTAAGTGGAAAACGATGT 1033

NZ_PPFQ01000003.1:131093-135446(+) ATCCCGGCCTACCAAACCGAGGCAAACTCCGAATACCAAGAAGTTTTAGCACGGGAGACACACTGCGGGTGATAAGGTCCGTGGTGGAAAGGGAAACAGCCCAGATCGCCAGCTAAGGTCCCAAAATCACAGTTAAGTGGAAAACGATGT 1033

NZ_LK937696.1:167613-171953(+) ATCCCGGCCTACCAAACCGAGGCAAACTCCGAATACCAAGAAGTTTTAGCACGGGAGACACACTGCGGGTGATAAGGTCCGTGGTGGAAAGGGAAACAGCCCAGATCGCCAGCTAAGGTCCCAAAATCACAGTTAAGTGGAAAACGATGT 1033

NZ_NOVI01000003.1:105965-110310(+) ATCCCGGCCTACCAAACCGAGGCAAACTCCGAATACCAAGAAGTTTTAGCACGGGAGACACACTGCGGGTGATAAGGTCCGTGGTGGAAAGGGAAACAGCCCAGATCGCCAGCTAAGGTCCCAAAATCACAGTTAAGTGGAAAACGATGT 1033

NZ_CP103426.1:167642-171981(+) ATCCCGGCCTACCAAACCGAGGCAAACTCCGAATACCAAGAAGTTTTAGCACGGGAGACACACTGCGGGTGATAAGGTCCGTGGTGGAAAGGGAAACAGCCCAGATCGCCAGCTAAGGTCCCAAAATCACAGTTAAGTGGAAAACGATGT 1033

NZ_JAOXDN010000007.1:36985-41324(-) ATCCCGGCCTACCAAACCGAGGCAAACTCCGAATACCAAGAAGTTTTAGCACGGGAGACACACTGCGGGTGATAAGGTCCGTGGTGGAAAGGGAAACAGCCCAGATCGCCAGCTAAGGTCCCAAAATCACAGTTAAGTGGAAAACGATGT 1033

NZ_JPVV01000013.1:13888-18051(+) ATCCCGGCCTACCAAACCGAGGCAAACTCCGAATACCAAGAAGTTTTAGCACGGGAGACACACTGCGGGTGATAAGGTCCGTGGTGGAAAGGGAAACAGCCCAGATCGCCAGCTAAGGTCCCAAAATCACAGTTAAGTGGAAAACGATGT 1033

NZ_CP007555.1:337406-341752(+) ATCCCGGCCTACCAAACCGAGGCAAACTCCGAATACCAAGAAGTTTTAGCACGGGAGACACACTGCGGGTGATAAGGTCCGTGGTGGAAAGGGAAACAGCCCAGATCGCCAGCTAAGGTCCCAAAATCACAGTTAAGTGGAAAACGATGT 1033

NZ_JASNNV010000010.1:39284-43631(-) ATCCCGGCCTACCAAACCGAGGCAAACTCCGAATACCAAGAAGTTTTAGCACGGGAGACACACTGCGGGTGATAAGGTCCGTGGTGGAAAGGGAAACAGCCCAGATCGCCAGCTAAGGTCCCAAAATCACAGTTAAGTGGAAAACGATGT 1033

NZ_JAOXFC010000012.1:14231-18577(+) ATCCCGGCCTACCAAACCGAGGCAAACTCCGAATACCAAGAAGTTTTAGCACGGGAGACACACTGCGGGTGATAAGGTCCGTGGTGGAAAGGGAAACAGCCCAGATCGCCAGCTAAGGTCCCAAAATCACAGTTAAGTGGAAAACGATGT 1033

NZ_CP103430.1:167658-171997(+) ATCCCGGCCTACCAAACCGAGGCAAACTCCGAATACCAAGAAGTTTTAGCACGGGAGACACACTGCGGGTGATAAGGTCCGTGGTGGAAAGGGAAACAGCCCAGATCGCCAGCTAAGGTCCCAAAATCACAGTTAAGTGGAAAACGATGT 1033

NZ_PDLP01000011.1:14441-18787(+) ATCCCGGCCTACCAAACCGAGGCAAACTCCGAATACCAAGAAGTTTTAGCACGGGAGACACACTGCGGGTGATAAGGTCCGTGGTGGAAAGGGAAACAGCCCAGATCGCCAGCTAAGGTCCCAAAATCACAGTTAAGTGGAAAACGATGT 1033

NZ_NOLM01000022.1:91220-95566(-) ATCCCGGCCTACCAAACCGAGGCAAACTCCGAATACCAAGAAGTTTTAGCACGGGAGACACACTGCGGGTGATAAGGTCCGTGGTGGAAAGGGAAACAGCCCAGATCGCCAGCTAAGGTCCCAAAATCACAGTTAAGTGGAAAACGATGT 1033

JBJCIS010000056.1:0-2780(-) ATCCCGGCCTACCAAACCGAGGCAAACTCCGAATACCAAGAAGTTTTAGCACGGGAGACACACTGCGGGTGATAAGGTCCGTGGTGGAAAGGGAAACAGCCCAGATCGCCAGCTAAGGTCCCAAAATCACAGTTAAGTGGAAAACGATGT 1033

NZ_CDBG01000001.1:179744-184090(+) ATCCCGGCCTACCAAACCGAGGCAAACTCCGAATACCAAGAAGTTTTAGCACGGGAGACACACTGCGGGTGATAAGGTCCGTGGTGGAAAGGGAAACAGCCCAGATCGCCAGCTAAGGTCCCAAAATCACAGTTAAGTGGAAAACGATGT 1033

NZ_CP103434.1:167622-171961(+) ATCCCGGCCTACCAAACCGAGGCAAACTCCGAATACCAAGAAGTTTTAGCACGGGAGACACACTGCGGGTGATAAGGTCCGTGGTGGAAAGGGAAACAGCCCAGATCGCCAGCTAAGGTCCCAAAATCACAGTTAAGTGGAAAACGATGT 1033

CP014354.1:76692-80603(-) ATCCCGGCCTACCAAACCGAGGCAAACTCCGAATACCAAGAAGTTTTAGCACGGGAGACACACTGCGGGTGATAAGGTCCGTGGTGGAAAGGGAAACAGCCCAGATCGCCAGCTAAGGTCCCAAAATCACAGTTAAGTGGAAAACGATGT 564

NOLR01000002.1:131833-136207(+) ATCCCGGCCTACCAAACCGAGGCAAACTCCGAATACCAAGAAGTTTTAGCACGGGAGACACACTGCGGGTGATAAGGTCCGTGGTGGAAAGGGAAACAGCCCAGATCGCCAGCTAAGGTCCCAAAATCACAGTTAAGTGGAAAACGATGT 1043

PPFR01000008.1:34360-38734(-) ATCCCGGCCTACCAAACCGAGGCAAACTCCGAATACCAAGAAGTTTTAGCACGGGAGACACACTGCGGGTGATAAGGTCCGTGGTGGAAAGGGAAACAGCCCAGATCGCCAGCTAAGGTCCCAAAATCACAGTTAAGTGGAAAACGATGT 1043

PPFQ01000003.1:131083-135464(+) ATCCCGGCCTACCAAACCGAGGCAAACTCCGAATACCAAGAAGTTTTAGCACGGGAGACACACTGCGGGTGATAAGGTCCGTGGTGGAAAGGGAAACAGCCCAGATCGCCAGCTAAGGTCCCAAAATCACAGTTAAGTGGAAAACGATGT 1043

NZ_AKYP01000181.1:1647-6020(-) ATCCCGGCCTACCAAACCGAGGCAAACTCCGAATACCAAGAAGTTTTAGCACGGGAGACACACTGCGGGTGATAAGGTCCGTGGTGGAAAGGGAAACAGCCCAGATCGCCAGCTAAGGTCCCAAAATCACAGTTAAGTGGAAAACGATGT 1043

NOVI01000003.1:105955-110328(+) ATCCCGGCCTACCAAACCGAGGCAAACTCCGAATACCAAGAAGTTTTAGCACGGGAGACACACTGCGGGTGATAAGGTCCGTGGTGGAAAGGGAAACAGCCCAGATCGCCAGCTAAGGTCCCAAAATCACAGTTAAGTGGAAAACGATGT 1043

CP018150.1:339294-343661(+) ATCCCGGCCTACCAAACCGAGGCAAACTCCGAATACCAAGAAGTTTTAGCACGGGAGACACACTGCGGGTGATAAGGTCCGTGGTGGAAAGGGAAACAGCCCAGATCGCCAGCTAAGGTCCCAAAATCACAGTTAAGTGGAAAACGATGT 1043

Consensus GGGAAGGCTCAGACAGCCAGGAGGTTGGCTTAGAAGCAGCCATCCTTTAAAGAAAGCGTAATAGCTCACTGGTCGAGTCGTCCTGCGCGGAAGATTTAACGGGGCTCAAACTGTGTACCGAAGCTGCGGCATCAGAAGACACTGTCTTCT 1193

JAKFBC010000002.1:34496-38842(-) (45) GGGAAGGCTCAGACAGCCAGGAGGTTGGCTTAGAAGCAGCCATCCTTTAAAGAAAGCGTAATAGCTCACTGGTCGAGTCGTCCTGCGCGGAAGATTTAACGGGGCTCAAACTGTGTACCGAAGCTGCGGCATCAGAAGACACTGTCTTCT 1183

NZ_AP019757.1:1739264-1743610(-) (31) GGGAAGGCTCAGACAGCCAGGAGGTTGGCTTAGAAGCAGCCATCCTTTAAAGAAAGCGTAATAGCTCACTGGTCGAGTCGTCCTGCGCGGAAGATTTAACGGGGCTCAAACTGTGTACCGAAGCTGCGGCATCAGAAGACACTGTCTTCT 1183

JBKOHN010000001.1:81530-85875(+) (25) GGGAAGGCTCAGACAGCCAGGAGGTTGGCTTAGAAGCAGCCATCCTTTAAAGAAAGCGTAATAGCTCACTGGTCGAGTCGTCCTGCGCGGAAGATTTAACGGGGCTCAAACTGTGTACCGAAGCTGCGGCATCAGAAGACACTGTCTTCT 1183

NC_011528.1:339307-343646(+) (14) GGGAAGGCTCAGACAGCCAGGAGGTTGGCTTAGAAGCAGCCATCCTTTAAAGAAAGCGTAATAGCTCACTGGTCGAGTCGTCCTGCGCGGAAGATTTAACGGGGCTCAAACTGTGTACCGAAGCTGCGGCATCAGAAGACACTGTCTTCT 1183

CP018005.1:167566-171940(+) (14) GGGAAGGCTCAGACAGCCAGGAGGTTGGCTTAGAAGCAGCCATCCTTTAAAGAAAGCGTAATAGCTCACTGGTCGAGTCGTCCTGCGCGGAAGATTTAACGGGGCTCAAACTGTGTACCGAAGCTGCGGCATCAGAAGACACTGTCTTCT 1193

NZ_CCAM010000005.1:71418-75764(+) (13) GGGAAGGCTCAGACAACCAGGAGGTTGGCTTAGAAGCAGCCATCCTTTAAAGAAAGCGTAATAGCTCACTGGTCGAGTCGTCCTGCGCGGAAGATTTAACGGGGCTCAAACTGTGTACCGAAGCTGCGGCATCAGAAGACACTGTCTTCT 1183

NZ_CCXO01000001.1:317226-321572(+) (11) GGGAAGGCTCAGACAGCCAGGAGGTTGGCTTAGAAGCAGCCATCCTTTAAAGAAAGCGTAATAGCTCACTGGTCGAGTCGTCCTGCGCGGAAGATTTAACGGGGCTCAAACTGTGTACCGAAGCTGCGGCATCAGAAGACACTGTCTTCT 1183

NC_011527.1:1751498-1755844(-) (8) GGGAAGGCTCAGACAGCCAGGAGGTTGGCTTAGAAGCAGCCATCCTTTAAAGAAAGCGTAATAGCTCACTGGTCGAGTCGTCCTGCGCGGAAGATTTAACGGGGCTCAAACTGTGTACCGAAGCTGCGGCATCAGAAGACACTGTCTTCT 1183

NZ_CP032542.1:88036-92382(-) (8) GGGAAGGCTCAGACAACCAGGAGGTTGGCTTAGAAGCAGCCATCCTTTAAAGAAAGCGTAATAGCTCACTGGTCGAGTCGTCCTGCGCGGAAGATTTAACGGGGCTCAAACTGTGTACCGAAGCTGCGGCATCAGAAGACACTGTCTTCT 1183

CP014551.1:161236-165610(+) (8) GGGAAGGCTCAGACAGCCAGGAGGTTGGCTTAGAAGCAGCCATCCTTTAAAGAAAGCGTAATAGCTCACTGGTCGAGTCGTCCTGCGCGGAAGATTTAACGGGGCTCAAACTGTGTACCGAAGCTGCGGCATCAGAAGACACTGTCTTCT 1193

CP013667.1:88011-92385(-) (8) GGGAAGGCTCAGACAACCAGGAGGTTGGCTTAGAAGCAGCCATCCTTTAAAGAAAGCGTAATAGCTCACTGGTCGAGTCGTCCTGCGCGGAAGATTTAACGGGGCTCAAACTGTGTACCGAAGCTGCGGCATCAGAAGACACTGTCTTCT 1193

CP107247.1:164350-168689(+) (7) GGGAAGGCTCAGACAGCCAGGAGGTTGGCTTAGAAGCAGCCATCCTTTAAAGAAAGCGTAATAGCTCACTGGTCGAGTCGTCCTGCGCGGAAGATTTAACGGGGCTCAAACTGTGTACCGAAGCTGCGGCATCAGAAGACACTGTCTTCT 1183

NZ_CP103435.1:167581-171920(+) (6) GGGAAGGCTCAGACAGCCAGGAGGTTGGCTTAGAAGCAGCCATCCTTTAAAGAAAGCGTAATAGCTCACTGGTCGAGTCGTCCTGCGCGGAAGATTTAACGGGGCTCAAACTGTGTACCGAAGCTGCGGCATCAGAAGACACTGTCTTCT 1183

CP014565.1:1711747-1716121(-) (3) GGGAAGGCTCAGACAGCCAGGAGGTTGGCTTAGAAGCAGCCATCCTTTAAAGAAAGCGTAATAGCTCACTGGTCGAGTCGTCCTGCGCGGAAGATTTAACGGGGCTCAAACTGTGTACCGAAGCTGCGGCATCAGAAGACACTGTCTTCT 1193

AAYJ01000007.1:0-1404(+) (3) ------------------------------------------------------------------------------------------------------------------------------------------------------

NZ_JAOXDR010000002.1:16672-21025(-) (3) GGGAAGGCTCAGACAACCAGGAGGTTGGCTTAGAAGCAGCCATCCTTTAAAGAAAGCGTAATAGCTCACTGGTCGAGTCGTCCTGCGCGGAAGATTTAACGGGGCTCAAACTGTGTACCGAAGCTGCGGCATCAGAAGACACTGTCTTCT 1183

NZ_CP103432.1:167699-172038(+) (3) GGGAAGGCTCAGACAACCAGGAGGTTGGCTTAGAAGCAGCCATCCTTTAAAGAAAGCGTAATAGCTCACTGGTCGAGTCGTCCTGCGCGGAAGATTTAACGGGGCTCAAACTGTGTACCGAAGCTGCGGCATCAGAAGACACTGTCTTCT 1183

NZ_CP103431.1:167697-172036(+) (3) GGGAAGGCTCAGACAGCCAGGAGGTTGGCTTAGAAGCAGCCATCCTTTAAAGAAAGCGTAATAGCTCACTGGTCGAGTCGTCCTGCGCGGAAGATTTAACGGGGCTCAAACTGTGTACCGAAGCTGCGGCATCAGAAGACACTGTCTTCT 1183

NZ_CP103428.1:167591-171929(+) (2) GGGAAGGCTCAGACAGCCAGGAGGTTGGCTTAGAAGCAGCCATCCTTTAAAGAAAGCGTAATAGCTCACTGGTCGAGTCGTCCTGCGCGGAAGATTTAACGGGGCTCAAACTGTGTACCGAAGCTGCGGCATCAGAAGACACTGTCTTCT 1183

NZ_JANTNR010000002.1:115350-119696(+) (2) GGGAAGGCTCAGACAGCCAGGAGGTTGGCTTAGAAGCAGCCATCCTTTAAAGAAAGCGTAATAGCTCACTGGTCGAGTCGTCCTGCGCGGAAGATTTAACGGGGCTCAAACTGTGTACCGAAGCTGCGGCATCAGAAGACACTGTCTTCT 1183

JARBIR010004564.1:3782-8128(-) (2) GGGAAGGCTCAGACAGCCAGGAGGTTGGCTTAGAAGCAGCCATCCTTTAAAGAAAGCGTAATAGCTCACTGGTCGAGTCGTCCTGCGCGGAAGATTTAACGGGGCTCAAACTGTGTACCGAAGCTGCGGCATCAGAAGACACTGTCTTCT 1183

NC_009727.1:1888349-1892695(-) (2) GGGAAGGCTCAGACAGCCAGGAGGTTGGCTTAGAAGCAGCCATCCTTTAAAGAAAGCGTAATAGCTCACTGGTCGAGTCGTCCTGCGCGGAAGATTTAACGGGGCTCAAACTGTGTACCGAAGCTGCGGCATCAGAAGACACTGTCTTCT 1183

AAYJ01000139.1:0-660(+) (2) ---------------------------------------------------------------------------------------------------------------------------------------------CTGTCTTCT 9

CP032542.1:88018-92392(-) (2) GGGAAGGCTCAGACAACCAGGAGGTTGGCTTAGAAGCAGCCATCCTTTAAAGAAAGCGTAATAGCTCACTGGTCGAGTCGTCCTGCGCGGAAGATTTAACGGGGCTCAAACTGTGTACCGAAGCTGCGGCATCAGAAGACACTGTCTTCT 1193

CP014559.1:232744-237117(+) (2) GGGAAGGCTCAGACAGCCAGGAGGTTGGCTTAGAAGCAGCCATCCTTTAAAGAAAGCGTAATAGCTCACTGGTCGAGTCGTCCTGCGCGGAAGATTTAACGGGGCTCAAACTGTGTACCGAAGCTGCGGCATCAGAAGACACTGTCTTCT 1193

CP007555.1:337408-341752(+) GGGAAGGCTCAGACAGCCAGGAGGTTGGCTTAGAAGCAGCCATCCTTTAAAGAAAGCGTAATAGCTCACTGGTCGAGTCGTCCTGCGCGGAAGATTTAACGGGGCTCAAACTGTGTACCGAAGCTGCGGCATCAGAAGACACTGTCTTCT 1181

NZ_CP014354.1:76710-81178(-) GGGAAGGCTCAGACAACCAGGAGGTTGGCTTAGAAGCAGCCATCCTTTAAAGAAAGCGTAATAGCTCACTGGTCGAGTCGTCCTGCGCGGAAGATTTAACGGGGCTCAAACTGTGTACCGAAGCTGCGGCATCAGAAGACACTGTCTTCT 1189

CP000890.1:243129-244433(+) ---------------------------------------------------------------------------------------------------------------------------------------------CTGTCTTCT 9

NZ_CP115461.1:167576-171922(+) GGGAAGGCTTAGACAGCCAGGATGTTGGCTTAGAAGCAGCCACCATTTAAAGAAAGCGTAATAGCTCACTGGTCGAGTCGGCCTGCGCGGAAGATTTAACGGGGCTCAAACTGTGTACCGAAGCTGCGGCATCAGAAGACACTGTCTTCT 1183

AP019759.1:167578-171919(+) GGGAAGGCTCAGACAGCCAGGAGGTTGGCTTAGAAGCAGCCATCCTTTAAAGAAAGCGTAATAGCTCACTGGTCGAGTCGTCCTGCGCGGAAGATTTAACGGGGCTCAAACTGTGTACCGAAGCTGCGGCATCAGAAGACACTGTCTTCT 1181

AP019757.1:1739267-1743608(-) GGGAAGGCTCAGACAGCCAGGAGGTTGGCTTAGAAGCAGCCATCCTTTAAAGAAAGCGTAATAGCTCACTGGTCGAGTCGTCCTGCGCGGAAGATTTAACGGGGCTCAAACTGTGTACCGAAGCTGCGGCATCAGAAGACACTGTCTTCT 1181

NOLN01000021.1:90861-95235(-) GGGAAGGCTCAGACAGCCAGGAGGTTGGCTTAGAAGCAGCCATCCTTTAAAGAAAGCGTAATAGCTCACTGGTCGAGTCGTCCTGCGCGGAAGATTTAACGGGGCTCAAACTGTGTACCGAAGCTGCGGCATCAGAAGACACTGTCTTCT 1193

NOLM01000022.1:91202-95576(-) GGGAAGGCTCAGACAGCCAGGAGGTTGGCTTAGAAGCAGCCATCCTTTAAAGAAAGCGTAATAGCTCACTGGTCGAGTCGTCCTGCGCGGAAGATTTAACGGGGCTCAAACTGTGTACCGAAGCTGCGGCATCAGAAGACACTGTCTTCT 1193

PDLP01000011.1:14431-18805(+) GGGAAGGCTCAGACAGCCAGGAGGTTGGCTTAGAAGCAGCCATCCTTTAAAGAAAGCGTAATAGCTCACTGGTCGAGTCGTCCTGCGCGGAAGATTTAACGGGGCTCAAACTGTGTACCGAAGCTGCGGCATCAGAAGACACTGTCTTCT 1193

CP014563.1:522940-527314(+) GGGAAGGCTCAGACAGCCAGGAGGTTGGCTTAGAAGCAGCCATCCTTTAAAGAAAGCGTAATAGCTCACTGGTCGAGTCGTCCTGCGCGGAAGATTTAACGGGGCTCAAACTGTGTACCGAAGCTGCGGCATCAGAAGACACTGTCTTCT 1193

CP000890.1:244922-245905(+) ------------------------------------------------------------------------------------------------------------------------------------------------------

NZ_NOLR01000002.1:131843-136189(+) GGGAAGGCTCAGACAGCCAGGAGGTTGGCTTAGAAGCAGCCATCCTTTAAAGAAAGCGTAATAGCTCACTGGTCGAGTCGTCCTGCGCGGAAGATTTAACGGGGCTCAAACTGTGTACCGAAGCTGCGGCATCAGAAGACACTGTCTTCT 1183

NZ_JAOXDP010000002.1:15794-20139(-) GGGAAGGCTCAGACAACCAGGAGGTTGGCTTAGAAGCAGCCATCCTTTAAAGAAAGCGTAATAGCTCACTGGTCGAGTCGTCCTGCGCGGAAGATTTAACGGGGCTCAAACTGTGTACCGAAGCTGCGGCATCAGAAGACACTGTCTTCT 1183

NZ_PPFR01000008.1:34378-38724(-) GGGAAGGCTCAGACAGCCAGGAGGTTGGCTTAGAAGCAGCCATCCTTTAAAGAAAGCGTAATAGCTCACTGGTCGAGTCGTCCTGCGCGGAAGATTTAACGGGGCTCAAACTGTGTACCGAAGCTGCGGCATCAGAAGACACTGTCTTCT 1183

NZ_PPFQ01000003.1:131093-135446(+) GGGAAGGCTCAGACAGCCAGGAGGTTGGCTTAGAAGCAGCCATCCTTTAAAGAAAGCGTAATAGCTCACTGGTCGAGTCGTCCTGCGCGGAAGATTTAACGGGGCTCAAACTGTGTACCGAAGCTGCGGCATCAGAAGACACTGTCTTCT 1183

NZ_LK937696.1:167613-171953(+) GGGAAGGCTCAGACAACCAGGAGGTTGGCTTAGAAGCAGCCATCCTTTAAAGAAAGCGTAATAGCTCACTGGTCGAGTCGTCCTGCGCGGAAGATTTAACGGGGCTCAAACTGTGTACCGAAGCTGCGGCATCAGAAGACACTGTCTTCT 1183

NZ_NOVI01000003.1:105965-110310(+) GGGAAGGCTCAGACAGCCAGGAGGTTGGCTTAGAAGCAGCCATCCTTTAAAGAAAGCGTAATAGCTCACTGGTCGAGTCGTCCTGCGCGGAAGATTTAACGGGGCTCAAACTGTGTACCGAAGCTGCGGCATCAGAAGACACTGTCTTCT 1183

NZ_CP103426.1:167642-171981(+) GGGAAGGCTCAGACAACCAGGAGGTTGGCTTAGAAGCAGCCATCCTTTAAAGAAAGCGTAATAGCTCACTGGTCGAGTCGTCCTGCGCGGAAGATTTAACGGGGCTCAAACTGTGTACCGAAGCTGCGGCATCAGAAGACACTGTCTTCT 1183

NZ_JAOXDN010000007.1:36985-41324(-) GGGAAGGCTCAGACAGCCAGGAGGTTGGCTTAGAAGCAGCCATCCTTTAAAGAAAGCGTAATAGCTCACTGGTCGAGTCGTCCTGCGCGGAAGATTTAACGGGGCTCAAACTGTGTACCGAAGCTGCGGCATCAGAAGACACTGTCTTCT 1183

NZ_JPVV01000013.1:13888-18051(+) GGGAAGGCTCAGACAGCCAGGAGGTTGGCTTAGAAGCAGCCATCCTTTAAAGAAAGCGTAATAGCTCACTGGTCGAGTCGTCCTGCGCGGAAGATTTAACGGGGCTCAAACTGTGTACCGAAGCTGCGGCATCAGAAGACACTGTCTTCT 1183

NZ_CP007555.1:337406-341752(+) GGGAAGGCTCAGACAGCCAGGAGGTTGGCTTAGAAGCAGCCATCCTTTAAAGAAAGCGTAATAGCTCACTGGTCGAGTCGTCCTGCGCGGAAGATTTAACGGGGCTCAAACTGTGTACCGAAGCTGCGGCATCAGAAGACACTGTCTTCT 1183

NZ_JASNNV010000010.1:39284-43631(-) GGGAAGGCTCAGACAGCCAGGAGGTTGGCTTAGAAGCAGCCATCCTTTAAAGAAAGCGTAATAGCTCACTGGTCGAGTCGTCCTGCGCGGAAGATTTAACGGGGCTCAAACTGTGTACCGAAGCTGCGGCATCAGAAGACACTGTCTTCT 1183

NZ_JAOXFC010000012.1:14231-18577(+) GGGAAGGCTCAGACAGCCAGGAGGTTGGCTTAGAAGCAGCCATCCTTTAAAGAAAGCGTAATAGCTCACTGGTCGAGTCGTCCTGCGCGGAAGATTTAACGGGGCTCAAACTGTGTACCGAAGCTGCGGCATCAGAAGACACTGTCTTCT 1183

NZ_CP103430.1:167658-171997(+) GGGAAGGCTCAGACAGCCAGGAGGTTGGCTTAGAAGCAGCCATCCTTTAAAGAAAGCGTAATAGCTCACTGGTCGAGTCGTCCTGCGCGGAAGATTTAACGGGGCTCAAACTGTGTACCGAAGCTGCGGCATCAGAAGACACTGTCTTCT 1183

NZ_PDLP01000011.1:14441-18787(+) GGGAAGGCTCAGACAGCCAGGAGGTTGGCTTAGAAGCAGCCATCCTTTAAAGAAAGCGTAATAGCTCACTGGTCGAGTCGTCCTGCGCGGAAGATTTAACGGGGCTCAAACTGTGTACCGAAGCTGCGGCATCAGAAGACACTGTCTTCT 1183

NZ_NOLM01000022.1:91220-95566(-) GGGAAGGCTCAGACAGCCAGGAGGTTGGCTTAGAAGCAGCCATCCTTTAAAGAAAGCGTAATAGCTCACTGGTCGAGTCGTCCTGCGCGGAAGATTTAACGGGGCTCAAACTGTGTACCGAAGCTGCGGCATCAGAAGACACTGTCTTCT 1183

JBJCIS010000056.1:0-2780(-) GGGAAGGCTCAGACAGCCAGGAGGTTGGCTTAGAAGCAGCCATCCTTTAAAGAAAGCGTAATAGCTCACTGGTCGAGTCGTCCTGCGCGGAAGATTTAACGGGGCTCAAACTGTGTACCGAAGCTGCGGCATCAGAAGACACTGTCTTCT 1183

NZ_CDBG01000001.1:179744-184090(+) GGGAAGGCTCAGACARCCAGGAGGTTGGCTTAGAAGCAGCCATCCTTTAAAGAAAGCGTAATAGCTCACTGGTCGAGTCGTCCTGCGCGGAAGATTTAACGGGGCTCAAACTGTGTACCGAAGCTGCGGCATCAGAAGACACTGTCTTCT 1183

NZ_CP103434.1:167622-171961(+) GGGAAGGCTCAGACAACCAGGAGGTTGGCTTAGAAGCAGCCATCCTTTAAAGAAAGCGTAATAGCTCACTGGTCGAGTCGTCCTGCGCGGAAGATTTAACGGGGCTCAAACTGTGTACCGAAGCTGCGGCATCAGAAGACACTGTCTTCT 1183

CP014354.1:76692-80603(-) GGGAAGGCTCAGACAACCAGGAGGTTGGCTTAGAAGCAGCCATCCTTTAAAGAAAGCGTAATAGCTCACTGGTCGAGTCGTCCTGCGCGGAAGATTTAACGGGGCTCAAACTGTGTACCGAAGCTGCGGCATCAGAAGACACTGTCTTCT 714

NOLR01000002.1:131833-136207(+) GGGAAGGCTCAGACAGCCAGGAGGTTGGCTTAGAAGCAGCCATCCTTTAAAGAAAGCGTAATAGCTCACTGGTCGAGTCGTCCTGCGCGGAAGATTTAACGGGGCTCAAACTGTGTACCGAAGCTGCGGCATCAGAAGACACTGTCTTCT 1193

PPFR01000008.1:34360-38734(-) GGGAAGGCTCAGACAGCCAGGAGGTTGGCTTAGAAGCAGCCATCCTTTAAAGAAAGCGTAATAGCTCACTGGTCGAGTCGTCCTGCGCGGAAGATTTAACGGGGCTCAAACTGTGTACCGAAGCTGCGGCATCAGAAGACACTGTCTTCT 1193

PPFQ01000003.1:131083-135464(+) GGGAAGGCTCAGACAGCCAGGAGGTTGGCTTAGAAGCAGCCATCCTTTAAAGAAAGCGTAATAGCTCACTGGTCGAGTCGTCCTGCGCGGAAGATTTAACGGGGCTCAAACTGTGTACCGAAGCTGCGGCATCAGAAGACACTGTCTTCT 1193

NZ_AKYP01000181.1:1647-6020(-) GGGAAGGCTCAGACAACCAGGAGGTTGGCTTAGAAGCAGCCATCCTTTAAAGAAAGCGTAATAGCTCACTGGTCGAGTCGTCCTGCGCGGAAGATTTAACGGGGCTCAAACTGTGTACCGAAGCTGCGGCATCAGAAGACACTGTCTTCT 1193

NOVI01000003.1:105955-110328(+) GGGAAGGCTCAGACAGCCAGGAGGTTGGCTTAGAAGCAGCCATCCTTTAAAGAAAGCGTAATAGCTCACTGGTCGAGTCGTCCTGCGCGGAAGATTTAACGGGGCTCAAACTGTGTACCGAAGCTGCGGCATCAGAAGACACTGTCTTCT 1193

CP018150.1:339294-343661(+) GGGAAGGCTCAGACAGCCAGGAGGTTGGCTTAGAAGCAGCCATCCTTTAAAGAAAGCGTAATAGCTCACTGGTCGAGTCGTCCTGCGCGGAAGATTTAACGGGGCTCAAACTGTGTACCGAAGCTGCGGCATCAGAAGACACTGTCTTCT 1193

Consensus GATGGGTAGAGGAGCGTTCTGTAAGCCTGTGAAGGTGAATCGAGAGGTTTGCTGGAGGTATCAGAAGTGCGAATGCTGACATAAGTAACGATAATGTGGGTGAAAAACCCACACGCCGAAAGTCTAAGGTTTCCTGCGCAACGTTAATCG 1343

JAKFBC010000002.1:34496-38842(-) (45) GATGGGTAGAGGAGCGTTCTGTAAGCCTGTGAAGGTGAATCGAGAGGTTTGCTGGAGGTATCAGAAGTGCGAATGCTGACATAAGTAACGATAATGTGGGTGAAAAACCCACACGCCGAAAGTCTAAGGTTTCCTGCGCAACGTTAATCG 1333

NZ_AP019757.1:1739264-1743610(-) (31) GATGGGTAGAGGAGCGTTCTGTAAGCCTGTGAAGGTGAATCGAGAGGTTTGCTGGAGGTATCAGAAGTGCGAATGCTGACATAAGTAACGATAATGTGGGTGAAAAACCCACACGCCAAAAGTCTAAGGTTTCCTGCGCAACGTTAATCG 1333

JBKOHN010000001.1:81530-85875(+) (25) GATGGGTAGAGGAGCGTTCTGTAAGCCTGTGAAGGTGAATCGAGAGGTTTGCTGGAGGTATCAGAAGTGCGAATGCTGACATAAGTAACGATAATGTGGGTGAAAAACCCACACGCCGAAAGTCTAAGGTTTCCTGCGCAACGTTAATCG 1333

NC_011528.1:339307-343646(+) (14) GATGGGTAGAGGAGCGTTCTGTAAGCCTGTGAAGGTGAATCGAGAGGTTTGCTGGAGGTATCAGAAGTGCGAATGCTGACATAAGTAACGATAATGTGGGTGAAAAACCCACACGCCGAAAGTCTAAGGTTTCCTGCGCAACGTTAATCG 1333

CP018005.1:167566-171940(+) (14) GATGGGTAGAGGAGCGTTCTGTAAGCCTGTGAAGGTGAATCGAGAGGTTTGCTGGAGGTATCAGAAGTGCGAATGCTGACATAAGTAACGATAATGTGGGTGAAAAACCCACACGCCGAAAGTCTAAGGTTTCCTGCGCAACGTTAATCG 1343

NZ_CCAM010000005.1:71418-75764(+) (13) GATGGGTAGAGGAGCGTTCTGTAAGCCTGTGAAGGTGAATCGAGAGGTTTGCTGGAGGTATCAGAAGTGCGAATGCTGACATAAGTAACGATAATGTGGGTGAAAAACCCACACGCCGAAAGTCTAAGGTTTCCTGCGCAACGTTAATCG 1333

NZ_CCXO01000001.1:317226-321572(+) (11) GATGGGTAGAGGAGCGTTCTGTAAGCCTGTGAAGGTGAATCGAGAGGTTTGCTGGAGGTATCAGAAGTGCGAATGCTGACATAAGTAACGATAATGTGGGTGAAAAACCCACACGCCGAAAGTCTAAGGTTTCCTGCGCAACGTTAATCG 1333

NC_011527.1:1751498-1755844(-) (8) GATGGGTAGAGGAGCGTTCTGTAAGCCTGTGAAGGTGAATCGAGAGGTTTGCTGGAGGCATCAGAAGTGCGAATGCTGACATAAGTAACGATAATGTGGGTGAAAAACCCACACGCCGAAAGTCTAAGGTTTCCTGCGCAACGTTAATCG 1333

NZ_CP032542.1:88036-92382(-) (8) GATGGGTAGAGGAGCGTTCTGTAAGCCTGTGAAGGTGAATCGAGAGGTTTGCTGGAGGTATCAGAAGTGCGAATGCTGACATAAGTAACGATAATGTGGGTGAAAAACCCACACGCCGAAAGTCTAAGGTTTCCTGCGCAACGTTAATCG 1333

CP014551.1:161236-165610(+) (8) GATGGGTAGAGGAGCGTTCTGTAAGCCTGTGAAGGTGAATCGAGAGGTTTGCTGGAGGTATCAGAAGTGCGAATGCTGACATAAGTAACGATAATGTGGGTGAAAAACCCACACGCCAAAAGTCTAAGGTTTCCTGCGCAACGTTAATCG 1343

CP013667.1:88011-92385(-) (8) GATGGGTAGAGGAGCGTTCTGTAAGCCTGTGAAGGTGAATCGAGAGGTTTGCTGGAGGTATCAGAAGTGCGAATGCTGACATAAGTAACGATAATGTGGGTGAAAAACCCACACGCCGAAAGTCTAAGGTTTCCTGCGCAACGTTAATCG 1343

CP107247.1:164350-168689(+) (7) GATGGGTAGAGGAGCGTTCTGTAAGCCTGTGAAGGTGAATCGAGAGGTTTGCTGGAGGTATCAGAAGTGCGAATGCTGACATAAGTAACGATAATGTGGGTGAAAAACCCACACGCCGAAAGTCTAAGGTTTCCTGCGCAACGTTAATCG 1333

NZ_CP103435.1:167581-171920(+) (6) GATGGGTAGAGGAGCGTTCTGTAAGCCTGTGAAGGTGAATCGAGAGGTTTGCTGGAGGTATCAGAAGTGCGAATGCTGACATAAGTAACGATAATGTGGGTGAAAAACCCACACGCCGAAAGTCTAAGGTTTCCTGCGCAACGTTAATCG 1333

CP014565.1:1711747-1716121(-) (3) GATGGGTAGAGGAGCGTTCTGTAAGCCTGTGAAGGTGAATCGAGAGGTTTGCTGGAGGCATCAGAAGTGCGAATGCTGACATAAGTAACGATAATGTGGGTGAAAAACCCACACGCCGAAAGTCTAAGGTTTCCTGCGCAACGTTAATCG 1343

AAYJ01000007.1:0-1404(+) (3) ------------------------------------------------------------------------------------------------------------------------------------------------------

NZ_JAOXDR010000002.1:16672-21025(-) (3) GATGGGTAGAGGAGCGTTCTGTAAGCCTGTGAAGGTGAATCGAGAGGTTTGCTGGAGGTATCAGAAGTGCGAATGCTGACATAAGTAACGATAATGTGGGTGAAAAACCCACACGCCGAAAGTCTAAGGTTTCCTGCGCAACGTTAATCG 1333

NZ_CP103432.1:167699-172038(+) (3) GATGGGTAGAGGAGCGTTCTGTAAGCCTGTGAAGGTGAATCGAGAGGTTTGCTGGAGGTATCAGAAGTGCGAATGCTGACATAAGTAACGATAATGTGGGTGAAAAACCCACACGCCGAAAGTCTAAGGTTTCCTGCGCAACGTTAATCG 1333

NZ_CP103431.1:167697-172036(+) (3) GATGGGTAGAGGAGCGTTCTGTAAGCCTGTGAAGGTGAATCGAGAGGTTTGCTGGAGGTATCAGAAGTGCGAATGCTGACATAAGTAACGATAATGTGGGTGAAAAACCCACACGCCGAAAGTCTAAGGTTTCCTGCGCAACGTTAATCG 1333

NZ_CP103428.1:167591-171929(+) (2) GATGGGTAGAGGAGCGTTCTGTAAGCCTGTGAAGGTGAATCGAGAGGTTTGCTGGAGGTATCAGAAGTGCGAATGCTGACATAAGTAACGATAATGTGGGTGAAAAACCCACACGCCGAAAGTCTAAGGTTTCCTGCGCAACGTTAATCG 1333

NZ_JANTNR010000002.1:115350-119696(+) (2) GATGGGTAGAGGAGCGTTCTGTAAGCCTGTGAAGGTGAATCGAGAGGTTTGCTGGAGGTATCAGAAGTGCGAATGCTGACATAAGTAACGATAATGTGGGTGAAAAACCCACACGCCGAAAGTCTAAGGTTTCCTGCGCAACGTTAATCG 1333

JARBIR010004564.1:3782-8128(-) (2) GATGGGTAGAGGAGCGTTCTGTAAGCCTGTGAAGGTGAATCGAGAGGTTTGCTGGAGGTATCAGAAGTGCGAATGCTGACATAAGTAACGATAATGTGGGTGAAAAACCCACACGCCGAAAGTCTAAGGTTTCCTGCGCAACGTTAATCG 1333

NC_009727.1:1888349-1892695(-) (2) GATGGGTAGAGGAGCGTTCTGTAAGCCTGTGAAGGTGAATCGAGAGGTTTGCTGGAGGTATCAGAAGTGCGAATGCTGACATAAGTAACGATAATGTGGGTGAAAAACCCACACGCCGAAAGTCTAAGGTTTCCTGCGCAACGTTAATCG 1333

AAYJ01000139.1:0-660(+) (2) GATGGGTAGAGGAGCGTTCTGTAAGCCTGTGAAGGTGAATCGAGAGGTTTGCTGGAGGTATCAGAAGTGCGAATGCTGACATAAGTAACGATAATGTGGGTGAAAAACCCACACGCCGAAAGTCTAAGGTTTCCTGCGCAACGTTAATCG 159

CP032542.1:88018-92392(-) (2) GATGGGTAGAGGAGCGTTCTGTAAGCCTGTGAAGGTGAATCGAGAGGTTTGCTGGAGGTATCAGAAGTGCGAATGCTGACATAAGTAACGATAATGTGGGTGAAAAACCCACACGCCGAAAGTCTAAGGTTTCCTGCGCAACGTTAATCG 1343

CP014559.1:232744-237117(+) (2) GATGGGTAGAGGAGCGTTCTGTAAGCCTGTGAAGGTGAATCGAGAGGTTTGCTGGAGGTATCAGAAGTGCGAATGCTGACATAAGTAACGATAATGTGGGTGAAAAACCCACACGCCGAAAGTCTAAGGTTTCCTGCGCAACGTTAATCG 1343

CP007555.1:337408-341752(+) GATGGGTAGAGGAGCGTTCTGTAAGCCTGTGAAGGTGAATCGAGAGGTTTGCTGGAGGTATCAGAAGTGCGAATGCTGACATAAGTAACGATAATGTGGGTGAAAAACCCACACGCCGAAAGTCTAAGGTTTCCTGCGCAACGTTAATCG 1331

NZ_CP014354.1:76710-81178(-) GATGGGTAGAGGAGCGTTCTGTAAGCCTGTGAAGGTGAATCGAGAGGTTTGCTGGAGGTATCAGAAGTGCGAATGCTGACATAAGTAACGATAATGTGGGTGAAAAACCCACACGCCGAAAGTCTAAGGTTTCCTGCGCAACGTTAATCG 1339

CP000890.1:243129-244433(+) GATGGGTAGAGGAGCGTTCTGTAAGCCTGTGAAGGTGAATCGAGAGGTTTGCTGGAGGTATCAGAAGTGCGAATGCTGACATAAGTAACGATAATGTGGGTGAAAAACCCACACGCCGAAAGTCTAAGGTTTCCTGCGCAACGTTAATCG 159

NZ_CP115461.1:167576-171922(+) GATGGGTAGAGGAGCGTTCTGTAAGCCTGTGAAGGTGAATCGAGAGGTTTGCTGGAGGTATCAGAAGTGCGAATGCTGACATAAGTAACGATAATGCGGGTGAAAAACCCACACGCCGAAAGTCTAAGGTTTCCTGCGCAACGTTAATCG 1333

AP019759.1:167578-171919(+) GATGGGTAGAGGAGCGTTCTGTAAGCCTGTGAAGGTGAATCGAGAGGTTTGCTGGAGGTATCAGAAGTGCGAATGCTGACATAAGTAACGATAATGTGGGTGAAAAACCCACACGCCGAAAGTCTAAGGTTTCCTGCGCAACGTTAATCG 1331

AP019757.1:1739267-1743608(-) GATGGGTAGAGGAGCGTTCTGTAAGCCTGTGAAGGTGAATCGAGAGGTTTGCTGGAGGTATCAGAAGTGCGAATGCTGACATAAGTAACGATAATGTGGGTGAAAAACCCACACGCCAAAAGTCTAAGGTTTCCTGCGCAACGTTAATCG 1331

NOLN01000021.1:90861-95235(-) GATGGGTAGAGGAGCGTTCTGTAAGCCTGTGAAGGTGAATCGAGAGGTTTGCTGGAGGTATCAGAAGTGCGAATGCTGACATAAGTAACGATAATGTGGGTGAAAAACCCACACGCCGAAAGTCTAAGGTTTCCTGCGCAACGTTAATCG 1343

NOLM01000022.1:91202-95576(-) GATGGGTAGAGGAGCGTTCTGTAAGCCTGTGAAGGTGAATCGAGAGGTTTGCTGGAGGTATCAGAAGTGCGAATGCTGACATAAGTAACGATAATGTGGGTGAAAAACCCACACGCCGAAAGTCTAAGGTTTCCTGCGCAACGTTAATCG 1343

PDLP01000011.1:14431-18805(+) GATGGGTAGAGGAGCGTTCTGTAAGCCTGTGAAGGTGAATCGAGAGGTTTGCTGGAGGTATCAGAAGTGCGAATGCTGACATAAGTAACGATAATGTGGGTGAAAAACCCACACGCCGAAAGTCTAAGGTTTCCTGCGCAACGTTAATCG 1343

CP014563.1:522940-527314(+) GATGGGTAGAGGAGCGTTCTGTAAGCCTGTGAAGGTGAATCGAGAGGTTTGCTGGAGGTATCAGAAGTGCGAATGCTGACATAAGTAACGATAATGTGGGTGAAAAACCCACACGCCGAAAGTCTAAGGTTTCCTGCGCAACGTTAATCG 1343

CP000890.1:244922-245905(+) ------------------------------------------------------------------------------------------------------------------------------------------------------

NZ_NOLR01000002.1:131843-136189(+) GATGGGTAGAGGAGCGTTCTGTAAGCCTGTGAAGGTGAATCGAGAGGTTTGCTGGAGGTATCAGAAGTGCGAATGCTGACATAAGTAACGATAATGTGGGTGAAAAACCCACACGCCAAAAGTCTAAGGTTTCCTGCGCAACGTTAATCG 1333

NZ_JAOXDP010000002.1:15794-20139(-) GATGGGTAGAGGAGCGTTCTGTAAGCCTGTGAAGGTGAATCGAGAGGTTTGCTGGAGGTATCAGAAGTGCGAATGCTGACATAAGTAACGATAATGTGGGTGAAAAACCCACACGCCGAAAGTCTAAGGTTTCCTGCGCAACGTTAATCG 1333

NZ_PPFR01000008.1:34378-38724(-) GATGGGTAGAGGAGCGTTCTGTAAGCCTGTGAAGGTGAATCGAGAGGTTTGCTGGAGGTATCAGAAGTGCGAATGCTGACATAAGTAACGATAATGTGGGTGAAAAACCCACACGCCGAAAGTCTAAGGTTTCCTGCGCAACGTTAATCG 1333

NZ_PPFQ01000003.1:131093-135446(+) GATGGGTAGAGGAGCGTTCTGTAAGCCTGTGAAGGTGAATCGAGAGGTTTGCTGGAGGTATCAGAAGTGCGAATGCTGACATAAGTAACGATAATGTGGGTGAAAAACCCACACGCCAAAAGTCTAAGGTTTCCTGCGCAACGTTAATCG 1333

NZ_LK937696.1:167613-171953(+) GATGGGTAGAGGAGCGTTCTGTAAGCCTGTGAAGGTGAATCGAGAGGTTTGCTGGAGGTATCAGAAGTGCGAATGCTGACATAAGTAACGATAATGTGGGTGAAAAACCCACACGCCGAAAGTCTAAGGTTTCCTGCGCAACGTTAATCG 1333

NZ_NOVI01000003.1:105965-110310(+) GATGGGTAGAGGAGCGTTCTGTAAGCCTGTGAAGGTGAATCGAGAGGTTTGCTGGAGGTATCAGAAGTGCGAATGCTGACATAAGTAACGATAATGTGGGTGAAAAACCCACACGCCGAAAGTCTAAGGTTTCCTGCGCAACGTTAATCG 1333

NZ_CP103426.1:167642-171981(+) GATGGGTAGAGGAGCGTTCTGTAAGCCTGTGAAGGTGAATCGAGAGGTTTGCTGGAGGTATCAGAAGTGCGAATGCTGACATAAGTAACGATAATGTGGGTGAAAAACCCACACGCCGAAAGTCTAAGGTTTCCTGCGCAACGTTAATCG 1333

NZ_JAOXDN010000007.1:36985-41324(-) GATGGGTAGAGGAGCGTTCTGTAAGCCTGTGAAGGTGAATCGAGAGGTTTGCTGGAGGTATCAGAAGTGCGAATGCTGACATAAGTAACGATAATGTGGGTGAAAAACCCACACGCCGAAAGTCTAAGGTTTCCTGCGCAACGTTAATCG 1333

NZ_JPVV01000013.1:13888-18051(+) GATGGGTAGAGGAGCGTTCTGTAAGCCTGTGAAGGTGAATCGAGAGGTTTGCTGGAGGTATCAGAAGTGCGAATGCTGACATAAGTAACGATAATGTGGGTGAAAAACCCACACGCCGAAAGTCTAAGGTTTCCTGCGCAACGTTAATCG 1333

NZ_CP007555.1:337406-341752(+) GATGGGTAGAGGAGCGTTCTGTAAGCCTGTGAAGGTGAATCGAGAGGTTTGCTGGAGGTATCAGAAGTGCGAATGCTGACATAAGTAACGATAATGTGGGTGAAAAACCCACACGCCGAAAGTCTAAGGTTTCCTGCGCAACGTTAATCG 1333

NZ_JASNNV010000010.1:39284-43631(-) GATGGGTAGAGGAGCGTTCTGTAAGCCTGTGAAGGTGAATCGAGAGGTTTGCTGGAGGTATCAGAAGTGCGAATGCTGACATAAGTAACGATAATGTGGGTGAAAAACCCACACGCCGAAAGTCTAAGGTTTCCTGCGCAACGTTAATCG 1333

NZ_JAOXFC010000012.1:14231-18577(+) GATGGGTAGAGGAGCGTTCTGTAAGCCTGTGAAGGTGAATCGAGAGGTTTGCTGGAGGTATCAGAAGTGCGAATGCTGACATAAGTAACGATAATGTGGGTGAAAAACCCACACGCCGAAAGTCTAAGGTTTCCTGCGCAACGTTAATCG 1333

NZ_CP103430.1:167658-171997(+) GATGGGTAGAGGAGCGTTCTGTAAGCCTGTGAAGGTGAATCGAGAGGTTTGCTGGAGGTATCAGAAGTGCGAATGCTGACATAAGTAACGATAATGTGGGTGAAAAACCCACACGCCGAAAGTCTAAGGTTTCCTGCGCAACGTTAATCG 1333

NZ_PDLP01000011.1:14441-18787(+) GATGGGTAGAGGAGCGTTCTGTAAGCCTGTGAAGGTGAATCGAGAGGTTTGCTGGAGGTATCAGAAGTGCGAATGCTGACATAAGTAACGATAATGTGGGTGAAAAACCCACACGCCGAAAGTCTAAGGTTTCCTGCGCAACGTTAATCG 1333

NZ_NOLM01000022.1:91220-95566(-) GATGGGTAGAGGAGCGTTCTGTAAGCCTGTGAAGGTGAATCGAGAGGTTTGCTGGAGGTATCAGAAGTGCGAATGCTGACATAAGTAACGATAATGTGGGTGAAAAACCCACACGCCGAAAGTCTAAGGTTTCCTGCGCAACGTTAATCG 1333

JBJCIS010000056.1:0-2780(-) GATGGGTAGAGGAGCGTTCTGTAAGCCTGTGAAGGTGAATCGAGAGGTTTGCTGGAGGTATCAGAAGTGCGAATGCTGACATAAGTAACGATAATGTGGGTGAAAAACCCACACGCCGAAAGTCTAAGGTTTCCTGCGCAACGTTAATCG 1333

NZ_CDBG01000001.1:179744-184090(+) GATGGGTAGAGGAGCGTTCTGTAAGCCTGTGAAGGTGAATCGAGAGGTTTGCTGGAGGTATCAGAAGTGCGAATGCTGACATAAGTAACGATAATGTGGGTGAAAAACCCACACGCCGAAAGTCTAAGGTTTCCTGCGCAACGTTAATCG 1333

NZ_CP103434.1:167622-171961(+) GATGGGTAGAGGAGCGTTCTGTAAGCCTGTGAAGGTGAATCGAGAGGTTTGCTGGAGGTATCAGAAGTGCGAATGCTGACATAAGTAACGATAATGTGGGTGAAAAACCCACACGCCGAAAGTCTAAGGTTTCCTGCGCAACGTTAATCG 1333

CP014354.1:76692-80603(-) GATGGGTAGAGGAGCGTTCTGTAAGCCTGTGAAGGTGAATCGAGAGGTTTGCTGGAGGTATCAGAAGTGCGAATGCTGACATAAGTAACGATAATGTGGGTGAAAAACCCACACGCCGAAAGTCTAAGGTTTCCTGCGCAACGTTAATCG 864

NOLR01000002.1:131833-136207(+) GATGGGTAGAGGAGCGTTCTGTAAGCCTGTGAAGGTGAATCGAGAGGTTTGCTGGAGGTATCAGAAGTGCGAATGCTGACATAAGTAACGATAATGTGGGTGAAAAACCCACACGCCAAAAGTCTAAGGTTTCCTGCGCAACGTTAATCG 1343

PPFR01000008.1:34360-38734(-) GATGGGTAGAGGAGCGTTCTGTAAGCCTGTGAAGGTGAATCGAGAGGTTTGCTGGAGGTATCAGAAGTGCGAATGCTGACATAAGTAACGATAATGTGGGTGAAAAACCCACACGCCGAAAGTCTAAGGTTTCCTGCGCAACGTTAATCG 1343

PPFQ01000003.1:131083-135464(+) GATGGGTAGAGGAGCGTTCTGTAAGCCTGTGAAGGTGAATCGAGAGGTTTGCTGGAGGTATCAGAAGTGCGAATGCTGACATAAGTAACGATAATGTGGGTGAAAAACCCACACGCCAAAAGTCTAAGGTTTCCTGCGCAACGTTAATCG 1343

NZ_AKYP01000181.1:1647-6020(-) GATGGGTAGAGGAGCGTTCTGTAAGCCTGTGAAGGTGAATCGAGAGGTTTGCTGGAGGTATCAGAAGTGCGAATGCTGACATAAGTAACGATAATGTGGGTGAA-AACCCACACGCCGAAAGTCTAAGGTTTCCTGCGCAACGTTAATCG 1342

NOVI01000003.1:105955-110328(+) GATGGGTAGAGGAGCGTTCTGTAAGCCTGTGAAGGTGAATCGAGAGGTTTGCTGGAGGTATCAGAAGTGCGAATGCTGACATAAGTAACGATAATGTGGGTGAAAAACCCACACGCCGAAAGTCTAAGGTTTCCTGCGCAACGTTAATCG 1343

CP018150.1:339294-343661(+) GATGGGTAGAGGAGCGTTCTGTAAGCCTGTGAAGGTGAATCGAGAGGTTTGCTGGAGGTATCAGAAGTGCGAATGCTGACATAAGTAACGATAATGTGGGTGAAAAACCCACACGCCGAAAGTCTAAGGTTTCCTGCGCAACGTTAATCG 1343

Consensus GCGCAGGGTGAGTCGGCCCCTAAGGCGAGGCAGAAATGCGTAGTCGATGGGAAACGGGTTAATATTCCCGTACTTTATAATACTGCGATGGGAGGACGGAGAAGGCTAGGTCAGCCACCCGATGGTTGTGGTGGTTTAAGTGTGTAGGAA 1493

JAKFBC010000002.1:34496-38842(-) (45) ACGCAGGGTGAGTCGGCCCCTAAGGCGAGGCAGAAATGCGTAGTCGATGGGAAACGGGTTAATATTCCCGTACTTTATAATACTGCGATGGGAGGACGGAGAAGGCTAGGTCAGCCACCCGATGGTTGTGGTGGTTTAAGTGTGTAGGAA 1483

NZ_AP019757.1:1739264-1743610(-) (31) GCGCAGGGTGAGTCGGCCCCTAAGGCGAGGCAGAAATGCGTAGTCGATGGGAAACGGGTTAATATTCCCGTACTTTATAATACTGCGATGGGAGGACGGAGAAGGCTAGGTCAGCCACCCGATGGTTGTGGTGGTTTAAGTGTGTAGGAA 1483

JBKOHN010000001.1:81530-85875(+) (25) GCGCAGGGTGAGTCGGCCCCTAAGGCGAGGCAGAAATGCGTAGTCGATGGGAAACGGGTTAATATTCCCGTACTTTATAATACTGCGATGGGAGGACGGAGAAGGCTAGGTCAGCCACCCGATGGTTGTGGTGGTTTAAGTGTGTAGGAA 1483

NC_011528.1:339307-343646(+) (14) GCGCAGGGTGAGTCGGCCCCTAAGGCGAGGCAGAAATGCGTAGTCGATGGGAAACGGGTTAATATTCCCGTACTTTACAATACTGCGATGGGAGGACGGAGAAGGCTAGGTCAGCCACCCGATGGTTGTGGTGGTTTAAGTGTGTAGGAA 1483

CP018005.1:167566-171940(+) (14) ACGCAGGGTGAGTCGGCCCCTAAGGCGAGGCAGAAATGCGTAGTCGATGGGAAACGGGTTAATATTCCCGTACTTTATAATACTGCGATGGGAGGACGGAGAAGGCTAGGTCAGCCACCCGATGGTTGTGGTGGTTTAAGTGTGTAGGAA 1493

NZ_CCAM010000005.1:71418-75764(+) (13) GCGCAGGGTGAGTCGGCCCCTAAGGCGAGGCAGAAATGCGTAGTCGATGGGAAACGGGTTAATATTCCCGTACTTTATAATACTGCGATGGGAGGACGGAGAAGGCTAGGTCAGCCACCCGATGGTTGTGGTGGTTTAAGTGTGTAGGAA 1483

NZ_CCXO01000001.1:317226-321572(+) (11) GCGCAGGGTGAGTCGGCCCCTAAGGCGAGGCAGAAATGCGTAGTCGATGGGAAACGGGTTAATATTCCCGTACTTTACAATACTGCGATGGGAGGACGGAGAAGGCTAGGTCAGCCACCCGATGGTTGTGGTGGTTTAAGTGTGTAGGAA 1483

NC_011527.1:1751498-1755844(-) (8) GCGCAGGGTGAGTCGGCCCCTAAGGCGAGGCAGAAATGCGTAGTCGATGGGAAACGGGTTAATATTCCCGTACTTTACAATACTGCGATGGGAGGACGGAGAAGGCTAGGTCAGCCACCCGATGGTTGTGGTGGTTTAAGTGTGTAGGAA 1483

NZ_CP032542.1:88036-92382(-) (8) GCGCAGGGTGAGTCGGCCCCTAAGGCGAGGCAGAAATGCGTAGTCGATGGGAAACGGGTTAATATTCCCGTACTTTATAATACTGCGATGGGAGGACGGAGAAGGCTAGGTCAGCCACCCGATGGTTGTGGTGGTTTAAGTGTGTAGGAA 1483

CP014551.1:161236-165610(+) (8) GCGCAGGGTGAGTCGGCCCCTAAGGCGAGGCAGAAATGCGTAGTCGATGGGAAACGGGTTAATATTCCCGTACTTTATAATACTGCGATGGGAGGACGGAGAAGGCTAGGTCAGCCACCCGATGGTTGTGGTGGTTTAAGTGTGTAGGAA 1493

CP013667.1:88011-92385(-) (8) GCGCAGGGTGAGTCGGCCCCTAAGGCGAGGCAGAAATGCGTAGTCGATGGGAAACGGGTTAATATTCCCGTACTTTATAATACTGCGATGGGAGGACGGAGAAGGCTAGGTCAGCCACCCGATGGTTGTGGTGGTTTAAGTGTGTAGGAA 1493

CP107247.1:164350-168689(+) (7) GCGCAGGGTGAGTCGGCCCCTAAGGCGAGGCAGAAATGCGTAGTCGATGGGAAACGGGTTAATATTCCCGTACTTTACAATACTGCGATGGGAGGACGGAGAAGGCTAGGTCAGCCACCCGATGGTTGTGGTGGTTTAAGTGTGTAGGAA 1483

NZ_CP103435.1:167581-171920(+) (6) ACGCAGGGTGAGTCGGCCCCTAAGGCGAGGCAGAAATGCGTAGTCGATGGGAAACGGGTTAATATTCCCGTACTTTATAATACTGCGATGGGAGGACGGAGAAGGCTAGGTCAGCCACCCGATGGTTGTGGTGGTTTAAGTGTGTAGGAA 1483

CP014565.1:1711747-1716121(-) (3) GCGCAGGGTGAGTCGGCCCCTAAGGCGAGGCAGAAATGCGTAGTCGATGGGAAACGGGTTAATATTCCCGTACTTTACAATACTGCGATGGGAGGACGGAGAAGGCTAGGTCAGCCACCCGATGGTTGTGGTGGTTTAAGTGTGTAGGAA 1493

AAYJ01000007.1:0-1404(+) (3) ------------------------------------------------------------------------------------------------------------------------------------------------------

NZ_JAOXDR010000002.1:16672-21025(-) (3) GCGCAGGGTGAGTCGGCCCCTAAGGCGAGGCAGAAATGCGTAGTCGATGGGAAACGGGTTAATATTCCCGTACTTTATAATACTGCGATGGGAGGACGGAGAAGGCTAGGTCAGCCACCCGATGGTTGTGGTGGTTTAAGTGTGTAGGAA 1483

NZ_CP103432.1:167699-172038(+) (3) GCGCAGGGTGAGTCGGCCCCTAAGGCGAGGCAGAAATGCGTAGTCGATGGGAAACGGGTTAATATTCCCGTACTTTATAATACTGCGATGGGAGGACGGAGAAGGCTAGGTCAGCCACCCGATGGTTGTGGTGGTTTAAGTGTGTAGGAA 1483

NZ_CP103431.1:167697-172036(+) (3) GCGCAGGGTGAGTCGGCCCCTAAGGCGAGGCAGAAATGCGTAGTCGATGGGAAACGGGTTAATATTCCCGTACTTTACAATACTGCGATGGGAGGACGGAGAAGGCTAGGTCAGCCACCCGATGGTTGTGGTGGTTTAAGTGTGTAGGAA 1483

NZ_CP103428.1:167591-171929(+) (2) GCGCAGGGTGAGTCGGCCCCTAAGGCGAGGCAGAAATGCGTAGTCGATGGGAAACGGGTTAATATTCCCGTACTTTATAATACTGCGATGGGAGGACGGAGAAGGCTAGGTCAGCCACCCGATGGTTGTGGTGGTTTAAGTGTGTAGGAA 1483

NZ_JANTNR010000002.1:115350-119696(+) (2) ACGCAGGGTGAGTCGGCCCCTAAGGCGAGGCAGAAATGCGTAGTCGATGGGAAACGGGTTAATATTCCCGTACTTTATAATACTGCGATGGGAGGACGGAGAAGGCTAGGTCAGCCACCCGATGGTTGTGGTGGTTTAAGTGTGTAGGAA 1483

JARBIR010004564.1:3782-8128(-) (2) GCGCAGGGTGAGTCGGCCCCTAAGGCGAGGCAGAAATGCGTAGTCGATGGGAAACGGGTTAATATTCCCGTACTTTATAATACTGCGATGGGAGGACGGAGAAGGCTAGGTCAGCCACCCGATGGTTGTGGTGGTTTAAGTGTGTAGGAA 1483

NC_009727.1:1888349-1892695(-) (2) GCGCAGGGTGAGTCGGCCCCTAAGGCGAGGCAGAAATGCGTAGTCGATGGGAAACGGGTTAATATTCCCGTACTTTACAATACTGCGATGGGAGGACGGAGAAGGCTAGGTCAGCCACCCGATGGTTGTGGTGGTTTAAGTGTGTAGGAA 1483

AAYJ01000139.1:0-660(+) (2) GCGCAGGGTGAGTCGGCCCCTAAGGCGAGGCAGAAATGCGTAGTCGATGGGAAACGGGTTAATATTCCCGTACTTTACAATACTGCGATGGGAGGACGGAGAAGGCTAGGTCAGCCACCCGATGGTTGTGGTGGTTTAAGTGTGTAGGAA 309

CP032542.1:88018-92392(-) (2) GCGCAGGGTGAGTCGGCCCCTAAGGCGAGGCAGAAATGCGTAGTCGATGGGAAACGGGTTAATATTCCCGTACTTTATAATACTGCGATGGGAGGACGGAGAAGGCTAGGTCAGCCACCCGATGGTTGTGGTGGTTTAAGTGTGTAGGAA 1493

CP014559.1:232744-237117(+) (2) GCGCAGGGTGAGTCGGCCCCTAAGGCGAGGCAGAAATGCGTAGTCGATGGGAAACGGGTTAATATTCCCGTACTTTATAATACTGCGATGGGAGGACGGAGAAGGCTAGGTCAGCCACCCGATGGTTGTGGTGGTTTAAGTGTGTAGGAA 1493

CP007555.1:337408-341752(+) GCGCAGGGTGAGTCGGCCCCTAAGGCGAGGCAGAAATGCGTAGTCGATGGGAAACGGGTTAATATTCCCGTACTTTACAATACTGCGATGGGAGGACGGAGAAGGCTAGGTCAGCCACCCGATGGTTGTGGTGGTTTAAGTGTGTAGGAA 1481

NZ_CP014354.1:76710-81178(-) GCGCAGGGTGAGTCGGCCCCTAAGGCGAGGCAGAAATGCGTAGTCGATGGGAAACGGGTTAATATTCCCGTACTTTATAATACTGCGATGGGAGGACGGAGAAGGCTAGGTCAGCCACCCGATGGTTGTGGTGGTTTAAGTGTGTAGGAA 1489

CP000890.1:243129-244433(+) GCGCAGGGTGAGTCGGCCCCTAAGGCGAGGCAGAAATGCGTAGTCGATGGGAAACGGGTTAATATTCCCGTACTTTATAATACTGCGATGGGAGGACGGAGAAGGCTAGGTCAGCCACCCGATGGTTGTGGTGGTTTAAGTGTGTAGGAA 309

NZ_CP115461.1:167576-171922(+) ACGCAGGGTGAGTCGGCCCCTAAGGCGAGGCAGAAATGCGTAGTCGATGGGAAACGGGTTAATATTCCCGTACTTTATAATACTGCGATGGGAGGACGGAGAAGGCTAGGTCAGCCACCCGATGGTTGTGGTGGTTTAAGTGTGTAGGAA 1483

AP019759.1:167578-171919(+) ACGCAGGGTGAGTCGGCCCCTAAGGCGAGGCAGAAATGCGTAGTCGATGGGAAACGGGTTAATATTCCCGTACTTTATAATACTGCGATGGGAGGACGGAGAAGGCTAGGTCAGCCACCCGATGGTTGTGGTGGTTTAAGTGTGTAGGAA 1481

AP019757.1:1739267-1743608(-) GCGCAGGGTGAGTCGGCCCCTAAGGCGAGGCAGAAATGCGTAGTCGATGGGAAACGGGTTAATATTCCCGTACTTTATAATACTGCGATGGGAGGACGGAGAAGGCTAGGTCAGCCACCCGATGGTTGTGGTGGTTTAAGTGTGTAGGAA 1481

NOLN01000021.1:90861-95235(-) GCGCAGGGTGAGTCGGCCCCTAAGGCGAGGCAGAAATGCGTAGTCGATGGGAAACGGGTTAATATTCCCGTACTTTACAATACTGCGATGGGAGGACGGAGAAGGCTAGGTCAGCCACCCGATGGTTGTGGTGGTTTAAGTGTGTAGGAA 1493

NOLM01000022.1:91202-95576(-) GCGCAGGGTGAGTCGGCCCCTAAGGCGAGGCAGAAATGCGTAGTCGATGGGAAACGGGTTAATATTCCCGTACTTTACAATACTGCGATGGGAGGACGGAGAAGGCTAGGTCAGCCACCCGATGGTTGTGGTGGTTTAAGTGTGTAGGAA 1493

PDLP01000011.1:14431-18805(+) GCGCAGGGTGAGTCGGCCCCTAAGGCGAGGCAGAAATGCGTAGTCGATGGGAAACGGGTTAATATTCCCGTACTTTACAATACTGCGATGGGAGGACGGAGAAGGCTAGGTCAGCCACCCGATGGTTGTGGTGGTTTAAGTGTGTAGGAA 1493

CP014563.1:522940-527314(+) GCGCAGGGTGAGTCGGCCCCTAAGGCGAGGCAGAAATGCGTAGTCGATGGGAAACGGGTTAATATTCCCGTACTTTACAATACTGCGATGGGAGGACGGAGAAGGCTAGGTCAGCCACCCGATGGTTGTGGTGGTTTAAGTGTGTAGGAA 1493

CP000890.1:244922-245905(+) ------------------------------------------------------------------------------------------------------------------------------------------------------

NZ_NOLR01000002.1:131843-136189(+) GCGCAGGGTGAGTCGGCCCCTAAGGCGAGGCAGAAATGCGTAGTCGATGGGAAACGGGTTAATATTCCCGTACTTTATAATACTGCGATGGGAGGACGGAGAAGGCTAGGTCAGCCACCCGATGGTTGTGGTGGTTTAAGTGTGTAGGAA 1483

NZ_JAOXDP010000002.1:15794-20139(-) GCGCAGGGTGAGTCGGCCCCTAAGGCGAGGCAGAAATGCGTAGTCGATGGGAAACGGGTTAATATTCCCGTACTTTATAATACTGCGATGGGAGGACGGAGAAGGCTAGGTCAGCCACCCGATGGTTGTGGTGGTTTAAGTGTGTAGGAA 1483

NZ_PPFR01000008.1:34378-38724(-) GCGCAGGGTGAGTCGGCCCCTAAGGCGAGGCAGAAATGCGTAGTCGATGGGAAACGGGTTAATATTCCCGTACTTTATAATACTGCGATGGGAGGACGGAGAAGGCTAGGTCAGCCACCCGATGGTTGTGGTGGTTTAAGTGTGTAGGAA 1483

NZ_PPFQ01000003.1:131093-135446(+) GCGCAGGGTGAGTCGGCCCCTAAGGCGAGGCAGAAATGCGTAGTCGATGGGAAACGGGTTAATATTCCCGTACTTTATAATACTGCGATGGGAGGACGGAGAAGGCTAGGTCAGCCACCCGATGGTTGTGGTGGTTTAAGTGTGTAGGAA 1483

NZ_LK937696.1:167613-171953(+) GCGCAGGGTGAGTCGGCCCCTAAGGCGAGGCAGAAATGCGTAGTCGATGGGAAACGGGTTAATATTCCCGTACTTTATAATACTGCGATGGGAGGACGGAGAAGGCTAGGTCAGCCACCCGATGGTTGTGGTGGTTTAAGTGTGTAGGAA 1483

NZ_NOVI01000003.1:105965-110310(+) GCGCAGGGTGAGTCGGCCCCTAAGGCGAGGCAGAAATGCGTAGTCGATGGGAAACGGGTTAATATTCCCGTACTTTATAATACTGCGATGGGAGGACGGAGAAGGCTAGGTCAGCCACCCGATGGTTGTGGTGGTTTAAGTGTGTAGGAA 1483

NZ_CP103426.1:167642-171981(+) GCGCAGGGTGAGTCGGCCCCTAAGGCGAGGCAGAAATGCGTAGTCGATGGGAAACGGGTTAATATTCCCGTACTTTATAATACTGCGATGGGAGGACGGAGAAGGCTAGGTCAGCCACCCGATGGTTGTGGTGGTTTAAGTGTGTAGGAA 1483

NZ_JAOXDN010000007.1:36985-41324(-) ACGCAGGGTGAGTCGGCCCCTAAGGCGAGGCAGAAATGCGTAGTCGATGGGAAACGGGTTAATATTCCCGTACTTTATAATACTGCGATGGGAGGACGGAGAAGGCTAGGTCAGCCACCCGATGGTTGTGGTGGTTTAAGTGTGTAGGAA 1483

NZ_JPVV01000013.1:13888-18051(+) GCGCAGGGTGAGTCGGCCCCTAAGGCGAGGCAGAAATGCGTAGTCGATGGGAAACGGGTTAATATTCCCGTACTTTACAATACTGCGATGGGAGGACGGAGAAGGCTAGGTCAGCCACCCGATGGTTGTGGTGGTTTAAGTGTGTAGGAA 1483

NZ_CP007555.1:337406-341752(+) GCGCAGGGTGAGTCGGCCCCTAAGGCGAGGCAGAAATGCGTAGTCGATGGGAAACGGGTTAATATTCCCGTACTTTACAATACTGCGATGGGAGGACGGAGAAGGCTAGGTCAGCCACCCGATGGTTGTGGTGGTTTAAGTGTGTAGGAA 1483

NZ_JASNNV010000010.1:39284-43631(-) GCGCAGGGTGAGTCGGCCCCTAAGGCGAGGCAGAAATGCGTAGTCGATGGGAAACGGGTTAATATTCCCGTACTTTACAATACTGCGATGGGAGGACGGAGAAGGCTAGGTCAGCCACCCGATGGTTGTGGTGGTTTAAGTGTGTAGGAA 1483

NZ_JAOXFC010000012.1:14231-18577(+) GCGCAGGGTGAGTCGGCCCCTAAGGCGAGGCAGAAATGCGTAGTCGATGGGAAACGGGTTAATATTCCCGTACTTTACAATACTGCGATGGGAGGACGGAGAAGGCTAGGTCAGCCACCCGATGGTTGTGGTGGTTTAAGTGTGTAGGAA 1483

NZ_CP103430.1:167658-171997(+) GCGCAGGGTGAGTCGGCCCCTAAGGCGAGGCAGAAATGCGTAGTCGATGGGAAACGGGTTAATATTCCCGTACTTTACAATACTGCGATGGGAGGACGGAGAAGGCTAGGTCAGCCACCCGATGGTTGTGGTGGTTTAAGTGTGTAGGAA 1483

NZ_PDLP01000011.1:14441-18787(+) GCGCAGGGTGAGTCGGCCCCTAAGGCGAGGCAGAAATGCGTAGTCGATGGGAAACGGGTTAATATTCCCGTACTTTACAATACTGCGATGGGAGGACGGAGAAGGCTAGGTCAGCCACCCGATGGTTGTGGTGGTTTAAGTGTGTAGGAA 1483

NZ_NOLM01000022.1:91220-95566(-) GCGCAGGGTGAGTCGGCCCCTAAGGCGAGGCAGAAATGCGTAGTCGATGGGAAACGGGTTAATATTCCCGTACTTTACAATACTGCGATGGGAGGACGGAGAAGGCTAGGTCAGCCACCCGATGGTTGTGGTGGTTTAAGTGTGTAGGAA 1483

JBJCIS010000056.1:0-2780(-) GCGCAGGGTGAGTCGGCCCCTAAGGCGAGGCAGAAATGCGTAGTCGATGGGAAACGGGTTAATATTCCCGTACTTTACAATACTGCGATGGGAGGACGGAGAAGGCTAGGTCAGCCACCCGATGGTTGTGGTGGTTTAAGTGTGTAGGAA 1483

NZ_CDBG01000001.1:179744-184090(+) GCGCAGGGTGAGTCGGCCCCTAAGGCGAGGCAGAAATGCGTAGTCGATGGGAAACGGGTTAATATTCCCGTACTTTAYAATACTGCGATGGGAGGACGGAGAAGGCTAGGTCAGCCACCCGATGGTTGTGGTGGTTTAAGTGTGTAGGAA 1483

NZ_CP103434.1:167622-171961(+) GCGCAGGGTGAGTCGGCCCCTAAGGCGAGGCAGAAATGCGTAGTCGATGGGAAACGGGTTAATATTCCCGTACTTTATAATACTGCGATGGGAGGACGGAGAAGGCTAGGTCAGCCACCCGATGGTTGTGGTGGTTTAAGTGTGTAGGAA 1483

CP014354.1:76692-80603(-) GCGCAGGGTGAGTCGGCCCCTAAGGCGAGGCAGAAATGCGTAGTCGATGGGAAACGGGTTAATATTCCCGTACTTTATAATACTGCGATGGGAGGACGGAGAAGGCTAGGTCAGCCACCCGATGGTTGTGGTGGTTTAAGTGTGTAGGAA 1014

NOLR01000002.1:131833-136207(+) GCGCAGGGTGAGTCGGCCCCTAAGGCGAGGCAGAAATGCGTAGTCGATGGGAAACGGGTTAATATTCCCGTACTTTATAATACTGCGATGGGAGGACGGAGAAGGCTAGGTCAGCCACCCGATGGTTGTGGTGGTTTAAGTGTGTAGGAA 1493

PPFR01000008.1:34360-38734(-) GCGCAGGGTGAGTCGGCCCCTAAGGCGAGGCAGAAATGCGTAGTCGATGGGAAACGGGTTAATATTCCCGTACTTTATAATACTGCGATGGGAGGACGGAGAAGGCTAGGTCAGCCACCCGATGGTTGTGGTGGTTTAAGTGTGTAGGAA 1493

PPFQ01000003.1:131083-135464(+) GCGCAGGGTGAGTCGGCCCCTAAGGCGAGGCAGAAATGCGTAGTCGATGGGAAACGGGTTAATATTCCCGTACTTTATAATACTGCGATGGGAGGACGGAGAAGGCTAGGTCAGCCACCCGATGGTTGTGGTGGTTTAAGTGTGTAGGAA 1493

NZ_AKYP01000181.1:1647-6020(-) GCGCAGGGTGAGTCGGCCCCTAAGGCGAGGCAGAAATGCGTAGTCGATGGGAAACGGGTTAATATTCCCGTACTTTATAATACTGCGATGGGAGGACGGAGAAGGCTAGGTCAGCCACCCGATGGTTGTGGTGGTTTAAGTGTGTAGGAA 1492

NOVI01000003.1:105955-110328(+) GCGCAGGGTGAGTCGGCCCCTAAGGCGAGGCAGAAATGCGTAGTCGATGGGAAACGGGTTAATATTCCCGTACTTTATAATACTGCGATGGGAGGACGGAGAAGGCTAGGTCAGCCACCCGATGGTTGTGGTGGTTTAAGTGTGTAGGAA 1493

CP018150.1:339294-343661(+) GCGCAGGGTGAGTCGGCCCCTAAGGCGAGGCAGAAATGCGTAGTCGATGGGAAACGGGTTAATATTCCCGTACTTTACAATACTGCGATGGGAGGACGGAGAAGGCTAGGTCAGCCACCCGATGGTTGTGGTGGTTTAAGTGTGTAGGAA 1493

Consensus GGGTTCTTTGGCAAATCCGGGAACTCAATTCCGAGACATGATGACGAAGTACGAACTTGTTCGTGCAAAGTGATTGATGCCACGCTTCCAGGAAAAGTCCCTAAGCTTCAGGTATTGTAAAACCGTACTATAAACCGACACAGGTGGACA 1643

JAKFBC010000002.1:34496-38842(-) (45) GGGTTCTTTGGCAAATCCGGGAACTCAATTCCGAGACATGATGACGAAGTACGAACTTGTTCGTGCAAAGTGATTGATGCCACGCTTCCAGGAAAAGTCCCTAAGCTTCAGGTATTGTAAAACCGTACTATAAACCGACACAGGTGGACA 1633

NZ_AP019757.1:1739264-1743610(-) (31) GGGTTCTTTGGCAAATCCGGGAACTCAATTCCGAGACATGATGACGAAGTACGAACTTGTTCGTGCAAAGTGATTGATGCCACGCTTCCAGGAAAAGTCCCTAAGCTTCAGGTATTGTAAAACCGTACTATAAACCGACACAGGTGGACA 1633

JBKOHN010000001.1:81530-85875(+) (25) GGGTTCTTTGGCAAATCCGGGAACTCAATTCCGAGACATGATGACGAAGTACGAACTTGTTCGTGCAAAGTGATTGATGCCACGCTTCCAGGAAAAGTCCCTAAGCTTCAGGTATTGTAAAACCGTACTATAAACCGACACAGGTGGACA 1633

NC_011528.1:339307-343646(+) (14) GGGTTCTTTGGCAAATCCGGGAACTCAATTCCGAGACATGATGACGAAGTACGAACTTGTTCGTGCAAAGTGATTGATGCCACGCTTCCAGGAAAAGTCCCTAAGCTTCAGGTATTGTAAAACTGTACTATAAACCGACACAGGTGGACA 1633

CP018005.1:167566-171940(+) (14) GGGTTCTTTGGCAAATCCGGGAACTCAATTCCGAGACATGATGACGAAGTACGAACTTGTTCGTGCAAAGTGATTGATGCCACGCTTCCAGGAAAAGTCCCTAAGCTTCAGGTATTGTAAAACCGTACTATAAACCGACACAGGTGGACA 1643

NZ_CCAM010000005.1:71418-75764(+) (13) GGGTTCTTTGGCAAATCCGGGAACTCAATTCCGAGACATGATGACGAAGTACGAACTTGTTCGTGCAAAGTGATTGATGCCACGCTTCCAGGAAAAGTCCCTAAGCTTCAGGTATTGTAAAACCGTACTATAAACCGACACAGGTGGACA 1633

NZ_CCXO01000001.1:317226-321572(+) (11) GGGTTCTTTGGCAAATCCGGGAACTCAATTCCGAGACATGATGACGAAGTACGAACTTGTTCGTGCAAAGTGATTGATGCCACGCTTCCAGGAAAAGTCCCTAAGCTTCAGGTATTGTAAAACTGTACTATAAACCGACACAGGTGGACA 1633

NC_011527.1:1751498-1755844(-) (8) GGGTTCTTTGGCAAATCCGGGAACTCAATTCCGAGACATGATGACGAAGTACGAACTTGTTCGTGCAAAGTGATTGATGCCACGCTTCCAGGAAAAGTCCCTAAGCTTCAGGTATTGTAAAACCGTACTATAAACCGACACAGGTGGACA 1633

NZ_CP032542.1:88036-92382(-) (8) GGGTTCTTTGGCAAATCCGGGAACTCAATTCCGAGACATGATGACGAAGTACGAACTTGTTCGTGCAAAGTGATTGATGCCACGCTTCCAGGAAAAGTCCCTAAGCTTCAGGTATTGTAAAACCGTACTATAAACCGACACAGGTGGACA 1633

CP014551.1:161236-165610(+) (8) GGGTTCTTTGGCAAATCCGGGAACTCAATTCCGAGACATGATGACGAAGTACGAACTTGTTCGTGCAAAGTGATTGATGCCACGCTTCCAGGAAAAGTCCCTAAGCTTCAGGTATTGTAAAACCGTACTATAAACCGACACAGGTGGACA 1643

CP013667.1:88011-92385(-) (8) GGGTTCTTTGGCAAATCCGGGAACTCAATTCCGAGACATGATGACGAAGTACGAACTTGTTCGTGCAAAGTGATTGATGCCACGCTTCCAGGAAAAGTCCCTAAGCTTCAGGTATTGTAAAACCGTACTATAAACCGACACAGGTGGACA 1643

CP107247.1:164350-168689(+) (7) GGGTTCTTTGGCAAATCCGGGAACTCAATTCCGAGACATGATGACGAAGTACGAACTTGTTCGTGCAAAGTGATTGATGCCACGCTTCCAGGAAAAGTCCCTAAGCTTCAGGTATTGTAAAACTGTACTATAAACCGACACAGGTGGACA 1633

NZ_CP103435.1:167581-171920(+) (6) GGGTTCTTTGGCAAATCCGGGAACTCAATTCCGAGACATGATGACGAAGTACGAACTTGTTCGTGCAAAGTGATTGATGCCACGCTTCCAGGAAAAGTCCCTAAGCTTCAGGTATTGTAAAACCGTACTATAAACCGACACAGGTGGACA 1633

CP014565.1:1711747-1716121(-) (3) GGGTTCTTTGGCAAATCCGGGAACTCAATTCCGAGACATGATGACGAAGTACGAACTTGTTCGTGCAAAGTGATTGATGCCACGCTTCCAGGAAAAGTCCCTAAGCTTCAGGTATTGTAAAACCGTACTATAAACCGACACAGGTGGACA 1643

AAYJ01000007.1:0-1404(+) (3) ------------------------------------------------------------------------------------------------------------------------------------------------------

NZ_JAOXDR010000002.1:16672-21025(-) (3) GGGTTCTTTGGCAAATCCGGGAACTCAATTCCGAGACATGATGACGAAGTACGAACTTGTTCGTGCAAAGTGATTGATGCCACGCTTCCAGGAAAAGTCCCTAAGCTTCAGGTATTGTAAAACCGTACTATAAACCGACACAGGTGGACA 1633

NZ_CP103432.1:167699-172038(+) (3) GGGTTCTTTGGCAAATCCGGGAACTCAATTCCGAGACATGATGACGAAGTACGAACTTGTTCGTGCAAAGTGATTGATGCCACGCTTCCAGGAAAAGTCCCTAAGCTTCAGGTATTGTAAAACCGTACTATAAACCGACACAGGTGGACA 1633

NZ_CP103431.1:167697-172036(+) (3) GGGTTCTTTGGCAAATCCGGGAACTCAATTCCGAGACATGATGACGAAGTACGAACTTGTTCGTGCAAAGTGATTGATGCCACGCTTCCAGGAAAAGTCCCTAAGCTTCAGGTATTGTAAAACTGTACTATAAACCGACACAGGTGGACA 1633

NZ_CP103428.1:167591-171929(+) (2) GGGTTCTTTGGCAAATCCGGGAACTCAATTCCGAGACATGATGACGAAGTACGAACTTGTTCGTGCAAAGTGATTGATGCCACGCTTCCAGGAAAAGTCCCTAAGCTTCAGGTATTGTAAAACCGTACTATAAACCGACACAGGTGGACA 1633

NZ_JANTNR010000002.1:115350-119696(+) (2) GGATTCTTTGGCAAATCCGGGAACTCAATTCCGAGACATGATGACGAAGTACGAACTTGTTCGTGCAAAGTGATTGATGCCACGCTTCCAGGAAAAGTCCCTAAGCTTCAGGTATTGTAAAACCGTACTATAAACCGACACAGGTGGACA 1633

JARBIR010004564.1:3782-8128(-) (2) GGGTTCTTTGGCAAATCCGGGAACTCAATTCCGAGACATGATGACGAAGTACGAACTTGTTCGTGCAAAGTGATTGATGCCACGCTTCCAGGAAAAGTCCCTAAGCTTCAGGTATTGTAAAACCGTACTATAAACCGACACAGGTGGACA 1633

NC_009727.1:1888349-1892695(-) (2) GGGTTCTTTGGCAAATCCGGGAACTCAATTCCGAGACATGATGACGAAGTACGAACTTGTTCGTGCAAAGTGATTGATGCCACGCTTCCAGGAAAAGTCCCTAAGCTTCAGGTATTGTAAAACCGTACTATAAACCGACACAGGTGGACA 1633

AAYJ01000139.1:0-660(+) (2) GGGTTCTTTGGCAAATCCGGGAACTCAATTCCGAGACATGATGACGAAGTACGAACTTGTTCGTGCAAAGTGATTGATGCCACGCTTCCAGGAAAAGTCCCTAAGCTTCAGGTATTGTAAAACTGTACTATAAACCGACACAGGTGGACA 459

CP032542.1:88018-92392(-) (2) GGGTTCTTTGGCAAATCCGGGAACTCAATTCCGAGACATGATGACGAAGTACGAACTTGTTCGTGCAAAGTGATTGATGCCACGCTTCCAGGAAAAGTCCCTAAGCTTCAGGTATTGTAAAACCGTACTATAAACCGACACAGGTGGACA 1643

CP014559.1:232744-237117(+) (2) GGGTTCTTTGGCAAATCCGGGAACTCAATTCCGAGACATGATGACGAAGTACGAACTTGTTCGTGCAAAGTGATTGATGCCACGCTTCCAGGAAAAGTCCCTAAGCTTCAGGTATTGTAAAACCGTACTATAAACCGACACAGGTGGACA 1643

CP007555.1:337408-341752(+) GGGTTCTTTGGCAAATCCGGGAACTCAATTCCGAGACATGATGACGAAGTACGAACTTGTTCGTGCAAAGTGATTGATGCCACGCTTCCAGGAAAAGTCCCTAAGCTTCAGGTATTGTAAAACTGTACTATAAACCGACACAGGTGGACA 1631

NZ_CP014354.1:76710-81178(-) GGGTTCTTTGGCAAATCCGGGAACTCAATTCCGAGACATGATGACGAAGTACGAACTTGTTCGTGCAAAGTGATTGATGCCACGCTTCCAGGAAAAGTCCCTAAGCTTCAGGTATTGTAAAACCGTACTATAAACCGACACAGGTGGACA 1639

CP000890.1:243129-244433(+) GGGTTCTTTGGCAAATCCGGGAACTCAATTCCGAGACATGATGACGAAGTACGAACTTGTTCGTGCAAAGTGATTGATGCCACGCTTCCAGGAAAAGTCCCTAAGCTTCAGGTATTGTAAAACCGTACTATAAACCGACACAGGTGGACA 459

NZ_CP115461.1:167576-171922(+) GGGTTCTTTGGCAAATCCGGGAACTCAATTCCGAGACATGATGACGAAGTACGAACTTGTTCGTGCAAAGTGATTGATGCCACGCTTCCAGGAAAAGTCCCTAAGCTTCAGGTATTGTAAAACCGTACTATAAACCGACACAGGTGGACA 1633

AP019759.1:167578-171919(+) GGGTTCTTTGGCAAATCCGGGAACTCAATTCCGAGACATGATGACGAAGTACGAACTTGTTCGTGCAAAGTGATTGATGCCACGCTTCCAGGAAAAGTCCCTAAGCTTCAGGTATTGTAAAACCGTACTATAAACCGACACAGGTGGACA 1631

AP019757.1:1739267-1743608(-) GGGTTCTTTGGCAAATCCGGGAACTCAATTCCGAGACATGATGACGAAGTACGAACTTGTTCGTGCAAAGTGATTGATGCCACGCTTCCAGGAAAAGTCCCTAAGCTTCAGGTATTGTAAAACCGTACTATAAACCGACACAGGTGGACA 1631

NOLN01000021.1:90861-95235(-) GGGTTCTTTGGCAAATCCGGGAACTCAATTCCGAGACATGATGACGAAGTACGAACTTGTTCGTGCAAAGTGATTGATGCCACGCTTCCAGGAAAAGTCCCTAAGCTTCAGGTATTGTAAAACCGTACTATAAACCGACACAGGTGGACA 1643

NOLM01000022.1:91202-95576(-) GGGTTCTTTGGCAAATCCGGGAACTCAATTCCGAGACATGATGACGAAGTACGAACTTGTTCGTGCAAAGTGATTGATGCCACGCTTCCAGGAAAAGTCCCTAAGCTTCAGGTATTGTAAAACCGTACTATAAACCGACACAGGTGGACA 1643

PDLP01000011.1:14431-18805(+) GGGTTCTTTGGCAAATCCGGGAACTCAATTCCGAGACATGATGACGAAGTACGAACTTGTTCGTGCAAAGTGATTGATGCCACGCTTCCAGGAAAAGTCCCTAAGCTTCAGGTATTGTAAAACTGTACTATAAACCGACACAGGTGGACA 1643

CP014563.1:522940-527314(+) GGGTTCTTTGGCAAATCCGGGAACTCAATTCCGAGACATGATGACGAAGTACGAACTTGTTCGTGCAAAGTGATTGATGCCACGCTTCCAGGAAAAGTCCCTAAGCTTCAGGTATTGTAAAACTGTACTATAAACCGACACAGGTGGACA 1643

CP000890.1:244922-245905(+) ------------------------------------------------------------------------------------------------------------------------------------------------------

NZ_NOLR01000002.1:131843-136189(+) GGGTTCTTTGGCAAATCCGGGAACTCAATTCCGAGACATGATGACGAAGTACGAACTTGTTCGTGCAAAGTGATTGATGCCACGCTTCCAGGAAAAGTCCCTAAGCTTCAGGTATTGTAAAACCGTACTATAAACCGACACAGGTGGACA 1633

NZ_JAOXDP010000002.1:15794-20139(-) GGGTTCTTTGGCAAATCCGGGAACTCAATTCCGAGACATGATGACGAAGTACGAACTTGTTCGTGCAAAGTGATTGATGCCACGCTTCCAGGAAAAGTCCCTAAGCTTCAGGTATTGTAAAACCGTACTATAAACCGACACAGGTGGACA 1633

NZ_PPFR01000008.1:34378-38724(-) GGGTTCTTTGGCAAATCCGGGAACTCAATTCCGAGACATGATGACGAAGTACGAACTTGTTCGTGCAAAGTGATTGATGCCACGCTTCCAGGAAAAGTCCCTAAGCTTCAGGTATTGTAAAACCGTACTATAAACCGACACAGGTGGACA 1633

NZ_PPFQ01000003.1:131093-135446(+) GGGTTCTTTGGCAAATCCGGGAACTCAATTCCGAGACATGATGACGAAGTACGAACTTGTTCGTGCAAAGTGATTGATGCCACGCTTCCAGGAAAAGTCCCTAAGCTTCAGGTATTGTAAAACCGTACTATAAACCGACACAGGTGGACA 1633

NZ_LK937696.1:167613-171953(+) GGGTTCTTTGGCAAATCCGGGAACTCAATTCCGAGACATGATGACGAAGTACGAACTTGTTCGTGCAAAGTGATTGATGCCACGCTTCCAGGAAAAGTCCCTAAGCTTCAGGTATTGTAAAACCGTACTATAAACCGACACAGGTGGACA 1633

NZ_NOVI01000003.1:105965-110310(+) GGGTTCTTTGGCAAATCCGGGAACTCAATTCCGAGACATGATGACGAAGTACGAACTTGTTCGTGCAAAGTGATCGATGCCACGCTTCCAGGAAAAGTCCCTAAGCTTCAGGTATTGTAAAACCGTACTATAAACCGACACAGGTGGACA 1633

NZ_CP103426.1:167642-171981(+) GGGTTCTTTGGCAAATCCGGGAACTCAATTCCGAGACATGATGACGAAGTACGAACTTGTTCGTGCAAAGTGATTGATGCCACGCTTCCAGGAAAAGTCCCTAAGCTTCAGGTATTGTAAAACCGTACTATAAACCGACACAGGTGGACA 1633

NZ_JAOXDN010000007.1:36985-41324(-) GGGTTCTTTGGCAAATCCGGGAACTCAATTCCGAGACATGATGACGAAGTACGAACTTGTTCGTGCAAAGTGATTGATGCCACGCTTCCAGGAAAAGTCCCTAAGCTTCAGGTATTGTAAAACTGTACTATAAACCGACACAGGTGGACA 1633

NZ_JPVV01000013.1:13888-18051(+) GGGTTCTTTGGCAAATCCGGGAACTCAATTCCGAGACATGATGACGAAGTACGAACTTGTTCGTGCAAAGTGATTGATGCCACGCTTCCAGGAAAAGTCCCTAAGCTTCAGGTATTGTAAAACTGTACTATAAACCGACACAGGTGGACA 1633

NZ_CP007555.1:337406-341752(+) GGGTTCTTTGGCAAATCCGGGAACTCAATTCCGAGACATGATGACGAAGTACGAACTTGTTCGTGCAAAGTGATTGATGCCACGCTTCCAGGAAAAGTCCCTAAGCTTCAGGTATTGTAAAACTGTACTATAAACCGACACAGGTGGACA 1633

NZ_JASNNV010000010.1:39284-43631(-) GGGTTCTTTGGCAAATCCGGGAACTCAATTCCGAGACATGATGACGAAGTACGAACTTGTTCGTGCAAAGTGATTGATGCCACGCTTCCAGGAAAAGTCCCTAAGCTTCAGGTATTGTAAAACTGTACTATAAACCGACACAGGTGGACA 1633

NZ_JAOXFC010000012.1:14231-18577(+) GGGTTCTTTGGCAAATCCGGGAACTCAATTCCGAGACATGATGACGAAGTACGAACTTGTTCGTGCAAAGTGATTGATGCCACGCTTCCAGGAAAAGTCCCTAAGCTTCAGGTATTGTAAAACTGTACTATAAACCGACACAGGTGGACA 1633

NZ_CP103430.1:167658-171997(+) GGGTTCTTTGGCAAATCCGGGAACTCAATTCCGAGACATGATGACGAAGTACGAACTTGTTCGTGCAAAGTGATTGATGCCACGCTTCCAGGAAAAGTCCCTAAGCTTCAGGTATTGTAAAACTGTACTATAAACCGACACAGGTGGACA 1633

NZ_PDLP01000011.1:14441-18787(+) GGGTTCTTTGGCAAATCCGGGAACTCAATTCCGAGACATGATGACGAAGTACGAACTTGTTCGTGCAAAGTGATTGATGCCACGCTTCCAGGAAAAGTCCCTAAGCTTCAGGTATTGTAAAACTGTACTATAAACCGACACAGGTGGACA 1633

NZ_NOLM01000022.1:91220-95566(-) GGGTTCTTTGGCAAATCCGGGAACTCAATTCCGAGACATGATGACGAAGTACGAACTTGTTCGTGCAAAGTGATTGATGCCACGCTTCCAGGAAAAGTCCCTAAGCTTCAGGTATTGTAAAACCGTACTATAAACCGACACAGGTGGACA 1633

JBJCIS010000056.1:0-2780(-) GGGTTCTTTGGCAAATCCGGGAACTCAATTCCGAGACATGATGACGAAGTACGAACTTGTTCGTGCAAAGTGATTGATGCCACGCTTCCAGGAAAAGTCCCTAAGCTTCAGGTATTGTAAAACTGTACTATAAACCGACACAGGTGGACA 1633

NZ_CDBG01000001.1:179744-184090(+) GGGTTCTTTGGCAAATCCGGGAACTCAATTCCGAGACATGATGACGAAGTACGAACTTGTTCGTGCAAAGTGATTGATGCCACGCTTCCAGGAAAAGTCCCTAAGCTTCAGGTATTGTAAAACTGTACTATAAACCGACACAGGTGGACA 1633

NZ_CP103434.1:167622-171961(+) GGGTTCTTTGGCAAATCCGGGAACTCAATTCCGAGACATGATGACGAAGTACGAACTTGTTCGTGCAAAGTGATTGATGCCACGCTTCCAGGAAAAGTCCCTAAGCTTCAGGTATTGTAAAACCGTACTATAAACCGACACAGGTGGACA 1633

CP014354.1:76692-80603(-) GGGTTCTTTGGCAAATCCGGGAACTCAATTCCGAGACATGATGACGAAGTACGAACTTGTTCGTGCAAAGTGATTGATGCCACGCTTCCAGGAAAAGTCCCTAAGCTTCAGGTATTGTAAAACCGTACTATAAACCGACACAGGTGGACA 1164

NOLR01000002.1:131833-136207(+) GGGTTCTTTGGCAAATCCGGGAACTCAATTCCGAGACATGATGACGAAGTACGAACTTGTTCGTGCAAAGTGATTGATGCCACGCTTCCAGGAAAAGTCCCTAAGCTTCAGGTATTGTAAAACCGTACTATAAACCGACACAGGTGGACA 1643

PPFR01000008.1:34360-38734(-) GGGTTCTTTGGCAAATCCGGGAACTCAATTCCGAGACATGATGACGAAGTACGAACTTGTTCGTGCAAAGTGATTGATGCCACGCTTCCAGGAAAAGTCCCTAAGCTTCAGGTATTGTAAAACCGTACTATAAACCGACACAGGTGGACA 1643

PPFQ01000003.1:131083-135464(+) GGGTTCTTTGGCAAATCCGGGAACTCAATTCCGAGACATGATGACGAAGTACGAACTTGTTCGTGCAAAGTGATTGATGCCACGCTTCCAGGAAAAGTCCCTAAGCTTCAGGTATTGTAAAACCGTACTATAAACCGACACAGGTGGACA 1643

NZ_AKYP01000181.1:1647-6020(-) GGGTTCTTTGGCAAATCCGGGAACTCAATTCCGAGACATGATGACGAAGTACGAACTTGTTCGTGCAAAGTGATTGATGCCACGCTTCCAGGAAAAGTCCCTAAGCTTCAGGTATTGTAAAACCGTACTATAAACCGACACAGGTGGACA 1642

NOVI01000003.1:105955-110328(+) GGGTTCTTTGGCAAATCCGGGAACTCAATTCCGAGACATGATGACGAAGTACGAACTTGTTCGTGCAAAGTGATCGATGCCACGCTTCCAGGAAAAGTCCCTAAGCTTCAGGTATTGTAAAACCGTACTATAAACCGACACAGGTGGACA 1643

CP018150.1:339294-343661(+) GGGTTCTTTGGCAAATCCGGGAACTCAATTCCGAGACATGATGACGAAGTACGAACTTGTTCGTGCAAAGTGATTGATGCCACGCTTCCAGGAAAAGTCCCTAAGCTTCAGGTATTGTAAAACTGTACTATAAACCGACACAGGTGGACA 1643

Consensus GGTAGAGAATACCAAGGCGCTTGAGAGAACTTGGGTGAAGGAACTAGGCAAAATGGCACCGTAACTTCGGGAGAAGGTGCGCCCTTGGTAAGTGAAGGTCCTTGCGACTGGAGCTGAAAAGGGTTGCAGATACCAGGTGGCTGCGACTGT 1793

JAKFBC010000002.1:34496-38842(-) (45) GGTAGAGAATACCAAGGCGCTTGAGAGAACTTGGGTGAAGGAACTAGGCAAAATGGCACCGTAACTTCGGGAGAAGGTGCGCCCTTGGTAAGTGAAGGTCCTTGCGACTGGAGCTGAAAAGGGTTGCAGATACCAGGTGGCTGCGACTGT 1783

NZ_AP019757.1:1739264-1743610(-) (31) GGTAGAGAATACCAAGGCGCTTGAGAGAACTTGGGTGAAGGAACTAGGCAAAATGGCACCGTAACTTCGGGAGAAGGTGCGCCCTTGGTAAGTGAAGGTCCTTGCGACTGGAGCTGAAAAGGGTTGCAGATACCAGGTGGCTGCGACTGT 1783

JBKOHN010000001.1:81530-85875(+) (25) GGTAGAGAATACCAAGGCGCTTGAGAGAACTTGGGTGAAGGAACTAGGCAAAATGGCACCGTAACTTCGGGAGAAGGTGCGCCCTTGGTAAGTGAAGGTCCTTGCGACTGGAGCTGAAAAGGGTTGCAGATACCAGGTGGCTGCGACTGT 1783

NC_011528.1:339307-343646(+) (14) GGTAGAGAATACCAAGGCGCTTGAGAGAACTTGGGTGAAGGAACTAGGCAAAATGGCACCGTAACTTCGGGAGAAGGTGCGCCCTTGGTAAGTGAAGGTCCTTGCGACTGGAGCTGAAAAGGGTTGCAGATACCAGGTGGCTGCGACTGT 1783

CP018005.1:167566-171940(+) (14) GGTAGAGAATACCAAGGCGCTTGAGAGAACTTGGGTGAAGGAACTAGGCAAAATGGCACCGTAACTTCGGGAGAAGGTGCGCCCTTGGTAAGTGAAGGTCCTTGCGACTGGAGCTGAAAAGGGTTGCAGATACCAGGTGGCTGCGACTGT 1793

NZ_CCAM010000005.1:71418-75764(+) (13) GGTAGAGAATACCAAGGCGCTTGAGAGAACTTGGGTGAAGGAACTAGGCAAAATGGCACCGTAACTTCGGGAGAAGGTGCGCCCTTGGTAAGTGAAGGTCCTTGCGACTGGAGCTGAAAAGGGTTGCAGATACCAGGTGGCTGCGACTGT 1783

NZ_CCXO01000001.1:317226-321572(+) (11) GGTAGAGAATACCAAGGCGCTTGAGAGAACTTGGGTGAAGGAACTAGGCAAAATGGCACCGTAACTTCGGGAGAAGGTGCGCCCTTGGTAAGTGAAGGTCCTTGCGACTGGAGCTGAAAAGGGTTGCAGATACCAGGTGGCTGCGACTGT 1783

NC_011527.1:1751498-1755844(-) (8) GGTAGAGAATACCAAGGCGCTTGAGAGAACTTGGGTGAAGGAACTAGGCAAAATGGCACCGTAACTTCGGGAGAAGGTGCGCCCTTGGTAAGTGAAGGTCCTTGCGACTGGAGCTGAAAAGGGTTGCAGATACCAGGTGGCTGCGACTGT 1783

NZ_CP032542.1:88036-92382(-) (8) GGTAGAGAATACCAAGGCGCTTGAGAGAACTTGGGTGAAGGAACTAGGCAAAATGGCACCGTAACTTCGGGAGAAGGTGCGCCCTTGGTAAGTGAAGGTCCTTGCGACTGGAGCTGAAAAGGGTTGCAGATACCAGGTGGCTGCGACTGT 1783

CP014551.1:161236-165610(+) (8) GGTAGAGAATACCAAGGCGCTTGAGAGAACTTGGGTGAAGGAACTAGGCAAAATGGCACCGTAACTTCGGGAGAAGGTGCGCCCTTGGTAAGTGAAGGTCCTTGCGACTGGAGCTGAAAAGGGTTGCAGATACCAGGTGGCTGCGACTGT 1793

CP013667.1:88011-92385(-) (8) GGTAGAGAATACCAAGGCGCTTGAGAGAACTTGGGTGAAGGAACTAGGCAAAATGGCACCGTAACTTCGGGAGAAGGTGCGCCCTTGGTAAGTGAAGGTCCTTGCGACTGGAGCTGAAAAGGGTTGCAGATACCAGGTGGCTGCGACTGT 1793

CP107247.1:164350-168689(+) (7) GGTAGAGAATACCAAGGCGCTTGAGAGAACTTGGGTGAAGGAACTAGGCAAAATGGCACCGTAACTTCGGGAGAAGGTGCGCCCTTGGTAAGTGAAGGTCCTTGCGACTGGAGCTGAAAAGGGTTGCAGATACCAGGTGGCTGCGACTGT 1783

NZ_CP103435.1:167581-171920(+) (6) GGTAGAGAATACCAAGGCGCTTGAGAGAACTTGGGTGAAGGAACTAGGCAAAATGGCACCGTAACTTCGGGAGAAGGTGCGCCCTTGGTAAGTGAAGGTCCTTGCGACTGGAGCTGAAAAGGGTTGCAGATACCAGGTGGCTGCGACTGT 1783

CP014565.1:1711747-1716121(-) (3) GGTAGAGAATACCAAGGCGCTTGAGAGAACTTGGGTGAAGGAACTAGGCAAAATGGCACCGTAACTTCGGGAGAAGGTGCGCCCTTGGTAAGTGAAGGTCCTTGCGACTGGAGCTGAAAAGGGTTGCAGATACCAGGTGGCTGCGACTGT 1793

AAYJ01000007.1:0-1404(+) (3) ------------------------------------------------------------------------------------------------------------------------------------------------------

NZ_JAOXDR010000002.1:16672-21025(-) (3) GGTAGAGAATACCAAGGCGCTTGAGAGAACTTGGGTGAAGGAACTAGGCAAAATGGCACCGTAACTTCGGGAGAAGGTGCGCCCTTGGTAAGTGAAGGTCCTTGCGACTGGAGCTGAAAAGGGTTGCAGATACCAGGTGGCTGCGACTGT 1783

NZ_CP103432.1:167699-172038(+) (3) GGTAGAGAATACCAAGGCGCTTGAGAGAACTTGGGTGAAGGAACTAGGCAAAATGGCACCGTAACTTCGGGAGAAGGTGCGCCCTTGGTAAGTGAAGGTCCTTGCGACTGGAGCTGAAAAGGGTTGCAGATACCAGGTGGCTGCGACTGT 1783

NZ_CP103431.1:167697-172036(+) (3) GGTAGAGAATACCAAGGCGCTTGAGAGAACTTGGGTGAAGGAACTAGGCAAAATGGCACCGTAACTTTGGGAGAAGGTGCGCCCTTGGTAAGTGAAGGTCCTTGCGACTGGAGCTGAAAAGGGTTGCAGATACCAGGTGGCTGCGACTGT 1783

NZ_CP103428.1:167591-171929(+) (2) GGTAGAGAATACCAAGGCGCTTGAGAGAACTTGGGTGAAGGAACTAGGCAAAATGGCACCGTAACTTCGGGAGAAGGTGCGCCCTTGGTAAGTGAAGGTCCTTGCGACTGGAGCTGAAAAGGGTTGCAGATACCAGGTGGCTGCGACTGT 1783

NZ_JANTNR010000002.1:115350-119696(+) (2) GGTAGAGAATACCAAGGCGCTTGAGAGAACTTGGGTGAAGGAACTAGGCAAAATGGCACCGTAACTTCGGGAGAAGGTGCGCCCTTGGTAAGTGAAGGTCCTTGCGACTGGAGCTGAAAAGGGTTGCAGATACCAGGTGGCTGCGACTGT 1783

JARBIR010004564.1:3782-8128(-) (2) GGTAGAGAATACCAAGGCGCTTGAGAGAACTTGGGTGAAGGAACTAGGCAAAATGGCACCGTAACTTCGGGAGAAGGTGCGCCCTTGGTAAGTGAAGGTCCTTGCGACTGGAGCTGAAAAGGGTTGCAGATACCAGGTGGCTGCGACTGT 1783

NC_009727.1:1888349-1892695(-) (2) GGTAGAGAATACCAAGGCGCTTGAGAGAACTTGGGTGAAGGAACTAGGCAAAATGGCACCGTAACTTCGGGAGAAGGTGCGCCCTTGGTAAGTGAAGGTCCTTGCGACTGGAGCTGAAAAGGGTTGCAGATACCAGGTGGCTGCGACTGT 1783

AAYJ01000139.1:0-660(+) (2) GGTAGAGAATACCAAGGCGCTTGAGAGAACTTGGGTGAAGGAACTAGGCAAAATGGCACCGTAACTTCGGGAGAAGGTGCGCCCTTGGTAAGTGAAGGTCCTTGCGACTGGAGCTGAAAAGGGTTGCAGATACCA--------------- 609

CP032542.1:88018-92392(-) (2) GGTAGAGAATACCAAGGCGCTTGAGAGAACTTGGGTGAAGGAACTAGGCAAAATGGCACCGTAACTTCGGGAGAAGGTGCGCCCTTGGTAAGTGAAGGTCCTTGCGACTGGAGCTGAAAAGGGTTGCAGATACCAGGTGGCTGCGACTGT 1793

CP014559.1:232744-237117(+) (2) GGTAGAGAATACCAAGGCGCTTGAGAGAACTTGGGTGAAGGAACTAGGCAAAATGGCACCGTAACTTCGGGAGAAGGTGCGCCCTTGGTAAGTGAAGGTCCTTGCGACTGGAGCTGAAAAGGGTTGCAGATACCAGGTGGCTGCGACTGT 1793

CP007555.1:337408-341752(+) GGTAGAGAATACCAAGGCGCTTGAGAGAACTTGGGTGAAGGAACTAGGCAAAATGGCACCGTAACTTCGGGAGAAGGTGCGCCCTTGGTAAGTGAAGGTCCTTGCGACTGGAGCTGAAAAGGGTTGCAGATACCAGGTGGCTGCGACTGT 1781

NZ_CP014354.1:76710-81178(-) GGTAGAGAATACCAAGGCGCTTGAGAGAACTTGGGTGAAGGAACTAGGCAAAATGGCACCGTAACTTCGGGAGAAGGTGCGCCCTTGGTAAGTGAAGGTCCTTGCGACTGGAGCTGAAAAGGGTTGCAGATACCAGGTGGCTGCGACTGT 1789

CP000890.1:243129-244433(+) GGTAGAGAATACCAAGGCGCTTGAGAGAACTTGGGTGAAGGAACTAGGCAAAATGGCACCGTAACTTCGGGAGAAGGTGCGCCCTTGGTAAGTGAAGGTCCTTGCGACTGGAGCTGAAAAGGGTTGCAGATACCAGGTGGCTGCGACTGT 609

NZ_CP115461.1:167576-171922(+) GGTAGAGAATACCAAGGCGCTTGAGAGAACTTGGGTGAAGGAACTAGGCAAAATGGCACCGTAACTTCGGGAGAAGGTGCGCCCTTGGTAAGTGAAGGTCCTTGCGACTGGAGCTGAAAAGGGTTGCAGATACCAGGTGGCTGCGACTGT 1783

AP019759.1:167578-171919(+) GGTAGAGAATACCAAGGCGCTTGAGAGAACTTGGGTGAAGGAACTAGGCAAAATGGCACCGTAACTTCGGGAGAAGGTGCGCCCTTGGTAAGTGAAGGTCCTTGCGACTGGAGCTGAAAAGGGTTGCAGATACCAGGTGGCTGCGACTGT 1781

AP019757.1:1739267-1743608(-) GGTAGAGAATACCAAGGCGCTTGAGAGAACTTGGGTGAAGGAACTAGGCAAAATGGCACCGTAACTTCGGGAGAAGGTGCGCCCTTGGTAAGTGAAGGTCCTTGCGACTGGAGCTGAAAAGGGTTGCAGATACCAGGTGGCTGCGACTGT 1781

NOLN01000021.1:90861-95235(-) GGTAGAGAATACCAAGGCGCTTGAGAGAACTTGGGTGAAGGAACTAGGCAAAATGGCACCGTAACTTCGGGAGAAGGTGCGCCCTTGGTAAGTGAAGGTCCTTGCGACTGGAGCTGAAAAGGGTTGCAGATACCAGGTGGCTGCGACTGT 1793

NOLM01000022.1:91202-95576(-) GGTAGAGAATACCAAGGCGCTTGAGAGAACTTGGGTGAAGGAACTAGGCAAAATGGCACCGTAACTTCGGGAGAAGGTGCGCCCTTGGTAAGTGAAGGTCCTTGCGACTGGAGCTGAAAAGGGTTGCAGATACCAGGTGGCTGCGACTGT 1793

PDLP01000011.1:14431-18805(+) GGTAGAGAATACCAAGGCGCTTGAGAGAACTTGGGTGAAGGAACTAGGCAAAATGGCACCGTAACTTCGGGAGAAGGTGCGCCCTTGGTAAGTGAAGGTCCTTGCGACTGGAGCTGAAAAGGGTTGCAGATACCAGGTGGCTGCGACTGT 1793

CP014563.1:522940-527314(+) GGTAGAGAATACCAAGGCGCTTGAGAGAACTTGGGTGAAGGAACTAGGCAAAATGGCACCGTAACTTCGGGAGAAGGTGCGCCCTTGGTAAGTGAAGGTCCTTGCGACTGGAGCTGAAAAGGGTTGCAGATACCAGGTGGCTGCGACTGT 1793

CP000890.1:244922-245905(+) ------------------------------------------------------------------------------------------------------------------------------------------------------

NZ_NOLR01000002.1:131843-136189(+) GGTAGAGAATACCAAGGCGCTTGAGAGAACTTGGGTGAAGGAACTAGGCAAAATGGCACCGTAACTTCGGGAGAAGGTGCGCCCTTGGTAAGTGAAGGTCCTTGCGACTGGAGCTGAAAAGGGTTGCAGATACCAGGTGGCTGCGACTGT 1783

NZ_JAOXDP010000002.1:15794-20139(-) GGTAGAGAATACCAAGGCGCTTGAGAGAACTTGGGTGAAGGAACTAGGCAAAATGGCACCGTAACTTCGGGAGAAGGTGCGCCCTTGGTAAGTGAAGGTCCTTGCGACTGGAGCTGAAAAGGGTTGCAGATACCAGGTGGCTGCGACTGT 1783

NZ_PPFR01000008.1:34378-38724(-) GGTAGAGAATACCAAGGCGCTTGAGAGAACTTGGGTGAAGGAACTAGGCAAAATGGCACCGTAACTTCGGGAGAAGGTGCGCCCTTGGTAAGTGAAGGTCCTTGCGACTGGAGCTGAAAAGGGTTGCAGATACCAGGTGGCTGCGACTGT 1783

NZ_PPFQ01000003.1:131093-135446(+) GGTAGAGAATACCAAGGCGCTTGAGAGAACTTGGGTGAAGGAACTAGGCAAAATGGCACCGTAACTTCGGGAGAAGGTGCGCCCTTGGTAAGTGAAGGTCCTTGCGACTGGAGCTGAAAAGGGTTGCAGATACCAGGTGGCTGCGACTGT 1783

NZ_LK937696.1:167613-171953(+) GGTAGAGAATACCAAGGCGCTTGAGAGAACTTGGGTGAAGGAACTAGGCAAAATGGCACCGTAACTTCGGGAGAAGGTGCGCCCTTGGTAAGTGAAGGTCCTTGCGACTGGAGCTGAAAAGGGTTGCAGATACCAGGTGGCTGCGACTGT 1783

NZ_NOVI01000003.1:105965-110310(+) GGTAGAGAATACCAAGGCGCTTGAGAGAACTTGGGTGAAGGAACTAGGCAAAATGGCACCGTAACTTCGGGAGAAGGTGCGCCCTTGGTAAGTGAAGGTCCTTGCGACTGGAGCTGAAAAGGGTTGCAGATACCAGGTGGCTGCGACTGT 1783

NZ_CP103426.1:167642-171981(+) GGTAGAGAATACCAAGGCGCTTGAGAGAACTTGGGTGAAGGAACTAGGCAAAATGGCACCGTAACTTCGGGAGAAGGTGCGCCCTTGGTAAGTGAAGGTCCTTGCGACTGGAGCTGAAAAGGGTTGCAGATACCAGGTGGCTGCGACTGT 1783

NZ_JAOXDN010000007.1:36985-41324(-) GGTAGAGAATACCAAGGCGCTTGAGAGAACTTGGGTGAAGGAACTAGGCAAAATGGCACCGTAACTTCGGGAGAAGGTGCGCCCTTGGTAAGTGAAGGTCCTTGCGACTGGAGCTGAAAAGGGTTGCAGATACCAGGTGGCTGCGACTGT 1783

NZ_JPVV01000013.1:13888-18051(+) GGTAGAGAATACCAAGGCGCTTGAGAGAACTTGGGTGAAGGAACTAGGCAAAATGGCACCGTAACTTCGGGAGAAGGTGCGCCCTTGGTAAGTGAAGGTCCTTGCGACTGGAGCTGAAAAGGGTTGCAGATACCAGGTGGCTGCGACTGT 1783

NZ_CP007555.1:337406-341752(+) GGTAGAGAATACCAAGGCGCTTGAGAGAACTTGGGTGAAGGAACTAGGCAAAATGGCACCGTAACTTCGGGAGAAGGTGCGCCCTTGGTAAGTGAAGGTCCTTGCGACTGGAGCTGAAAAGGGTTGCAGATACCAGGTGGCTGCGACTGT 1783

NZ_JASNNV010000010.1:39284-43631(-) GGTAGAGAATACCAAGGCGCTTGAGAGAACTTGGGTGAAGGAACTAGGCAAAATGGCACCGTAACTTCGGGAGAAGGTGCGCCCTTGGTAAGTGAAGGTCCTTGCGACTGGAGCTGAAAAGGGTTGCAGATACCAGGTGGCTGCGACTGT 1783

NZ_JAOXFC010000012.1:14231-18577(+) GGTAGAGAATACCAAGGCGCTTGAGAGAACTTGGGTGAAGGAACTAGGCAAAATGGCACCGTAACTTCGGGAGAAGGTGCGCCCTTGGTAAGTGAAGGTCCTTGCGACTGGAGCTGAAAAGGGTTGCAGATACCAGGTGGCTGCGACTGT 1783

NZ_CP103430.1:167658-171997(+) GGTAGAGAATACCAAGGCGCTTGAGAGAACTTGGGTGAAGGAACTAGGCAAAATGGCACCGTAACTTCGGGAGAAGGTGCGCCCTTGGTAAGTGAAGGTCCTTGCGACTGGAGCTGAAAAGGGTTGCAGATACCAGGTGGCTGCGACTGT 1783

NZ_PDLP01000011.1:14441-18787(+) GGTAGAGAATACCAAGGCGCTTGAGAGAACTTGGGTGAAGGAACTAGGCAAAATGGCACCGTAACTTCGGGAGAAGGTGCGCCCTTGGTAAGTGAAGGTCCTTGCGACTGGAGCTGAAAAGGGTTGCAGATACCAGGTGGCTGCGACTGT 1783

NZ_NOLM01000022.1:91220-95566(-) GGTAGAGAATACCAAGGCGCTTGAGAGAACTTGGGTGAAGGAACTAGGCAAAATGGCACCGTAACTTCGGGAGAAGGTGCGCCCTTGGTAAGTGAAGGTCCTTGCGACTGGAGCTGAAAAGGGTTGCAGATACCAGGTGGCTGCGACTGT 1783

JBJCIS010000056.1:0-2780(-) GGTAGAGAATACCAAGGCGCTTGAGAGAACTTGGGTGAAGGAACTAGGCAAAATGGCACCGTAACTTCGGGAGAAGGTGCGCCCTTGGTAAGTGAAGGTCCTTGCGACTGGAGCTGAAAAGGGTTGCAGATACCAGGTGGCTGCGACTGT 1783

NZ_CDBG01000001.1:179744-184090(+) GGTAGAGAATACCAAGGCGCTTGAGAGAACTTGGGTGAAGGAACTAGGCAAAATGGCACCGTAACTTCGGGAGAAGGTGCGCCCTTGGTAAGTGAAGGTCCTTGCGACTGGAGCTGAAAAGGGTTGCAGATACCAGGTGGCTGCGACTGT 1783

NZ_CP103434.1:167622-171961(+) GGTAGAGAATACCAAGGCGCTTGAGAGAACTTGGGTGAAGGAACTAGGCAAAATGGCACCGTAACTTCGGGAGAAGGTGCGCCCTTGGTAAGTGAAGGTCCTTGCGACTGGAGCTGAAAAGGGTTGCAGATACCAGGTGGCTGCGACTGT 1783

CP014354.1:76692-80603(-) GGTAGAGAATACCAAGGCGCTTGAGAGAACTTGGGTGAAGGAACTAGGCAAAATGGCACCGTAACTTCGGGAGAAGGTGCGCCCTTGGTAAGTGAAGGTCCTTGCGACTGGAGCTGAAAAGGGTTGCAGATACCAGGTGGCTGCGACTGT 1314

NOLR01000002.1:131833-136207(+) GGTAGAGAATACCAAGGCGCTTGAGAGAACTTGGGTGAAGGAACTAGGCAAAATGGCACCGTAACTTCGGGAGAAGGTGCGCCCTTGGTAAGTGAAGGTCCTTGCGACTGGAGCTGAAAAGGGTTGCAGATACCAGGTGGCTGCGACTGT 1793

PPFR01000008.1:34360-38734(-) GGTAGAGAATACCAAGGCGCTTGAGAGAACTTGGGTGAAGGAACTAGGCAAAATGGCACCGTAACTTCGGGAGAAGGTGCGCCCTTGGTAAGTGAAGGTCCTTGCGACTGGAGCTGAAAAGGGTTGCAGATACCAGGTGGCTGCGACTGT 1793

PPFQ01000003.1:131083-135464(+) GGTAGAGAATACCAAGGCGCTTGAGAGAACTTGGGTGAAGGAACTAGGCAAAATGGCACCGTAACTTCGGGAGAAGGTGCGCCCTTGGTAAGTGAAGGTCCTTGCGACTGGAGCTGAAAAGGGTTGCAGATACCAGGTGGCTGCGACTGT 1793

NZ_AKYP01000181.1:1647-6020(-) GGTAGAGAATACCAAGGCGCTTGAGAGAACTTGGGTGAAGGAACTAGGCAAAATGGCACCGTAACTTCGGGAGAAGGTGCGCCCTTGGTAAGTGAAGGTCCTTGCGACTGGAGCTGAAAAGGGTTGCAGATACCAGGTGGCTGCGACTGT 1792

NOVI01000003.1:105955-110328(+) GGTAGAGAATACCAAGGCGCTTGAGAGAACTTGGGTGAAGGAACTAGGCAAAATGGCACCGTAACTTCGGGAGAAGGTGCGCCCTTGGTAAGTGAAGGTCCTTGCGACTGGAGCTGAAAAGGGTTGCAGATACCAGGTGGCTGCGACTGT 1793

CP018150.1:339294-343661(+) GGTAGAGAATACCAAGGCGCTTGAGAGAACTTGGGTGAAGGAACTAGGCAAAATGGCACCGTAACTTCGGGAGAAGGTGCGCCCTTGGTAAGTGAAGGTCCTTGCGACTGGAGCTGAAAAGGGTTGCAGATACCAGGTGGCTGCGACTGT 1793

Consensus TTACTAAAAACACAGCACTCTGCAAACTCGTAAGAGGACGTATAGGGTGTGACGCCTGCCCGGTGCCGGAAGGTTAAGTGATGGGGTTAGCCCTCGGGCGAAGCTCTTGATCGAAGCCCCGGTAAACGGCGGCCGTAACTATAACGGTCC 1943

JAKFBC010000002.1:34496-38842(-) (45) TTACTAAAAACACAGCACTCTGCAAACTCGTAAGAGGACGTATAGGGTGTGACGCCTGCCCGGTGCCGGAAGGTTAAGTGATGGGGTTAGCCCTCGGGCGAAGCTCTTGATCGAAGCCCCGGTAAACGGCGGCCGTAACTATAACGGTCC 1933

NZ_AP019757.1:1739264-1743610(-) (31) TTACTAAAAACACAGCACTCTGCAAACTCGTAAGAGGACGTATAGGGTGTGACGCCTGCCCGGTGCCGGAAGGTTAAGTGATGGGGTTAGCCCTCGGGCGAAGCTCTTGATCGAAGCCCCGGTAAACGGCGGCCGTAACTATAACGGTCC 1933

JBKOHN010000001.1:81530-85875(+) (25) TTACTAAAAACACAGCACTCTGCAAACTCGTAAGAGGACGTATAGGGTGTGACGCCTGCCCGGTGCCGGAAGGTTAAGTGATGGGGTTAGCCCTCGGGCGAAGCTCTTGATCGAAGCCCCGGTAAACGGCGGCCGTAACTATAACGGTCC 1933

NC_011528.1:339307-343646(+) (14) TTACTAAAAACACAGCACTCTGCAAACTCGTAAGAGGACGTATAGGGTGTGACGCCTGCCCGGTGCCGGAAGGTTAAGTGATGGGGTTAGCCCTCGGGCGAAGCTCTTGATCGAAGCCCCGGTAAACGGCGGCCGTAACTATAACGGTCC 1933

CP018005.1:167566-171940(+) (14) TTACTAAAAACACAGCACTCTGCAAACTCGTAAGAGGACGTATAGGGTGTGACGCCTGCCCGGTGCCGGAAGGTTAAGTGATGGGGTTAGCCCTCGGGCGAAGCTCTTGATCGAAGCCCCGGTAAACGGCGGCCGTAACTATAACGGTCC 1943

NZ_CCAM010000005.1:71418-75764(+) (13) TTACTAAAAACACAGCACTCTGCAAACTCGTAAGAGGACGTATAGGGTGTGACGCCTGCCCGGTGCCGGAAGGTTAAGTGATGGGGTTAGCCCTCGGGCGAAGCTCTTGATCGAAGCCCCGGTAAACGGCGGCCGTAACTATAACGGTCC 1933

NZ_CCXO01000001.1:317226-321572(+) (11) TTACTAAAAACACAGCACTCTGCAAACTCGTAAGAGGACGTATAGGGTGTGACGCCTGCCCGGTGCCGGAAGGTTAAGTGATGGGGTTAGCCCTCGGGCGAAGCTCTTGATCGAAGCCCCGGTAAACGGCGGCCGTAACTATAACGGTCC 1933

NC_011527.1:1751498-1755844(-) (8) TTACTAAAAACACAGCACTCTGCAAACTCGTAAGAGGACGTATAGGGTGTGACGCCTGCCCGGTGCCGGAAGGTTAAGTGATGGGGTTAGCCCTCGGGCGAAGCTCTTGATCGAAGCCCCGGTAAACGGCGGCCGTAACTATAACGGTCC 1933

NZ_CP032542.1:88036-92382(-) (8) TTACTAAAAACACAGCACTCTGCAAACTCGTAAGAGGACGTATAGGGTGTGACGCCTGCCCGGTGCCGGAAGGTTAAGTGATGGGGTTAGCCCTCGGGCGAAGCTCTTGATCGAAGCCCCGGTAAACGGCGGCCGTAACTATAACGGTCC 1933

CP014551.1:161236-165610(+) (8) TTACTAAAAACACAGCACTCTGCAAACTCGTAAGAGGACGTATAGGGTGTGACGCCTGCCCGGTGCCGGAAGGTTAAGTGATGGGGTTAGCCCTCGGGCGAAGCTCTTGATCGAAGCCCCGGTAAACGGCGGCCGTAACTATAACGGTCC 1943

CP013667.1:88011-92385(-) (8) TTACTAAAAACACAGCACTCTGCAAACTCGTAAGAGGACGTATAGGGTGTGACGCCTGCCCGGTGCCGGAAGGTTAAGTGATGGGGTTAGCCCTCGGGCGAAGCTCTTGATCGAAGCCCCGGTAAACGGCGGCCGTAACTATAACGGTCC 1943

CP107247.1:164350-168689(+) (7) TTACTAAAAACACAGCACTCTGCAAACTCGTAAGAGGACGTATAGGGTGTGACGCCTGCCCGGTGCCGGAAGGTTAAGTGATGGGGTTAGCCCTCGGGCGAAGCTCTTGATCGAAGCCCCGGTAAACGGCGGCCGTAACTATAACGGTCC 1933

NZ_CP103435.1:167581-171920(+) (6) TTACTAAAAACACAGCACTCTGCAAACTCGTAAGAGGACGTATAGGGTGTGACGCCTGCCCGGTGCCGGAAGGTTAAGTGATGGGGTTAGCCCTCGGGCGAAGCTCTTGATCGAAGCCCCGGTAAACGGCGGCCGTAACTATAACGGTCC 1933

CP014565.1:1711747-1716121(-) (3) TTACTAAAAACACAGCACTCTGCAAACTCGTAAGAGGACGTATAGGGTGTGACGCCTGCCCGGTGCCGGAAGGTTAAGTGATGGGGTTAGCCCTCGGGCGAAGCTCTTGATCGAAGCCCCGGTAAACGGCGGCCGTAACTATAACGGTCC 1943

AAYJ01000007.1:0-1404(+) (3) ------------------------------------------------------------------------------------------------------------------------------------------------------

NZ_JAOXDR010000002.1:16672-21025(-) (3) TTACTAAAAACACAGCACTCTGCAAACTCGTAAGAGGACGTATAGGGTGTGACGCCTGCCCGGTGCCGGAAGGTTAAGTGATGGGGTTAGCCCTCGGGCGAAGCTCTTGATCGAAGCCCCGGTAAACGGCGGCCGTAACTATAACGGTCC 1933

NZ_CP103432.1:167699-172038(+) (3) TTACTAAAAACACAGCACTCTGCAAACTCGTAAGAGGACGTATAGGGTGTGACGCCTGCCCGGTGCCGGAAGGTTAAGTGATGGGGTTAGCCCTCGGGCGAAGCTCTTGATCGAAGCCCCGGTAAACGGCGGCCGTAACTATAACGGTCC 1933

NZ_CP103431.1:167697-172036(+) (3) TTACTAAAAACACAGCACTCTGCAAACTCGTAAGAGGACGTATAGGGTGTGACGCCTGCCCGGTGCCGGAAGGTTAAGTGATGGGGTTAGCCCTCGGGCGAAGCTCTTGATCGAAGCCCCGGTAAACGGCGGCCGTAACTATAACGGTCC 1933

NZ_CP103428.1:167591-171929(+) (2) TTACTAAAAACACAGCACTCTGCAAACTCGTAAGAGGACGTATAGGGTGTGACGCCTGCCCGGTGCCGGAAGGTTAAGTGATGGGGTTAGCCCTCGGGCGAAGCTCTTGATCGAAGCCCCGGTAAACGGCGGCCGTAACTATAACGGTCC 1933

NZ_JANTNR010000002.1:115350-119696(+) (2) TTACTAAAAACACAGCACTCTGCAAACTCGTAAGAGGACGTATAGGGTGTGACGCCTGCCCGGTGCCGGAAGGTTAAGTGATGGGGTTAGCCCTCGGGCGAAGCTCTTGATCGAAGCCCCGGTAAACGGCGGCCGTAACTATAACGGTCC 1933

JARBIR010004564.1:3782-8128(-) (2) TTACTAAAAACACAGCACTCTGCAAACTCGTAAGAGGACGTATAGGGTGTGACGCCTGCCCGGTGCCGGAAGGTTAAGTGATGGGGTTAGCCCTCGGGCGAAGCTCTTGATCGAAGCCCCGGTAAACGGCGGCCGTAACTATAACGGTCC 1933

NC_009727.1:1888349-1892695(-) (2) TTACTAAAAACACAGCACTCTGCAAACTCGTAAGAGGACGTATAGGGTGTGACGCCTGCCCGGTGCCGGAAGGTTAAGTGATGGGGTTAGCCCTCGGGCGAAGCTCTTGATCGAAGCCCCGGTAAACGGCGGCCGTAACTATAACGGTCC 1933

AAYJ01000139.1:0-660(+) (2) ------------------------------------------------------------------------------------------------------------------------------------------------------ 759

CP032542.1:88018-92392(-) (2) TTACTAAAAACACAGCACTCTGCAAACTCGTAAGAGGACGTATAGGGTGTGACGCCTGCCCGGTGCCGGAAGGTTAAGTGATGGGGTTAGCCCTCGGGCGAAGCTCTTGATCGAAGCCCCGGTAAACGGCGGCCGTAACTATAACGGTCC 1943

CP014559.1:232744-237117(+) (2) TTACTAAAAACACAGCACTCTGCAAACTCGTAAGAGGACGTATAGGGTGTGACGCCTGCCCGGTGCCGGAAGGTTAAGTGATGGGGTTAGCCCTCGGGCGAAGCTCTTGATCGAAGCCCCGGTAAACGGCGGCCGTAACTATAACGGTCC 1943

CP007555.1:337408-341752(+) TTACTAAAAACACAGCACTCTGCAAACTCGTAAGAGGACGTATAGGGTGTGACGCCTGCCCGGTGCCGGAAGGTTAAGTGATGGGGTTAGCCCTCGGGCGAAGCTCTTGATCGAAGCCCCGGTAAACGGCGGCCGTAACTATGACGGTCC 1931

NZ_CP014354.1:76710-81178(-) TTACTAAAAACACAGCACTCTGCAAACTCGTAAGAGGACGTATAGGGTGTGACGCCTGCCCGGTGCCGGAAGGTTAAGTGATGGGGTTAGCCCTCGGGCGAAGCTCTTGATCGAAGCCCCGGTAAACGGCGGCCGTAACTATAACGGTCC 1939

CP000890.1:243129-244433(+) TTACTAAAAACACAGCACTCTGCAAACTCGTAAGAGGACGTATAGGGTGTGACGCCTGCCCGGTGCCGGAAGGTTAAGTGATGGGGTTAGCCCTCGGGCGAAGCTCTTGATCGAAGCCCCGGTAAACGGCGGCCGTAACTATAACGGTCC 759

NZ_CP115461.1:167576-171922(+) TTACTAAAAACACAGCACTCTGCAAACTCGTAAGAGGACGTATAGGGTGTGACGCCTGCCCGGTGCCGGAAGGTTAAGTGATGGGGTTAGCCCTCGGGCGAAGCTCTTGATCGAAGCCCCGGTAAACGGCGGCCGTAACTATAACGGTCC 1933

AP019759.1:167578-171919(+) TTACTAAAAACACAGCACTCTGCAAACTCGTAAGAGGACGTATAGGGTGTGACGCCTGCCCGGTGCCGGAAGGTTAAGTGATGGGGTTAGCCCTCGGGCGAAGCTCTTGATCGAAGCCCCGGTAAACGGCGGCCGTAACTATAACGGTCC 1931

AP019757.1:1739267-1743608(-) TTACTAAAAACACAGCACTCTGCAAACTCGTAAGAGGACGTATAGGGTGTGACGCCTGCCCGGTGCCGGAAGGTTAAGTGATGGGGTTAGCCCTCGGGCGAAGCTCTTGATCGAAGCCCCGGTAAACGGCGGCCGTAACTATAACGGTCC 1931

NOLN01000021.1:90861-95235(-) TTACTAAAAACACAGCACTCTGCAAACTCGTAAGAGGACGTATAGGGTGTGACGCCTGCCCGGTGCCGGAAGGTTAAGTGATGGGGTTAGCCCTCGGGCGAAGCTCTTGATCGAAGCCCCGGTAAACGGCGGCCGTAACTATAACGGTCC 1943

NOLM01000022.1:91202-95576(-) TTACTAAAAACACAGCACTCTGCAAACTCGTAAGAGGACGTATAGGGTGTGACGCCTGCCCGGTGCCGGAAGGTTAAGTGATGGGGTTAGCTCTCGGGCGAAGCTCTTGATCGAAGCCCCGGTAAACGGCGGCCGTAACTATAACGGTCC 1943

PDLP01000011.1:14431-18805(+) TTACTAAAAACACAGCACTCTGCAAACTCGTAAGAGGACGTATAGGGTGTGACGCCTGCCCGGTGCCGGAAGGTTAAGTGATGGGGTTAGCCCTCGGGCGAAGCTCTTGATCGAAGCCCCGGTAAACGGCGGCCGTAACTATAACGGTCC 1943

CP014563.1:522940-527314(+) TTACTAAAAACACAGCACTCTGCAAACTCGTAAGAGGACGTATAGGGTGTGACGCCTGCCCGGTGCCGGAAGGTTAAGTGATGGGGTTAGCCCTCGGGCGAAGCTCTTGATCGAAGCCCCGGTAAACGGCGGCCGTAACTATAACGGTCC 1943

CP000890.1:244922-245905(+) ------------------------------------------------------------------------------------------------------------------------------------------------------

NZ_NOLR01000002.1:131843-136189(+) TTACTAAAAACACAGCACTCTGCAAACTCGTAAGAGGACGTATAGGGTGTGACGCCTGCCCGGTGCCGGAAGGTTAAGTGATGGGGTTAGCCCTCGGGCGAAGCTCTTGATCGAAGCCCCGGTAAACGGCGGCCGTAACTATAACGGTCC 1933

NZ_JAOXDP010000002.1:15794-20139(-) TTACTAAAAACACAGCACTCTGCAAACTCGTAAGAGGACGTATAGGGTGTGACGCCTGCCCGGTGCCGGAAGGTTAAGTGATGGGGTTAGCCCTCGGGCGAAGCTCTTGATCGAAGCCCCGGTAAACGGCGGCCGTAACTATAACGGTCC 1933

NZ_PPFR01000008.1:34378-38724(-) TTACTAAAAACACAGCACTCTGCAAACTCGTAAGAGGACGTATAGGGTGTGACGCCTGCCCGGTGCCGGAAGGTTAAGTGATGGGGTTAGCCCTCGGGCGAAGCTCTTGATCGAAGCCCCGGTAAACGGCGGCCGTAACTATAACGGTCC 1933

NZ_PPFQ01000003.1:131093-135446(+) TTACTAAAAACACAGCACTCTGCAAACTCGTAAGAGGACGTATAGGGTGTGACGCCTGCCCGGTGCCGGAAGGTTAAGTGATGGGGTTAGCCCTCGGGCGAAGCTCTTGATCGAAGCCCCGGTAAACGGCGGCCGTAACTATAACGGTCC 1933

NZ_LK937696.1:167613-171953(+) TTACTAAAAACACAGCACTCTGCAAACTCGTAAGAGGACGTATAGGGTGTGACGCCTGCCCGGTGCCGGAAGGTTAAGTGATGGGGTTAGCCCTCGGGCGAAGCTCTTGATCGAAGCCCCGGTAAACGGCGGCCGTAACTATAACGGTCC 1933

NZ_NOVI01000003.1:105965-110310(+) TTACTAAAAACACAGCACTCTGCAAACTCGTAAGAGGACGTATAGGGTGTGACGCCTGCCCGGTGCCGGAAGGTTAAGTGATGGGGTTAGCCCTCGGGCGAAGCTCTTGATCGAAGCCCCGGTAAACGGCGGCCGTAACTATAACGGTCC 1933

NZ_CP103426.1:167642-171981(+) TTACTAAAAACACAGCACTCTGCAAACTCGTAAGAGGACGTATAGGGTGTGACGCCTGCCCGGTGCCGGAAGGTTAAGTGATGGGGTTAGCCCTCGGGCGAAGCTCTTGATCGAAGCCCCGGTAAACGGCGGCCGTAACTATAACGGTCC 1933

NZ_JAOXDN010000007.1:36985-41324(-) TTACTAAAAACACAGCACTCTGCAAACTCGTAAGAGGACGTATAGGGTGTGACGCCTGCCCGGTGCCGGAAGGTTAAGTGATGGGGTTAGCCCTCGGGCGAAGCTCTTGATCGAAGCCCCGGTAAACGGCGGCCGTAACTATAACGGTCC 1933

NZ_JPVV01000013.1:13888-18051(+) TTACTAAAAACACAGCACTCTGCAAACTCGTAAGAGGACGTATAGGGTGTGACGCCTGCCCGGTGCCGGAAGGTTAAGTGATGGGGTTAGCCCTCGGGCGAAGCTCTTGATCGAAGCCCCGGTAAACGGCGGCCGTAACTATAACGGTCC 1933

NZ_CP007555.1:337406-341752(+) TTACTAAAAACACAGCACTCTGCAAACTCGTAAGAGGACGTATAGGGTGTGACGCCTGCCCGGTGCCGGAAGGTTAAGTGATGGGGTTAGCCCTCGGGCGAAGCTCTTGATCGAAGCCCCGGTAAACGGCGGCCGTAACTATGACGGTCC 1933

NZ_JASNNV010000010.1:39284-43631(-) TTACTAAAAACACAGCACTCTGCAAACTCGTAAGAGGACGTATAGGGTGTGACGCCTGCCCGGTGCCGGAAGGTTAAGTGATGGGGTTAGCCCTCGGGCGAAGCTCTTGATCGAAGCCCCGGTAAACGGCGGCCGTAACTATAACGGTCC 1933

NZ_JAOXFC010000012.1:14231-18577(+) TTACTAAAAACACAGCACTCTGCAAACTCGTAAGAGGACGTATAGGGTGTGACGCCTGCCCGGTGCCGGAAGGTTAAGTGATGGGGTTAGCCCTCGGGCGAAGCTCTTGATCGAAGCCCCGGTAAACGGCGGCCGTAACTATAACGGTCC 1933

NZ_CP103430.1:167658-171997(+) TTACTAAAAACACAGCACTCTGCAAACTCGTAAGAGGACGTATAGGGTGTGACGCCTGCCCGGTGCCGGAAGGTTAAGTGATGGGGTTAGCCCTCGGGCGAAGCTCTTGATCGAAGCCCCGGTAAACGGCGGCCGTAACTATAACGGTCC 1933

NZ_PDLP01000011.1:14441-18787(+) TTACTAAAAACACAGCACTCTGCAAACTCGTAAGAGGACGTATAGGGTGTGACGCCTGCCCGGTGCCGGAAGGTTAAGTGATGGGGTTAGCCCTCGGGCGAAGCTCTTGATCGAAGCCCCGGTAAACGGCGGCCGTAACTATAACGGTCC 1933

NZ_NOLM01000022.1:91220-95566(-) TTACTAAAAACACAGCACTCTGCAAACTCGTAAGAGGACGTATAGGGTGTGACGCCTGCCCGGTGCCGGAAGGTTAAGTGATGGGGTTAGCTCTCGGGCGAAGCTCTTGATCGAAGCCCCGGTAAACGGCGGCCGTAACTATAACGGTCC 1933

JBJCIS010000056.1:0-2780(-) TTACTAAAAACACAGCACTCTGCAAACTCGTAAGAGGACGTATAGGGTGTGACGCCTGCCCGGTGCCGGAAGGTTAAGTGATGGGGTTAGCCCTCGGGCGAAGCTCTTGATCGAAGCCCCGGTAAACGGCGGCCGTAACTATAACGGTCC 1933

NZ_CDBG01000001.1:179744-184090(+) TTACTAAAAACACAGCACTCTGCAAACTCGTAAGAGGACGTATAGGGTGTGACGCCTGCCCGGTGCCGGAAGGTTAAGTGATGGGGTTAGCCCTCGGGCGAAGCTCTTGATCGAAGCCCCGGTAAACGGCGGCCGTAACTATAACGGTCC 1933

NZ_CP103434.1:167622-171961(+) TTACTAAAAACACAGCACTCTGCAAACTCGTAAGAGGACGTATAGGGTGTGACGCCTGCCCGGTGCCGGAAGGTTAAGTGATGGGGTTAGCCCTCGGGCGAAGCTCTTGATCGAAGCCCCGGTAAACGGCGGCCGTAACTATAACGGTCC 1933

CP014354.1:76692-80603(-) TTACTAAAAACACAGCACTCTGCAAACTCGTAAGAGGACGTATAGGGTGTGACGCCTGCCCGGTGCCGGAAGGTTAAGTGATGGGGTTAGCCCTCGGGCGAAGCTCTTGATCGAAGCCCCGGTAAACGGCGGCCGTAACTATAACGGTCC 1464

NOLR01000002.1:131833-136207(+) TTACTAAAAACACAGCACTCTGCAAACTCGTAAGAGGACGTATAGGGTGTGACGCCTGCCCGGTGCCGGAAGGTTAAGTGATGGGGTTAGCCCTCGGGCGAAGCTCTTGATCGAAGCCCCGGTAAACGGCGGCCGTAACTATAACGGTCC 1943

PPFR01000008.1:34360-38734(-) TTACTAAAAACACAGCACTCTGCAAACTCGTAAGAGGACGTATAGGGTGTGACGCCTGCCCGGTGCCGGAAGGTTAAGTGATGGGGTTAGCCCTCGGGCGAAGCTCTTGATCGAAGCCCCGGTAAACGGCGGCCGTAACTATAACGGTCC 1943

PPFQ01000003.1:131083-135464(+) TTACTAAAAACACAGCACTCTGCAAACTCGTAAGAGGACGTATAGGGTGTGACGCCTGCCCGGTGCCGGAAGGTTAAGTGATGGGGTTAGCCCTCGGGCGAAGCTCTTGATCGAAGCCCCGGTAAACGGCGGCCGTAACTATAACGGTCC 1943

NZ_AKYP01000181.1:1647-6020(-) TTACTAAAAACACAGCACTCTGCAAACTCGTAAGAGGACGTATAGGGTGTGACGCCTGCCCGGTGCCGGAAGGTTAAGTGATGGGGTTAGCCCTCGGGCGAAGCTCTTGATCGAAGCCCCGGTAAACGGCGGCCGTAACTATAACGGTCC 1942

NOVI01000003.1:105955-110328(+) TTACTAAAAACACAGCACTCTGCAAACTCGTAAGAGGACGTATAGGGTGTGACGCCTGCCCGGTGCCGGAAGGTTAAGTGATGGGGTTAGCCCTCGGGCGAAGCTCTTGATCGAAGCCCCGGTAAACGGCGGCCGTAACTATAACGGTCC 1943

CP018150.1:339294-343661(+) TTACTAAAAACACAGCACTCTGCAAACTCGTAAGAGGACGTATAGGGTGTGACGCCTGCCCGGTGCCGGAAGGTTAAGTGATGGGGTTAGCCCTCGGGCGAAGCTCTTGATCGAAGCCCCGGTAAACGGCGGCCGTAACTATAACGGTCC 1943

Consensus TAAGGTAGCGAAATTCCTTGTCGGGTAAGTTCCGACCTGCACGAATGGCGTAACGATAGCCACGCTGTCTCCACCCAAGACTCAGTGAAATTGAAATCGCTGTGAAGATGCAGCGTACCCGCGGCTAGACGGAAAGACCCCGTGAACCTT 2093

JAKFBC010000002.1:34496-38842(-) (45) TAAGGTAGCGAAATTCCTTGTCGGGTAAGTTCCGACCTGCACGAATGGCGTAACGATAGCCACGCTGTCTCCACCCAAGACTCAGTGAAATTGAAATCGCTGTGAAGATGCAGCGTACCCGCGGCTAGACGGAAAGACCCCGTGAACCTT 2083

NZ_AP019757.1:1739264-1743610(-) (31) TAAGGTAGCGAAATTCCTTGTCGGGTAAGTTCCGACCTGCACGAATGGCGTAACGATAGCCACGCTGTCTCCACCCAAGACTCAGTGAAATTGAAATCGCTGTGAAGATGCAGCGTACCCGCGGCTAGACGGAAAGACCCCGTGAACCTT 2083

JBKOHN010000001.1:81530-85875(+) (25) TAAGGTAGCGAAATTCCTTGTCGGGTAAGTTCCGACCTGCACGAATGGCGTAACGATAGCCACGCTGTCTCCACCCAAGACTCAGTGAAATTGAAATCGCTGTGAAGATGCAGCGTACCCGCGGCTAGACGGAAAGACCCCGTGAACCTT 2083

NC_011528.1:339307-343646(+) (14) TAAGGTAGCGAAATTCCTTGTCGGGTAAGTTCCGACCTGCACGAATGGCGTAACGATAGCCACGCTGTCTCCACCCAAGACTCAGTGAAATTGAAATCGCTGTGAAGATGCAGCGTACCCGCGGCTAGACGGAAAGACCCCGTGAACCTT 2083

CP018005.1:167566-171940(+) (14) TAAGGTAGCGAAATTCCTTGTCGGGTAAGTTCCGACCTGCACGAATGGCGTAACGATAGCCACGCTGTCTCCACCCAAGACTCAGTGAAATTGAAATCGCTGTGAAGATGCAGCGTACCCGCGGCTAGACGGAAAGACCCCGTGAACCTT 2093

NZ_CCAM010000005.1:71418-75764(+) (13) TAAGGTAGCGAAATTCCTTGTCGGGTAAGTTCCGACCTGCACGAATGGCGTAACGATAGCCACGCTGTCTCCACCCAAGACTCAGTGAAATTGAAATCGCTGTGAAGATGCAGCGTACCCGCGGCTAGACGGAAAGACCCCGTGAACCTT 2083

NZ_CCXO01000001.1:317226-321572(+) (11) TAAGGTAGCGAAATTCCTTGTCGGGTAAGTTCCGACCTGCACGAATGGCGTAACGATAGCCACGCTGTCTCCACCCAAGACTCAGTGAAATTGAAATCGCTGTGAAGATGCAGCGTACCCGCGGCTAGACGGAAAGACCCCGTGAACCTT 2083

NC_011527.1:1751498-1755844(-) (8) TAAGGTAGCGAAATTCCTTGTCGGGTAAGTTCCGACCTGCACGAATGGCGTAACGATAGCCACGCTGTCTCCACCCAAGACTCAGTGAAATTGAAATCGCTGTGAAGATGCAGCGTACCCGCGGCTAGACGGAAAGACCCCGTGAACCTT 2083

NZ_CP032542.1:88036-92382(-) (8) TAAGGTAGCGAAATTCCTTGTCGGGTAAGTTCCGACCTGCACGAATGGCGTAACGATAGCCACGCTGTCTCCACCCAAGACTCAGTGAAATTGAAATCGCTGTGAAGATGCAGCGTACCCGCGGCTAGACGGAAAGACCCCGTGAACCTT 2083

CP014551.1:161236-165610(+) (8) TAAGGTAGCGAAATTCCTTGTCGGGTAAGTTCCGACCTGCACGAATGGCGTAACGATAGCCACGCTGTCTCCACCCAAGACTCAGTGAAATTGAAATCGCTGTGAAGATGCAGCGTACCCGCGGCTAGACGGAAAGACCCCGTGAACCTT 2093

CP013667.1:88011-92385(-) (8) TAAGGTAGCGAAATTCCTTGTCGGGTAAGTTCCGACCTGCACGAATGGCGTAACGATAGCCACGCTGTCTCCACCCAAGACTCAGTGAAATTGAAATCGCTGTGAAGATGCAGCGTACCCGCGGCTAGACGGAAAGACCCCGTGAACCTT 2093

CP107247.1:164350-168689(+) (7) TAAGGTAGCGAAATTCCTTGTCGGGTAAGTTCCGACCTGCACGAATGGCGTAACGATAGCCACGCTGTCTCCACCCAAGACTCAGTGAAATTGAAATCGCTGTGAAGATGCAGCGTACCCGCGGCTAGACGGAAAGACCCCGTGAACCTT 2083

NZ_CP103435.1:167581-171920(+) (6) TAAGGTAGCGAAATTCCTTGTCGGGTAAGTTCCGACCTGCACGAATGGCGTAACGATAGCCACGCTGTCTCCACCCAAGACTCAGTGAAATTGAAATCGCTGTGAAGATGCAGCGTACCCGCGGCTAGACGGAAAGACCCCGTGAACCTT 2083

CP014565.1:1711747-1716121(-) (3) TAAGGTAGCGAAATTCCTTGTCGGGTAAGTTCCGACCTGCACGAATGGCGTAACGATAGCCACGCTGTCTCCACCCAAGACTCAGTGAAATTGAAATCGCTGTGAAGATGCAGCGTACCCGCGGCTAGACGGAAAGACCCCGTGAACCTT 2093

AAYJ01000007.1:0-1404(+) (3) ----------------------------GTTCCGACCTGCACGAATGGCGTAACGATAGCCACGCTGTCTCCACCCAAGACTCAGTGAAATTGAAATCGCTGTGAAGATGCAGCGTACCCGCGGCTAGACGGAAAGACCCCGTGAACCTT 122

NZ_JAOXDR010000002.1:16672-21025(-) (3) TAAGGTAGCGAAATTCCTTGTCGGGTAAGTTCCGACCTGCACGAATGGCGTAACGATAGCCACGCTGTCTCCACCCAAGACTCAGTGAAATTGAAATCGCTGTGAAGATGCAGCGTACCCGCGGCTAGACGGAAAGACCCCGTGAACCTT 2083

NZ_CP103432.1:167699-172038(+) (3) TAAGGTAGCGAAATTCCTTGTCGGGTAAGTTCCGACCTGCACGAATGGCGTAACGATAGCCACGCTGTCTCCACCCAAGACTCAGTGAAATTGAAATCGCTGTGAAGATGCAGCGTACCCGCGGCTAGACGGAAAGACCCCGTGAACCTT 2083

NZ_CP103431.1:167697-172036(+) (3) TAAGGTAGCGAAATTCCTTGTCGGGTAAGTTCCGACCTGCACGAATGGCGTAACGATAGCCACGCTGTCTCCACCCAAGACTCAGTGAAATTGAAATCGCTGTGAAGATGCAGCGTACCCGCGGCTAGACGGAAAGACCCCGTGAACCTT 2083

NZ_CP103428.1:167591-171929(+) (2) TAAGGTAGCGAAATTCCTTGTCGGGTAAGTTCCGACCTGCACGAATGGCGTAACGATAGCCACGCTGTCTCCACCCAAGACTCAGTGAAATTGAAATCGCTGTGAAGATGCAGCGTACCCGCGGCTAGACGGAAAGACCCCGTGAACCTT 2083

NZ_JANTNR010000002.1:115350-119696(+) (2) TAAGGTAGCGAAATTCCTTGTCGGGTAAGTTCCGACCTGCACGAATGGCGTAACGATAGCCACGCTGTCTCCACCCAAGACTCAGTGAAATTGAAATCGCTGTGAAGATGCAGCGTACCCGCGGCTAGACGGAAAGACCCCGTGAACCTT 2083

JARBIR010004564.1:3782-8128(-) (2) TAAGGTAGCGAAATTCCTTGTCGGGTAAGTTCCGACCTGCACGAATGGCGTAACGATAGCCACGCTGTCTCCACCCAAGACTCAGTGAAATTGAAATCGCTGTGAAGATGCAGCGTACCCGCGGCTAGACGGAAAGACCCCGTGAACCTT 2083

NC_009727.1:1888349-1892695(-) (2) TAAGGTAGCGAAATTCCTTGTCGGGTAAGTTCCGACCTGCACGAATGGCGTAACGATAGCCACGCTGTCTCCACCCAAGACTCAGTGAAATTGAAATCGCTGTGAAGATGCAGCGTACCCGCGGCTAGACGGAAAGACCCCGTGAACCTT 2083

AAYJ01000139.1:0-660(+) (2) ------------------------------------------------------------------------------------------------------------------------------------------------------ 909

CP032542.1:88018-92392(-) (2) TAAGGTAGCGAAATTCCTTGTCGGGTAAGTTCCGACCTGCACGAATGGCGTAACGATAGCCACGCTGTCTCCACCCAAGACTCAGTGAAATTGAAATCGCTGTGAAGATGCAGCGTACCCGCGGCTAGACGGAAAGACCCCGTGAACCTT 2093

CP014559.1:232744-237117(+) (2) TAAGGTAGCGAAATTCCTTGTCGGGTAAGTTCCGACCTGCACGAATGGCGTAACGATAGCCACGCTGTCTCCACCCAAGACTCAGTGAAATTGAAATCGCTGTGAAGATGCAGCGTACCCGCGGCTAGACGGAAAGACCCCGTGAACCTT 2093

CP007555.1:337408-341752(+) TAAGGTAGCGAAATTCCTTGTCGGGTAAGTTCCGACCTGCACGAATGGCGTAACGATAGCCACGCTGTCTCCACCCAAGACTCAGTGAAATTGAAATCGCTGTGAAGATGCAGCGTACCCGCGGCTAGACGGAAAGACCCCGTGAACCTT 2081

NZ_CP014354.1:76710-81178(-) TAAGGTAGCGAAATTCCTTGTCGGGTAAGTTCCGACCTGCACGAATGGCGTAACGATAGCCACGCTGTCTCCACCCAAGACTCAGTGAAATTGAAATCGCTGTGAAGATGCAGCGTACCCGCGGCTAGACGGAAAGACCCCGTGAACCTT 2089

CP000890.1:243129-244433(+) TAAGGTAGCGAAATTCCTTGTCGGGTAA-------------------------------------------------------------------------------------------------------------------------- 909

NZ_CP115461.1:167576-171922(+) TAAGGTAGCGAAATTCCTTGTCGGGTAAGTTCCGACCTGCACGAATGGCGTAACGATAGCCACGCTGTCTCCACCCAAGACTCAGTGAAATTGAAATCGCTGTGAAGATGCAGCGTACCCGCGGCTAGACGGAAAGACCCCGTGAACCTT 2083

AP019759.1:167578-171919(+) TAAGGTAGCGAAATTCCTTGTCGGGTAAGTTCCGACCTGCACGAATGGCGTAACGATAGCCACGCTGTCTCCACCCAAGACTCAGTGAAATTGAAATCGCTGTGAAGATGCAGCGTACCCGCGGCTAGACGGAAAGACCCCGTGAACCTT 2081

AP019757.1:1739267-1743608(-) TAAGGTAGCGAAATTCCTTGTCGGGTAAGTTCCGACCTGCACGAATGGCGTAACGATAGCCACGCTGTCTCCACCCAAGACTCAGTGAAATTGAAATCGCTGTGAAGATGCAGCGTACCCGCGGCTAGACGGAAAGACCCCGTGAACCTT 2081

NOLN01000021.1:90861-95235(-) TAAGGTAGCGAAATTCCTTGTCGGGTAAGTTCCGACCTGCACGAATGGCGTAACGATAGCCACGCTGTCTCCACCCAAGACTCAGTGAAATTGAAATCGCTGTGAAGATGCAGCGTACCCGCGGCTAGACGGAAAGACCCCGTGAACCTT 2093

NOLM01000022.1:91202-95576(-) TAAGGTAGCGAAATTCCTTGTCGGGTAAGTTCCGACCTGCACGAATGGCGTAACGATAGCCACGCTGTCTCCACCCAAGACTCAGTGAAATTGAAATCGCTGTGAAGATGCAGCGTACCCGCGGCTAGACGGAAAGACCCCGTGAACCTT 2093

PDLP01000011.1:14431-18805(+) TAAGGTAGCGAAATTCCTTGTCGGGTAAGTTCCGACCTGCACGAATGGCGTAACGATAGCCACGCTGTCTCCACCCAAGACTCAGTGAAATTGAAATCGCTGTGAAGATGCAGCGTACCCGCGGCTAGACGGAAAGACCCCGTGAACCTT 2093

CP014563.1:522940-527314(+) TAAGGTAGCGAAATTCCTTGTCGGGTAAGTTCCGACCTGCACGAATGGCGTAACGATAGCCACGCTGTCTCCACCCAAGACTCAGTGAAATTGAAATCGCTGTGAAGATGCAGCGTACCCGCGGCTAGACGGAAAGACCCCGTGAACCTT 2093

CP000890.1:244922-245905(+) ----------------------------GTTCCGACCTGCACGAATGGCGTAACGATAGCCACGCTGTCTCCACCCAAGACTCAGTGAAATTGAAATCGCTGTGAAGATGCAGCGTACCCGCGGCTAGACGGAAAGACCCCGTGAACCTT 122

NZ_NOLR01000002.1:131843-136189(+) TAAGGTAGCGAAATTCCTTGTCGGGTAAGTTCCGACCTGCACGAATGGCGTAACGATAGCCACGCTGTCTCCACCCAAGACTCAGTGAAATTGAAATCGCTGTGAAGATGCAGCGTACCCGCGGCTAGACGGAAAGACCCCGTGAACCTT 2083

NZ_JAOXDP010000002.1:15794-20139(-) TAAGGTAGCGAAATTCCTTGTCGGGTAAGTTCCGACCTGCACGAATGGCGTAACGATAGCCACGCTGTCTCCACCCAAGACTCAGTGAAATTGAAATCGCTGTGAAGATGCAGCGTACCCGCGGCTAGACGGAAAGACCCCGTGAACCTT 2083

NZ_PPFR01000008.1:34378-38724(-) TAAGGTAGCGAAATTCCTTGTCGGGTAAGTTCCGACCTGCACGAATGGCGTAACGATAGCCACGCTGTCTCCACCCAAGACTCAGTGAAATTGAAATCGCTGTGAAGATGCAGCGTACCCGCGGCTAGACGGAAAGACCCCGTGAACCTT 2083

NZ_PPFQ01000003.1:131093-135446(+) TAAGGTAGCGAAATTCCTTGTCGGGTAAGTTCCGACCTGCACGAATGGCGTAACGATAGCCACGCTGTCTCCACCCAAGACTCAGTGAAATTGAAATCGCTGTGAAGATGCAGCGTACCCGCGGCTAGACGGAAAGACCCCGTGAACCTT 2083

NZ_LK937696.1:167613-171953(+) TAAGGTAGCGAAATTCCTTGTCGGGTAAGTTCCGACCTGCACGAATGGCGTAACGATAGCCACGCTGTCTCCACCCAAGACTCAGTGAAATTGAAATCGCTGTGAAGATGCAGCGTACCCGCGGCTAGACGGAAAGACCCCGTGAACCTT 2083

NZ_NOVI01000003.1:105965-110310(+) TAAGGTAGCGAAATTCCTTGTCGGGTAAGTTCCGACCTGCACGAATGGCGTAACGATAGCCACGCTGTCTCCACCCAAGACTCAGTGAAATTGAAATCGCTGTGAAGATGCAGCGTACCCGCGGCTAGACGGAAAGACCCCGTGAACCTT 2083

NZ_CP103426.1:167642-171981(+) TAAGGTAGCGAAATTCCTTGTCGGGTAAGTTCCGACCTGCACGAATGGCGTAACGATAGCCACGCTGTCTCCACCCAAGACTCAGTGAAATTGAAATCGCTGTGAAGATGCAGCGTACCCGCGGCTAGACGGAAAGACCCCGTGAACCTT 2083

NZ_JAOXDN010000007.1:36985-41324(-) TAAGGTAGCGAAATTCCTTGTCGGGTAAGTTCCGACCTGCACGAATGGCGTAACGATAGCCACGCTGTCTCCACCCAAGACTCAGTGAAATTGAAATCGCTGTGAAGATGCAGCGTACCCGCGGCTAGACGGAAAGACCCCGTGAACCTT 2083

NZ_JPVV01000013.1:13888-18051(+) TAAGGTAGCGAAATTCCTTGTCGGGTAAGTTCCGACCTGCACGAATGGCGTAACGATAGCCACGCTGTCTCCACCCAAGACTCAGTGAAATTGAAATCGCTGTGAAGATGCAGCGTACCCGCGGCTAGACGGAAAGACCCCGTGAACCTT 2083

NZ_CP007555.1:337406-341752(+) TAAGGTAGCGAAATTCCTTGTCGGGTAAGTTCCGACCTGCACGAATGGCGTAACGATAGCCACGCTGTCTCCACCCAAGACTCAGTGAAATTGAAATCGCTGTGAAGATGCAGCGTACCCGCGGCTAGACGGAAAGACCCCGTGAACCTT 2083

NZ_JASNNV010000010.1:39284-43631(-) TAAGGTAGCGAAATTCCTTGTCGGGTAAGTTCCGACCTGCACGAATGGCGTAACGATAGCCACGCTGTCTCCACCCAAGACTCAGTGAAATTGAAATCGCTGTGAAGATGCAGCGTACCCGCGGCTAGACGGAAAGACCCCGTGAACCTT 2083

NZ_JAOXFC010000012.1:14231-18577(+) TAAGGTAGCGAAATTCCTTGTCGGGTAAGTTCCGACCTGCACGAATGGCGTAACGATAGCCACGCTGTCTCCACCCAAGACTCAGTGAAATTGAAATCGCTGTGAAGATGCAGCGTACCCGCGGCTAGACGGAAAGACCCCGTGAACCTT 2083

NZ_CP103430.1:167658-171997(+) TAAGGTAGCGAAATTCCTTGTCGGGTAAGTTCCGACCTGCACGAATGGCGTAACGATAGCCACGCTGTCTCCACCCAAGACTCAGTGAAATTGAAATCGCTGTGAAGATGCAGCGTACCCGCGGCTAGACGGAAAGACCCCGTGAACCTT 2083

NZ_PDLP01000011.1:14441-18787(+) TAAGGTAGCGAAATTCCTTGTCGGGTAAGTTCCGACCTGCACGAATGGCGTAACGATAGCCACGCTGTCTCCACCCAAGACTCAGTGAAATTGAAATCGCTGTGAAGATGCAGCGTACCCGCGGCTAGACGGAAAGACCCCGTGAACCTT 2083

NZ_NOLM01000022.1:91220-95566(-) TAAGGTAGCGAAATTCCTTGTCGGGTAAGTTCCGACCTGCACGAATGGCGTAACGATAGCCACGCTGTCTCCACCCAAGACTCAGTGAAATTGAAATCGCTGTGAAGATGCAGCGTACCCGCGGCTAGACGGAAAGACCCCGTGAACCTT 2083

JBJCIS010000056.1:0-2780(-) TAAGGTAGCGAAATTCCTTGTCGGGTAA-------------------------------------------------------------------------------------------------------------------------- 2083

NZ_CDBG01000001.1:179744-184090(+) TAAGGTAGCGAAATTCCTTGTCGGGTAAGTTCCGACCTGCACGAATGGCGTAACGATAGCCACGCTGTCTCCACCCAAGACTCAGTGAAATTGAAATCGCTGTGAAGATGCAGCGTACCCGCGGCTAGACGGAAAGACCCCGTGAACCTT 2083

NZ_CP103434.1:167622-171961(+) TAAGGTAGCGAAATTCCTTGTCGGGTAAGTTCCGACCTGCACGAATGGCGTAACGATAGCCACGCTGTCTCCACCCAAGACTCAGTGAAATTGAAATCGCTGTGAAGATGCAGCGTACCCGCGGCTAGACGGAAAGACCCCGTGAACCTT 2083

CP014354.1:76692-80603(-) TAAGGTAGCGAAATTCCTTGTCGGGTAAGTTCCGACCTGCACGAATGGCGTAACGATAGCCACGCTGTCTCCACCCAAGACTCAGTGAAATTGAAATCGCTGTGAAGATGCAGCGTACCCGCGGCTAGACGGAAAGACCCCGTGAACCTT 1614

NOLR01000002.1:131833-136207(+) TAAGGTAGCGAAATTCCTTGTCGGGTAAGTTCCGACCTGCACGAATGGCGTAACGATAGCCACGCTGTCTCCACCCAAGACTCAGTGAAATTGAAATCGCTGTGAAGATGCAGCGTACCCGCGGCTAGACGGAAAGACCCCGTGAACCTT 2093

PPFR01000008.1:34360-38734(-) TAAGGTAGCGAAATTCCTTGTCGGGTAAGTTCCGACCTGCACGAATGGCGTAACGATAGCCACGCTGTCTCCACCCAAGACTCAGTGAAATTGAAATCGCTGTGAAGATGCAGCGTACCCGCGGCTAGACGGAAAGACCCCGTGAACCTT 2093

PPFQ01000003.1:131083-135464(+) TAAGGTAGCGAAATTCCTTGTCGGGTAAGTTCCGACCTGCACGAATGGCGTAACGATAGCCACGCTGTCTCCACCCAAGACTCAGTGAAATTGAAATCGCTGTGAAGATGCAGCGTACCCGCGGCTAGACGGAAAGACCCCGTGAACCTT 2093

NZ_AKYP01000181.1:1647-6020(-) TAAGGTAGCGAAATTCCTTGTCGGGTAAGTTCCGACCTGCACGAATGGCGTAACGATAGCCACGCTGTCTCCACCCAAGACTCAGTGAAATTGAAATCGCTGTGAAGATGCAGCGTACCCGCGGCTAGACGGAAAGACCCCGTGAACCTT 2092

NOVI01000003.1:105955-110328(+) TAAGGTAGCGAAATTCCTTGTCGGGTAAGTTCCGACCTGCACGAATGGCGTAACGATAGCCACGCTGTCTCCACCCAAGACTCAGTGAAATTGAAATCGCTGTGAAGATGCAGCGTACCCGCGGCTAGACGGAAAGACCCCGTGAACCTT 2093

CP018150.1:339294-343661(+) TAAGGTAGCGAAATTCCTTGTCGGGTAAGTTCCGACCTGCACGAATGGCGTAACGATAGCCACGCTGTCTCCACCCAAGACTCAGTGAAATTGAAATCGCTGTGAAGATGCAGCGTACCCGCGGCTAGACGGAAAGACCCCGTGAACCTT 2093

Consensus TACTACAGCTTTACACTGAACTTTGAATATGCCTGTGTAGGATAGGTGGGAGGCTTTGAAGCTGGAGCGCTAGCTCCAGTGGAGCCAACCTTGAAATACCACCCTGGCGTGTTTGAGGTTCTAACCCTGTCCCGTAATCCGGGTCGGGAA 2243

JAKFBC010000002.1:34496-38842(-) (45) TACTACAGCTTTACACTGAACTTTGAATATGCCTGTGTAGGATAGGTGGGAGGCTTTGAAGCTGGAGCGCTAGCTCCAGTGGAGCCAACCTTGAAATACCACCCTGGCGTGTTTGAGGTTCTAACCCTGTCCCGTAATCCGGGTCGGGAA 2233

NZ_AP019757.1:1739264-1743610(-) (31) TACTACAGCTTTACACTGAACTTTGAATATGCCTGTGTAGGATAGGTGGGAGGCTTTGAAGCTGGAGCGCTAGCTCCAGTGGAGCCAACCTTGAAATACCACCCTGGCGTGTTTGAGGTTCTAACCCTGTCCCGTAATCCGGGTCGGGAA 2233

JBKOHN010000001.1:81530-85875(+) (25) TACTACAGCTTTACACTGAACTTTGAATATGCCTGTGTAGGATAGGTGGGAGGCTTTGAAGCTGGAGCGCTAGCTCCAGTGGAGCCAACCTTGAAATACCACCCTGGCGTGTTTGAGGTTCTAACCCTGTCCCGTAATCCGGGTCGGGAA 2233

NC_011528.1:339307-343646(+) (14) TACTACAGCTTTACACTGAACTTTGAATATGCCTGTGTAGGATAGGTGGGAGGCTTTGAAGCTGGAGCGCTAGCTCCAGTGGAGCCAACCTTGAAATACCACCCTGGCGTGTTTGAGGTTCTAACCCTGTCCCGTAATCCGGGTCGGGAA 2233

CP018005.1:167566-171940(+) (14) TACTACAGCTTTACACTGAACTTTGAATATGCCTGTGTAGGATAGGTGGGAGGCTTTGAAGCTGGAGCGCTAGCTCCAGTGGAGCCAACCTTGAAATACCACCCTGGCGTGTTTGAGGTTCTAACCCTGTCCCGTAATCCGGGTCGGGAA 2243

NZ_CCAM010000005.1:71418-75764(+) (13) TACTACAGCTTTACACTGAACTTTGAATATGCCTGTGTAGGATAGGTGGGAGGCTTTGAAGCTGGAGCGCTAGCTCCAGTGGAGCCAACCTTGAAATACCACCCTGGCGTGTTTGAGGTTCTAACCCTGTCCCGTAATCCGGGTCGGGAA 2233

NZ_CCXO01000001.1:317226-321572(+) (11) TACTACAGCTTTACACTGAACTTTGAATATGCCTGTGTAGGATAGGTGGGAGGCTTTGAAGCTGGAGCGCTAGCTCCAGTGGAGCCAACCTTGAAATACCACCCTGGCGTGTTTGAGGTTCTAACCCTGTCCCGTAATCCGGGTCGGGAA 2233

NC_011527.1:1751498-1755844(-) (8) TACTACAGCTTTACACTGAACTTTGAATATGCCTGTGTAGGATAGGTGGGAGGCTTTGAAGCTGGAGCGCTAGCTCCAGTGGAGCCAACCTTGAAATACCACCCTGGCGTGTTTGAGGTTCTAACCCTGTCCCGTAATCCGGGTCGGGAA 2233

NZ_CP032542.1:88036-92382(-) (8) TACTACAGCTTTACACTGAACTTTGAATATGCCTGTGTAGGATAGGTGGGAGGCTTTGAAGCTGGAGCGCTAGCTCCAGTGGAGCCAACCTTGAAATACCACCCTGGCGTGTTTGAGGTTCTAACCCTGTCCCGTAATCCGGGTCGGGAA 2233

CP014551.1:161236-165610(+) (8) TACTACAGCTTTACACTGAACTTTGAATATGCCTGTGTAGGATAGGTGGGAGGCTTTGAAGCTGGAGCGCTAGCTCCAGTGGAGCCAACCTTGAAATACCACCCTGGCGTGTTTGAGGTTCTAACCCTGTCCCGTAATCCGGGTCGGGAA 2243

CP013667.1:88011-92385(-) (8) TACTACAGCTTTACACTGAACTTTGAATATGCCTGTGTAGGATAGGTGGGAGGCTTTGAAGCTGGAGCGCTAGCTCCAGTGGAGCCAACCTTGAAATACCACCCTGGCGTGTTTGAGGTTCTAACCCTGTCCCGTAATCCGGGTCGGGAA 2243

CP107247.1:164350-168689(+) (7) TACTACAGCTTTACACTGAACTTTGAATATGCCTGTGTAGGATAGGTGGGAGGCTTTGAAGCTGGAGCGCTAGCTCCAGTGGAGCCAACCTTGAAATACCACCCTGGCGTGTTTGAGGTTCTAACCCTGTCCCGTAATCCGGGTCGGGAA 2233

NZ_CP103435.1:167581-171920(+) (6) TACTACAGCTTTACACTGAACTTTGAATATGCCTGTGTAGGATAGGTGGGAGGCTTTGAAGCTGGAGCGCTAGCTCCAGTGGAGCCAACCTTGAAATACCACCCTGGCGTGTTTGAGGTTCTAACCCTGTCCCGTAATCCGGGTCGGGAA 2233

CP014565.1:1711747-1716121(-) (3) TACTACAGCTTTACACTGAACTTTGAATATGCCTGTGTAGGATAGGTGGGAGGCTTTGAAGCTGGAGCGCTAGCTCCAGTGGAGCCAACCTTGAAATACCACCCTGGCGTGTTTGAGGTTCTAACCCTGTCCCGTAATCCGGGTCGGGAA 2243

AAYJ01000007.1:0-1404(+) (3) TACTACAGCTTTACACTGAACTTTGAATATGCCTGTGTAGGATAGGTGGGAGGCTTTGAAGCTGGAGCGCTAGCTCCAGTGGAGCCAACCTTGAAATACCACCCTGGCGTGTTTGAGGTTCTAACCCTGTCCCGTAATCCGGGTCGGGAA 272

NZ_JAOXDR010000002.1:16672-21025(-) (3) TACTACAGCTTTACACTGAACTTTGAATATGCCTGTGTAGGATAGGTGGGAGGCTTTGAAGCTGGAGCGCTAGCTCCAGTGGAGCCAACCTTGAAATACCACCCTGGCGTGTTTGAGGTTCTAACCCTGTCCCGTAATCCGGGTCGGGAA 2233

NZ_CP103432.1:167699-172038(+) (3) TACTACAGCTTTACACTGAACTTTGAATATGCCTGTGTAGGATAGGTGGGAGGCTTTGAAGCTGGAGCGCTAGCTCCAGTGGAGCCAACCTTGAAATACCACCCTGGCGTGTTTGAGGTTCTAACCCTGTCCCGTAATCCGGGTCGGGAA 2233

NZ_CP103431.1:167697-172036(+) (3) TACTACAGCTTTACACTGAACTTTGAATATGCCTGTGTAGGATAGGTGGGAGGCTTTGAAGCTGGAGCGCTAGCTCCAGTGGAGCCAACCTTGAAATACCACCCTGGCGTGTTTGAGGTTCTAACCCTGTCCCGTAATCCGGGTCGGGAA 2233

NZ_CP103428.1:167591-171929(+) (2) TACTACAGCTTTACACTGAACTTTGAATATGCCTGTGTAGGATAGGTGGGAGGCTTTGAAGCTGGAGCGCTAGCTCCAGTGGAGCCAACCTTGAAATACCACCCTGGCGTGTTTGAGGTTCTAACCCTGTCCCGTAATCCGGGTCGGGAA 2233

NZ_JANTNR010000002.1:115350-119696(+) (2) TACTACAGCTTTACACTGAACTTTGAATATGCCTGTGTAGGATAGGTGGGAGGCTTTGAAGCTGGAGCGCTAGCTCCAGTGGAGCCAACCTTGAAATACCACCCTGGCGTGTTTGAGGTTCTAACCCTGTCCCGTAATCCGGGTCGGGAA 2233

JARBIR010004564.1:3782-8128(-) (2) TACTACAGCTTTACACTGAACTTTGAATATGCCTGTGTAGGATAGGTGGGAGGCTTTGAAGCTGGAGCGCTAGCTCCAGTGGAGCCAACCTTGAAATACCACCCTGGCGTGTTTGAGGTTCTAACCCTGTCCCGTAATCCGGGTCGGGAA 2233

NC_009727.1:1888349-1892695(-) (2) TACTACAGCTTTACACTGAACTTTGAATATGCCTGTGTAGGATAGGTGGGAGGCTTTGAAGCTGGAGCGCTAGCTCCAGTGGAGCCAACCTTGAAATACCACCCTGGCGTGTTTGAGGTTCTAACCCTGTCCCGTAATCCGGGTCGGGAA 2233

AAYJ01000139.1:0-660(+) (2) ------------------------------------------------------------------------------------------------------------------------------------------------------ 1059

CP032542.1:88018-92392(-) (2) TACTACAGCTTTACACTGAACTTTGAATATGCCTGTGTAGGATAGGTGGGAGGCTTTGAAGCTGGAGCGCTAGCTCCAGTGGAGCCAACCTTGAAATACCACCCTGGCGTGTTTGAGGTTCTAACCCTGTCCCGTAATCCGGGTCGGGAA 2243

CP014559.1:232744-237117(+) (2) TACTACAGCTTTACACTGAACTTTGAATATGCCTGTGTAGGATAGGTGGGAGGCTTTGAAGCTGGAGCGCTAGCTCCAGTGGAGCCAACCTTGAAATACCACCCTGGCGTGTTTGAGGTTCTAACCCTGTCCCGTAATCCGGGTCGGGAA 2243

CP007555.1:337408-341752(+) TACTACAGCTTTACACTGAACTTTGAATATGCCTGTGTAGGATAGGTGGGAGGCTTTGAAGCTGGAGCGCTAGCTCCAGTGGAGCCAACCTTGAAATACCACCCTGGCGTGTTTGAGGTTCTAACCCTGTCCCGTAATCCGGGTCGGGAA 2231

NZ_CP014354.1:76710-81178(-) TACTACAGCTTTACACTGAACTTTGAATATGCCTGTGTAGGATAGGTGGGAGGCTTTGAAGCTGGAGCGCTAGCTCCAGTGGAGCCAACCTTGAAATACCACCCTGGCGTGTTTGAGGTTCTAACCCTGTCCCGTAATCCGGGTCGGGAA 2239

CP000890.1:243129-244433(+) ------------------------------------------------------------------------------------------------------------------------------------------------------ 1059

NZ_CP115461.1:167576-171922(+) TACTACAGCTTTACACTGAACTTTGAATATGCCTGTGTAGGATAGGTGGGAGGCTTTGAAGCTGGAGCGCTAGCTCCAGTGGAGCCAACCTTGAAATACCACCCTGGCGTGTTTGAGGTTCTAACCCTGTCCCGTAATCCGGGTCGGGAA 2233

AP019759.1:167578-171919(+) TACTACAGCTTTACACTGAACTTTGAATATGCCTGTGTAGGATAGGTGGGAGGCTTTGAAGCTGGAGCGCTAGCTCCAGTGGAGCCAACCTTGAAATACCACCCTGGCGTGTTTGAGGTTCTAACCCTGTCCCGTAATCCGGGTCGGGAA 2231

AP019757.1:1739267-1743608(-) TACTACAGCTTTACACTGAACTTTGAATATGCCTGTGTAGGATAGGTGGGAGGCTTTGAAGCTGGAGCGCTAGCTCCAGTGGAGCCAACCTTGAAATACCACCCTGGCGTGTTTGAGGTTCTAACCCTGTCCCGTAATCCGGGTCGGGAA 2231

NOLN01000021.1:90861-95235(-) TACTACAGCTTTACACTGAACTTTGAATATGCCTGTGTAGGATAGGTGGGAGGCTTTGAAGCTGGAGCGCTAGCTCCAGTGGAGCCAACCTTGAAATACCACCCTGGCGTGTTTGAGGTTCTAACCCTGTCCCGTAATCCGGGTCGGGAA 2243

NOLM01000022.1:91202-95576(-) TACTACAGCTTTACACTGAACTTTGAATATGCCTGTGTAGGATAGGTGGGAGGCTTTGAAGCTGGAGCGCTAGCTCCAGTGGAGCCAACCTTGAAATACCACCCTGGCGTGTTTGAGGTTCTAACCCTGTCCCGTAATCCGGGTCGGGAA 2243

PDLP01000011.1:14431-18805(+) TACTACAGCTTTACACTGAACTTTGAATATGCCTGTGTAGGATAGGTGGGAGGCTTTGAAGCTGGAGCGCTAGCTCCAGTGGAGCCAACCTTGAAATACCACCCTGGCGTGTTTGAGGTTCTAACCCTGTCCCGTAATCCGGGTCGGGAA 2243

CP014563.1:522940-527314(+) TACTACAGCTTTACACTGAACTTTGAATATGCCTGTGTAGGATAGGTGGGAGGCTTTGAAGCTGGAGCGCTAGCTCCAGTGGAGCCAACCTTGAAATACCACCCTGGCGTGTTTGAGGTTCTAACCCTGTCCCGTAATCCGGGTCGGGAA 2243

CP000890.1:244922-245905(+) TACTACAGCTTTACACTGAACTTTGAATATGCCTGTGTAGGATAGGTGGGAGGCTTTGAAGCTGGAGCGCTAGCTCCAGTGGAGCCAACCTTGAAATACCACCCTGGCGTGTTTGAGGTTCTAACCCTGTCCCGTAATCCGGGTCGGGAA 272

NZ_NOLR01000002.1:131843-136189(+) TACTACAGCTTTACACTGAACTTTGAATATGCCTGTGTAGGATAGGTGGGAGGCTTTGAAGCTGGAGCGCTAGCTCCAGTGGAGCCAACCTTGAAATACCACCCTGGCGTGTTTGAGGTTCTAACCCTGTCCCGTAATCCGGGTCGGGAA 2233

NZ_JAOXDP010000002.1:15794-20139(-) TACTACAGCTTTACACTGAACTTTGAATATGCCTGTGTAGGATAGGTGGGAGGCTTTGAAGCTGGAGCGCTAGCTCCAGTGGAGCCAACCTTGAAATACCACCCTGGCGTGTTTGAGGTTCTAACCCTGTCCCGTAATCCGGGTCGGGAA 2233

NZ_PPFR01000008.1:34378-38724(-) TACTACAGCTTTACACTGAACTTTGAATATGCCTGTGTAGGATAGGTGGGAGGCTTTGAAGCTGGAGCGCTAGCTCCAGTGGAGCCAACCTTGAAATACCACCCTGGCGTGTTTGAGGTTCTAACCCTGTCCCGTAATCCGGGTCGGGAA 2233

NZ_PPFQ01000003.1:131093-135446(+) TACTACAGCTTTACACTGAACTTTGAATATGCCTGTGTAGGATAGGTGGGAGGCTTTGAAGCTGGAGCGCTAGCTCCAGTGGAGCCAACCTTGAAATACCACCCTGGCGTGTTTGAGGTTCTAACCCTGTCCCGTAATCCGGGTCGGGAA 2233

NZ_LK937696.1:167613-171953(+) TACTACAGCTTTACACTGAACTTTGAATATGCCTGTGTAGGATAGGTGGGAGGCTTTGAAGCTGGAGCGCTAGCTCCAGTGGAGCCAACCTTGAAATACCACCCTGGCGTGTTTGAGGTTCTAACCCTGTCCCGTAATCCGGGTCGGGAA 2233

NZ_NOVI01000003.1:105965-110310(+) TACTACAGCTTTACACTGAACTTTGAATATGCCTGTGTAGGATAGGTGGGAGGCTTTGAAGCTGGAGCGCTAGCTCCAGTGGAGCCAACCTTGAAATACCACCCTGGCGTGTTTGAGGTTCTAACCCTGTCCCGTAATCCGGGTCGGGAA 2233

NZ_CP103426.1:167642-171981(+) TACTACAGCTTTACACTGAACTTTGAATATGCCTGTGTAGGATAGGTGGGAGGCTTTGAAGCTGGAGCGCTAGCTCCAGTGGAGCCAACCTTGAAATACCACCCTGGCGTGTTTGAGGTTCTAACCCTGTCCCGTAATCCGGGTCGGGAA 2233

NZ_JAOXDN010000007.1:36985-41324(-) TACTACAGCTTTACACTGAACTTTGAATATGCCTGTGTAGGATAGGTGGGAGGCTTTGAAGCTGGAGCGCTAGCTCCAGTGGAGCCAACCTTGAAATACCACCCTGGCGTGTTTGAGGTTCTAACCCTGTCCCGTAATCCGGGTCGGGAA 2233

NZ_JPVV01000013.1:13888-18051(+) TACTACAGCTTTACACTGAACTTTGAATATGCCTGTGTAGGATAGGTGGGAGGCTTTGAAGCTGGAGCGCTAGCTCCAGTGGAGCCAACCTTGAAATACCACCCTGGCGTGTTTGAGGTTCTAACCCTGTCCCGTAATCCGGGTCGGGAA 2233

NZ_CP007555.1:337406-341752(+) TACTACAGCTTTACACTGAACTTTGAATATGCCTGTGTAGGATAGGTGGGAGGCTTTGAAGCTGGAGCGCTAGCTCCAGTGGAGCCAACCTTGAAATACCACCCTGGCGTGTTTGAGGTTCTAACCCTGTCCCGTAATCCGGGTCGGGAA 2233

NZ_JASNNV010000010.1:39284-43631(-) TACTACAGCTTTACACTGAACTTTGAATATGCCTGTGTAGGATAGGTGGGAGGCTTTGAAGCTGGAGCGCTAGCTCCAGTGGAGCCAACCTTGAAATACCACCCTGGCGTGTTTGAGGTTCTAACCCTGTCCCGTAATCCGGGTCGGGAA 2233

NZ_JAOXFC010000012.1:14231-18577(+) TACTACAGCTTTACACTGAACTTTGAATATGCCTGTGTAGGATAGGTGGGAGGCTTTGAAGCTGGAGCGCTAGCTCCAGTGGAGCCAACCTTGAAATACCACCCTGGCGTGTTTGAGGTTCTAACCCTGTCCCGTAATCCGGGTCGGGAA 2233

NZ_CP103430.1:167658-171997(+) TACTACAGCTTTACACTGAACTTTGAATATGCCTGTGTAGGATAGGTGGGAGGCTTTGAAGCTGGAGCGCTAGCTCCAGTGGAGCCAACCTTGAAATACCACCCTGGCGTGTTTGAGGTTCTAACCCTGTCCCGTAATCCGGGTCGGGAA 2233

NZ_PDLP01000011.1:14441-18787(+) TACTACAGCTTTACACTGAACTTTGAATATGCCTGTGTAGGATAGGTGGGAGGCTTTGAAGCTGGAGCGCTAGCTCCAGTGGAGCCAACCTTGAAATACCACCCTGGCGTGTTTGAGGTTCTAACCCTGTCCCGTAATCCGGGTCGGGAA 2233

NZ_NOLM01000022.1:91220-95566(-) TACTACAGCTTTACACTGAACTTTGAATATGCCTGTGTAGGATAGGTGGGAGGCTTTGAAGCTGGAGCGCTAGCTCCAGTGGAGCCAACCTTGAAATACCACCCTGGCGTGTTTGAGGTTCTAACCCTGTCCCGTAATCCGGGTCGGGAA 2233

JBJCIS010000056.1:0-2780(-) ------------------------------------------------------------------------------------------------------------------------------------------------------ 2233

NZ_CDBG01000001.1:179744-184090(+) TACTACAGCTTTACACTGAACTTTGAATATGCCTGTGTAGGATAGGTGGGAGGCTTTGAAGCTGGAGCGCTAGCTCCAGTGGAGCCAACCTTGAAATACCACCCTGGCGTGTTTGAGGTTCTAACCCTGTCCCGTAATCCGGGTCGGGAA 2233

NZ_CP103434.1:167622-171961(+) TACTACAGCTTTACACTGAACTTTGAATATGCCTGTGTAGGATAGGTGGGAGGCTTTGAAGCTGGAGCGCTAGCTCCAGTGGAGCCAACCTTGAAATACCACCCTGGCGTGTTTGAGGTTCTAACCCTGTCCCGTAATCCGGGTCGGGAA 2233

CP014354.1:76692-80603(-) TACTACAGCTTTACACTGAACTTTGAATATGCCTGTGTAGGATAGGTGGGAGGCTTTGAAGCTGGAGCGCTAGCTCCAGTGGAGCCAACCTTGAAATACCACCCTGGCGTGTTTGAGGTTCTAACCCTGTCCCGTAATCCGGGTCGGGAA 1764

NOLR01000002.1:131833-136207(+) TACTACAGCTTTACACTGAACTTTGAATATGCCTGTGTAGGATAGGTGGGAGGCTTTGAAGCTGGAGCGCTAGCTCCAGTGGAGCCAACCTTGAAATACCACCCTGGCGTGTTTGAGGTTCTAACCCTGTCCCGTAATCCGGGTCGGGAA 2243

PPFR01000008.1:34360-38734(-) TACTACAGCTTTACACTGAACTTTGAATATGCCTGTGTAGGATAGGTGGGAGGCTTTGAAGCTGGAGCGCTAGCTCCAGTGGAGCCAACCTTGAAATACCACCCTGGCGTGTTTGAGGTTCTAACCCTGTCCCGTAATCCGGGTCGGGAA 2243

PPFQ01000003.1:131083-135464(+) TACTACAGCTTTACACTGAACTTTGAATATGCCTGTGTAGGATAGGTGGGAGGCTTTGAAGCTGGAGCGCTAGCTCCAGTGGAGCCAACCTTGAAATACCACCCTGGCGTGTTTGAGGTTCTAACCCTGTCCCGTAATCCGGGTCGGGAA 2243

NZ_AKYP01000181.1:1647-6020(-) TACTACAGCTTTACACTGAACTTTGAATATGCCTGTGTAGGATAGGTGGGAGGCTTTGAAGCTGGAGCGCTAGCTCCAGTGGAGCCAACCTTGAAATACCACCCTGGCGTGTTTGAGGTTCTAACCCTGTCCCGTAATCCGGGTCGGGAA 2242

NOVI01000003.1:105955-110328(+) TACTACAGCTTTACACTGAACTTTGAATATGCCTGTGTAGGATAGGTGGGAGGCTTTGAAGCTGGAGCGCTAGCTCCAGTGGAGCCAACCTTGAAATACCACCCTGGCGTGTTTGAGGTTCTAACCCTGTCCCGTAATCCGGGTCGGGAA 2243

CP018150.1:339294-343661(+) TACTACAGCTTTACACTGAACTTTGAATATGCCTGTGTAGGATAGGTGGGAGGCTTTGAAGCTGGAGCGCTAGCTCCAGTGGAGCCAACCTTGAAATACCACCCTGGCGTGTTTGAGGTTCTAACCCTGTCCCGTAATCCGGGTCGGGAA 2243

Consensus CAGTGTATGGTGGGTAGTTTGACTGGGGCGGTCTCCTCCAAAAGAGTAACGGAGGAGTACAAAGGTACCCTCAGCACGGTCGGAAATCGTGCATTGTGTGCAAAGGCATAAGGGTGCTTGACTGCGAGACTGACAAGTCGAGCAGGTACG 2393

JAKFBC010000002.1:34496-38842(-) (45) CAGTGTATGGTGGGTAGTTTGACTGGGGCGGTCTCCTCCAAAAGAGTAACGGAGGAGTACAAAGGTACCCTCAGCACGGTCGGAAATCGTGCATTGTGTGCAAAGGCATAAGGGTGCTTGACTGCGAGACTGACAAGTCGAGCAGGTACG 2383

NZ_AP019757.1:1739264-1743610(-) (31) CAGTGTATGGTGGGTAGTTTGACTGGGGCGGTCTCCTCCAAAAGAGTAGCGGAGGAGTACAAAGGTACCCTCAGCACGGTCGGAAATCGTGCATTGTGTGCAAAGGCATAAGGGTGCTTGACTGCGAGACTGACAAGTCGAGCAGGTACG 2383

JBKOHN010000001.1:81530-85875(+) (25) CAGTGTATGGTGGGTAGTTTGACTGGGGCGGTCTCCTCCAAAAGAGTAACGGAGGAGTACAAAGGTACCCTCAGCACGGTCGGAAATCGTGCATTGTGTGCAAAGGCATAAGGGTGCTTGACTGCGAGACTGACAAGTCGAGCAGGTACG 2383

NC_011528.1:339307-343646(+) (14) CAGTGTATGGTGGGTAGTTTGACTGGGGCGGTCTCCTCCAAAAGAGTAACGGAGGAGTACAAAGGTACCCTCAGCACGGTCGGAAATCGTGCATTGTGTGCAAAGGCATAAGGGTGCTTGACTGCGAGACTGACAAGTCGAGCAGGTACG 2383

CP018005.1:167566-171940(+) (14) CAGTGTATGGTGGGTAGTTTGACTGGGGCGGTCTCCTCCAAAAGAGTAACGGAGGAGTACAAAGGTACCCTCAGCACGGTCGGAAATCGTGCATTGTGTGCAAAGGCATAAGGGTGCTTGACTGCGAGACTGACAAGTCGAGCAGGTACG 2393

NZ_CCAM010000005.1:71418-75764(+) (13) CAGTGTATGGTGGGTAGTTTGACTGGGGCGGTCTCCTCCAAAAGAGTAACGGAGGAGTACAAAGGTACCCTCAGCACGGTCGGAAATCGTGCATTGTGTGCAAAGGCATAAGGGTGCTTGACTGCGAGACTGACAAGTCGAGCAGGTACG 2383

NZ_CCXO01000001.1:317226-321572(+) (11) CAGTGTATGGTGGGTAGTTTGACTGGGGCGGTCTCCTCCAAAAGAGTAACGGAGGAGTACAAAGGTACCCTCAGCACGGTCGGAAATCGTGCATTGTGTGCAAAGGCATAAGGGTGCTTGACTGCGAGACTGACAAGTCGAGCAGGTACG 2383

NC_011527.1:1751498-1755844(-) (8) CAGTGTATGGTGGGTAGTTTGACTGGGGCGGTCTCCTCCAAAAGAGTAACGGAGGAGTACAAAGGTACCCTCAGCACGGTCGGAAATCGTGCATTGTGTGCAAAGGCATAAGGGTGCTTGACTGCGAGACTGACAAGTCGAGCAGGTACG 2383

NZ_CP032542.1:88036-92382(-) (8) CAGTGTATGGTGGGTAGTTTGACTGGGGCGGTCTCCTCCAAAAGAGTAACGGAGGAGTACAAAGGTACCCTCAGCACGGTCGGAAATCGTGCATTGTGTGCAAAGGCATAAGGGTGCTTGACTGCGAGACTGACAAGTCGAGCAGGTACG 2383

CP014551.1:161236-165610(+) (8) CAGTGTATGGTGGGTAGTTTGACTGGGGCGGTCTCCTCCAAAAGAGTAGCGGAGGAGTACAAAGGTACCCTCAGCACGGTCGGAAATCGTGCATTGTGTGCAAAGGCATAAGGGTGCTTGACTGCGAGACTGACAAGTCGAGCAGGTACG 2393

CP013667.1:88011-92385(-) (8) CAGTGTATGGTGGGTAGTTTGACTGGGGCGGTCTCCTCCAAAAGAGTAACGGAGGAGTACAAAGGTACCCTCAGCACGGTCGGAAATCGTGCATTGTGTGCAAAGGCATAAGGGTGCTTGACTGCGAGACTGACAAGTCGAGCAGGTACG 2393

CP107247.1:164350-168689(+) (7) CAGTGTATGGTGGGTAGTTTGACTGGGGCGGTCTCCTCCAAAAGAGTAACGGAGGAGTACAAAGGTATCCTCAGCACGGTCGGAAATCGTGCATTGTGTGCAAAGGCATAAGGGTGCTTGACTGCGAGACTGACAAGTCGAGCAGGTACG 2383

NZ_CP103435.1:167581-171920(+) (6) CAGTGTATGGTGGGTAGTTTGACTGGGGCGGTCTCCTCCAAAAGAGTAACGGAGGAGTACAAAGGTACCCTCAGCACGGTCGGAAATCGTGCATTGTGTGCAAAGGCATAAGGGTGCTTGACTGCGAGACTGACAAGTCGAGCAGGTACG 2383

CP014565.1:1711747-1716121(-) (3) CAGTGTATGGTGGGTAGTTTGACTGGGGCGGTCTCCTCCAAAAGAGTAACGGAGGAGTACAAAGGTACCCTCAGCACGGTCGGAAATCGTGCATTGTGTGCAAAGGCATAAGGGTGCTTGACTGCGAGACTGACAAGTCGAGCAGGTACG 2393

AAYJ01000007.1:0-1404(+) (3) CAGTGTATGGTGGGTAGTTTGACTGGGGCGGTCTCCTCCAAAAGAGTAACGGAGGAGTACAAAGGTACCCTCAGCACGGTCGGAAATCGTGCATTGTGTGCAAAGGCATAAGGGTGCTTGACTGCGAGACTGACAAGTCGAGCAGGTACG 422

NZ_JAOXDR010000002.1:16672-21025(-) (3) CAGTGTATGGTGGGTAGTTTGACTGGGGCGGTCTCCTCCAAAAGAGTAACGGAGGAGTACAAAGGTACCCTCAGCACGGTCGGAAATCGTGCATTGTGTGCAAAGGCATAAGGGTGCTTGACTGCGAGACTGACAAGTCGAGCAGGTACG 2383

NZ_CP103432.1:167699-172038(+) (3) CAGTGTATGGTGGGTAGTTTGACTGGGGCGGTCTCCTCCAAAAGAGTAACGGAGGAGTACAAAGGTACCCTCAGCACGGTCGGAAATCGTGCATTGTGTGCAAAGGCATAAGGGTGCTTGACTGCGAGACTGACAAGTCGAGCAGGTACG 2383

NZ_CP103431.1:167697-172036(+) (3) CAGTGTATGGTGGGTAGTTTGACTGGGGCGGTCTCCTCCAAAAGAGTAACGGAGGAGTACAAAGGTACCCTCAGCACGGTCGGAAATCGTGCATTGTGTGCAAAGGCATAAGGGTGCTTGACTGCGAGACTGACAAGTCGAGCAGGTACG 2383

NZ_CP103428.1:167591-171929(+) (2) CAGTGTATGGTGGGTAGTTTGACTGGGGCGGTCTCCTCCAAAAGAGTAACGGAGGAGTACAAAGGTACCCTCAGCACGGTCGGAAATCGTGCATTGTGTGCAAAGGCATAAGGGTGCTTGACTGCGAGACTGACAAGTCGAGCAGGTACG 2383

NZ_JANTNR010000002.1:115350-119696(+) (2) CAGTGTATGGTGGGTAGTTTGACTGGGGCGGTCTCCTCCAAAAGAGTAACGGAGGAGTACAAAGGTACCCTCAGCACGGTCGGAAATCGTGCATTGTGTGCAAAGGCATAAGGGTGCTTGACTGCGAGACTGACAAGTCGAGCAGGTACG 2383

JARBIR010004564.1:3782-8128(-) (2) CAGTGTATGGTGGGTAGTTTGACTGGGGCGGTCTCCTCCAAAAGAGTAACGGAGGAGTACAAAGGTACCCTCAGCACGGTCGGAAATCGTGCATTGTGTGCAAAGGCATAAGGGTGCTTGACTGCGAGACTGACAAGTCGAGCAGGTACG 2383

NC_009727.1:1888349-1892695(-) (2) CAGTGTATGGTGGGTAGTTTGACTGGGGCGGTCTCCTCCAAAAGAGTAACGGAGGAGTACAAAGGTACCCTCAGCACGGTCGGAAATCGTGCATTGTGTGCAAAGGCATAAGGGTGCTTGACTGCGAGACTGACAAGTCGAGCAGGTACG 2383

AAYJ01000139.1:0-660(+) (2) ------------------------------------------------------------------------------------------------------------------------------------------------------ 1209

CP032542.1:88018-92392(-) (2) CAGTGTATGGTGGGTAGTTTGACTGGGGCGGTCTCCTCCAAAAGAGTAACGGAGGAGTACAAAGGTACCCTCAGCACGGTCGGAAATCGTGCATTGTGTGCAAAGGCATAAGGGTGCTTGACTGCGAGACTGACAAGTCGAGCAGGTACG 2393

CP014559.1:232744-237117(+) (2) CAGTGTATGGTGGGTAGTTTGACTGGGGCGGTCTCCTCCAAAAGAGTAACGGAGGAGTACAAAGGTACCCTCAGCACGGTCGGAAATCGTGCATTGTGTGCAAAGGCATAAGGGTGCTTGACTGCGAGACTGACAAGTCGAGCAGGTACG 2393

CP007555.1:337408-341752(+) CAGTGTATGGTGGGTAGTTTGACTGGGGCGGTCTCCTCCAAAAGAGTAACGGAGGAGTACAAAGGTACCCTCAGCACGGTCGGAAATCGTGCATTGTGTGCAAAGGCATAAGGGTGCTTGACTGCGAGACTGACAAGTCGAGCAGGTACG 2381

NZ_CP014354.1:76710-81178(-) CAGTGTATGGTGGGTAGTTTGACTGGGGCGGTCTCCTCCAAAAGAGTAACGGAGGAGTACAAAGGTACCCTCAGCACGGTCGGAAATCGTGCATTGTGTGCAAAGGCATAAGGGTGCTTGACTGCGAGACTGACAAGTCGAGCAGGTACG 2389

CP000890.1:243129-244433(+) ------------------------------------------------------------------------------------------------------------------------------------------------------ 1209

NZ_CP115461.1:167576-171922(+) CAGTGTATGGTGGGTAGTTTGACTGGGGCGGTCTCCTCCAAAAGAGTAACGGAGGAGTACAAAGGTACCCTCAGCACGGTCGGAAATCGTGCATTGTGTGCAAAGGCATAAGGGTGCTTGACTGCGAGAGTGACGGCGCGAGCAGGTACG 2383

AP019759.1:167578-171919(+) CAGTGTATGGTGGGTAGTTTGACTGGGGCGGTCTCCTCCAAAAGAGTAACGGAGGAGTACAAAGGTACCCTCAGCACGGTCGGAAATCGTGCATTGTGTGCAAAGGCATAAGGGTGCTTGACTGCGAGACTGACAAGTCGAGCAGGTACG 2381

AP019757.1:1739267-1743608(-) CAGTGTATGGTGGGTAGTTTGACTGGGGCGGTCTCCTCCAAAAGAGTAGCGGAGGAGTACAAAGGTACCCTCAGCACGGTCGGAAATCGTGCATTGTGTGCAAAGGCATAAGGGTGCTTGACTGCGAGACTGACAAGTCGAGCAGGTACG 2381

NOLN01000021.1:90861-95235(-) CAGTGTATGGTGGGTAGTTTGACTGGGGCGGTCTCCTCCAAAAGAGTAACGGAGGAGTACAAAGGTACCCTCAGCACGGTCGGAAATCGTGCATTGTGTGCAAAGGCATAAGGGTGCTTGACTGCGAGACTGACAAGTCGAGCAGGTACG 2393

NOLM01000022.1:91202-95576(-) CAGTGTATGGTGGGTAGTTTGACTGGGGCGGTCTCCTCCAAAAGAGTAACGGAGGAGTACAAAGGTACCCTCAGCACGGTCGGAAATCGTGCATTGTGTGCAAAGGCATAAGGGTGCTTGACTGCGAGACTGACAAGTCGAGCAGGTACG 2393

PDLP01000011.1:14431-18805(+) CAGTGTATGGTGGGTAGTTTGACTGGGGCGGTCTCCTCCAAAAGAGTAACGGAGGAGTACAAAGGTACCCTCAGCACGGTCGGAAATCGTGCATTGTGTGCAAAGGCATAAGGGTGCTTGACTGCGAGACTGACAAGTCGAGCAGGTACG 2393

CP014563.1:522940-527314(+) CAGTGTATGGTGGGTAGTTTGACTGGGGCGGTCTCCTCCAAAAGAGTAACGGAGGAGTACAAAGGTACCCTCAGCACGGTCGGAAATCGTGCATTGTGTGCAAAGGCATAAGGGTGCTTGACTGCGAGACTGACAAGTCGAGCAGGTACG 2393

CP000890.1:244922-245905(+) CAGTGTATGGTGGGTAGTTTGACTGGGGCGGTCTCCTCCAAAAGAGTAACGGAGGAGTACAAAGGTACCCTCAGCACGGTCGGAAATCGTGCATTGTGTGCAAAGGCATAAGGGTGCTTGACTGCGAGACTGACAAGTCGAGCAGGTACG 422

NZ_NOLR01000002.1:131843-136189(+) CAGTGTATGGTGGGTAGTTTGACTGGGGCGGTCTCCTCCAAAAGAGTAGCGGAGGAGTACAAAGGTACCCTCAGCACGGTCGGAAATCGTGCATTGTGTGCAAAGGCATAAGGGTGCTTGACTGCGAGACTGACAAGTCGAGCAGGTACG 2383

NZ_JAOXDP010000002.1:15794-20139(-) CAGTGTATGGTGGGTAGTTTGACTGGGGCGGTCTCCTCCAAAAGAGTAACGGAGGAGTACAAAGGTACCCTCAGCACGGTCGGAAATCGTGCATTGTGTGCAAAGGCATAAGGGTGCTTGACTGCGAGACTGACAAGTCGAGCAGGTACG 2383

NZ_PPFR01000008.1:34378-38724(-) CAGTGTATGGTGGGTAGTTTGACTGGGGCGGTCTCCTCCAAAAGAGTAACGGAGGAGTACAAAGGTACCCTCAGCACGGTCGGAAATCGTGCATTGTGTGCAAAGGCATAAGGGTGCTTGACTGCGAGACTGACAAGTCGAGCAGGTACG 2383

NZ_PPFQ01000003.1:131093-135446(+) CAGTGTATGGTGGGTAGTTTGACTGGGGCGGTCTCCTCCAAAAGAGTAGCGGAGGAGTACAAAGGTACCCTCAGCACGGTCGGAAATCGTGCATTGTGTGCAAAGGCATAAGGGTGCTTGACTGCGAGACTGACAAGTCGAGCAGGTACG 2383

NZ_LK937696.1:167613-171953(+) CAGTGTATGGTGGGTAGTTTGACTGGGGCGGTCTCCTCCAAAAGAGTAACGGAGGAGTACAAAGGTACCCTCAGCACGGTCGGAAATCGTGCATTGTGTGCAAAGGCATAAGGGTGCTTGACTGCGAGACTGACAAGTCGAGCAGGTACG 2383

NZ_NOVI01000003.1:105965-110310(+) CAGTGTATGGTGGGTAGTTTGACTGGGGCGGTCTCCTCCAAAAGAGTAACGGAGGAGTACAAAGGTACCCTCAGCACGGTCGGAAATCGTGCATTGTGTGCAAAGGCATAAGGGTGCTTGACTGCGAGACTGACAAGTCGAGCAGGTACG 2383

NZ_CP103426.1:167642-171981(+) CAGTGTATGGTGGGTAGTTTGACTGGGGCGGTCTCCTCCAAAAGAGTAACGGAGGAGTACAAAGGTACCCTCAGCACGGTCGGAAATCGTGCATTGTGTGCAAAGGCATAAGGGTGCTTGACTGCGAGACTGACAAGTCGAGCAGGTACG 2383

NZ_JAOXDN010000007.1:36985-41324(-) CAGTGTATGGTGGGTAGTTTGACTGGGGCGGTCTCCTCCAAAAGAGTAACGGAGGAGTACAAAGGTACCCTCAGCACGGTCGGAAATCGTGCATTGTGTGCAAAGGCATAAGGGTGCTTGACTGCGAGACTGACAAGTCGAGCAGGTACG 2383

NZ_JPVV01000013.1:13888-18051(+) CAGTGTATGGTGGGTAGTTTGACTGGGGCGGTCTCCTCCAAAAGAGTAACGGAGGAGTACAAAGGTACCCTCAGCACGGTCGGAAATCGTGCATTGTGTGCAAAGGCATAAGGGTGCTTGACTGCGAGACTGACAAGTCGAGCAGGTACG 2383

NZ_CP007555.1:337406-341752(+) CAGTGTATGGTGGGTAGTTTGACTGGGGCGGTCTCCTCCAAAAGAGTAACGGAGGAGTACAAAGGTACCCTCAGCACGGTCGGAAATCGTGCATTGTGTGCAAAGGCATAAGGGTGCTTGACTGCGAGACTGACAAGTCGAGCAGGTACG 2383

NZ_JASNNV010000010.1:39284-43631(-) CAGTGTATGGTGGGTAGTTTGACTGGGGCGGTCTCCTCCAAAAGAGTAACGGAGGAGTACAAAGGTACCCTCAGCACGGTCGGAAATCGTGCATTGTGTGCAAAGGCATAAGGGTGCTTGACTGCGAGACTGACAAGTCGAGCGGGTACG 2383

NZ_JAOXFC010000012.1:14231-18577(+) CAGTGTATGGTGGGTAGTTTGACTGGGGCGGTCTCCTCCAAAAGAGTAACGGAGGAGTACAAAGGTACCCTCAGCACGGTCGGAAATCGTGCATTGTGTGCAAAGGCATAAGGGTGCTTGACTGCGAGACTGACAAGTCGAGCAGGTACG 2383

NZ_CP103430.1:167658-171997(+) CAGTGTATGGTGGGTAGTTTGACTGGGGCGGTCTCCTCCAAAAGAGTAACGGAGGAGTACAAAGGTACCCTCAGCACGGTCGGAAATCGTGCATTGTGTGCAAAGGCATAAGGGTGCTTGACTGCGAGACTGACAAGTCGAGCAGGTACG 2383

NZ_PDLP01000011.1:14441-18787(+) CAGTGTATGGTGGGTAGTTTGACTGGGGCGGTCTCCTCCAAAAGAGTAACGGAGGAGTACAAAGGTACCCTCAGCACGGTCGGAAATCGTGCATTGTGTGCAAAGGCATAAGGGTGCTTGACTGCGAGACTGACAAGTCGAGCAGGTACG 2383

NZ_NOLM01000022.1:91220-95566(-) CAGTGTATGGTGGGTAGTTTGACTGGGGCGGTCTCCTCCAAAAGAGTAACGGAGGAGTACAAAGGTACCCTCAGCACGGTCGGAAATCGTGCATTGTGTGCAAAGGCATAAGGGTGCTTGACTGCGAGACTGACAAGTCGAGCAGGTACG 2383

JBJCIS010000056.1:0-2780(-) ------------------------------------------------------------------------------------------------------------------------------------------------------ 2383

NZ_CDBG01000001.1:179744-184090(+) CAGTGTATGGTGGGTAGTTTGACTGGGGCGGTCTCCTCCAAAAGAGTAACGGAGGAGTACAAAGGTACCCTCAGCACGGTCGGAAATCGTGCATTGTGTGCAAAGGCATAAGGGTGCTTGACTGCGAGACTGACAAGTCGAGCAGGTACG 2383

NZ_CP103434.1:167622-171961(+) CAGTGTATGGTGGGTAGTTTGACTGGGGCGGTCTCCTCCAAAAGAGTAACGGAGGAGTACAAAGGTACCCTCAGCACGGTCGGAAATCGTGCATTGTGTGCAAAGGCATAAGGGTGCTTGACTGCGAGACTGACAAGTCGAGCAGGTACG 2383

CP014354.1:76692-80603(-) CAGTGTATGGTGGGTAGTTTGACTGGGGCGGTCTCCTCCAAAAGAGTAACGGAGGAGTACAAAGGTACCCTCAGCACGGTCGGAAATCGTGCATTGTGTGCAAAGGCATAAGGGTGCTTGACTGCGAGACTGACAAGTCGAGCAGGTACG 1914

NOLR01000002.1:131833-136207(+) CAGTGTATGGTGGGTAGTTTGACTGGGGCGGTCTCCTCCAAAAGAGTAGCGGAGGAGTACAAAGGTACCCTCAGCACGGTCGGAAATCGTGCATTGTGTGCAAAGGCATAAGGGTGCTTGACTGCGAGACTGACAAGTCGAGCAGGTACG 2393

PPFR01000008.1:34360-38734(-) CAGTGTATGGTGGGTAGTTTGACTGGGGCGGTCTCCTCCAAAAGAGTAACGGAGGAGTACAAAGGTACCCTCAGCACGGTCGGAAATCGTGCATTGTGTGCAAAGGCATAAGGGTGCTTGACTGCGAGACTGACAAGTCGAGCAGGTACG 2393

PPFQ01000003.1:131083-135464(+) CAGTGTATGGTGGGTAGTTTGACTGGGGCGGTCTCCTCCAAAAGAGTAGCGGAGGAGTACAAAGGTACCCTCAGCACGGTCGGAAATCGTGCATTGTGTGCAAAGGCATAAGGGTGCTTGACTGCGAGACTGACAAGTCGAGCAGGTACG 2393

NZ_AKYP01000181.1:1647-6020(-) CAGTGTATGGTGGGTAGTTTGACTGGGGCGGTCTCCTCCAAAAGAGTAACGGAGGAGTACAAAGGTACCCTCAGCACGGTCGGAAATCGTGCATTGTGTGCAAAGGCATAAGGGTGCTTGACTGCGAGACTGACAAGTCGAGCAGGTACG 2392

NOVI01000003.1:105955-110328(+) CAGTGTATGGTGGGTAGTTTGACTGGGGCGGTCTCCTCCAAAAGAGTAACGGAGGAGTACAAAGGTACCCTCAGCACGGTCGGAAATCGTGCATTGTGTGCAAAGGCATAAGGGTGCTTGACTGCGAGACTGACAAGTCGAGCAGGTACG 2393

CP018150.1:339294-343661(+) CAGTGTATGGTGGGTAGTTTGACTGGGGCGGTCTCCTCCAAAAGAGTAACGGAGGAGTACAAAGGTACCCTCAGCACGGTCGGAAATCGTGCATTGTGTGCAAAGGCATAAGGGTGCTTGACTGCGAGACTGACAAGTCGAGCAGGTACG 2393

Consensus AAAGTAGGTCTTAGTGATCCGGTGGTCCTTTATGGAAGGGCCATC----GCTCAACGGATAAAAGGTACTCCGGGGATAACAGGCTGATACCACCCAAGAGTTCATATCGACGGTGGTGTTTGGCACCTCGATGTCGGCTCATCACATCC 2539

JAKFBC010000002.1:34496-38842(-) (45) AAAGTAGGTCTTAGTGATCCGGTGGTCCTTTATGGAAGGGCCATC----GCTCAACGGATAAAAGGTACTCCGGGGATAACAGGCTGATACCACCCAAGAGTTCATATCGACGGTGGTGTTTGGCACCTCGATGTCGGCTCATCACATCC 2529

NZ_AP019757.1:1739264-1743610(-) (31) AAAGTAGGTCTTAGTGATCCGGTGGTCCTTTATGGAAGGGCCATC----GCTCAACGGATAAAAGGTACTCCGGGGATAACAGGCTGATACCACCCAAGAGTTCATATCGACGGTGGTGTTTGGCACCTCGATGTCGGCTCATCACATCC 2529

JBKOHN010000001.1:81530-85875(+) (25) AAAGTAGGTCTTAGTGATCCGGTGGTCCTTTATGGAAGGGCCATC----GCTCAACGGATAAAAGGTACTCCGGGGATAACAGGCTGATACCACCCAAGAGTTCATATCGACGGTGGTGTTTGGCACCTCGATGTCGGCTCATCACATCC 2529

NC_011528.1:339307-343646(+) (14) AAAGTAGGTCTTAGTGATCCGGTGGTCCTTTATGGAAGGGCCATC----GCTCAACGGATAAAAGGTACTCCGGGGATAACAGGCTGATACCACCCAAGAGTTCATATCGACGGTGGTGTTTGGCACCTCGATGTCGGCTCATCACATCC 2529

CP018005.1:167566-171940(+) (14) AAAGTAGGTCTTAGTGATCCGGTGGTCCTTTATGGAAGGGCCATC----GCTCAACGGATAAAAGGTACTCCGGGGATAACAGGCTGATACCACCCAAGAGTTCATATCGACGGTGGTGTTTGGCACCTCGATGTCGGCTCATCACATCC 2539

NZ_CCAM010000005.1:71418-75764(+) (13) AAAGTAGGTCTTAGTGATCCGGTGGTCCTTTATGGAAGGGCCATC----GCTCAACGGATAAAAGGTACTCCGGGGATAACAGGCTGATACCACCCAAGAGTTCATATCGACGGTGGTGTTTGGCACCTCGATGTCGGCTCATCACATCC 2529

NZ_CCXO01000001.1:317226-321572(+) (11) AAAGTAGGTCTTAGTGATCCGGTGGTCCTTTATGGAAGGGCCATC----GCTCAACGGATAAAAGGTACTCCGGGGATAACAGGCTGATACCACCCAAGAGTTCATATCGACGGTGGTGTTTGGCACCTCGATGTCGGCTCATCACATCC 2529

NC_011527.1:1751498-1755844(-) (8) AAAGTAGGTCTTAGTGATCCGGTGGTCCTTTATGGAAGGGCCATC----GCTCAACGGATAAAAGGTACTCCGGGGATAACAGGCTGATACCACCCAAGAGTTCATATCGACGGTGGTGTTTGGCACCTCGATGTCGGCTCATCACATCC 2529

NZ_CP032542.1:88036-92382(-) (8) AAAGTAGGTCTTAGTGATCCGGTGGTCCTTTATGGAAGGGCCATC----GCTCAACGGATAAAAGGTACTCCGGGGATAACAGGCTGATACCACCCAAGAGTTCATATCGACGGTGGTGTTTGGCACCTCGATGTCGGCTCATCACATCC 2529

CP014551.1:161236-165610(+) (8) AAAGTAGGTCTTAGTGATCCGGTGGTCCTTTATGGAAGGGCCATC----GCTCAACGGATAAAAGGTACTCCGGGGATAACAGGCTGATACCACCCAAGAGTTCATATCGACGGTGGTGTTTGGCACCTCGATGTCGGCTCATCACATCC 2539

CP013667.1:88011-92385(-) (8) AAAGTAGGTCTTAGTGATCCGGTGGTCCTTTATGGAAGGGCCATC----GCTCAACGGATAAAAGGTACTCCGGGGATAACAGGCTGATACCACCCAAGAGTTCATATCGACGGTGGTGTTTGGCACCTCGATGTCGGCTCATCACATCC 2539

CP107247.1:164350-168689(+) (7) AAAGTAGGTCTTAGTGATCCGGTGGTCCTTTATGGAAGGGCCATC----GCTCAACGGATAAAAGGTACTCCGGGGATAACAGGCTGATACCACCCAAGAGTTCATATCGACGGTGGTGTTTGGCACCTCGATGTCGGCTCATCACATCC 2529

NZ_CP103435.1:167581-171920(+) (6) AAAGTAGGTCTTAGTGATCCGGTGGTCCTTTATGGAAGGGCCATC----GCTCAACGGATAAAAGGTACTCCGGGGATAACAGGCTGATACCACCCAAGAGTTCATATCGACGGTGGTGTTTGGCACCTCGATGTCGGCTCATCACATCC 2529

CP014565.1:1711747-1716121(-) (3) AAAGTAGGTCTTAGTGATCCGGTGGTCCTTTATGGAAGGGCCATC----GCTCAACGGATAAAAGGTACTCCGGGGATAACAGGCTGATACCACCCAAGAGTTCATATCGACGGTGGTGTTTGGCACCTCGATGTCGGCTCATCACATCC 2539

AAYJ01000007.1:0-1404(+) (3) AAAGTAGGTCTTAGTGATCCGGTGGTCCTTTATGGAAGGGCCATC----GCTCAACGGATAAAAGGTACTCCGGGGATAACAGGCTGATACCACCCAAGAGTTCATATCGACGGTGGTGTTTGGCACCTCGATGTCGGCTCATCACATCC 568

NZ_JAOXDR010000002.1:16672-21025(-) (3) AAAGTAGGTCTTAGTGATCCGGTGGTCCTTTATGGAAGGGCCATC----GCTCAACGGATAAAAGGTACTCCGGGGATAACAGGCTGATACCACCCAAGAGTTCATATCGACGGTGGTGTTTGGCACCTCGATGTCGGCTCATCACATCC 2529

NZ_CP103432.1:167699-172038(+) (3) AAAGTAGGTCTTAGTGATCCGGTGGTCCTTTATGGAAGGGCCATC----GCTCAACGGATAAAAGGTACTCCGGGGATAACAGGCTGATACCACCCAAGAGTTCATATCGACGGTGGTGTTTGGCACCTCGATGTCGGCTCATCACATCC 2529

NZ_CP103431.1:167697-172036(+) (3) AAAGTAGGTCTTAGTGATCCGGTGGTCCTTTATGGAAGGGCCATC----GCTCAACGGATAAAAGGTACTCCGGGGATAACAGGCTGATACCACCCAAGAGTTCATATCGACGGTGGTGTTTGGCACCTCGATGTCGGCTCATCACATCC 2529

NZ_CP103428.1:167591-171929(+) (2) AAAGTAGGTCTTAGTGATCCGGTGGTCCTTTATGGAAGGGCCATC----GCTCAACGGATAAAAGGTACTCCGGGGATAACAGGCTGATACCACCCAAGAGTTCATATCGACGGTGGTGTTTGGCACCTCGATGTCGGCTCATCACATCC 2529

NZ_JANTNR010000002.1:115350-119696(+) (2) AAAGTAGGTCTTAGTGATCCGGTGGTCCTTTATGGAAGGGCCATC----GCTCAACGGATAAAAGGTACTCCGGGGATAACAGGCTGATACCACCCAAGAGTTCATATCGACGGTGGTGTTTGGCACCTCGATGTCGGCTCATCACATCC 2529

JARBIR010004564.1:3782-8128(-) (2) AAAGTAGGTCTTAGTGATCCGGTGGTCCTTTATGGAAGGGCCATC----GCTCAACGGATAAAAGGTACTCCGGGGATAACAGGCTGATACCACCCAAGAGTTCATATCGACGGTGGTGTTTGGCACCTCGATGTCGGCTCATCACATCC 2529

NC_009727.1:1888349-1892695(-) (2) AAAGTAGGTCTTAGTGATCCGGTGGTCCTTTATGGAAGGGCCATC----GCTCAACGGATAAAAGGTACTCCGGGGATAACAGGCTGATACCACCCAAGAGTTCATATCGACGGTGGTGTTTGGCACCTCGATGTCGGCTCATCACATCC 2529

AAYJ01000139.1:0-660(+) (2) ------------------------------------------------------------------------------------------------------------------------------------------------------ 1359

CP032542.1:88018-92392(-) (2) AAAGTAGGTCTTAGTGATCCGGTGGTCCTTTATGGAAGGGCCATC----GCTCAACGGATAAAAGGTACTCCGGGGATAACAGGCTGATACCACCCAAGAGTTCATATCGACGGTGGTGTTTGGCACCTCGATGTCGGCTCATCACATCC 2539

CP014559.1:232744-237117(+) (2) AAAGTAGGTCTTAGTGATCCGGTGGTCCTTTATGGAAGGGCCATC----GCTCAACGGATAAAAGGTACTCCGGGGATAACAGGCTGATACCACCCAAGAGTTCATATCGACGGTGGTGTTTGGCACCTCGATGTCGGCTCATCACATCC 2539

CP007555.1:337408-341752(+) AAAGTAGGTCTTAGTGATCCGGTGGTCCTTTATGGAAGGGCCATC----GCTCAACGGATAAAAGGTACTCCGGGGATAACAGGCTGATACCACCCAAGAGTTCATATCGACGGTGGTGTTTGGCACCTCGATGTCGGCTCATCACATCC 2527

NZ_CP014354.1:76710-81178(-) AAAGTAGGTCTTAGTGATCCGGTGGTCCTTTATGGAAGGGCCATCGCTCGCTCAACGGATAAAAGGTACTCCGGGGATAACAGGCTGATACCACCCAAGAGTTCATATCGACGGTGGTGTTTGGCACCTCGATGTCGGCTCATCACATCC 2539

CP000890.1:243129-244433(+) ------------------------------------------------------------------------------------------------------------------------------------------------------ 1359

NZ_CP115461.1:167576-171922(+) AAAGTAGGTCTTAGTGATCCGGTGGTTCTGCATGGAAGGGCCATC----GCTCAACGGATAAAAGGTACTCCGGGGATAACAGGCTGATACCGCCCAAGAGTTCATATCGACGGCGGTGTTTGGCACCTCGATGTCGGCTCATCACATCC 2529

AP019759.1:167578-171919(+) AAAGTAGGTCTTAGTGATCCGGTGGTCCTTTATGGAAGGGCCATC----GCTCAACGGATAAAAGGTACTCCGGGGATAACAGGCTGATACCACCCAAGAGTTCATATCGACGGTGGTGTTTGGCACCTCGATGTCGGCTCATCACATCC 2527

AP019757.1:1739267-1743608(-) AAAGTAGGTCTTAGTGATCCGGTGGTCCTTTATGGAAGGGCCATC----GCTCAACGGATAAAAGGTACTCCGGGGATAACAGGCTGATACCACCCAAGAGTTCATATCGACGGTGGTGTTTGGCACCTCGATGTCGGCTCATCACATCC 2527

NOLN01000021.1:90861-95235(-) AAAGTAGGTCTTAGTGATCCGGTGGTCCTTTATGGAAGGGCCATC----GCTCAACGGATAAAAGGTACTCCGGGGATAACAGGCTGATACCACCCAAGAGTTCATATCGACGGTGGTGTTTGGCACCTCGATGTCGGCTCATCACATCC 2539

NOLM01000022.1:91202-95576(-) AAAGTAGGTCTTAGTGATCCGGTGGTCCTTTATGGAAGGGCCATC----GCTCAACGGATAAAAGGTACTCCGGGGATAACAGGCTGATACCACCCAAGAGTTCATATCGACGGTGGTGTTTGGCACCTCGATGTCGGCTCATCACATCC 2539

PDLP01000011.1:14431-18805(+) AAAGTAGGTCTTAGTGATCCGGTGGTCCTTTATGGAAGGGCCATC----GCTCAACGGATAAAAGGTACTCCGGGGATAACAGGCTGATACCACCCAAGAGTTCATATCGACGGTGGTGTTTGGCACCTCGATGTCGGCTCATCACATCC 2539

CP014563.1:522940-527314(+) AAAGTAGGTCTTAGTGATCCGGTGGTCCTTTATGGAAGGGCCATC----GCTCAACGGATAAAAGGTACTCCGGGGATAACAGGCTGATACCACCCAAGAGTTCATATCGACGGTGGTGTTTGGCACCTCGATGTCGGCTCATCACATCC 2539

CP000890.1:244922-245905(+) AAAGTAGGTCTTAGTGATCCGGTGGTCCTTTATGGAAGGGCCATC----GCTCAACGGATAAAAGGTACTCCGGGGATAACAGGCTGATACCACCCAAGAGTTCATATCGACGGTGGTGTTTGGCACCTCGATGTCGGCTCATCACATCC 568

NZ_NOLR01000002.1:131843-136189(+) AAAGTAGGTCTTAGTGATCCGGTGGTCCTTTATGGAAGGGCCATC----GCTCAACGGATAAAAGGTACTCCGGGGATAACAGGCTGATACCACCCAAGAGTTCATATCGACGGTGGTGTTTGGCACCTCGATGTCGGCTCATCACATCC 2529

NZ_JAOXDP010000002.1:15794-20139(-) AAAGTAGGTCTTAGTGATCCGGTGGTCCTTTATGGAAGGGCCATC----GCTCAACGGATAAAAGGTACTCCGGGGATAACAGGCTGATACCACCCAAGAGTTCATATCGACGGTGGTGTTTGGCACCTCGATGTCGGCTCATCACATCC 2529

NZ_PPFR01000008.1:34378-38724(-) AAAGTAGGTCTTAGTGATCCGGTGGTCCTTTATGGAAGGGCCATC----GCTCAACGGATAAAAGGTACTCCGGGGATAACAGGCTGATACCACCCAAGAGTTCATATCGACGGTGGTGTTTGGCACCTCGATGTCGGCTCATCACATCC 2529

NZ_PPFQ01000003.1:131093-135446(+) AAAGTAGGTCTTAGTGATCCGGTGGTCCTTTATGGAAGGGCCATC----GCTCAACGGATAAAAGGTACTCCGGGGATAACAGGCTGATACCACCCAAGAGTTCATATCGACGGTGGTGTTTGGCACCTCGATGTCGGCTCATCACATCC 2529

NZ_LK937696.1:167613-171953(+) AAAGTAGGTCTTAGTGATCCGGTGGTCCTTTATGGAAGGGCCATC----GCTCAACGGATAAAAGGTACTCCGGGGATAACAGGCTGATACCACCCAAGAGTTCATATCGACGGTGGTGTTTGGCACCTCGATGTCGGCTCATCACATCC 2529

NZ_NOVI01000003.1:105965-110310(+) AAAGTAGGTCTTAGTGATCCGGTGGTCCTTTATGGAAGGGCCATC----GCTCAACGGATAAAAGGTACTCCGGGGATAACAGGCTGATACCACCCAAGAGTTCATATCGACGGTGGTGTTTGGCACCTCGATGTCGGCTCATCACATCC 2529

NZ_CP103426.1:167642-171981(+) AAAGTAGGTCTTAGTGATCCGGTGGTCCTTTATGGAAGGGCCATC----GCTCAACGGATAAAAGGTACTCCGGGGATAACAGGCTGATACCACCCAAGAGTTCATATCGACGGTGGTGTTTGGCACCTCGATGTCGGCTCATCACATCC 2529

NZ_JAOXDN010000007.1:36985-41324(-) AAAGTAGGTCTTAGTGATCCGGTGGTCCTTTATGGAAGGGCCATC----GCTCAACGGATAAAAGGTACTCCGGGGATAACAGGCTGATACCACCCAAGAGTTCATATCGACGGTGGTGTTTGGCACCTCGATGTCGGCTCATCACATCC 2529

NZ_JPVV01000013.1:13888-18051(+) AAAGTAGGTCTTAGTGATCCGGTGGTCCTTTATGGAAGGGCCATC----GCTCAACGGATAAAAGGTACTCCGGGGATAACAGGCTGATACCACCCAAGAGTTCATATCGACGGTGGTGTTTGGCACCTCGATGTCGGCTCATCACATCC 2529

NZ_CP007555.1:337406-341752(+) AAAGTAGGTCTTAGTGATCCGGTGGTCCTTTATGGAAGGGCCATC----GCTCAACGGATAAAAGGTACTCCGGGGATAACAGGCTGATACCACCCAAGAGTTCATATCGACGGTGGTGTTTGGCACCTCGATGTCGGCTCATCACATCC 2529

NZ_JASNNV010000010.1:39284-43631(-) AAAGTAGGTCTTAGTGATCCGGTGGTCCTTTATGGAAGGGCCATC----GCTCAACGGATAA-AGGTACTCCGGGGATAACAGGCTGATACCACCCAAGAGTTCATATCGACGGTGGTGTTTGGCACCTCGATGTCGGCTCATCACATCC 2528

NZ_JAOXFC010000012.1:14231-18577(+) AAAGTAGGTCTTAGTGATCCGGTGGTCCTTTATGGAAGGGCCATC----GCTCAACGGATAAAAGGTACTCCGGGGATAACAGGCTGATACCACCCAAGAGTTCATATCGACGGTGGTGTTTGGCACCTCGATGTCGGCTCATCACATCC 2529

NZ_CP103430.1:167658-171997(+) AAAGTAGGTCTTAGTGATCCGGTGGTCCTTTATGGAAGGGCCATC----GCTCAACGGATAAAAGGTACTCCGGGGATAACAGGCTGATACCACCCAAGAGTTCATATCGACGGTGGTGTTTGGCACCTCGATGTCGGCTCATCACATCC 2529

NZ_PDLP01000011.1:14441-18787(+) AAAGTAGGTCTTAGTGATCCGGTGGTCCTTTATGGAAGGGCCATC----GCTCAACGGATAAAAGGTACTCCGGGGATAACAGGCTGATACCACCCAAGAGTTCATATCGACGGTGGTGTTTGGCACCTCGATGTCGGCTCATCACATCC 2529

NZ_NOLM01000022.1:91220-95566(-) AAAGTAGGTCTTAGTGATCCGGTGGTCCTTTATGGAAGGGCCATC----GCTCAACGGATAAAAGGTACTCCGGGGATAACAGGCTGATACCACCCAAGAGTTCATATCGACGGTGGTGTTTGGCACCTCGATGTCGGCTCATCACATCC 2529

JBJCIS010000056.1:0-2780(-) ------------------------------------------------------------------------------------------------------------------------------------------------------ 2533

NZ_CDBG01000001.1:179744-184090(+) AAAGTAGGTCTTAGTGATCCGGTGGTCCTTTATGGAAGGGCCATC----GCTCAACGGATAAAAGGTACTCCGGGGATAACAGGCTGATACCACCCAAGAGTTCATATCGACGGTGGTGTTTGGCACCTCGATGTCGGCTCATCACATCC 2529

NZ_CP103434.1:167622-171961(+) AAAGTAGGTCTTAGTGATCCGGTGGTCCTTTATGGAAGGGCCATC----GCTCAACGGATAAAAGGTACTCCGGGGATAACAGGCTGATACCACCCAAGAGTTCATATCGACGGTGGTGTTTGGCACCTCGATGTCGGCTCATCACATCC 2529

CP014354.1:76692-80603(-) AAAGTAGGTCTTAGTGATCCGGTGGTCCTTTATGGAAGGGCCATCGCTCGCTCAACGGATAAAAGGTACTCCGGGGATAACAGGCTGATACCACCCAAGAGTTCATATCGACGGTGGTGTTTGGCACCTCGATGTCGGCTCATCACATCC 2064

NOLR01000002.1:131833-136207(+) AAAGTAGGTCTTAGTGATCCGGTGGTCCTTTATGGAAGGGCCATC----GCTCAACGGATAAAAGGTACTCCGGGGATAACAGGCTGATACCACCCAAGAGTTCATATCGACGGTGGTGTTTGGCACCTCGATGTCGGCTCATCACATCC 2539

PPFR01000008.1:34360-38734(-) AAAGTAGGTCTTAGTGATCCGGTGGTCCTTTATGGAAGGGCCATC----GCTCAACGGATAAAAGGTACTCCGGGGATAACAGGCTGATACCACCCAAGAGTTCATATCGACGGTGGTGTTTGGCACCTCGATGTCGGCTCATCACATCC 2539

PPFQ01000003.1:131083-135464(+) AAAGTAGGTCTTAGTGATCCGGTGGTCCTTTATGGAAGGGCCATC----GCTCAACGGATAAAAGGTACTCCGGGGATAACAGGCTGATACCACCCAAGAGTTCATATCGACGGTGGTGTTTGGCACCTCGATGTCGGCTCATCACATCC 2539

NZ_AKYP01000181.1:1647-6020(-) AAAGTAGGTCTTAGTGATCCGGTGGTCCTTTATGGAAGGGCCATC----GCTCAACGGATAAAAGGTACTCCGGGGATAACAGGCTGATACCACCCAAGAGTTCATATCGACGGTGGTGTTTGGCACCTCGATGTCGGCTCATCACATCC 2538

NOVI01000003.1:105955-110328(+) AAAGTAGGTCTTAGTGATCCGGTGGTCCTTTATGGAAGGGCCATC----GCTCAACGGATAAAAGGTACTCCGGGGATAACAGGCTGATACCACCCAAGAGTTCATATCGACGGTGGTGTTTGGCACCTCGATGTCGGCTCATCACATCC 2539

CP018150.1:339294-343661(+) AAAGTAGGTCTTAGTGATCCGGTGGTCCTTTATGGAAGGGCCATC----GCTCAACGGATAAAAGGTACTCCGGGGATAACAGGCTGATACCACCCAAGAGTTCATATCGACGGTGGTGTTTGGCACCTCGATGTCGGCTCATCACATCC 2539

Consensus TGGGGCTGTAGCCGGTCCCAAGGGTATGGCTGTTCGCCATTTAAAGTGGTACGTGAGCTGGGTTTAGAACGTCGTGAGACAGTTCGGTCCCTATCTGCCGTGGGCGTTGGAGATTTGAGAGGAGCTGCTCCTAGTACGAGAGGAC--CGG 2687

JAKFBC010000002.1:34496-38842(-) (45) TGGGGCTGTAGCCGGTCCCAAGGGTATGGCTGTTCGCCATTTAAAGTGGTACGTGAGCTGGGTTTAGAACGTCGTGAGACAGTTCGGTCCCTATCTGCCGTGGGCGTTGGAGATTTGAGAGGAGCTGCTCCTAGTACGAGAGGAC--CGG 2677

NZ_AP019757.1:1739264-1743610(-) (31) TGGGGCTGTAGCCGGTCCCAAGGGTATGGCTGTTCGCCATTTAAAGTGGTACGTGAGCTGGGTTTAGAACGTCGTGAGACAGTTCGGTCCCTATCTGCCGTGGGCGTTGGAGATTTGAGAGGAGCTGCTCCTAGTACGAGAGGAC--CGG 2677

JBKOHN010000001.1:81530-85875(+) (25) TGGGGCTGTAGCCGGTCCCAAGGGTATGGCTGTTCGCCATTTAAAGTGGTACGTGAGCTGGGTTTAGAACGTCGTGAGACAGTTCGGTCCCTATCTGCCGTGGGCGTTGGAGATTTGAGAGGAGCTGCTCCTAGTACGAGAGGAC--CGG 2677

NC_011528.1:339307-343646(+) (14) TGGGGCTGTAGCCGGTCCCAAGGGCATGGCTGTTCGCCATTTAAAGTGGTACGTGAGCTGGGTTTAGAACGTCGTGAGACAGTTCGGTCCCTATCTGCCGTGGGCGTTGGAGATTTGAGAGGAGCTGCTCCTAGTACGAGAGGAC--CGG 2677

CP018005.1:167566-171940(+) (14) TGGGGCTGTAGCCGGTCCCAAGGGTATGGCTGTTCGCCATTTAAAGTGGTACGTGAGCTGGGTTTAGAACGTCGTGAGACAGTTCGGTCCCTATCTGCCGTGGGCGTTGGAGATTTGAGAGGAGCTGCTCCTAGTACGAGAGGAC--CGG 2687

NZ_CCAM010000005.1:71418-75764(+) (13) TGGGGCTGTAGCCGGTCCCAAGGGTATGGCTGTTCGCCATTTAAAGTGGTACGTGAGCTGGGTTTAGAACGTCGTGAGACAGTTCGGTCCCTATCTGCCGTGGGCGTTGGAGATTTGAGAGGAGCTGCTCCTAGTACGAGAGGAC--CGG 2677

NZ_CCXO01000001.1:317226-321572(+) (11) TGGGGCTGTAGCCGGTCCCAAGGGCATGGCTGTTCGCCATTTAAAGTGGTACGTGAGCTGGGTTTAGAACGTCGTGAGACAGTTCGGTCCCTATCTGCCGTGGGCGTTGGAGATTTGAGAGGAGCTGCTCCTAGTACGAGAGGAC--CGG 2677

NC_011527.1:1751498-1755844(-) (8) TGGGGCTGTAGCCGGTCCCAAGGGTATGGCTGTTCGCCATTTAAAGTGGTACGTGAGCTGGGTTTAGAACGTCGTGAGACAGTTCGGTCCCTATCTGCCGTGGGCGTTGGAGATTTGAGAGGAGCTGCTCCTAGTACGAGAGGAC--CGG 2677

NZ_CP032542.1:88036-92382(-) (8) TGGGGCTGTAGCCGGTCCCAAGGGTATGGCTGTTCGCCATTTAAAGTGGTACGTGAGCTGGGTTTAGAACGTCGTGAGACAGTTCGGTCCCTATCTGCCGTGGGCGTTGGAGATTTGAGAGGAGCTGCTCCTAGTACGAGAGGAC--CGG 2677

CP014551.1:161236-165610(+) (8) TGGGGCTGTAGCCGGTCCCAAGGGTATGGCTGTTCGCCATTTAAAGTGGTACGTGAGCTGGGTTTAGAACGTCGTGAGACAGTTCGGTCCCTATCTGCCGTGGGCGTTGGAGATTTGAGAGGAGCTGCTCCTAGTACGAGAGGAC--CGG 2687

CP013667.1:88011-92385(-) (8) TGGGGCTGTAGCCGGTCCCAAGGGTATGGCTGTTCGCCATTTAAAGTGGTACGTGAGCTGGGTTTAGAACGTCGTGAGACAGTTCGGTCCCTATCTGCCGTGGGCGTTGGAGATTTGAGAGGAGCTGCTCCTAGTACGAGAGGAC--CGG 2687

CP107247.1:164350-168689(+) (7) TGGGGCTGTAGCCGGTCCCAAGGGCATGGCTGTTCGCCATTTAAAGTGGTACGTGAGCTGGGTTTAGAACGTCGTGAGACAGTTCGGTCCCTATCTGCCGTGGGCGTTGGAGATTTGAGAGGAGCTGCTCCTAGTACGAGAGGAC--CGG 2677

NZ_CP103435.1:167581-171920(+) (6) TGGGGCTGTAGCCGGTCCCAAGGGTATGGCTGTTCGCCATTTAAAGTGGTACGTGAGCTGGGTTTAGAACGTCGTGAGACAGTTCGGTCCCTATCTGCCGTGGGCGTTGGAGATTTGAGAGGAGCTGCTCCTAGTACGAGAGGAC--CGG 2677

CP014565.1:1711747-1716121(-) (3) TGGGGCTGTAGCCGGTCCCAAGGGTATGGCTGTTCGCCATTTAAAGTGGTACGTGAGCTGGGTTTAGAACGTCGTGAGACAGTTCGGTCCCTATCTGCCGTGGGCGTTGGAGATTTGAGAGGAGCTGCTCCTAGTACGAGAGGAC--CGG 2687

AAYJ01000007.1:0-1404(+) (3) TGGGGCTGTAGCCGGTCCCAAGGGCATGGCTGTTCGCCATTTAAAGTGGTACGTGAGCTGGGTTTAGAACGTCGTGAGACAGTTCGGTCCCTATCTGCCGTGGGCGTTGGAGATTTGAGAGGAGCTGCTCCTAGTACGAGAGGAC--CGG 716

NZ_JAOXDR010000002.1:16672-21025(-) (3) TGGGGCTGTAGCCGGTCCCAAGGGTATGGCTGTTCGCCATTTAAAGTGGTACGTGAGCTGGGTTTAGAACGTCGTGAGACAGTTCGGTCCCTATCTGCCGTGGGCGTTGGAGATTTGAGAGGAGCTGCTCCTAGTACGAGAGGAC--CGG 2677

NZ_CP103432.1:167699-172038(+) (3) TGGGGCTGTAGCCGGTCCCAAGGGTATGGCTGTTCGCCATTTAAAGTGGTACGTGAGCTGGGTTTAGAACGTCGTGAGACAGTTCGGTCCCTATCTGCCGTGGGCGTTGGAGATTTGAGAGGAGCTGCTCCTAGTACGAGAGGAC--CGG 2677

NZ_CP103431.1:167697-172036(+) (3) TGGGGCTGTAGCCGGTCCCAAGGGCATGGCTGTTCGCCATTTAAAGTGGTACGTGAGCTGGGTTTAGAACGTCGTGAGACAGTTCGGTCCCTATCTGCCGTGGGCGTTGGAGATTTGAGAGGAGCTGCTCCTAGTACGAGAGGAC--CGG 2677

NZ_CP103428.1:167591-171929(+) (2) TGGGGCTGTAGCCGGTCCCAAGGGTATGGCTGTTCGCCATTTAAAGTGGTACGTGAGCTGGGTTTAGAACGTCGTGAGACAGTTCGGTCCCTATCTGCCGTGGGCGTTGGAGATTTGAGAGGAGCTGCTCCTAGTACGAGAGGAC--CGG 2677

NZ_JANTNR010000002.1:115350-119696(+) (2) TGGGGCTGTAGCCGGTCCCAAGGGTATGGCTGTTCGCCATTTAAAGTGGTACGTGAGCTGGGTTTAGAACGTCGTGAGACAGTTCGGTCCCTATCTGCCGTGGGCGTTGGAGATTTGAGAGGAGCTGCTCCTAGTACGAGAGGAC--CGG 2677

JARBIR010004564.1:3782-8128(-) (2) TGGGGCTGTAGCCGGTCCCAAGGGTATGGCTGTTCGCCATTTAAAGTGGTACGTGAGCTGGGTTTAGAACGTCGTGAGACAGTTCGGTCCCTATCTGCCGTGGGCGTTGGAGATTTGAGAGGAGCTGCTCCTAGTACGAGAGGAC--CGG 2677

NC_009727.1:1888349-1892695(-) (2) TGGGGCTGTAGCCGGTCCCAAGGGTATGGCTGTTCGCCATTTAAAGTGGTACGTGAGCTGGGTTTAGAACGTCGTGAGACAGTTCGGTCCCTATCTGCCGTGGGCGTTGGAGATTTGAGAGGAGCTGCTCCTAGTACGAGAGGAC--CGG 2677

AAYJ01000139.1:0-660(+) (2) ------------------------------------------------------------------------------------------------------------------------------------------------------ 1509

CP032542.1:88018-92392(-) (2) TGGGGCTGTAGCCGGTCCCAAGGGTATGGCTGTTCGCCATTTAAAGTGGTACGTGAGCTGGGTTTAGAACGTCGTGAGACAGTTCGGTCCCTATCTGCCGTGGGCGTTGGAGATTTGAGAGGAGCTGCTCCTAGTACGAGAGGAC--CGG 2687

CP014559.1:232744-237117(+) (2) TGGGGCTGTAGCCGGTCCCAAGGGTATGGCTGTTCGCCATTTAAAGTGGTACGTGAGCTGGGTTTAGAACGTCGTGAGACAGTTCGGTCCCTATCTGCCGTGGGCGTTGGAGATTTGAGAGGAGCTGCTCCTAGTACGAGAGGAC--CGG 2687

CP007555.1:337408-341752(+) TGGGGCTGTAGCCGGTCCCAAGGGCATGGCTGTTCGCCATTTAAAGTGGTACGTGAGCTGGGTTTAGAACGTCGTGAGACAGTTCGGTCCCTATCTGCCGTGGGCGTTGGAGATTTGAGAGGAGCTGCTCCTAGTACGAGAGGAC--CGG 2675

NZ_CP014354.1:76710-81178(-) TGGGGCTGTAGCCGGTCCCAAGGGTATGGCTGTTCGCCATTTAAAGTGGTACGTGAGCTGGGTTTAGAACGTCGTGAGANNNNTCGGTCCCTATCTGCCGTGGGCGTTGGAGATTTGAGAGGAGCTGCTCCTAGTACGAGAGGAC--CGG 2687

CP000890.1:243129-244433(+) ------------------------------------------------------------------------------------------------------------------------------------------------------ 1509

NZ_CP115461.1:167576-171922(+) TGGGGCTGAAGTAGGTCCCAAGGGTATGGCTGTTCGCCATTTAAAGTGGTACGCGAGCTGGGTTTAGAACGTCGTGAGACAGTTCGGTCCCTATCTGTCGTGGGCGAAGGAAGTTTGAGAGGAGCTGCTCCTAGTACGAGAGGAC--CGG 2677

AP019759.1:167578-171919(+) TGGGGCTGTAGCCGGTCCCAAGGGTATGGCTGTTCGCCATTTAAAGTGGTACGTGAGCTGGGTTTAGAACGTCGTGAGACAGTTCGGTCCCTATCTGCCGTGGGCGTTGGAGATTTGAGAGGAGCTGCTCCTAGTACGAGAGGAC--CGG 2675

AP019757.1:1739267-1743608(-) TGGGGCTGTAGCCGGTCCCAAGGGTATGGCTGTTCGCCATTTAAAGTGGTACGTGAGCTGGGTTTAGAACGTCGTGAGACAGTTCGGTCCCTATCTGCCGTGGGCGTTGGAGATTTGAGAGGAGCTGCTCCTAGTACGAGAGGAC--CGG 2675

NOLN01000021.1:90861-95235(-) TGGGGCTGTAGCCGGTCCCAAGGGTATGGCTGTTCGCCATTTAAAGTGGTACGTGAGCTGGGTTTAGAACGTCGTGAGACAGTTCGGTCCCTATCTGCCGTGGGCGTTGGAGATTTGAGAGGAGCTGCTCCTAGTACGAGAGGAC--CGG 2687

NOLM01000022.1:91202-95576(-) TGGGGCTGTAGCCGGTCCCAAGGGTATGGCTGTTCGCCATTTAAAGTGGTACGTGAGCTGGGTTTAGAACGTCGTGAGACAGTTCGGTCCCTATCTGCCGTGGGCGTTGGAGATTTGAGAGGAGCTGCTCCTAGTACGAGAGGAC--CGG 2687

PDLP01000011.1:14431-18805(+) TGGGGCTGTAGCCGGTCCCAAGGGCATGGCTGTTCGCCATTTAAAGTGGTACGTGAGCTGGGTTTAGAACGTCGTGAGACAGTTCGGTCCCTATCTGCCGTGGGCGTTGGAGATTTGAGAGGAGCTGCTCCTAGTACGAGAGGAC--CGG 2687

CP014563.1:522940-527314(+) TGGGGCTGTAGCCGGTCCCAAGGGCATGGCTGTTCGCCATTTAAAGTGGTACGTGAGCTGGGTTTAGAACGTCGTGAGACAGTTCGGTCCCTATCTGCCGTGGGCGTTGGAGATTTGAGAGGAGCTGCTCCTAGTACGAGAGGAC--CGG 2687

CP000890.1:244922-245905(+) TGGGGCTGTAGCCGGTCCCAAGGGTATGGCTGTTCGCCATTTAAAGTGGTACGTGAGCTGGGTTTAGAACGTCGTGAGACAGTTCGGTCCCTATCTGCCGTGGGCGTTGGAGATTTGAGAGGAGCTGCTCCTAGTACGAGAGGAC--CGG 716

NZ_NOLR01000002.1:131843-136189(+) TGGGGCTGTAGCCGGTCCCAAGGGTATGGCTGTTCGCCATTTAAAGTGGTACGTGAGCTGGGTTTAGAACGTCGTGAGACAGTTCGGTCCCTATCTGCCGTGGGCGTTGGAGATTTGAGAGGAGCTGCTCCTAGTACGAGAGGAC--CGG 2677

NZ_JAOXDP010000002.1:15794-20139(-) TGGGGCTGTAGCCGGTCCCAAGGGTATGGCTGTTCGCCATTTAAAGTGGTACGTGAGCTGGGTTTAGAACGTCGTGAGACAGTTCGGTCCCTATCTGCCGTGGGCGTTGGAGATTTGAGAGGAGCTGCTCCTAGTACGAGAGGAC--CGG 2677

NZ_PPFR01000008.1:34378-38724(-) TGGGGCTGTAGCCGGTCCCAAGGGTATGGCTGTTCGCCATTTAAAGTGGTACGTGAGCTGGGTTTAGAACGTCGTGAGACAGTTCGGTCCCTATCTGCCGTGGGCGTTGGAGATTTGAGAGGAGCTGCTCCTAGTACGAGAGGAC--CGG 2677

NZ_PPFQ01000003.1:131093-135446(+) TGGGGCTGTAGCCGGTCCCAAGGGTATGGCTGTTCGCCATTTAAAGTGGTACGTGAGCTGGGTTTAGAACGTCGTGAGACAGTTCGGTCCCTATCTGCCGTGGGCGTTGGAGATTTGAGAGGAGCTGCTCCTAGTACGAGAGGAC--CGG 2677

NZ_LK937696.1:167613-171953(+) TGGGGCTGTAGCCGGTCCCAAGGGTATGGCTGTTCGCCATTTAAAGTGGTACGTGAGCTGGGTTTAGAACGTCGTGAGACAGTTCGGTCCCTATCTGCCGTGGGCGTTGGAGATTTGAGAGGAGCTGCTCCTAGTACGAGAGGAC--CGG 2677

NZ_NOVI01000003.1:105965-110310(+) TGGGGCTGTAGCCGGTCCCAAGGGTATGGCTGTTCGCCATTTAAAGTGGTACGTGAGCTGGGTTTAGAACGTCGTGAGACAGTTCGGTCCCTATCTGCCGTGGGCGTTGGAGATTTGAGAGGAGCTGCTCCTAGTACGAGAGGAC--CGG 2677

NZ_CP103426.1:167642-171981(+) TGGGGCTGTAGCCGGTCCCAAGGGTATGGCTGTTCGCCATTTAAAGTGGTACGTGAGCTGGGTTTAGAACGTCGTGAGACAGTTCGGTCCCTATCTGCCGTGGGCGTTGGAGATTTGAGAGGAGCTGCTCCTAGTACGAGAGGAC--CGG 2677

NZ_JAOXDN010000007.1:36985-41324(-) TGGGGCTGTAGCCGGTCCCAAGGGTATGGCTGTTCGCCATTTAAAGTGGTACGTGAGCTGGGTTTAGAACGTCGTGAGACAGTTCGGTCCCTATCTGCCGTGGGCGTTGGAGATTTGAGAGGAGCTGCTCCTAGTACGAGAGGAC--CGG 2677

NZ_JPVV01000013.1:13888-18051(+) TGGGGCTGTAGCCGGTCCCAAGGGCATGGCTGTTCGCCATTTAAAGTGGTACGTGAGCTGGGTTTAGAACGTCGTGAGACAGTTCGGTCCCTATCTGCCGTGGGCGTTGGAGATTTGAGAGGAGCTGCTCCTAGTACGAGAGGAC--CGG 2677

NZ_CP007555.1:337406-341752(+) TGGGGCTGTAGCCGGTCCCAAGGGCATGGCTGTTCGCCATTTAAAGTGGTACGTGAGCTGGGTTTAGAACGTCGTGAGACAGTTCGGTCCCTATCTGCCGTGGGCGTTGGAGATTTGAGAGGAGCTGCTCCTAGTACGAGAGGAC--CGG 2677

NZ_JASNNV010000010.1:39284-43631(-) TGGGGCTGTAGCCGGTCCCAAGGGCATGGCTGTTCGCCATTTAAAGTGGTACGTGAGCTGGGTTTAGAACGTCGTGAGACAGTTCGGTCCCTATCTGCCGTGGGCGTTGGAGATTTGAGAGGAGCTGCTCCTAGTACGAGAGGATTGCGG 2678

NZ_JAOXFC010000012.1:14231-18577(+) TGGGGCTGTAGCCGGTCCCAAGGGCATGGCTGTTCGCCATTTAAAGTGGTACGTGAGCTGGGTTTAGAACGTCGTGAGACAGTTCGGTCCCTATCTGCCGTGGGCGTTGGAGATTTGAGAGGAGCTGCTCCTAGTACGAGAGGAC--CGG 2677

NZ_CP103430.1:167658-171997(+) TGGGGCTGTAGCCGGTCCCAAGGGCATGGCTGTTCGCCATTTAAAGTGGTACGTGAGCTGGGTTTAGAACGTCGTGAGACAGTTCGGTCCCTATCTGCCGTGGGCGTTGGAGATTTGAGAGGAGCTGCTCCTAGTACGAGAGGAC--CGG 2677

NZ_PDLP01000011.1:14441-18787(+) TGGGGCTGTAGCCGGTCCCAAGGGCATGGCTGTTCGCCATTTAAAGTGGTACGTGAGCTGGGTTTAGAACGTCGTGAGACAGTTCGGTCCCTATCTGCCGTGGGCGTTGGAGATTTGAGAGGAGCTGCTCCTAGTACGAGAGGAC--CGG 2677

NZ_NOLM01000022.1:91220-95566(-) TGGGGCTGTAGCCGGTCCCAAGGGTATGGCTGTTCGCCATTTAAAGTGGTACGTGAGCTGGGTTTAGAACGTCGTGAGACAGTTCGGTCCCTATCTGCCGTGGGCGTTGGAGATTTGAGAGGAGCTGCTCCTAGTACGAGAGGAC--CGG 2677

JBJCIS010000056.1:0-2780(-) ------------------------------------------------------------------------------------------------------------------------------------------------------ 2683

NZ_CDBG01000001.1:179744-184090(+) TGGGGCTGTAGCCGGTCCCAAGGGCATGGCTGTTCGCCATTTAAAGTGGTACGTGAGCTGGGTTTAGAACGTCGTGAGACAGTTCGGTCCCTATCTGCCGTGGGCGTTGGAGATTTGAGAGGAGCTGCTCCTAGTACGAGAGGAC--CGG 2677

NZ_CP103434.1:167622-171961(+) TGGGGCTGTAGCCGGTCCCAAGGGTATGGCTGTTCGCCATTTAAAGTGGTACGTGAGCTGGGTTTAGAACGTCGTGAGACAGTTCGGTCCCTATCTGCCGTGGGCGTTGGAGATTTGAGAGGAGCTGCTCCTAGTACGAGAGGAC--CGG 2677

CP014354.1:76692-80603(-) TGGGGCTGTAGCCGGTCCCAAGGGTATGGCTGTTCGCCATTTAAAGTGGTACGTGAGCTGGGTTTAGAACGTCGTGAGANNNNTCGGTCCCTATCTGCCGTGGGCGTTGGAGATTTGAGAGGAGCTGCTCCTAGTACGAGAGGAC--CGG 2212

NOLR01000002.1:131833-136207(+) TGGGGCTGTAGCCGGTCCCAAGGGTATGGCTGTTCGCCATTTAAAGTGGTACGTGAGCTGGGTTTAGAACGTCGTGAGACAGTTCGGTCCCTATCTGCCGTGGGCGTTGGAGATTTGAGAGGAGCTGCTCCTAGTACGAGAGGAC--CGG 2687

PPFR01000008.1:34360-38734(-) TGGGGCTGTAGCCGGTCCCAAGGGTATGGCTGTTCGCCATTTAAAGTGGTACGTGAGCTGGGTTTAGAACGTCGTGAGACAGTTCGGTCCCTATCTGCCGTGGGCGTTGGAGATTTGAGAGGAGCTGCTCCTAGTACGAGAGGAC--CGG 2687

PPFQ01000003.1:131083-135464(+) TGGGGCTGTAGCCGGTCCCAAGGGTATGGCTGTTCGCCATTTAAAGTGGTACGTGAGCTGGGTTTAGAACGTCGTGAGACAGTTCGGTCCCTATCTGCCGTGGGCGTTGGAGATTTGAGAGGAGCTGCTCCTAGTACGAGAGGAC--CGG 2687

NZ_AKYP01000181.1:1647-6020(-) TGGGGCTGTAGCCGGTCCCAAGGGTATGGCTGTTCGCCATTTAAAGTGGTACGTGAGCTGGGTTTAGAACGTCGTGAGACAGTTCGGTCCCTATCTGCCGTGGGCGTTGGAGATTTGAGAGGAGCTGCTCCTAGTACGAGAGGAC--CGG 2686

NOVI01000003.1:105955-110328(+) TGGGGCTGTAGCCGGTCCCAAGGGTATGGCTGTTCGCCATTTAAAGTGGTACGTGAGCTGGGTTTAGAACGTCGTGAGACAGTTCGGTCCCTATCTGCCGTGGGCGTTGGAGATTTGAGAGGAGCTGCTCCTAGTACGAGAGGAC--CGG 2687

CP018150.1:339294-343661(+) TGGGGCTGTAGCCGGTCCCAAGGGCATGGCTGTTCGCCATTTAAAGTGGTACGTGAGCTGGGTTTAGAACGTCGTGAGACAGTTCGGTCCCTATCTGCCGTGGGCGTTGGAGATTTGAGAGGAGCTGCTCCTAGTACGAGAGGAC--CGG 2687

Consensus AGTGGACGTACCTCTGGTGTTCCGGTTGTCACGCCAGTGGCATTGCCGGGTAGCTAAGTACGGACGGGATAACCGCTGAAAGCATCTAAGCGGGAAGCCCCCCTCAAGATGAGATCTCCCGGACCTTTAAGGTCCCTAAAGATTCGTTGA 2837

JAKFBC010000002.1:34496-38842(-) (45) AGTGGACGTACCTCTGGTGTTCCGGTTGTCACGCCAGTGGCATTGCCGGGTAGCTAAGTACGGACGGGATAACCGCTGAAAGCATCTAAGCGGGAAGCCCCCCTCAAGATGAGATCTCCCGGACCTTTAAGGTCCCTAAAGATTCGTTGA 2827

NZ_AP019757.1:1739264-1743610(-) (31) AGTGGACGTACCTCTGGTGTTCCGGTTGTCACGCCAGTGGCATTGCCGGGTAGCTAAGTACGGACGGGATAACCGCTGAAAGCATCTAAGCGGGAAGCCCCCCTCAAGATGAGATCTCCCGGACCTTTAAGGTCCCTAAAGATTCGTTGA 2827

JBKOHN010000001.1:81530-85875(+) (25) AGTGGACGTACCTCTGGTGTTCCGGTTGTCACGCCAGTGGCATTGCCGGGTAGCTAAGTACGGACGGGATAACCGCTGAAAGCATCTAAGCGGGAAGCCCCCCTCAAGATGAGATCTCCCGGACCTTTAAGGTCCCTAAAGATTCGTTGA 2827

NC_011528.1:339307-343646(+) (14) AGTGGACGTACCTCTGGTGTTCCGGTTGTCACGCCAGTGGCATTGCCGGGTAGCTAAGTACGGACGGGATAACCGCTGAAAGCATCTAAGCGGGAAGCCCCCCTCAAGATGAGATCTCCCGGACCTTTAAGGTCCCTAAAGATTCGTTGA 2827

CP018005.1:167566-171940(+) (14) AGTGGACGTACCTCTGGTGTTCCGGTTGTCACGCCAGTGGCATTGCCGGGTAGCTAAGTACGGACGGGATAACCGCTGAAAGCATCTAAGCGGGAAGCCCCCCTCAAGATGAGATCTCCCGGACCTTTAAGGTCCCTAAAGATTCGTTGA 2837

NZ_CCAM010000005.1:71418-75764(+) (13) AGTGGACGTACCTCTGGTGTTCCGGTTGTCACGCCAGTGGCATTGCCGGGTAGCTAAGTACGGACGGGATAACCGCTGAAAGCATCTAAGCGGGAAGCCCCCCTCAAGATGAGATCTCCCGGACCTTTAAGGTCCCTAAAGATTCGTTGA 2827

NZ_CCXO01000001.1:317226-321572(+) (11) AGTGGACGTACCTCTGGTGTTCCGGTTGTCACGCCAGTGGCATTGCCGGGTAGCTAAGTACGGACGGGATAACCGCTGAAAGCATCTAAGCGGGAAGCCCCCCTCAAGATGAGATCTCCCGGACCTTTAAGGTCCCTAAAGATTCGTTGA 2827

NC_011527.1:1751498-1755844(-) (8) AGTGGACGTACCTCTGGTGTTCCGGTTGTCACGCCAGTGGCATTGCCGGGTAGCTAAGTACGGACGGGATAACCGCTGAAAGCATCTAAGCGGGAAGCCCCCCTCAAGATGAGATCTCCCGGACCTTTAAGGTCCCTAAAGATTCGTTGA 2827

NZ_CP032542.1:88036-92382(-) (8) AGTGGACGTACCTCTGGTGTTCCGGTTGTCACGCCAGTGGCATTGCCGGGTAGCTAAGTACGGACGGGATAACCGCTGAAAGCATCTAAGCGGGAAGCCCCCCTCAAGATGAGATCTCCCGGACCTTTAAGGTCCCTAAAGATTCGTTGA 2827

CP014551.1:161236-165610(+) (8) AGTGGACGTACCTCTGGTGTTCCGGTTGTCACGCCAGTGGCATTGCCGGGTAGCTAAGTACGGACGGGATAACCGCTGAAAGCATCTAAGCGGGAAGCCCCCCTCAAGATGAGATCTCCCGGACCTTTAAGGTCCCTAAAGATTCGTTGA 2837

CP013667.1:88011-92385(-) (8) AGTGGACGTACCTCTGGTGTTCCGGTTGTCACGCCAGTGGCATTGCCGGGTAGCTAAGTACGGACGGGATAACCGCTGAAAGCATCTAAGCGGGAAGCCCCCCTCAAGATGAGATCTCCCGGACCTTTAAGGTCCCTAAAGATTCGTTGA 2837

CP107247.1:164350-168689(+) (7) AGTGGACGTACCTCTGGTGTTCCGGTTGTCACGCCAGTGGCATTGCCGGGTAGCTAAGTACGGACGGGATAACCGCTGAAAGCATCTAAGCGGGAAGCCCCCCTCAAGATGAGATCTCCCGGACCTTTAAGGTCCCTAAAGATTCGTTGA 2827

NZ_CP103435.1:167581-171920(+) (6) AGTGGACGTACCTCTGGTGTTCCGGTTGTCACGCCAGTGGCATTGCCGGGTAGCTAAGTACGGACGGGATAACCGCTGAAAGCATCTAAGCGGGAAGCCCCCCTCAAGATGAGATCTCCCGGACCTTTAAGGTCCCTAAAGATTCGTTGA 2827

CP014565.1:1711747-1716121(-) (3) AGTGGACGTACCTCTGGTGTTCCGGTTGTCACGCCAGTGGCATTGCCGGGTAGCTAAGTACGGACGGGATAACCGCTGAAAGCATCTAAGCGGGAAGCCCCCCTCAAGATGAGATCTCCCGGACCTTTAAGGTCCCTAAAGATTCGTTGA 2837

AAYJ01000007.1:0-1404(+) (3) AGTGGACGTACCTCTGGTGTTCCGGTTGTCACGCCAGTGGCATTGCCGGGTAGCTAAGTACGGACGGGATAACCGCTGAAAGCATCTAAGCGGGAAGCCCCCCTCAAGATGAGATCTCCCGGACCTTTAAGGTCCCTAAAGATTCGTTGA 866

NZ_JAOXDR010000002.1:16672-21025(-) (3) AGTGGACGTACCTCTGGTGTTCCGGTTGTCACGCCAGTGGCATTGCCGGGTAGCTAAGTACGGACGGGATAACCGCTGAAAGCATCTAAGCGGGAAGCCCCCCTCAAGATGAGATCTCCCGGACCTTTAAGGTCCCTAAAGATTCGTTGA 2827

NZ_CP103432.1:167699-172038(+) (3) AGTGGACGTACCTCTGGTGTTCCGGTTGTCACGCCAGTGGCATTGCCGGGTAGCTAAGTACGGACGGGATAACCGCTGAAAGCATCTAAGCGGGAAGCCCCCCTCAAGATGAGATCTCCCGGACCTTTAAGGTCCCTAAAGATTCGTTGA 2827

NZ_CP103431.1:167697-172036(+) (3) AGTGGACGTACCTCTGGTGTTCCGGTTGTCACGCCAGTGGCATTGCCGGGTAGCTAAGTACGGACGGGATAACCGCTGAAAGCATCTAAGTGGGAAGCCCCCCTCAAGATGAGATCTCCCGGACCTTTAAGGTCCCTAAAGATTCGTTGA 2827

NZ_CP103428.1:167591-171929(+) (2) AGTGGACGTACCTCTGGTGTTCCGGTTGTCACGCCAGTGGCATTGCCGGGTAGCTAAGTACGGACGGGATAACCGCTGAAAGCATCTAAGCGGGAAGCCCCCCTCAAGATGAGATCTCCCGGACCTTTAAGGTCCCTAAAGATTCGTTGA 2827

NZ_JANTNR010000002.1:115350-119696(+) (2) AGTGGACGTACCTCTGGTGTTCCGGTTGTCACGCCAGTGGCATTGCCGGGTAGCTAAGTACGGACGGGATAACCGCTGAAAGCATCTAAGCGGGAAGCCCCCCTCAAGATGAGATCTCCCGGACCTTTAAGGTCCCTAAAGATTCGTTGA 2827

JARBIR010004564.1:3782-8128(-) (2) AGTGGACGTACCTCTGGTGTTCCGGTTGTCACGCCAGTGGCATTGCCGGGTAGCTAAGTACGGACGGGATAACCGCTGAAAGCATCTAAGCGGGAAGCCCCCCTCAAGATGAGATCTCCCGGACCTTTAAGGTCCCTAAAGATTCGTTGA 2827

NC_009727.1:1888349-1892695(-) (2) AGTGGACGTACCTCTGGTGTTCCGGTTGTCACGCCAGTGGCATTGCCGGGTAGCTAAGTACGGACGGGATAACCGCTGAAAGCATCTAAGCGGGAAGCCCCCCTCAAGATGAGATCTCCCGGACCTTTAAGGTCCCTAAAGATTCGTTGA 2827

AAYJ01000139.1:0-660(+) (2) ------------------------------------------------------------------------------------------------------------------------------------------------------ 1659

CP032542.1:88018-92392(-) (2) AGTGGACGTACCTCTGGTGTTCCGGTTGTCACGCCAGTGGCATTGCCGGGTAGCTAAGTACGGACGGGATAACCGCTGAAAGCATCTAAGCGGGAAGCCCCCCTCAAGATGAGATCTCCCGGACCTTTAAGGTCCCTAAAGATTCGTTGA 2837

CP014559.1:232744-237117(+) (2) AGTGGACGTACCTCTGGTGTTCCGGTTGTCACGCCAGTGGCATTGCCGGGTAGCTAAGTACGGACGGGATAACCGCTGAAAGCATCTAAGCGGGAAGCCCCCCTCAAGATGAGATCTCCCGGACCTTTAAGGTCCCTAAAGATTCGTTGA 2837

CP007555.1:337408-341752(+) AGTGGACGTACCTCTGGTGTTCCGGTTGTCACGCCAGTGGCATTGCCGGGTAGCTAAGTACGGACGGGATAACCGCTGAAAGCATCTAAGCGGGAAGCCCCCCTCAAGATGAGATCTCCCGGACCTTTAAGGTCCCTAAAGATTCGTTGA 2825

NZ_CP014354.1:76710-81178(-) AGTGGACGTACCTCTGGTGTTCCGGTTGTCACGCCAGTGGCATTGCCGGGTAGCTAAGTACGGACGGGATAACCGCTGAAAGCATCTAAGCGGGAAGCCCCCCTCAAGATGAGATCTCCCGGACCTTTAAGGTCCCTAAAGATTCGTTGA 2837

CP000890.1:243129-244433(+) ------------------------------------------------------------------------------------------------------------------------------------------------------ 1659

NZ_CP115461.1:167576-171922(+) AGTGGACGTACCTCTGGTGTTCCGGTTGTCACGCCAGTGGCATTGCCGGGTAGCTAAGTACGGACGGGATAACCGCTGAAAGCATCTAAGCGGGAAGCCCCCCTCAAGATGAGATCTCCCGGACCTTTAAGGTCCCTAAAGATTCGTTGA 2827

AP019759.1:167578-171919(+) AGTGGACGTACCTCTGGTGTTCCGGTTGTCACGCCAGTGGCATTGCCGGGTAGCTAAGTACGGACGGGATAACCGCTGAAAGCATCTAAGCGGGAAGCCCCCCTCAAGATGAGATCTCCCGGACCTTTAAGGTCCCTAAAGATTCGTTGA 2825

AP019757.1:1739267-1743608(-) AGTGGACGTACCTCTGGTGTTCCGGTTGTCACGCCAGTGGCATTGCCGGGTAGCTAAGTACGGACGGGATAACCGCTGAAAGCATCTAAGCGGGAAGCCCCCCTCAAGATGAGATCTCCCGGACCTTTAAGGTCCCTAAAGATTCGTTGA 2825

NOLN01000021.1:90861-95235(-) AGTGGACGTACCTCTGGTGTTCCGGTTGTCACGCCAGTGGCATTGCCGGGTAGCTAAGTACGGACGGGATAACCGCTGAAAGCATCTAAGCGGGAAGCCCCCCTCAAGATGAGATCTCCCGGACCTTTAAGGTCCCTAAAGATTCGTTGA 2837

NOLM01000022.1:91202-95576(-) AGTGGACGTACCTCTGGTGTTCCGGTTGTCACGCCAGTGGCATTGCCGGGTAGCTAAGTACGGACGGGATAACCGCTGAAAGCATCTAAGCGGGAAGCCCCCCTCAAGATGAGATCTCCCGGACCTTTAAGGTCCCTAAAGATTCGTTGA 2837

PDLP01000011.1:14431-18805(+) AGTGGACGTACCTCTGGTGTTCCGGTTGTCACGCCAGTGGCATTGCCGGGTAGCTAAGTACGGACGGGATAACCGCTGAAAGCATCTAAGCGGGAAGCCCCCCTCAAGATGAGATCTCCCGGACCTTTAAGGTCCCTAAAGATTCGTTGA 2837

CP014563.1:522940-527314(+) AGTGGACGTACCTCTGGTGTTCCGGTTGTCACGCCAGTGGCATTGCCGGGTAGCTAAGTACGGACGGGATAACCGCTGAAAGCATCTAAGCGGGAAGCCCCCCTCAAGATGAGATCTCCCGGACCTTTAAGGTCCCTAAAGATTCGTTGA 2837

CP000890.1:244922-245905(+) AGTGGACGTACCTCTGGTGTTCCGGTTGTCACGCCAGTGGCATTGCCGGGTAGCTAAGTACGGACGGGATAACCGCTGAAAGCATCTAAGCGGGAAGCCCCCCTCAAGATGAGATCTCCCGGACCTTTAAGGTCCCTAAAGATTCGTTGA 866

NZ_NOLR01000002.1:131843-136189(+) AGTGGACGTACCTCTGGTGTTCCGGTTGTCACGCCAGTGGCATTGCCGGGTAGCTAAGTACGGACGGGATAACCGCTGAAAGCATCTAAGCGGGAAGCCCCCCTCAAGATGAGATCTCCCGGACCTTTAAGGTCCCTAAAGATTCGTTGA 2827

NZ_JAOXDP010000002.1:15794-20139(-) AGTGGACGTACCTCTGGTGTTCCGGTTGTCACGCCAGTGGCATTGCCGGGTAGCTAAGTACGGACGGGATAACCGCTGAAAGCATCTAAGCGGGAAGCCCCCCTCAAGATGAGATCTCCCGGACCTTTAAGGTCCCTAAAGATTCGTTGA 2827

NZ_PPFR01000008.1:34378-38724(-) AGTGGACGTACCTCTGGTGTTCCGGTTGTCACGCCAGTGGCATTGCCGGGTAGCTAAGTACGGACGGGATAACCGCTGAAAGCATCTAAGCGGGAAGCCCCCCTCAAGATGAGATCTCCCGGACCTTTAAGGTCCCTAAAGATTCGTTGA 2827

NZ_PPFQ01000003.1:131093-135446(+) AGTGGACGTACCTCTGGTGTTCCGGTTGTCACGCCAGTGGCATTGCCGGGTAGCTAAGTACGGACGGGATAACCGCTGAAAGCATCTAAGCGGGAAGCCCCCCTCAAGATGAGATCTCCCGGACCTTTAAGGTCCCTAAAGATTCGTTGA 2827

NZ_LK937696.1:167613-171953(+) AGTGGACGTACCTCTGGTGTTCCGGTTGTCACGCCAGTGGCATTGCCGGGTAGCTAAGTACGGACGGGATAACCGCTGAAAGCATCTAAGCGGGAAGCCCCCCTCAAGATGAGATCTCCCGGACCTTTAAGGTCCCTAAAGATTCGTTGA 2827

NZ_NOVI01000003.1:105965-110310(+) AGTGGACGTACCTCTGGTGTTCCGGTTGTCACGCCAGTGGCATTGCCGGGTAGCTAAGTACGGACGGGATAACCGCTGAAAGCATCTAAGCGGGAAGCCCCCCTCAAGATGAGATCTCCCGGACCTTTAAGGTCCCTAAAGATTCGTTGA 2827

NZ_CP103426.1:167642-171981(+) AGTGGACGTACCTCTGGTGTTCCGGTTGTCACGCCAGTGGCATTGCCGGGTAGCTAAGTACGGACGGGATAACCGCTGAAAGCATCTAAGCGGGAAGCCCCCCTCAAGATGAGATCTCCCGGACCTTTAAGGTCCCTAAAGATTCGTTGA 2827

NZ_JAOXDN010000007.1:36985-41324(-) AGTGGACGTACCTCTGGTGTTCCGGTTGTCACGCCAGTGGCATTGCCGGGTAGCTAAGTACGGACGGGATAACCGCTGAAAGCATCTAAGCGGGAAGCCCCCCTCAAGATGAGATCTCCCGGACCTTTAAGGTCCCTAAAGATTCGTTGA 2827

NZ_JPVV01000013.1:13888-18051(+) AGTGGACGTACCTCTGGTGTTCCGGTTGTCACGCCAGTGGCATTGCCGGGTAGCTAAGTACGGACGGGATAACCGCTGAAAGCATCTAAGCGGGAAGCCCCCCTCAAGATGAGATCTCCCGGACCTTTAAGGTCCCTAAAGATTCGTTGA 2827

NZ_CP007555.1:337406-341752(+) AGTGGACGTACCTCTGGTGTTCCGGTTGTCACGCCAGTGGCATTGCCGGGTAGCTAAGTACGGACGGGATAACCGCTGAAAGCATCTAAGCGGGAAGCCCCCCTCAAGATGAGATCTCCCGGACCTTTAAGGTCCCTAAAGATTCGTTGA 2827

NZ_JASNNV010000010.1:39284-43631(-) AGTGGACGTACCTCTGGTGTTCCGGTTGTCACGCCAGTGGCATTGCCGGGTAGCTAAGTACGGACGGGATAACCGCTGAAAGCATCTAAGCGGGAAGCCCCCCTCAAGATGAGATCTCCCGGACCTTTAAGGTCCCTAAAGATTCGTTGA 2828

NZ_JAOXFC010000012.1:14231-18577(+) AGTGGACGTACCTCTGGTGTTCCGGTTGTCACGCCAGTGGCATTGCCGGGTAGCTAAGTACGGACGGGATAACCGCTGAAAGCATCTAAGCGGGAAGCCCCCCTCAAGATGAGATCTCCCGGACCTTTAAGGTCCCTAAAGATTCGTTGA 2827

NZ_CP103430.1:167658-171997(+) AGTGGACGTACCTCTGGTGTTCCGGTTGTCACGCCAGTGGCATTGCCGGGTAGCTAAGTACGGACGGGATAACCGCTGAAAGCATCTAAGCGGGAAGCCCCCCTCAAGATGAGATCTCCCGGACCTTTAAGGTCCCTAAAGATTCGTTGA 2827

NZ_PDLP01000011.1:14441-18787(+) AGTGGACGTACCTCTGGTGTTCCGGTTGTCACGCCAGTGGCATTGCCGGGTAGCTAAGTACGGACGGGATAACCGCTGAAAGCATCTAAGCGGGAAGCCCCCCTCAAGATGAGATCTCCCGGACCTTTAAGGTCCCTAAAGATTCGTTGA 2827

NZ_NOLM01000022.1:91220-95566(-) AGTGGACGTACCTCTGGTGTTCCGGTTGTCACGCCAGTGGCATTGCCGGGTAGCTAAGTACGGACGGGATAACCGCTGAAAGCATCTAAGCGGGAAGCCCCCCTCAAGATGAGATCTCCCGGACCTTTAAGGTCCCTAAAGATTCGTTGA 2827

JBJCIS010000056.1:0-2780(-) ------------------------------------------------------------------------------------------------------------------------------------------------------ 2833

NZ_CDBG01000001.1:179744-184090(+) AGTGGACGTACCTCTGGTGTTCCGGTTGTCACGCCAGTGGCATTGCCGGGTAGCTAAGTACGGACGGGATAACCGCTGAAAGCATCTAAGCGGGAAGCCCCCCTCAAGATGAGATCTCCCGGACCTTTAAGGTCCCTAAAGATTCGTTGA 2827

NZ_CP103434.1:167622-171961(+) AGTGGACGTACCTCTGGTGTTCCGGTTGTCACGCCAGTGGCATTGCCGGGTAGCTAAGTACGGACGGGATAACCGCTGAAAGCATCTAAGCGGGAAGCCCCCCTCAAGATGAGATCTCCCGGACCTTTAAGGTCCCTAAAGATTCGTTGA 2827

CP014354.1:76692-80603(-) AGTGGACGTACCTCTGGTGTTCCGGTTGTCACGCCAGTGGCATTGCCGGGTAGCTAAGTACGGACGGGATAACCGCTGAAAGCATCTAAGCGGGAAGCCCCCCTCAAGATGAGATCTCCCGGACCTTTAAGGTCCCTAAAGATTCGTTGA 2362

NOLR01000002.1:131833-136207(+) AGTGGACGTACCTCTGGTGTTCCGGTTGTCACGCCAGTGGCATTGCCGGGTAGCTAAGTACGGACGGGATAACCGCTGAAAGCATCTAAGCGGGAAGCCCCCCTCAAGATGAGATCTCCCGGACCTTTAAGGTCCCTAAAGATTCGTTGA 2837

PPFR01000008.1:34360-38734(-) AGTGGACGTACCTCTGGTGTTCCGGTTGTCACGCCAGTGGCATTGCCGGGTAGCTAAGTACGGACGGGATAACCGCTGAAAGCATCTAAGCGGGAAGCCCCCCTCAAGATGAGATCTCCCGGACCTTTAAGGTCCCTAAAGATTCGTTGA 2837

PPFQ01000003.1:131083-135464(+) AGTGGACGTACCTCTGGTGTTCCGGTTGTCACGCCAGTGGCATTGCCGGGTAGCTAAGTACGGACGGGATAACCGCTGAAAGCATCTAAGCGGGAAGCCCCCCTCAAGATGAGATCTCCCGGACCTTTAAGGTCCCTAAAGATTCGTTGA 2837

NZ_AKYP01000181.1:1647-6020(-) AGTGGACGTACCTCTGGTGTTCCGGTTGTCACGCCAGTGGCATTGCCGGGTAGCTAAGTACGGACGGGATAACCGCTGAAAGCATCTAAGCGGGAAGCCCCCCTCAAGATGAGATCTCCCGGACCTTTAAGGTCCCTAAAGATTCGTTGA 2836

NOVI01000003.1:105955-110328(+) AGTGGACGTACCTCTGGTGTTCCGGTTGTCACGCCAGTGGCATTGCCGGGTAGCTAAGTACGGACGGGATAACCGCTGAAAGCATCTAAGCGGGAAGCCCCCCTCAAGATGAGATCTCCCGGACCTTTAAGGTCCCTAAAGATTCGTTGA 2837

CP018150.1:339294-343661(+) AGTGGACGTACCTCTGGTGTTCCGGTTGTCACGCCAGTGGCATTGCCGGGTAGCTAAGTACGGACGGGATAACCGCTGAAAGCATCTAAGCGGGAAGCCCCCCTCAAGATGAGATCTCCCGGACCTTTAAGGTCCCTAAAGATTCGTTGA 2837

Consensus AGACGACAACGTTGATAGGCAGGGTGTGGAAGCTCAGTAATGAGTGAAGCTAACCTGTACTAATTAATCGTGCGACTTGACTATGTAACCCTAAATGGTTTC 2939

JAKFBC010000002.1:34496-38842(-) (45) AGACGACAACGTTGATAGGCAGGGTGTGGAAGCTCAGTAATGAGTGAAGCTAACCTGTACTAATTAATCGTGCGACTTGACTAT------------------ 2911

NZ_AP019757.1:1739264-1743610(-) (31) AGACGACAACGTTGATAGGCAGGGTGTGGAAGCTCAGTAATGAGTGAAGCTAACCTGTACTAATTAATCGTGCGACTTGACTAT------------------ 2911

JBKOHN010000001.1:81530-85875(+) (25) AGACGACAACGTTGATAGGCAGGGTGTGGAAGCTCAGTAATGAGTGAAGCTAACCTGTACTAATTAATCGTGCGACTTGACTAT------------------ 2911

NC_011528.1:339307-343646(+) (14) AGACGACAACGTTGATAGGCAGGGTGTGGAAGCTCAGTAATGAGTGAAGCTAACCTGTACTAATTAATCGTGCGACTTGACTAT------------------ 2911

CP018005.1:167566-171940(+) (14) AGACGACAACGTTGATAGGCAGGGTGTGGAAGCTCAGTAATGAGTGAAGCTAACCTGTACTAATTAATCGTGCGACTTGACTATGTAACC------------ 2927

NZ_CCAM010000005.1:71418-75764(+) (13) AGACGACAACGTTGATAGGCAGGGTGTGGAAGCTCAGTAATGAGTGAAGCTAACCTGTACTAATTAATCGTGCGACTTGACTAT------------------ 2911

NZ_CCXO01000001.1:317226-321572(+) (11) AGACGACAACGTTGATAGGCAGGGTGTGGAAGCTCAGTAATGAGTGAAGCTAACCTGTACTAATTAATCGTGCGACTTGACTAT------------------ 2911

NC_011527.1:1751498-1755844(-) (8) AGACGACAACGTTGATAGGCAGGGTGTGGAAGCTCAGTAATGAGTGAAGCTAACCTGTACTAATTAATCGTGCGACTTGACTAT------------------ 2911

NZ_CP032542.1:88036-92382(-) (8) AGACGACAACGTTGATAGGCAGGGTGTGGAAGCTCAGTAATGAGTGAAGCTAACCTGTACTAATTAATCGTGCGACTTGACTAT------------------ 2911

CP014551.1:161236-165610(+) (8) AGACGACAACGTTGATAGGCAGGGTGTGGAAGCTCAGTAATGAGTGAAGCTAACCTGTACTAATTAATCGTGCGACTTGACTATGTAACC------------ 2927

CP013667.1:88011-92385(-) (8) AGACGACAACGTTGATAGGCAGGGTGTGGAAGCTCAGTAATGAGTGAAGCTAACCTGTACTAATTAATCGTGCGACTTGACTATGTAACC------------ 2927

CP107247.1:164350-168689(+) (7) AGACGACAACGTTGATAGGCAGGGTGTGGAAGCTCAGTAATGAGTGAAGCTAACCTGTACTAATTAATCGTGCGACTTGACTAT------------------ 2911

NZ_CP103435.1:167581-171920(+) (6) AGACGACAACGTTGATAGGCAGGGTGTGGAAGCTCAGTAATGAGTGAAGCTAACCTGTACTAATTAATCGTGCGACTTGACTAT------------------ 2911

CP014565.1:1711747-1716121(-) (3) AGACGACAACGTTGATAGGCAGGGTGTGGAAGCTCAGTAATGAGTGAAGCTAACCTGTACTAATTAATCGTGCGACTTGACTATGTAACC------------ 2927

AAYJ01000007.1:0-1404(+) (3) AGACGACAACGTTGATAGGCAGGGTGTGGAAGCTCAGTAATGAGTGAAGCTAACCTGTACTAATTAATCGTGCGACTTGACTAT------------------ 950

NZ_JAOXDR010000002.1:16672-21025(-) (3) AGACGACAACGTTGATAGGCAGGGTGTGGAAGCTCAGTAATGAGTGAAGCTAACCTGTACTAATTAATCGTGCGACTTGACTAT------------------ 2911

NZ_CP103432.1:167699-172038(+) (3) AGACGACAACGTTGATAGGCAGGGTGTGGAAGCTCAGTAATGAGTGAAGCTAACCTGTACTAATTAATCGTGCGACTTGACTAT------------------ 2911

NZ_CP103431.1:167697-172036(+) (3) AGACGACAACGTTGATAGGCAGGGTGTGGAAGCTCAGTAATGAGTGAAGCTAACCTGTACTAATTAATCGTGCGACTTGACTAT------------------ 2911

NZ_CP103428.1:167591-171929(+) (2) AGACGACAACGTTGATAGGCAGGGTGTGGAAGCTCAGTAATGAGTGAAGCTAACCTGTACTAATTAATCGTGCGACTTGACTAT------------------ 2911

NZ_JANTNR010000002.1:115350-119696(+) (2) AGACGACAACGTTGATAGGCAGGGTGTGGAAGCTCAGTAATGAGTGAAGCTAACCTGTACTAATTAATCGTGCGACTTGACTAT------------------ 2911

JARBIR010004564.1:3782-8128(-) (2) AGACGACAACGTTGATAGGCAGGGTGTGGAAGCTCAGTAATGAGTGAAGCTAACCTGTACTAATTAATCGTGCGACTTGACTAT------------------ 2911

NC_009727.1:1888349-1892695(-) (2) AGACGACAACGTTGATAGGCAGGGTGTGGAAGCTCAGTAATGAGTGAAGCTAACCTGTACTAATTAATCGTGCGACTTGACTAT------------------ 2911

AAYJ01000139.1:0-660(+) (2) ------------------------------------------------------------------------------------------------------ 594

CP032542.1:88018-92392(-) (2) AGACGACAACGTTGATAGGCAGGGTGTGGAAGCTCAGTAATGAGTGAAGCTAACCTGTACTAATTAATCGTGCGACTTGACTATGTAACC------------ 2927

CP014559.1:232744-237117(+) (2) AGACGACAACGTTGATAGGCAGGGTGTGGAAGCTCAGTAATGAGTGAAGCTAACCTGTACTAATTAATCGTGCGACTTGACTATGTAACC------------ 2927

CP007555.1:337408-341752(+) AGACGACAACGTTGATAGGCAGGGTGTGGAAGCTCAGTAATGAGTGAAGCTAACCTGTACTAATTAATCGTGCGACTTGACTAT------------------ 2909

NZ_CP014354.1:76710-81178(-) AGACGACAACGTTGATAGGCAGGGTGTGGAAGCTCAGTAATGAGTGAAGCTAACCTGTACTAATTAATCGTGCGACTTGACTAT------------------ 2921

CP000890.1:243129-244433(+) ------------------------------------------------------------------------------------------------------ 787

NZ_CP115461.1:167576-171922(+) AGACGACAACGTTGATAGGCAGGGTGTGGAAGCTCAGTAATGAGTGAAGCTAACCTGTACTAATTAATCGTGCGACTTGACTAT------------------ 2911

AP019759.1:167578-171919(+) AGACGACAACGTTGATAGGCAGGGTGTGGAAGCTCAGTAATGAGTGAAGCTAACCTGTACTAATTAATCGTGCGACTTGAC--------------------- 2906

AP019757.1:1739267-1743608(-) AGACGACAACGTTGATAGGCAGGGTGTGGAAGCTCAGTAATGAGTGAAGCTAACCTGTACTAATTAATCGTGCGACTTGAC--------------------- 2906

NOLN01000021.1:90861-95235(-) AGACGACAACGTTGATAGGCAGGGTGTGGAAGCTCAGTAATGAGTGAAGCTAACCTGTACTAATTAATCGTGCGACTTGACTATGTAACC------------ 2927

NOLM01000022.1:91202-95576(-) AGACGACAACGTTGATAGGCAGGGTGTGGAAGCTCAGTAATGAGTGAAGCTAACCTGTACTAATTAATCGTGCGACTTGACTATGTAACC------------ 2927

PDLP01000011.1:14431-18805(+) AGACGACAACGTTGATAGGCAGGGTGTGGAAGCTCAGTAATGAGTGAAGCTAACCTGTACTAATTAATCGTGCGACTTGACTATGTAACC------------ 2927

CP014563.1:522940-527314(+) AGACGACAACGTTGATAGGCAGGGTGTGGAAGCTCAGTAATGAGTGAAGCTAACCTGTACTAATTAATCGTGCGACTTGACTATGTAACC------------ 2927

CP000890.1:244922-245905(+) AGACGACAACGTTGATAGGCAGGGTGTGGAAGCTCAGTAATGAGTGAAGCTAACCTGTACTAATTAATCGTGCGACTTGACTAT------------------ 950

NZ_NOLR01000002.1:131843-136189(+) AGACGACAACGTTGATAGGCAGGGTGTGGAAGCTCAGTAATGAGTGAAGCTAACCTGTACTAATTAATCGTGCGACTTGACTAT------------------ 2911

NZ_JAOXDP010000002.1:15794-20139(-) AGACGACAACGTTGATAGGCAGGGTGTGGAAGCTCAGTAATGAGTGAAGCTAACCTGTACTAATTAATCGTGCGACTTGACTAT------------------ 2911

NZ_PPFR01000008.1:34378-38724(-) AGACGACAACGTTGATAGGCAGGGTGTGGAAGCTCAGTAATGAGTGAAGCTAACCTGTACTAATTAATCGTGCGACTTGACTAT------------------ 2911

NZ_PPFQ01000003.1:131093-135446(+) AGACGACAACGTTGATAGGCAGGGTGTGGAAGCTCAGTAATGAGTGAAGCTAACCTGTACTAATTAATCGTGCGACTTGACTAT------------------ 2911

NZ_LK937696.1:167613-171953(+) AGACGACAACGTTGATAGGCAGGGTGTGGAAGCTCAGTAATGAGTGAAGCTAACCTGTACTAATTAATCGTGCGACTTGACTAT------------------ 2911

NZ_NOVI01000003.1:105965-110310(+) AGACGACAACGTTGATAGGCAGGGTGTGGAAGCTTAGTAATGAGTGAAGCTAACCTGTACTAATTAATCGTGCGACTTGACTAT------------------ 2911

NZ_CP103426.1:167642-171981(+) AGACGACAACGTTGATAGGCAGGGTGTGGAAGCTCAGTAATGAGTGAAGCTAACCTGTACTAATTAATCGTGCGACTTGACTAT------------------ 2911

NZ_JAOXDN010000007.1:36985-41324(-) AGACGACAACGTTGATAGGCAGGGTGTGGAAGCTCAGTAATGAGTGAAGCTAACCTGTACTAATTAATCGTGCGACTTGACTAT------------------ 2911

NZ_JPVV01000013.1:13888-18051(+) AGACGACAACGTTGATAGGCAGGGTGTGGAAGCTCAGTAATGAGTGAAGCTAACCTGTACTAATTAATCGTGCGACTTGACTAT------------------ 2911

NZ_CP007555.1:337406-341752(+) AGACGACAACGTTGATAGGCAGGGTGTGGAAGCTCAGTAATGAGTGAAGCTAACCTGTACTAATTAATCGTGCGACTTGACTAT------------------ 2911

NZ_JASNNV010000010.1:39284-43631(-) AGACGACAACGTTGATAGGCAGGGTGTGGAAGCTCAGTAATGAGTGAAGCTAACCTGTACTAATTAATCGTGCGACTTGACTAT------------------ 2912

NZ_JAOXFC010000012.1:14231-18577(+) AGACGACAACGTTGATAGGCAGGGTGTGGAAGCTCAGTAATGAGTGAAGCTAACCTGTACTAATTAATCGTGCGACTTGACTAT------------------ 2911

NZ_CP103430.1:167658-171997(+) AGACGACAACGTTGATAGGCAGGGTGTGGAAGCTCAGTAATGAGTGAAGCTAACCTGTACTAATTAATCGTGCGACTTGACTAT------------------ 2911

NZ_PDLP01000011.1:14441-18787(+) AGACGACAACGTTGATAGGCAGGGTGTGGAAGCTCAGTAATGAGTGAAGCTAACCTGTACTAATTAATCGTGCGACTTGACTAT------------------ 2911

NZ_NOLM01000022.1:91220-95566(-) AGACGACAACGTTGATAGGCAGGGTGTGGAAGCTCAGTAATGAGTGAAGCTAACCTGTACTAATTAATCGTGCGACTTGACTAT------------------ 2911

JBJCIS010000056.1:0-2780(-) ------------------------------------------------------------------------------------------------------ 1961

NZ_CDBG01000001.1:179744-184090(+) AGACGACAACGTTGATAGGCAGGGTGTGGAAGCTCAGTAATGAGTGAAGCTAACCTGTACTAATTAATCGTGCGACTTGACTAT------------------ 2911

NZ_CP103434.1:167622-171961(+) AGACGACAACGTTGATAGGCAGGGTGTGGAAGCTCAGTAATGAGTGAAGCTAACCTGTACTAATTAATCGTGCGACTTGACTAT------------------ 2911

CP014354.1:76692-80603(-) AGACGACAACGTTGATAGGCAGGGTGTGGAAGCTCAGTAATGAGTGAAGCTAACCTGTACTAATTAATCGTGCGACTTGACTATGTAACCCTAAATGGTTTC 2464

NOLR01000002.1:131833-136207(+) AGACGACAACGTTGATAGGCAGGGTGTGGAAGCTCAGTAATGAGTGAAGCTAACCTGTACTAATTAATCGTGCGACTTGACTATGTAACCCTAAATGGTTTC 2939

PPFR01000008.1:34360-38734(-) AGACGACAACGTTGATAGGCAGGGTGTGGAAGCTCAGTAATGAGTGAAGCTAACCTGTACTAATTAATCGTGCGACTTGACTATGTAACCCTAAATGGTTTC 2939

PPFQ01000003.1:131083-135464(+) AGACGACAACGTTGATAGGCAGGGTGTGGAAGCTCAGTAATGAGTGAAGCTAACCTGTACTAATTAATCGTGCGACTTGACTATGTAACCCTAAATGGTTTC 2939

NZ_AKYP01000181.1:1647-6020(-) AGACGACAACGTTGATAGGCAGGGTGTGGAAGCTCAGTAATGAGTGAAGCTAACCTGTACTAATTAATCGTGCGACTTGACTATGTAACCCTAAATGGTTTC 2938

NOVI01000003.1:105955-110328(+) AGACGACAACGTTGATAGGCAGGGTGTGGAAGCTTAGTAATGAGTGAAGCTAACCTGTACTAATTAATCGTGCGACTTGACTATGTAACCCTAAATGGTTTC 2939

CP018150.1:339294-343661(+) AGACGACAACGTTGATAGGCAGGGTGTGGAAGCTCAGTAATGAGTGAAGCTAACCTGTACTAATTAATCGTGCGACTTGACTATGTAACCCTAAATGGTTTC 2939
