# Supplementary material for: Cryo-EM reveals multiple mechanisms of ribosome inhibition by doxycycline
Source: Nat Commun. 2026 Jun 1;17:7049. doi: 10.1038/s41467-026-73421-5 (PMC13392368; doi:10.1038/s41467-026-73421-5)
Supplement: Supplementary file 6 — Source Data [file 41467_2026_73421_MOESM6_ESM.zip › File 5.docx]

**Source data file 5: species alignment of 23S rRNA.** The consensus of the alignment of *Coxiella* 23s rRNA sequences (Supplementary file 1) was aligned with representative rRNA sequences from species that have a ribosome structure in the PDB. Four other proteobacteria (*Acinetobacter baumannii*, *Escherichia coli*, *Pseudomonas aeruginosa*, and *Vibrio natriegens*) and two Hydrobacteria (*Porphyromonas gingivalis* and *Borrelia burgdorferi*) have such structures. Where possible, the sequence of the same strain used for the structure was used; where the genome of this strain is not available in NCBI, an alternative of the same species was chosen. These seven sequences were aligned together using the MUSCLE algorithm in Geneious v.2026.0.2. Bases within 4 Å of a doxycycline molecule in the 50S ribosome structure in complex with doxycycline are indicated with an arrow. In the structure, these are bases U1796, A2074, A2075, A2078, U2457, G2521, U2522, U2601, U2602, U2625, C2626, and C2627. These are indicated in the alignment with arrows, coloured red for those conserved in all species. Three bases show alterations in one or both Hydrobacteria (blue arrows); U1796 and U2602 are both altered to cytosine. These two bases form a highly conserved non-Watson-Crick base pair, which forms with both a U=U or C=C pair. Comparison of the *P. gingivalis* and *C. burnetii* ribosomes shows that there is no alteration in the location of these bases (Supplementary Figure 13); the same is true for the *B. burgdorferi* structure. In *P. gingivalis,* C2627 is altered to uracil. This base makes a Watson-Crick base pair (to G2073 in *C. burnetii*). In *P. gingivalis*, the equivalent base alters to adenine to maintain the Watson-Crick base pair. U1796, U2602, and C2627 all contact the doxycycline molecules through base stacking (especially with the phenyl rings of two doxycycline molecules), with no interactions with the hydrogen bond donors or acceptors on the bases. Consequently, these alterations are conservative and are likely to support a doxycycline triple stack in each of these organisms.

Consensus AGGGTTRYATGGTCAAGTGAATAAGCGCATACGGTGGATGCCTTGGCAGTCAGAGGCGATGAAGGACGTGGTAACCTGCGAAAAGCTTCGGGGAGGCGGCAAACACCCTTTGATCCGGAGATTTCCGAATGGGGAAACCCACCTAGTTWA 150

*A baumannii* ----------AGTCAAGTAATTAAGTGCATGTGGTGGATGCCTTGGCAGTCAGAGGCGATGAAAGACGTGATAGCCTGCGAAAAGCTCCGGGGAGGCGGCAAATATCCTTTGATCCGGAGATGTCTGAATGGGGGAACCCACCTACTTTA 140

*B burgdorferi* ------ATATGGTCAAAGTAATAAGAGTCTATGGTGAATGCCTAGGAGCTTTAAGGCGAAGAAGGTCGTGGTAAGCTGCGAAAAGCTTGGGGGAG-AAGCAAACATTTATTGATCCCAAGATTACCGAATGGAGTAATCCAGCTAGCAAG 143

*C burnetii* AGGGTTGCATGGTCAAGTGAATAAGCGCATATGGTGGATGCCTTGGCAGTAAGAGGCGATGAAGGACGTAGTAGCCTGCGATAAGCTTCGGGGAGCTGGCAAACAAGCGATGATCCGGAGATTTCCGAATGGGGAAACCCAACCTTTC-- 148

*E coli* ----------GGTTAAGCGACTAAGCGTACACGGTGGATGCCCTGGCAGTCAGAGGCGATGAAGGACGTGCTAATCTGCGATAAGCGTCGGTAAGGTGATATGAACCGTTATAACCGGCGATTTCCGAATGGGGAAACCCAGTGTGTTTC 140

*P aeruginosa* ----------GGTCAAGTGAAGAAGCGCATACGGTGGATGCCTTGGCAGTCAGAGGCGATGAAAGACGTGGTAGCCTGCGAAAAGCTTCGGGGAGTCGGCAAACAGACTTTGATCCGGAGATCTCTGAATGGGGGAACCCACCTAGGATA 140

*P gingivalis* ----------------------AAGGGCAGACGGTGGATGCCTAGGCTCTCGGAGGCGAAGAAGGACGTGATAAGCTGCGAAAAGCTGCGGGAAT-CGGCACATACGAATTGATCCGCAGATATCCGAATGGGGCAACCCGTCAGGCCAA 127

*V natriegens* ----------NGTTAAGTGACTAAGCGTACACGGTGGATGCCTTGGCAGTCAGAGGCGATGAAGGACGTATTAACTTGCGATAAGCCCAGATTAGGCAGTAAAAGCCACTTGAGTCTGGGATTTCCGAATGGGGAAACCCACTTACATA- 139

Consensus --GGTAGGTATCDTGTACTGAATNCATAGGTWTAAGAGGCG-AACCAGGGGAACTGAAACATCTAAGTACCCTGAGGAAAAGAAATCAACCGAGATTCCCTCAGTAGCGGCGAGCGAACGGGGATTAGCCCATAAVCN-NNTN------- 289

*A baumannii* -AGGTAGGTATTGCAACATGAATACATAGTGTTGCAAGGCG-AACGAGGGGAAGTGAAACATCTCAGTACCCTTAGGAAAAGAAATCAATTGAGATTCCCTCAGTAGCGGCGAGCGAACGGGGATCAGCCCATTAAG-----T------- 276

*B burgdorferi* ATGCTAGCTATCTATTATC-------TAAATAATAGAGGCGATACCAGGGGAAGTGAACCATCTAAGTACCCTGAGGAAAAGAAATCAA-AGAGATTCCCTTAGTAGTGGCGAGCGAAAAGGGAGTAGCCCAAACTTTAAATGTGTCAAG 285

*C burnetii* ---GAGGTTATCGTATACTGAATTCATAGGTATACGAGGCG-AACCTGGGGAACTGAAACATCTAAGTACCCAGAGGAAAAGAAATCAACCGAGATTCCGTCAGTAGCGGCGAGCGAAAGCGGAACAGCCCAGTTACTAAATC------- 287

*E coli* -GACACACTATCATTAACTGAATCCATAGGTTAATGAGGCG-AACCGGGGGAACTGAAACATCTAAGTACCCCGAGGAAAAGAAATCAACCGAGATTCCCCCAGTAGCGGCGAGCGAACGGGGAGGAGCCCAGAGCCTGAATC------- 281

*P aeruginosa* -ACCTAGGTATCTTGTACTGAATCCATAGGTGCAAGAGGCG-AACCAGGGGAACTGAAACATCTAAGTACCCTGAGGAAAAGAAATCAACCGAGATTCCCTTAGTAGTGGCGAGCGAACGGGGATTAGCCCTTAAGC------------- 275

*P gingivalis* --GGCCTGACACATGAATTGAT---------TTCATGAGCG-AACGCGGGGAACTGAAACATCTCATTACCCGTAGGAGAAGAAAACAAAAGTGATTCCCTCAGTAGTGGCGAGCGAACGGGGATTAGCCCAAACCGGCTTTGTTTCG-- 263

*V natriegens* --AGTAAGTATCCTGTTGTGAATACATAGCAACAGGAGGCG-AACCGGGGGAACTGAAACATCTAAGTACCCCGAGGAAAAGAAATCAACCGAGATTCCGAAAGTAGCGGCGAGCGAAATTGGACTAGCCCTTAAGC------------- 273

Consensus ---------------------------------------------------------------------AWTRTTTGTTTTAGHRGAACGBTCTGGAAAGTBCGGCCATAGAGGGTGATAGCCCCGTACDCGAAAGGGYAAAT------- 363

*A baumannii* ---------------------------------------------------------------------TATGTGTGTTTTAGTGGAACGCTCTGGGAAGTGCGAACGTAGAGGGTGATATTCCCGTACACGAAAGGGCACAC------- 350

*B burgdorferi* CTGCAGAGCGTTGCATTTATGGGGTTGTAGGACGTTTAGGCTTAGTCTGTAATAAGCAAAAAAGTTACAAAATATTTATATAGAAGAATAATCTGGAAAGTTTAACCAAAGAAGGTGATAGTCCTGTAATTTAAATGTAAATA-TCTTTT 434

*C burnetii* ---------------------------------------------------------------------ATTATTTGTTCTAGCAGAATGTTCTGGAAAGTTCAGCCATAGCGGGTGATAGCCCCGTACGCGAAAGAGTAAAT------- 361

*E coli* ---------------------------------------------------------------------AGTGTGTGTGTTAGTGGAAGCGTCTGGAAAGGCGCGCGATACAGGGTGACAGCCCCGTACACAAAAATGCACAT------- 355

*P aeruginosa* ---------------------------------------------------------------------TTCATTGATTTTAGCGGAACGCTCTGGAAAGTGCGGCCATAGTGGGTGATAGCCCCGTACGCGAAAGGATCTTT------- 349

*P gingivalis* ------------GCAAAGTCGGGGTAATAGGACTTCG--------------------ACAAAAGTTT--AATGATAGATATAGGAGAACCTACTGGAAAGTATGGCCGAAGAGCATGAAAGCTGCGTATCCGACATATCGAACAATAGAC 379

*V natriegens* ---------------------------------------------------------------------TTTACACACGTTAGACGAACGGTCTGGAAAGGCCGACGATACAGGGTGATAGTCCCGTAGTTGACGATGTGTGT------- 347

Consensus -AMAG----TGA---AGATGAGTAGGGCGGGACACGTGAWATCTTGTCTGAATATGGGGGGACCATCCTCCAAGGCTAAATACTCCTGACTGACCGATAGTGAACCAGTACCGTGAGGGAAAGGCGAAAAGAACCCCGGHGAGGGGAGTG 505

*A baumannii* -ATAA----TGA---TGACGAGTAGGGCGAGGCACGTGAAACCTTGTCTGAATATGGGGGGACCATCCTCCAAGGCTAAATACTCCTGACTGACCGATAGTGAACCAGTACCGTGAGGGAAAGGCGAAAAGAACCCCTGTGAGGGGAGTG 492

*B burgdorferi* TAAAA----TGT---TCCTGAGTAGGACGAGGCACGAGAAACCTTGTTTGAAGCTGGGGAGACCACTCTCCAAGGCTAAATACTAGAAAGCTACCGATAGAGAA-GAGTACCGTGAGGGAAAGGTGAAAAGAACCCCG-GGAGGGGAGTG 575

*C burnetii* -AATGTGGGTAA---CGATGAGTAGGTCGGGACACGTGGTATCTTGACTGAACATGGGGGGACCATCCTCCAAGGCTAAATACTCCTTACTGACCGATAGCGAACCAGTACCGTGAGGGAAAGGTGAAAAGAACCCCGGCGAGGGGAGTG 507

*E coli* -GCTG----TGAGCTCGATGAGTAGGGCGGGACACGTGGTATCCTGTCTGAATATGGGGGGACCATCCTCCAAGGCTAAATACTCCTGACTGACCGATAGTGAACCAGTACCGTGAGGGAAAGGCGAAAAGAACCCCGGCGAGGGGAGTG 500

*P aeruginosa* -GAAG----TGA---AATCGAGTAGGACGGAGCACGAGAAACTTTGTCTGAACATGGGGGGACCATCCTCCAAGGCTAAATACTACTGACTGACCGATAGTGAACCAGTACCGTGAGGGAAAGGCGAAAAGAACCCCGGAGAGGGGAGTG 491

*P gingivalis* GACGG----AGC---ACCTGAGTAGCGCGGGACACGAGACATCCTGTGTGAATTGGCGGGGCCCATCCCGTAAGGCTAAATACTCCCGAGAGACCGATAGTGAACCAGTACCGTGAGGGAAAGGTGAAAAGAACCTCGAACAGAGGAGTG 522

*V natriegens* -TCAG----TGA---AATCGAGTAGGGCGGGACACGTGATATCCTGTCTGAATATGGGGGGACCATCCTCCAAGGCTAAATACTACTGACTGACCGATAGTGAACCAGTACCGTGAGGGAAAGGCGAAAAGAACCCCTGTGAGGGGAGTG 489

Consensus AAATAGAACCTGAAACCGTATGCGTACAAGCAGTRGGAGCATBCT---TGTGGTGTGACTGCGTACCTTTTGTATAATGGGTCAGCGACTTATATTCTGTAGCAAGGTTAACCG-T---------ATAGGGGAGCCGTAGSGAAASCGAG 642

*A baumannii* AAATAGATCCTGAAACCGCATGCATACAAGCAGTGGGAGCACCTT---CGTGGTGTGACTGCGTACCTTTTGTATAATGGGTCAGCGACTTATATTCAGTAGCGAGGTTAACCG-T---------ATAGGGGAGCCGTAGAGAAATCGAG 629

*B burgdorferi* AAATAGAA-CTGAAACCGTAGACTTACAAGCAGTCAAAGCCGTAATTTATTGCGGTGATGGCGTGCCTTTTGCATAATGAACCTGCGAGTTATCATGTCTAGCAAGATTAAAGCAT-AG----AAGTGCTGGAGTCGAAGCGAAAGCGAG 719

*C burnetii* AAATAGAACCTGAAACCGTATGCGTACAAGCAGTAGGAGCATTTCTTCGGAAATGTGACTGCGTACCTTTTGTATAATGGGTCAGCGACTTACTTGTTGTAGCGAGCTTAACCG-T---------CTAGGGGAGGCGTAGGGAAACCGAG 647

*E coli* AAAAAGAACCTGAAACCGTGTACGTACAAGCAGTGGGAGCATGCT--TAGGCGTGTGACTGCGTACCTTTTGTATAATGGGTCAGCGACTTATATTCTGTAGCAAGGTTAACCG-A---------ATAGGGGAGCCGAAGGGAAACCGAG 638

*P aeruginosa* AAATAGAACCTGAAACCGTATGCGTACAAGCAGTGGGAGCCTACT---TGTTAGGTGACTGCGTACCTTTTGTATAATGGGTCAGCGACTTATATTCAGTGGCAAGCTTAACCG-T---------ATAGGGTAGGCGTAGCGAAAGCGAG 628

*P gingivalis* CAATAGACCCTGAACCCGTCTGCCTACAAGCGGTAGGAGCGCCAT---TAAGGTGTGACTGCGTGCCTTTTGCATAATGAACCTACGAGTTACTGTTTGTGGCAAGGTTAATTG-TTATAATCAAGACAAGGAGCCGAAGCGAAAGCGAG 668

*V natriegens* AAATAGAACCTGAAACCGTGTACGTACAAGCAGTAGGAGCAGGCT---TGTCCTGTGACTGCGTACCTTTTGTATAATGGGTCAGCGACTTATATTCAGTGGCAAGGTTAACCA-T---------CTAGGGGAGCCGTAGGGAAACCGAG 626

Consensus TCTTAAAAGGGCG----TTTAGTTGCWGGGTATAGACCCGAAACCRGGTGATCTATCCATGGGCAGGTTGAAGGTTGGGTAACACTAACTGGAGGACCGAACCCACTAATGTTGAAAAATTAGGGGATGACTTGTGGATAGGGGTGAAAG 788

*A baumannii* TCTTAATAGGGCG----TTTAGTTGCTGGGTATAGACCCGAAACCAGGCGATCTATCCATGAGCAGGTTGAAGGTTGGGTAACACTAACTGGAGGACCGAACCCACTGTCGTTGAAAAGCCAGGGGATGACTTGTGGATAGGGGTGAAAG 775

*B burgdorferi* TCTTAAAAGGGCG---ATTTAGTTAGATGTGGTAGACCCGAAGCCGAGTGATCTATTTATGGCCAGGCTGAAGCTTGGGTAAAACCAAGTGGAGGGCCGAACTCTAGTCTGTTTAAAAAGGCAGYGATGAGCTGTGAATAGGAGTGAAAG 866

*C burnetii* TCCGAAATGGGCG----TTTAGTTGCAACGAGTAGACCCGAAACCGAGCGATCTATCTATGGCCAGGGTGAAGGTCAGGTAACACTGACTGGAGGCCCGAACCCACTAATGTTGAAAAATTAGGGGATGAGCTGTGGATAGGAGTGAAAG 793

*E coli* TCTTAACTGGGCG----TTAAGTTGCAGGGTATAGACCCGAAACCCGGTGATCTAGCCATGGGCAGGTTGAAGGTTGGGTAACACTAACTGGAGGACCGAACCGACTAATGTTGAAAAATTAGCGGATGACTTGTGGCTGGGGGTGAAAG 784

*P aeruginosa* TCTTAATAGGGCG----TTTAGTCGCTGGGTATAGACCCGAAACCGGGCGATCTATCCATGAGCAGGTTGAAGGTTAGGTAACACTGACTGGAGGACCGAACCCACTCCCGTTGAAAAGGTAGGGGATGACTTGTGGATCGGAGTGAAAG 774

*P gingivalis* TCTTAAAAGGGCGCCCATTTAGTCACGAGCAGTAGACGCGAAACCAAGTGATCTACCCTTGGTCAGGTTGAAGGTTAGGTAACACTAACTGGAGGACCGAATCGGTAAGCGTTGAAAAGCTTTCGAATGAACTGAGGGTAGGGGTGAAAG 818

*V natriegens* TCTTAACTGGGCG----TTCAGTCTCTGGATATAGACCCGAAACCAGGTGATCTAGCCATGGGCAGGTTGAAGGTTGAGTAACATCAACTGGAGGACCGAACCGACTAATGTTGAAAAATTAGCGGATGACTTGTGGCTAGGGGTGAAAG 772

Consensus GCTAATCAAACTYGGAGATAGCTGGTTCTCCCCGAAAGCTATTTAGGTAGCGCCTCGTGT-ATGACTAYTGGGGGTAGAGCACTGTTTCGGCTAGGGGGTCATCCCGRCTTACCAAACCGATGCAAACTCCGAATACCARTAAGTGCT-A 936

*A baumannii* GCTAATCAAGCCTGGTGATAGCTGGTTCTCCCCGAAAGCTATTTAGGTAGCGCCTCGGACGAATACCATAGGGGGTAGAGCACTGTTTCGGCTAGGGGGTCATCCCGACTTACCAAACCGATGCAAACTCCGAATACCTATGAGTACT-A 924

*B burgdorferi* GCTAAACAAACTCGGAGATAGCTGGTTCTCCCCGAAATGGATTTAAGTTCAGCCTTATTT-TAGTTTAATAGAGGTAGAGCACTAATTGAGCTAGGGCC-TGTCAAAGGGTACCAAACTCAGTTAAACTCCGAATGCTATTAAATGAT-G 1013

*C burnetii* GCTAATCAAGCTCGGAGATAGCTGGTTCTCCTCGAAAGCTATTTAGGTAGCGCCTCGTGT-ATGACTCTTGGGGGTAGAGCACTGTTTCGGCTAGGGGGCCATCCCGGCCTACCAAACCGAGGCAAACTCCGAATACCAAGAAGTTTT-A 941

*E coli* GCCAATCAAACCGGGAGATAGCTGGTTCTCCCCGAAAGCTATTTAGGTAGCGCCTCGTGAACTCATCTCCGGGGGTAGAGCACTGTTTCGGCAAGGGGGTCATCCCGACTTACCAACCCGATGCAAACTGCGAATACCGGAGAATGTT-A 933

*P aeruginosa* GCTAATCAAGCTCGGAGATAGCTGGTTCTCCTCGAAAGCTATTTAGGTAGCGCCTCATGT-ATCACTCTGGGGGGTAGAGCACTGTTTCGGCTAGGGGGTCATCCCGACTTACCAAACCGATGCAAACTCCGAATACCCAGAAGTGCCGA 923

*P gingivalis* GCTAATCAAACTTGGAGATAGCTCGTACTCCCCGAAATGCATTTAGGTGCAGCCTGTTGG-ATGTTATCATGAGGTAGAGCGACTGATTGGATGCGAGGGTTTCACCGCCTATCAAGTCCAGATAAACTCCGAATGCATGATAATTGA-C 966

*V natriegens* GCCAATCAAACCTGGAGATAGCTGGTTCTCCCCGAAAGCTATTTAGGTAGCGCCTCGGACGAATACTACTGGGGGTAGAGCACTGTTAAGGCTAGG-----------------CAACCCTTTGCAAACTCCGAATACCAGTAAGTACT-A 904

Consensus -CACGGGAGACACACKGCGGGTGCTAACGTCCGTCGTGGAGAGGGAAACAACCCAGACCGCCAGCTAAGGTCCCAAAATCATTGTTAAGTGGGA--AACGATGTGGGAAGGCTYAGACAGCYAGGAGGTTGGCTTAGAAGCAGCCAT-CC 1082

*A baumannii* -TCCGGGAGACAGACTGCGGGTGCTAACGTCCGTAGTCAAGAGGAAAACAATCCAGACCGCCAGCTAAGGCCCCAAAATCATAGTTAAGTGGGA--AACGATGTGGGAAGGCATAGACAGCTAGGAGGTTGGCTTAGAAGCAGCCAC-CC 1070

*B burgdorferi* -AATAGGAGTGAGACTYTGGGCGATAAGGTTCATAGTCGAGAGGGAAACAACCCAGACCAACAGCTAAGGTCTCAAAAAT-GTGTTAAGTGGAA--AAGGAGGTTTAGGTACGTAAACAGCCAGGAGGTTGGCTTAGAAGCAGCCATACC 1159

*C burnetii* GCACGGGAGACACACTGCGGGTGATAAGGTCCGTGGTGGAAAGGGAAACAGCCCAGATCGCCAGCTAAGGTCCCAAAATCACAGTTAAGTGGAA--AACGATGTGGGAAGGCTCAGACAGCCAGGAGGTTGGCTTAGAAGCAGCCAT-CC 1088

*E coli* TCACGGGAGACACACGGCGGGTGCTAACGTCCGTCGTGAAGAGGGAAACAACCCAGACCGCCAGCTAAGGTCCCAAAGTCATGGTTAAGTGGGA--AACGATGTGGGAAGGCCCAGACAGCCAGGATGTTGGCTTAGAAGCAGCCAT-CA 1080

*P aeruginosa* GCATGGGAGACACACGGCGGGTGCTAACGTCCGTCGTGAAAAGGGAAACAACCCAGACCGCCAGCTAAGGTCCCAAAGTTGTGGTTAAGTGGTA--AACGATGTGGGAAGGCTTAGACAGCTAGGAGGTTGGCTTAGAAGCAGCCAC-CC 1070

*P gingivalis* -CGATGGAGTGAGGGCATGGGTGCTAAGGTCCATGTCCGAGAGGAGAAGAATCCGGACCACCGGCTAAGGTCCCGAAATAATTGCTAAGTTGTAAAAACGAAGTCAAGATGCAGAGACAGCTAGGATGTTGGCTTGGAAGCAGCCATTCA 1115

*V natriegens* -TCCGGGAGACACACGGCGGGTGCTAACGTCCGTCGTGGAGAGGGAAACAACCCAGACCGCCAGCTAAGGTCCCAAATTA-CTACTAAGTGGGA--AACGATGTGGGAAGGCTCAGACA------------------------------- 1019

Consensus TTTAAAGAAAGCGTAATAGCTCACTRGTCGAGTCGGCCTGCGCGGAAGATGTAACGGGGCTA-AACAATGTACCGAAGCTGCGGCAGTAAAS-AA----TG-NTTAHTGGGTAGGGGAGCGTTCTGTAAGCCTGTGAAGGTGAATTGAGA 1225

*A baumannii* TTTAAAGAAAGCGTAATAGCTCACTAGTCGAGTCGGCCTGCGCGGAAGATGTAACGGGGCTAAAACTATGTGCCGAAGCTGCGGATGTATAC-TT----TG-TATACGTGGTAGGGGAGCGTTCTGTAAGCCGATGAAGGTGTGTTGAGA 1214

*B burgdorferi* TTTAAAGAGTGCGTAATAGCTCACTGGTCGAGTACTTAAGCGCCGATAATGTAACGGGGCTA-AACACATTACCGAAGCTTTGGATCTTAACGAA----AG-TTAAGATGGTAGGGGAGCGTTCTGTAAGCCAGAGAAGTTAAACTGGAA 1303

*C burnetii* TTTAAAGAAAGCGTAATAGCTCACTGGTCGAGTCGTCCTGCGCGGAAGATTTAACGGGGCTCAAACTGTGTACCGAAGCTGCGGCATCAGAAGACACTGTCTTCTGATGGGTAGAGGAGCGTTCTGTAAGCCTGTGAAGGTGAATCGAGA 1238

*E coli* TTTAAAGAAAGCGTAATAGCTCACTGGTCGAGTCGGCCTGCGCGGAAGATGTAACGGGGCTA-AACCATGCACCGAAGCTGCGGCAGCGACG-CT--TATGCGTTGTTGGGTAGGGGAGCGTTCTGTAAGCCTGTGAAGGTGTACTGTGA 1226

*P aeruginosa* TTTAAAGAAAGCGTAATAGCTCACTAGTCGAGTCGGCCTGCGCGGAAGATGTAACGGGGCTCAAACCACACACCGAAGCTGCGGGTGTCACG-TA----AG--TGACGCGGTAGAGGAGCGTTCTGTAAGCCTGTGAAGGTGAGTTGAGA 1213

*P gingivalis* TTTAAAGAGTGCGTAACAGCTCACTAGTCGAGGATTTTGGCATGGATAATA-CACGGGCATA-AGCAATTTACCGAAGCCGTGGGATATAGT-AA--------TATATCGGTAGGGGAGCATTCCAGCGACGTAGAAGGGAAAAGGGCGA 1254

*V natriegens* -----------------------------GAGTCGGCCTGCGCGGAAGATGTAACGGGGCTA-AGTAGTAAACCGAAGCTGCGGCAATACT--A---------GTATTGGGTAGGGGAGCGTTCTGTAAGCGGTTGAAGGTGTGTGGTAA 1128

Consensus AGTTTGCTGGAGGTATCAGAAGTGCGAATGCTGACATGAGTAACGATAAAGGGGGTGAAAAACCCCCTCGCCGAAAGACCAAGGGTTCCTGTSCAACGTTAATCGGSGCAGGGTKAGTCGGCCCCTAAGGCGAGGCCGAAAGGCGTAGTC 1375

*A baumannii* AGCATGCTGGAGGTATCAGAAGTGCGAATGCTGACGTGAGTAACGACAAAACGGGTGAAAAACCCGTTCGCCGAAAGACCAAGGGTTCCAGTCCAACGTTAATCGGGGCTGGGTGAGTCGACCCCTAAGGCGAGGCCGAAAGGCGTAGTC 1364

*B burgdorferi* AGTTTGATGGAGGTATCAGAAGTGAGAATGCAGGTATGAGTAACGAAAAAATGGGTGAGATTCCCATTCGCCGAAAACCTAAGGTTTCCTGGGTAAAGGTCGTCTTCCCAGGGTTAGTCGGCCCCTAAGGCAAAGCTGAAAAGTGTAGTC 1453

*C burnetii* GGTTTGCTGGAGGTATCAGAAGTGCGAATGCTGACATAAGTAACGATAATGTGGGTGAAAAACCCACACGCCGAAAGTCTAAGGTTTCCTGCGCAACGTTAATCGGCGCAGGGTGAGTCGGCCCCTAAGGCGAGGCAGAAATGCGTAGTC 1388

*E coli* GGTATGCTGGAGGTATCAGAAGTGCGAATGCTGACATAAGTAACGATAAAGCGGGTGAAAAGCCCGCTCGCCGGAAGACCAAGGGTTCCTGTCCAACGTTAATCGGGGCAGGGTGAGTCGACCCCTAAGGCGAGGCCGAAAGGCGTAGTC 1376

*P aeruginosa* AGCTTGCTGGAGGTATCAGAAGTGCGAATGCTGACATGAGTAACGACAATGGGTGTGAAAAACACCCACGCCGAAAGACCAAGGGTTCCTGCGCAACGTTAATCGACGCAGGGTTAGTCGGTTCCTAAGGCGAGGCTGAAAAGCGTAGTC 1363

*P gingivalis* CTTTTTCTGGAGTTTCTGGAAAAGCAAATGTAGGTATAAGTAACGATAAAGGGGGCGAGAACCCCCCTCGCCGAAAGACCAAGGTTTCCTGATCAACGCTAATCGGATCAGGGTTAGTCGGGGCCTAAGGATAAGCCGAATGGCGATTCC 1404

*V natriegens* CGCATGCTGGACGTATCAGAAGTGCGAATGCTGACATGAGTAACGATAAAGGGGGTGAAAAACCTCCTCGCCGGAAGACCAAGGGTTCCTGTCCAACGTTAATCGGGGCAGGGTAAGTCGACCCCTAAGGCGAGGCCGAAAGGCGTAGTC 1278

Consensus GATGGGA-AACGGGTTAATATTCCCGTACTTCTTATTACTGCGATGGGGGGACGGAGAAGGCTARGT-CRGCCTGGCGATGGTTGTCCAGGTTTAAGGGTGTAGGCTKGGATCT---TAGGCAAATCCGGGAW-CT-CAAGGCT-GA--- 1514

*A baumannii* GATGGGA-AAATGGTTAATATTCCATTACTTCTGTGTAATGCGATGAGAGGACGGAGAAGGCTAAAT-CAGCCTGGCGTTGGTTGTCCAGGTGAAAGGATGTAGGCATGTATCT---TAGGCAAATCCGGGGTACT-CTATGCT-GA--- 1504

*B burgdorferi* GATGGGA-AACGGGTTAATATTCCCGTACCTCTTATAGTTTCGATGGAGTGACGCATGAGGTTAACTACTGCTAGGCGATGGTTGTCCTAGTTTAAGCATTAAGGCGATGATCTTAATAGGAAAATCCGTTAA----GAGAGCT-AA--- 1594

*C burnetii* GATGGGA-AACGGGTTAATATTCCCGTACTTTATAATACTGCGATGGGAGGACGGAGAAGGCTAGGT-CAGCCACCCGATGGTTGTGGTGGTTTAAGTGTGTAGGAAGGGTTCT---TTGGCAAATCCGGGAA-CT-CAATTCC-GA--- 1527

*E coli* GATGGGA-AACAGGTTAATATTCCTGTACTTGGTGTTACTGCGAAGGGGGGACGGAGAAGGCTATGT-TGGCCGGGCGACGGTTGTCCCGGTTTAAGCGTGTAGGCTGGTTTTC---CAGGCAAATCCGGAAA-AT-CAAGGCT-GA--- 1515

*P aeruginosa* GATGGGA-AACAGGTTAATATTCCTGTACTTCTGGTTACTGCGATGGAGGGACGGAGAAGGCTAGGC-CAGCTTGGCGTTGGTTGTCCAAGTTTAAGGTGGTAGGCTGAAATCT---TAGGTAAATCCGGGGT-TT-CAAGGCC-GA--- 1502

*P gingivalis* GATGGAAGAACCGGTTAATATTCCGGTACTGATACAGAGAGCGATGTGGTGACGGAGAAGTGACAGTCCGGCCGTCTGACGGAATAGGCGGTTAAAGGGTGTAGATGTTGATCGGGGTAGGCAAATCCGCCCT----GAGAGTCGAACCT 1550

*V natriegens* GATGGGA-AACGGGTTAATATTCCCGTACTTCTTACAATTGCGATGGGGGGACGGAGAAGGCTAGGT-GGGCCTGGCGACGGTTGTCCAGGTTCAAGTGCGTAGGCTTGAGAGT---TAGGTAAATCCGGCTCTCTCTAAGGCT-GA--- 1419

Consensus GACATGATGACGAGTHC-TA-CT-ACGAGDGYGAAGTGRTTGATGCCATGCTTCCAGGAAA-AGCCTCTAAGCTTCAGGTAATAAGGAACCGTACCCCAAACCGACACAGGTGGTCGGGTAGAGAATACCAAGGCGCTTGAGAGAACTCG 1660

*A baumannii* GATCTGATAGCAAGCTG-TA-CT-TGTACAGCGAAGTGGTTGATGCCATGCTTCCAGGAAA-AGTCTCTAAGCTTCAGTTACACAGGAATCGTACCCGAAACCGACACAGGTGGTCAGGTCGAGTAGACCAAGGCGCTTGAGAGAACTCT 1650

*B burgdorferi* GATGTGATGATGAGTGC-TATTTAGGTAGCATGAAATGTAGGTAGTCAAGGTGCCAAGAAATAGCTTCTAAGGTTAGGC-TATAAGGGACCGTACCGCAAACCGACACAGGTAGGTGGGATGAAAATTCTAAGGCGCGCGAGAGAATCCA 1742

*C burnetii* GACATGATGACGAAGTACGAACTTGTTCGTGCAAAGTGATTGATGCCACGCTTCCAGGAAA-AGTCCCTAAGCTTCAGGTATTGTAAAACCGTACTATAAACCGACACAGGTGGACAGGTAGAGAATACCAAGGCGCTTGAGAGAACTTG 1676

*E coli* GGCGTGATGACGAGGCA----CT-ACGGTGCTGAAGCAACAAATGCCCTGCTTCCAGGAAA-AGCCTCTAAGCATCAGGTAACATCAAATCGTACCCCAAACCGACACAGGTGGTCAGGTAGAGAATACCAAGGCGCTTGAGAGAACTCG 1659

*P aeruginosa* GAGCTGATGACGAGTCG-TCTTT-TAGATGACGAAGTGGTTGATGCCATGCTTCCAAGAAA-AGCTTCTAAGCTTCAGGTAACCAGGAACCGTACCCCAAACCGACACAGGTGGTCGGGTAGAGAATACCAAGGCGCTTGAGAGAACTCG 1649

*P gingivalis* GACAGTACCCGGAGTAC-AA-GT-ACGA-AGGGATAAGGACGTAAACCGGCTCCCAAGAAA-ACCCGCTAAGCATATTT-CTGTGTTACCCGTACCGTAAACCGACACAGGTGGTTGGGTTGAGTATACTAAGGCGCTCGAGAGATTCGC 1694

*V natriegens* GACACGACGTCGAGCAC----CT-ACGGGTGTGAAGTCATTGATGCCATGCTTCCAGGAAA-AGCCTCTAAGCTTCAGATTGTAAGGAATCGTACCCCAAACCGACACAGGTGGTCGGGTAGAGAATACCAAGGCGCTTGAGAGAACTCG 1563

Consensus GGTGAAGGAACTAGGCAAAATGGTACCGTAACTTCGGGAGAAGGTACGCCSTT--GTMGGTGAAGG-ACTTGCTNCTGGAGCTGAARAVGGTCGCAGATACCAGGTCGCTGCGACTGTTTATTAAAAACACAGCACTCTGCAAACACGWA 1807

*A baumannii* GCTGAAGGAACTAGGCAAAATGGTACCGTAACTTCGGGAGAAGGTACGCTGTT--GTTGGTGATGGAACTCGCTTCCTGAGCTGACGACAGCCGCAGAAACCAGGCCGCTGCAACTGTTTATTAAAAACATAGCACTCTGCAAACACGAA 1798

*B burgdorferi* CGTTAAGGAACTCTGCAAAATACGTACGTAACTTCGGGATAAGT-ACGACCTA--------------------------AGC--AATTAGGTAGCATAAAAATGGTCCAAACGACTGTTTACCAAAAACACAGGTCTCTGCAAATCTGTA 1863

*C burnetii* GGTGAAGGAACTAGGCAAAATGGCACCGTAACTTCGGGAGAAGGTGCGCCCTT-GGTAAGTGAAGGTCCTTGCGACTGGAGCTGAAAAGGGTTGCAGATACCAGGTGGCTGCGACTGTTTACTAAAAACACAGCACTCTGCAAACTCGTA 1825

*E coli* GGTGAAGGAACTAGGCAAAATGGTGCCGTAACTTCGGGAGAAGGCACGCTGATATGTAGGTGAAGCGACTTGCTCGTGGAGCTGAAATCAGTCGAAGATACCAGCTGGCTGCAACTGTTTATTAAAAACACAGCACTGTGCAAACACGAA 1809

*P aeruginosa* GGTGAAGGAACTAGGCAAAATGGCACCGTAACTTCGGGAGAAGGTGCGCCGGC--TAGGGTGAAGG-ATTTACTCCGTAAGCTCTGGCTGGTCGAAGATACCAGGCCGCTGCGACTGTTTATTAAAAACACAGCACTCTGCAAACACGAA 1796

*P gingivalis* GGTTAAGGAACTAGGCAAAATGGTCCTGTAACTTCGGGAGAAAGGACGCCTGT--CTC-------------------------CGGACAGGCCGCAGAAACCAGGCCCAGGCGACTGTTTAACAAAAACACAAGGCTATGCAAAAAAGCA 1817

*V natriegens* GGTGAAGGAACTAGGCAAAATGGTACCGTAACTTCGGGAGAAGGTACGCTCTC--GACGGTGAAGTCCCTCGCGGATGGAGCTATTGAGAGTCGCAGATACCAGGTGGCTGCAACTGTTTATTAAAAACACAGCACTGTGCAAAATCGTA 1711

Consensus AGTGGACGTATAGGGTGTGACGCCTGCCCGGTGCCGGAAGGTTAATTGATGGGGTTAGCG--CAAGCGAAGCTCTTGATCGAAGCCCCGGTAAACGGCGGCCGTAACTATAACGGTCCTAAGGTAGCGAAATTCCTTGTCGGGTAAGTTC 1955

*A baumannii* AGTGGACGTATAGGGTGTGATGCCTGCCCGGTGCTGGAAGGTTAATTGATGGGGTTAGCG--TAAGCGAAGCTCTTGATCGAAGCCCCAGTAAACGGCGGCCGTAACTATAACGGTCCTAAGGTAGCGAAATTCCTTGTCGGGTAAGTTC 1946

*B burgdorferi* AAGAGAAGTATAGGGACTGACACCTGCCCGGTGCTGGAAGGTTAAGAGGAGATGTTAGTT--TATACGAAGCATTGAATTTAAGCCCCAGTAAACGGCGGCCGTAACTATAACGGTCCTAAGGTAGCGAAATTCCTTGTCGGGTAAGTTC 2011

*C burnetii* AGAGGACGTATAGGGTGTGACGCCTGCCCGGTGCCGGAAGGTTAAGTGATGGGGTTAGCCCTCGGGCGAAGCTCTTGATCGAAGCCCCGGTAAACGGCGGCCGTAACTATAACGGTCCTAAGGTAGCGAAATTCCTTGTCGGGTAAGTTC 1975

*E coli* AGTGGACGTATACGGTGTGACGCCTGCCCGGTGCCGGAAGGTTAATTGATGGGGTTAGCG--CAAGCGAAGCTCTTGATCGAAGCCCCGGTAAACGGCGGCCGTAACTATAACGGTCCTAAGGTAGCGAAATTCCTTGTCGGGTAAGTTC 1957

*P aeruginosa* AGTGGACGTATAGGGTGTGACGCCTGCCCGGTGCCGGAAGGTTAATTGATGGGGTTAGCG--CAAGCGAAGCTCTTGATCGAAGCCCCGGTAAACGGCGGCCGTAACTATAACGGTCCTAAGGTAGCGAAATTCCTTGTCGGGTAAGTTC 1944

*P gingivalis* ATTTGAGGTATATAGTCTGACACCTGCCCGGTGCTGGAAGGTTAAGAGGAGGAGTCATCG-TCAAGAGAAGCTCTGAATTGAAGCCCCAGTAAACGGCGGCCGTAACTATAACGGTCCTAAGGTAGCGAAATTCCTTGTCGGGTAAGTTC 1966

*V natriegens* AGATGACGTATACGGTGTGACGCCTGCCCGGTGCCGGAAGGTTAATTGATGGGGTTAGACTTCGGTCGAAGCTCTTGATCGAAGCCCCGGTAAACGGCGGCCGTAACTATAACGGTCCTAAGGTAGCGAAATTCCTTGTCGGGTAAGTTC 1861

Consensus CGACCTGCACGAATGGCGTAACGATGGCGACGCTGTCTCCACCCGAGACTCAGTGAAATTGAAATCGCTGTGAAGATGCAGTGTACCCGCGGCTAGACGGAAAGACCCCGTGAACCTTTACTATAGCTTGACACTGAACTTTGACCCTRC 2105

*A baumannii* CGACCTGCACGAATGGCATAATGATGGCGGCGCTGTCTCCAGCAGAGGCTCAGTGAAATCGAAATCGCTGTGAAGATGCAGTGTACCCGCGGCTAGACGGAAAGACCCCGTGAACCTTTACTGCAGCTTGACACTGAACTTTGACCTTAC 2096

*B burgdorferi* CGACCCGCACGAATGGTGTAACGATTTGGACGCTGTCTCAACGTGGAGCTCGGTGAAATTGAAGTATCGGTGAAGATGCCGATTACTTGTGGTTAGACGGAAAGACCCCGTGAACCTTTACTATAGCTTGGTATTGAGATTTGATTAAAT 2161

*C burnetii* CGACCTGCACGAATGGCGTAACGATAGCCACGCTGTCTCCACCCAAGACTCAGTGAAATTGAAATCGCTGTGAAGATGCAGCGTACCCGCGGCTAGACGGAAAGACCCCGTGAACCTTTACTACAGCTTTACACTGAACTTTGAATATGC 2125

*E coli* CGACCTGCACGAATGGCGTAATGATGGCCAGGCTGTCTCCACCCGAGACTCAGTGAAATTGAACTCGCTGTGAAGATGCAGTGTACCCGCGGCAAGACGGAAAGACCCCGTGAACCTTTACTATAGCTTGACACTGAACATTGAGCCTTG 2107

*P aeruginosa* CGACCTGCACGAATGGCGTAACGATGGCGGCGCTGTCTCCACCCGAGACTCAGTGAAATTGAAATCGCTGTGAAGATGCAGTGTATCCGCGGCTAGACGGAAAGACCCCGTGAACCTTTACTGTAGCTTTGCACTGGACTTTGAGCCTGC 2094

*P gingivalis* CGACCTGCACGAATGGTGTAACGATCTGGGCACTGTCTCAACCGCGATCTCGGTGAAATTGTAGTATCGGTGAAGATGCCGATTACCCGCAACGGGACGAAAAGACCCCGTGAACCTTTACTATAGCTTTACATTGTATTTGGGCATCAG 2116

*V natriegens* CGACCTGCACGAATGGCGTAATGATGGCCACGCTGTCTCCACCCGAGACTCAGTGAAATTGAAATCGCTGTGAAGATGCAGTGTACCCGCGGCTAGACGGAAAGACCCCGTGAACCTTTACTACAGCTTGGCACTGAACATTGACCCTGT 2011

Consensus ATGTGTAGGATAGGTGGGAGGCTTTGAAGCKGGRACGCYAGTTCBMGTGGAGCCRACCTTGAAATACCACCCTKGTATGKTTGAGGTTCTAACTCTGACCCGTAATCCGGGTCGAGGACAGTGTCTGGTGGGTAGTTTGACTGGGGCGGT 2255

*A baumannii* TTGTGTAGGATAGGTGGGAGGCTTTGAAGCTGGAACGCTAGTTCCAGTGGAGCCGTCCTTGAAATACCACCCTGGTAATGTTGAGGTTCTAACTCTGTCCCGTGATCCGGGACGAGGACCGTGTCTGGTGGGTAGTTTGACTGGGGCGGT 2246

*B burgdorferi* ATGTGTAGGATAGGTGGGAGACTTTGAAGCTATCTCGTTAGGGGTAGTGGAGTCAATCTTGAAATACCACCCTTGTTTAATTAGGTTTCTAACTTATAG--------AAATATGAGGAGAGTGCCAGGTGGGTAGTTTGACTGGGGCGGT 2303

*C burnetii* CTGTGTAGGATAGGTGGGAGGCTTTGAAGCTGGAGCGCTAGCTCCAGTGGAGCCAACCTTGAAATACCACCCTGGCGTGTTTGAGGTTCTAACCCTGTCCCGTAATCCGGGTCGGGAACAGTGTATGGTGGGTAGTTTGACTGGGGCGGT 2275

*E coli* ATGTGTAGGATAGGTGGGAGGCTTTGAAGTGTGGACGCCAGTCTGCATGGAGCCGACCTTGAAATACCACCCTTTAATGTTTGATGTTCTAACGTTGACCCGTAATCCGGGTTGCGGACAGTGTCTGGTGGGTAGTTTGACTGGGGCGGT 2257

*P aeruginosa* TTGTGTAGGATAGGTGGGAGGCTTTGAAGCGTGGACGCCAGTTCGCGTGGAGCCATCCTTGAAATACCACCCTGGCATGCTTGAGGTTCTAACTCTGGTCCGTAATCCGGATCGAGGACAGTGTATGGTGGGCAGTTTGACTGGGGCGGT 2244

*P gingivalis* ATGTGTAGGATAGGCCGGAGGCAGAGAAGCGGGTACGCCAGTATTCGTGGAGTCGATGTTGAAATACGGCCCTTTTGATGTTTGGATACTAACTCGCG---------GCGTGCGAGGACAGTGTATGGTGGGTAGTTTGACTGGGGTGGT 2257

*V natriegens* G-------------------------------------------------------------------------------TTGATGTTCTAACGTTGACCCCTTATCGGGGTTGCGGACAGTGCCTGGTGGGTAGTTTGACTGGGGCGGT 2082

Consensus CTCCTCCTAAAGAGTAACGGAGGAGTACGAAGGTGCGCTCAGCCCGGTCGGAAATCGGGCGGTGAGTGTAAAGGCATAAGCGCGCTTGACTGCGAGACTGACAAGTCGAGCAGGTACGAAAGTAGGTCTTAGTGATCCGGTGGTTCTGWA 2405

*A baumannii* CTCCTCCTAAAGAGTAACGGAGGAGTACGAAGGTGCGCTCAGCGTGGTCGGAAATCACGCGTAGAGTATAAAGGCAAAAGCGCGCTTAACTGCGAGACCCACAAGTCGAGCAGGTACGAAAGTAGGTCTTAGTGATCCGGTGGTTCTGTA 2396

*B burgdorferi* CGCCTCCTAAAGAGTAACGGAGGTGCGCAAAGGTTACCTTAGAGTGGTTGGAAATCACTCTGTAAGTGTAAAGGCATAAGGTAGCTTAACTGTAAGACTGACAAGTCGAACAGATACGAAAGTAGGTCTTAGTGATCTGGCGGTGGCAAG 2453

*C burnetii* CTCCTCCAAAAGAGTAACGGAGGAGTACAAAGGTACCCTCAGCACGGTCGGAAATCGTGCATTGTGTGCAAAGGCATAAGGGTGCTTGACTGCGAGACTGACAAGTCGAGCAGGTACGAAAGTAGGTCTTAGTGATCCGGTGGTCCTTTA 2425

*E coli* CTCCTCCTAAAGAGTAACGGAGGAGCACGAAGGTTGGCTAATCCTGGTCGGACATCAGGAGGTTAGTGCAATGGCATAAGCCAGCTTGACTGCGAGCGTGACGGCGCGAGCAGGTGCGAAAGCAGGTCATAGTGATCCGGTGGTTCTGAA 2407

*P aeruginosa* CTCCTCCTAAAGAGTAACGGAGGAGTACGAAGGTGCGCTCAGACCGGTCGGAAATCGGTCGCAGAGTATAAAGGCAAAAGCGCGCTTGACTGCGAGACAGACACGTCGAGCAGGTACGAAAGTAGGTCTTAGTGATCCGGTGGTTCTGTA 2394

*P gingivalis* CGCCTCCAAAAGCGTAACGGAGGCTTCTAAAGGTACCCTCAGGCCGATTGGTAACCGGTCGCAGAGTGTAATGGCACAAGGGTGCTTGACTGGGAGACAAACAAGTCGCACAGGTAGGAAACTAGAGCATAGTGATCCGGTGGTTCCGCA 2407

*V natriegens* CTCCTCCCAAAGCGTAACGGAGGAGCACGAAGGTGGGCTAATCACGGTTC------GTGAGGTTAGTGCAATGGCATAAGCCCGCTTGACTGCGAGAATGACAATTCGAGCAGGTGCGAAAGCAGGTCATAGTGATCCGGTGGTTCTGAA 2226

Consensus TGGAAGGGCCATCGCTCAACGGATAAAAGGTACTCCGGGGATAACAGGCTGATACCGCCCAAGAGTTCATATCGACGGCGGTGTTTGGCACCTCGATGTCGGCTCATCACATCCTGGGGCTGAAGCAGGTCCCAAGGGTATGGCTGTTCG 2555

*A baumannii* TGGAAGGGCCATCGCTCAACGGATAAAAGGTACTCTGGGGATAACAGGCTGATACCGCCCAAGAGTTCATATCGACGGCGGTGTTTGGCACCTCGATGTCGGCTCATCTCATCCTGGGGCTGAAGCAGGTCCCAAGGGTATGGCTGTTCG 2546

*B burgdorferi* TGGAAGCGCCGTCACTTAACGAATAAAAGGTACTCCGGGGATAACAGGCTTATCCTTCCCAAGAGTTCACATCGACGGAAGGGTTTGGCACCTCGATGTCGGCTCATCGCATCCTAGGGCTGGAGCAGGTCCTAAGGGTATGGCTGTTCG 2603

*C burnetii* TGGAAGGGCCATCGCTCAACGGATAAAAGGTACTCCGGGGATAACAGGCTGATACCACCCAAGAGTTCATATCGACGGTGGTGTTTGGCACCTCGATGTCGGCTCATCACATCCTGGGGCTGTAGCCGGTCCCAAGGGTATGGCTGTTCG 2575

*E coli* TGGAAGGGCCATCGCTCAACGGATAAAAGGTACTCCGGGGATAACAGGCTGATACCGCCCAAGAGTTCATATCGACGGCGGTGTTTGGCACCTCGATGTCGGCTCATCACATCCTGGGGCTGAAGTAGGTCCCAAGGGTATGGCTGTTCG 2557

*P aeruginosa* TGGAAGGGCCATCGCTCAACGGATAAAAGGTACTCCGGGGATAACAGGCTGATACCGCCCAAGAGTTCATATCGACGGCGGTGTTTGGCACCTCGATGTCGGCTCATCACATCCTGGGGCTGAAGCCGGTCCCAAGGGTATGGCTGTTCG 2544

*P gingivalis* TGGAAGGGCCATCGCTCAAAGGATAAAAGGTACTCCGGGGATAACAGGCTGATCACTCCCAAGAGCTCATATCGACGGAGTGGTTTGGCACCTCGATGTCGGCTCGTCACATCCTGGGGCTGGAGAAGGTCCCAAGGGTTGGGCTGTTCG 2557

*V natriegens* TGGAAGGGCCATCGCTCAACGGATAAAAGGTACTCCGGGGATAACAGGCTGATACCGCCCAAGAGTTCATATCGACGGCGGTGTTTGGCACCTCGATGTCGGCTCATCACATCCTGGGGCTGAAGTCGGTCCCAAGGGTATGGCTGTTCG 2376

Consensus CCATTTAAAGTGGTACGCGAGCTGGGTTTAGAACGTCGTGAGACAGTTCGGTCCCTATCTGCCGTGGGCGTTGGAGATTTGAGAGGGGCTGCTCCTAGTACGAGAGGACCGGAGTGGACGAACCTCTGGTGTTCCGGTTGTCACGCCAGT 2705

*A baumannii* CCATTTAAAGAGGTACGCGAGCTGGGTTTAGAACGTCGTGAGACAGTTCGGTCCCTATCTACCGTGGGCGCTGGAAATTTGAGAGGATCTGCTCCTAGTACGAGAGGACCAGAGTGGACGAACCTCTGGTGTACCGGTTGTGACGCCAGT 2696

*B burgdorferi* CCATTTAAAGCGGTACGCGAGCTGGGTTCAGAACGTCGTGAGACAGTTTGGTCCCTATCTGCCACAAGCGTTGGATATTTGAGAGGAGCTATCTTTAGTACGAGAGGACCGAGATGGACGAACCTCTAGTGTGCCAGTTATTCTGCCAAG 2753

*C burnetii* CCATTTAAAGTGGTACGTGAGCTGGGTTTAGAACGTCGTGAGACAGTTCGGTCCCTATCTGCCGTGGGCGTTGGAGATTTGAGAGGAGCTGCTCCTAGTACGAGAGGACCGGAGTGGACGTACCTCTGGTGTTCCGGTTGTCACGCCAGT 2725

*E coli* CCATTTAAAGTGGTACGCGAGCTGGGTTTAGAACGTCGTGAGACAGTTCGGTCCCTATCTGCCGTGGGCGCTGGAGAACTGAGGGGGGCTGCTCCTAGTACGAGAGGACCGGAGTGGACGCATCACTGGTGTTCGGGTTGTCATGCCAAT 2707

*P aeruginosa* CCATTTAAAGTGGTACGCGAGCTGGGTTTAGAACGTCGTGAGACAGTTCGGTCCCTATCTGCCGTGGACGTTTGAGATTTGAGAGGGGCTGCTCCTAGTACGAGAGGACCGGAGTGGACGAACCTCTGGTGTTCCGGTTGTCACGCCAGT 2694

*P gingivalis* CCCATTAAAGTGGCACGCGAGCTGGGTTCAGAACGTCGTGAGACAGTTCGGTCTCTATCTGTTGTGGGCGCAGGAAATTTGCGAGGGTCTGACACTAGTACGAGAGGACCGTGTTGGACAGACCCCTGGTTTACCGGTTGTACCGCCAGG 2707

*V natriegens* CCATTTAAAGTGGTACGCGAGCTGGGTTTAGAACGTCGTGAGACAGTTCGGTCCCTATCTGCCGTGGGCGTTGGAGAATTGAAAGGGGCTGCTCCTAGTACGAGAGGACCGGAGTGGACGAACCTCTGGTGTTCGGGTTGTGTCGCCAGA 2526

Consensus GG-CATTGCCGGGTAGCTAAGTTCGGAARGGATAACCGCTGAAAGCATCTAAGCGGGAAGCCTGCCTCAAGATGAGATCTCCCTGA-ACTTTAAGTNCCCTAAAGGGTCGTTGAAGACTACGACGTTGATAGGCTGGGTGTGTAAGCGTA 2853

*A baumannii* CG-CATCGCCGGGTAGCTATGTTCGGAAGGGATAACCGCTGAAAGCATCTAAGCGGGAAGCCTACCTCAAGATAAGATTTCCCTAGGACTTTATGTCCTCTAAAGAGCCGTTCGAGACTAGGACGTTGATAGGTTGGATGTGGAAGCATA 2845

*B burgdorferi* GGTAAGTGCTGGGTAGCTACGTTCGGAAAGGATAACCGCTGAAAGCATCTAAGTGGGAAGCCTTCCTCAAGATGAGATATCCT-----------------TTAAGGGTCCTGGAAGAATACCAGGTTGATAGGTTAGAAGTGTAAGTATA 2886

*C burnetii* GG-CATTGCCGGGTAGCTAAGTACGGACGGGATAACCGCTGAAAGCATCTAAGCGGGAAGCCCCCCTCAAGATGAGATCTCCCGGA-CCTTTAAGGTCCCTAAAGATTCGTTGAAGACGACAACGTTGATAGGCAGGGTGTGGAAGCTCA 2873

*E coli* GG-CACTGCCCGGTAGCTAAATGCGGAAGAGATAAGTGCTGAAAGCATCTAAGCACGAAACTTGCCCCGAGATGAGTTCTCCCTGACTCCTTGAGAGTCCTGAAGGAACGTTGAAGACGACGACGTTGATAGGCCGGGTGTGTAAGCGCA 2856

*P aeruginosa* GG-CATTGCCGGGTAGCTATGTTCGGAAAAGATAACCGCTGAAAGCATCTAAGCGGGAAACTTGCCTCAAGATGAGATCTCACTGGGAACTTGATTCCCCTGAAGGGCCGTCGAAGACTACGACGTTGATAGGCTGGGTGTGTAAGCGTT 2843

*P gingivalis* TG-CACCGCCGGGTATCCACGTCTGGTAAGGATAAGTGCTGAAAGCATCTAAGCACGAAGCCGGCCTCAAGATAAGATTTCCATAA---------------ATAGGGTGGTTAAAGACTATGACCTTGATAGGCTGCAGGTGTATGATTG 2841

*V natriegens* CG-CATTGCCCGGTAGCTAAGTTCGGGATCGATAACCGCTGAAAGCATCTAAGCGGGAAGCGAGCCTTGAGATGAGTTCTCCCTGATACTTTAAGTATCCTAAAGGGTTGTCGTAGACTACGACGTTGATAGGCAGGGTGTGTAAGCGTT 2675

Consensus GTGATGCGTTAAGCTAACCAGTACTAATTGCCCGTGAGGCTTGACCA------------------T 2901

*A baumannii* GTGATATGTGAAGCTGACCAATACTAATTGCTCGTGAGGCTTGACTATACAACAC--------CCA 2903

*B burgdorferi* GCAATATATTAAGCTGACTAATACTAATTACCCGTATCTTTGGCCAT------------------A 2934

*C burnetii* GTAATGAGTGAAGCTAACCTGTACTAATTAATCGTGCGACTTGACTATGTAACCCTAAATGGTTTC 2939

*E coli* GCGATGCGTTGAGCTAACCGGTACTAATGAACCGTGAGGCTTAACCT------------------T 2904

*P aeruginosa* GTGAGGCGTTGAGCTAACCAGTACTAATTGCCCGTGAGGCTTGACCA------------------T 2891

*P gingivalis* GTAA-CAATTAAGCCGAGCAGTACTAATAGCCCGAAACTTTTGTGCA------------------T 2888

*V natriegens* GTGAGGCGTTGAGCTAACCTGTACTAATTGCCCGTGAGGCTTAAC--------------------C 2721
