# Supplementary material for: Cryo-EM reveals multiple mechanisms of ribosome inhibition by doxycycline
Source: Nat Commun. 2026 Jun 1;17:7049. doi: 10.1038/s41467-026-73421-5 (PMC13392368; doi:10.1038/s41467-026-73421-5)
Supplement: Supplementary file 5 — Transparent Peer Review file [file 41467_2026_73421_MOESM5_ESM.pdf]

nature portfolio

Peer Review File

Cryo-EM reveals multiple mechanisms of ribosome inhibition by doxycycline

Corresponding Author: Professor Nicholas Harmer

This file contains all reviewer reports in order by version, followed by all author rebuttals in order by version.

Version 0:

Reviewer comments:

Reviewer #1

(Remarks to the Author)
This manuscript provides the structures of doxycycline in complex with ribosomes from C. burnetii and E. coli while providing new mechanisms for translation inhibition by doxycycline. In solving these structures, the authors also identified ribosomes in complex with HPFcold and a novel peptide CLaSP. These structures are informative about diversity in translation. The manuscript is topical due to the rise of antimicrobial resistant pathogens and the need for novel antibiotics. I have some comments about the manuscript below:
Major Comments:
1. It is unclear how extra density from the C. burnetii 70S was identified as CBU_0020. Was the structure high enough resolution that the sequence could be determined or was another method used.
2. HPFcold makes several interactions with the C. burnetii ribosome, are any of these amino acids conserved, indicating that they are essential for HPFcold function?
3. The minimal inhibitory concentration experiments data explained on page 9 line 173 to 175 is missing. This should be included to be interpreted by the audience.
4. Are there any bacteria that lack of the HPFcold protein and do they have a different MIC to tetracycline antibiotics.
5. Why is it important that HPFcold would protect the anti-Shine Dalgarno from RNase access. Wouldn't the majority of the ribosome be sensitive to RNase?
6. Page 12 Line 227 to 229 "bL27 has been hypothesized to function in communication within the ribosome, a function CLaSP may also provide" communication of what information and how does it do this?
7. Are the nucleotides that doxycycline interacts with in the peptide exit tunnel conserved for both C. burnetii and E. coli. This is important because if these nucleotides can change their sequence then natural resistance may be found in nature.
8. Your structures were solved at a antibiotic concentration of 50 µM which for doxycycline is roughly 22 mg/mL. Since the MIC for doxycycline for C. brunetii is 0.015 µg/mL is there evidence that this binding would be observed at concentrations closer to the MIC?

Minor Comments:
1. The use of A-site and A site is incorrect throughout the manuscript, please go over where A-site is hyphenated and ensure that it needs to be. Same is true for the P and E sites.
2. Page 4 line 59 – "At the A-site the tRNA anti-codon pairs to the mRNA". Anti-codon pairs is unclear.
3. Page 4 line 59-60 – "Amino acids are moved from the tRNA to the nascent chain at the P-site" The nascent chain is added to the amino acid of the A site. Clarification would help.
4. There are several areas of the manuscript where the details on translation are vague although they are well defined. For example Page 4 Line 61 "tRNAs move to the E-site and leave the ribosome". This could be clarified and elaborated on.
5. Page 5 line 78 – "Focused classification was used to fully elucidate antibiotic behavior". It is unclear what behavior is being discussed.
6. Page 5 line 92 – post translation modifications are listed but this should be post-transcriptional modifications. This is in the supplemental as well.
7. Page 7 line 125 – "CLaSP appears to stabilize the ribosome by inserting between the r23S and r5S, anchoring the two (Fig. 3c-d). What is meant by stabilize, is this region not stable in other species?
8. Coxiellaceae spelling varies throughout the manuscript.
9. Sometimes the 5S and 23S rRNA are referred to r5S and r23S.
10. Page 12 Line 224-226: "The role of CLaSP remains unclear, since the r5S rRNA of C. burnetii is truncated compared to

other bacteria (Fig. S5a), suggesting a role in ribosome assembly and r23S and r5S stability. Figure S5a does not show the r5S rRNA, Fig S6a does but it just shows the helix as being shorter, not necessarily as truncated.
11. Throughout the discussion the supplemental figures are called incorrectly.

Reviewer #2

(Remarks to the Author)
The study titled "Cryo-EM reveals multiple mechanisms of ribosome inhibition by doxycycline," by Stuart et al. reports high-resolution cryo-EM structures of the E. coli and Coxiella burnetii 70S ribosomes complexed with doxycycline. Beyond the canonical 30S A-site binding, doxycycline binds the 50S subunit exit tunnel in both species. In C. burnetii, a stack of three doxycycline molecules blocks the exit tunnel. In E. coli, doxycycline binding triggers a major, previously uncharacterized rearrangement of the peptidyl transferase center (PTC), essentially "zippering" the RNA into an inactive state. The work further reports a new hibernation factor, HPFcold (containing a cold shock domain), and a Coxiellaceae-specific peptide, CLaSP, which is proposed to stabilize the large subunit.

This work is significant as it challenges the long-standing view that tetracyclines primarily inhibit translation by blocking the A site (together with a recent bioRxiv manuscript, see below). The discovery of a 50S-targeted mechanism explains the "exquisite" sensitivity of C. burnetii to doxycycline (MIC 0.01-0.04ug/ml) compared to E. coli (~8 ug/ml). Furthermore, the observation of a large-scale PTC rearrangement in E. coli suggests that this highly conserved region is more dynamic than previously thought. The quality of the EM maps presented is excellent, achieving resolutions as high as 1.77 Å for the E. coli 70S and 2.16 Å for the rearranged state. The use of focused classification was essential to resolve the relatively small subpopulation (~12%) of ribosomes in the rearranged state, which might have been overlooked in previous studies. The authors effectively used MIC experiments with various tetracycline derivatives (tetracycline, doxycycline, tigecycline, eravacycline) to support their structural findings. They demonstrate that bulkier later-generation tetracyclines, which cannot fit into the 50S stack, are less potent against C. burnetii, validating the importance of the exit tunnel site.

It would be advisable to perform mass-spectrometry on the samples, such that the identity of the newly identified CLaSP peptide could be confirmed – not only relying on the EM maps.

In the section "Doxycycline binds to both large and small ribosomal subunits" (line 75), the authors should cite a new study deposited in bioRxiv (https://www.biorxiv.org/content/10.1101/2025.09.25.678686v1), which also reports binding of DOX (stacked conformation) in the peptide exit tunnel. This should also be presented in the discussion section, as it is relevant to the current study. This paper should again be cited around line 146.

Around line 107, the authors should refer to a review paper deposited in bioRxiv (https://www.biorxiv.org/content/10.1101/2025.11.04.686544v2), in which HPFcold was presented (e.g. see figure 6 in this paper).

Overall, this study advances our understanding of antibiotic action and ribosome biology. By combining high-resolution structural biology with microbiological data, the authors provide a compelling explanation for species-specific drug sensitivity and reveal unexpected flexibility in the ribosome's catalytic core. The paper is well-written, and the findings provide a roadmap for designing more potent, potentially bactericidal, tetracycline derivatives that target the 50S subunit.

Minor

In models 9T2Y (Dox70S_Cb) and 9T1N (Dox50S_triple_Cb), U2629 proximal to DOX is not properly modeled into the density. Density indicates it should base pair with A759. Furthermore, nucleotide numbering in 9T1N (Dox50S_triple_Cb) is not the same as in the 50S subunit of 9T2Y (Dox70S_Cb). rRNA nucleotide numbering in 9T1N should be fixed.

In the 9T1N map, there is additional density that has not been modeled, located adjacent to helix 21 (e.g., residue G385) and helix 9 (e.g., residue C205) of the 23S rRNA.

Supplementary Table 2 (Data collection and refinement parameters) is difficult to read due to the very small font size and confusing column headings. The table should be generated in, for instance, Word, and not as an image. Several fields could be removed to improve clarity and conciseness, and PDB and EMDB accession codes should be provided at the top of each column. More importantly, there are substantial inconsistencies in the naming and annotation of deposited maps and models. Multiple files labeled as 30S or 50S correspond to full 70S maps (e.g., 9SLG is labeled Dox50S_Ec, although it corresponds to a 70S map; 9T0Z is a 70S map but is named as 9T0Z_HPFcold_30S_Cb_map.mrc; 9SLG_Dox50S_map.mrc, 9SX2_Dox30S_Ec_map.mrc, 9T61_Dox30S_Cb_map.mrc also corresponds to 70S maps), making it difficult to interpret the deposited data. In addition, models such as C. burnetii HPFcold appear to originate from the same 70S dataset but are presented as separate entries without clear justification. If local refinements were performed, the corresponding local and global maps should be deposited (and even composite map, if used to refine the model) together with the models and mentioned separately in the table. Finally, several model names (e.g., "E. coli Rearrange" and other mentioned above) are unclear and non-standard. Sharpening B-factors for the maps should also be indicated in the table.

Line 157, The identity of nucleotide 2082 is not U, but A

Line 192, The identity of nucleotide 2506 is not C, but U. Similarly, 2063 is a C, not U.

Line 198, Wrong nomenclature, this is uL4, not bL4. The authors should pay close attention to the r-protein nomenclature, nucleotide identity and numbering. Same for their Supplementary Table 1. For the now accepted nomenclature, please see Ban N et al. Curr. Opin. Struct. Biol. (2014), 165-169 or https://bangroup.ethz.ch/research/nomenclature-of-ribosomal-proteins.html

Line 215, Here and throughout the text, r16S is not the usual nomenclature. It should be 16S rRNA, not r16S rRNA. Same comment at line 224, 23S and 5S rRNAs.

Lines 226-227, “The position of CLaSP overlaps that of the…” overlaps with that…

Line 235, The authors mean reduced PTMs, but this term can bring confusion as it could also refer to reduced (shorter) rRNA forms.

Line 243, U2506 is E. coli numbering, but the authors discuss C. burnetii ribosome here. E. coli number should instead be in parentheses.

Lines 276 and 278, bL4 should be uS4.

Figure 5e: The label should read “A-site” instead of “a-site.”

Line 59, Page 4: anti-codon (should be “anticodon”)

Line 352: 1.07m (should be typed as “million” particles)

Lines 355-365: 655k…306k, 372 k (should be typed as 655,000 particles)

Reviewer #3

(Remarks to the Author)
Stuart et al. shown a comprehensive cryo-EM analysis of doxycycline bound ribosomes from C. burnetii and E. coli, revealing unexpected antibiotic binding modes in the large ribosomal subunit. The identification of a stacked triple-Dox conformation blocking the nascent peptide exit tunnel in C. burnetii, as well as a striking Dox induced rearrangement of PTC and PET in E. coli, is a beautiful conceptual advance in our understanding of tetracyclines action. In addition, the author also discovered a new ribosomal protein (CLaSP) and a new hibernation factor (HPFcold) in C. burnetii ribosome.
In general, this work is technically sound, with exceptionally high resolution cryo-EM maps, and careful model building. Overall, the conclusions are largely supported by the data, however, I have few suggestions which may improve this manuscript.

Major concern:
1. According to the authors, the daily clinical dose of Dox is 200 mg, which should correspond to a peak plasma concentration below ~10 μM. However, the structures were solved using 50 μM doxycycline (this is understandable for achieving high occupancy). This raises the possibility that the additional binding site, including the stacked binding mode, may be induced by unphysiological concentrations that are not achieved during standard treatment. While I understand that higher local concentrations may occur in certain tissues or under in vitro conditions, I would be more suggest to use physiologically relevant concentrations. Additionally, please clearly state the Dox concentration used in the main text and in the relevant figure legends.
2. Several statements in the manuscript used a direct causal relationship between the observed structural states and Dox MIC values, which is not sufficiently supported by the data. MIC is influenced by many factors, including uptake, efflux, solubility, metabolism, and ribosome binding at both the 30S and 50S subunits.
For example, the statement in Line 255 that “binding in the large subunit … is the major determinant of the Dox MIC in this pathogen” is not supported experimentally and is likely incorrect. Eravacycline can also bind the exit tunnel, and despite the dramatic PTC rearrangements induced by Dox, it does not strongly inhibit E. coli. The triple-stacked binding mode in C. burnetii may therefore not necessarily correlate with higher inhibitory efficiency and could even represent a less efficient or non-physiological state induced by high Dox concentrations.
Throughout the manuscript, strong statements such as “explains,” “major determinant,” or “accounts for” should be softened to more appropriate language.

Suggestions:
1. In my view, the central advance of this study is the identification of a new mechanism by which Dox inhibits the ribosome. I would therefore strongly recommend restructuring the manuscript such that the Dox inhibition mechanism is presented first and more prominently, followed by the description of the new hibernation factor and ribosomal protein. At present, the flow of the story is difficult to follow, and the most conceptually important findings appear too late in the manuscript.
2. In the Abstract, the statement “challenging the concept that this region is largely static” is misleading. It is well known that antibiotics, nascent polypeptide chains, and even peptide bond formation itself can remodel the peptidyl transferase center (PTC) to some extent. This sentence should therefore be changed.
3. Line 92: “There are notably fewer rRNA post-translational protein modifications (PTMs)” is confusing. Please clarify whether this refers to rRNA modifications or ribosomal protein modifications.

4. Line 101: “mRNA entrance tunnel” should be “exit tunnel.”
5. The abbreviation “r16S” is used repeatedly in this study. However, this is not a standard abbreviation for 16S rRNA and should be changed.
6. Line 144: The term “incomplete binding” is not appropriate and should be changed to a more accurate description. Moreover, this sentence disrupts the logic of the paragraph, as E. coli structures have not yet been introduced at this point. I would suggest to remove it.
7. Line 168 states “To test this hypothesis”, referring to the idea that lack of additional chemical elaboration may facilitate this stacked binding mode. However, the following MIC experiments do not directly support this hypothesis, as MIC values are influenced by many non-ribosomal reasons.
8. Line 242: “To date, no antibiotic has been observed to block the exit tunnel so extensively” does not add any meaningful information and should be removed.
9. Cryo-EM particle numbers are consistently reported as approximate values (such as 417k particles). The exact numbers must be used.
10. Supplementary Figure 9. HPFcold is not present in the E. coli sample.
11. Figure citations should be checked carefully. For example, the main text (Line 244) cites Fig. S8 in a context where it does not appear relevant, the Supplementary Discussion uses Fig. S8e as support for the claim about A2503 C8 methylation and C. burnetii doxycycline binding. Since Fig. S8 is a processing pipeline, it does not seem to contain the needed structural evidence. Moreover, Line 225/227 should likely cite Fig. S6a/S6b (rather than Fig. S5a/5b); Line 246 “Fig. S9” should be Fig. S10 and Line 276 “Fig. S10b” should be Fig. S11b. Maybe even more?
12. Please consider converting Supplementary Table 2 into a standard Nature format table.

Reviewer #4

(Remarks to the Author)
I co-reviewed this manuscript with one of the reviewers who provided the listed reports. This is part of the Nature Communications initiative to facilitate training in peer review and to provide appropriate recognition for Early Career Researchers who co-review manuscripts.

Version 1:

Reviewer comments:

Reviewer #1

(Remarks to the Author)
All comments and concerns that I had about the manuscript have been addressed. The authors provided additional figures that have strengthened their arguments that the doxycycline trimer in the peptide exit tunnel is functionally relevant by justifying the concentration used and characterizing the conservation of the peptide exit tunnel nucleotides. Furthermore, they have characterize the conservation of HPFcold residues that interact with the anti-Shine-Dalgarno sequence, strengthening the article.
I do have a few minor comments that have arisen since submission of the manuscript. These include:
1. In the abstract “cryo electron-microscopy” is hyphenated incorrectly, it should be cryo-electron microscopy.
2. Page 2 line 59 – “anti-Shine Dalgarno” should be “anti-Shine-Dalgarno”
3. Page 11 line 213-215 has a sentence “Other key rearrangements include A2062 ‘flipping in’, away from the PTC to base pair with dihydrouridine 2449 and, via a non-canonical bae pair, with A2450.” This is interesting however, I can not see this interaction in the figures.
4. Supplemental discussion. “Gram-negatives” should be “Gram-negative bacteria”

Reviewer #2

(Remarks to the Author)
The authors have appropriately addressed my initial concerns in the revised manuscript. Despite the absence of mass spectrometry-based identification of the CLaSP peptide, the electron density map is indeed of high-enough resolution to confidently confirm its identity.

In Supplementary Table 2, the standard practice is to report model resolution at a FSC cutoff of 0.5, not 0.143. The sharpening B factor should be provided for each deposited map. Finally, for Rms deviations, what are the values reported in parentheses?

Reviewer #3

(Remarks to the Author)
The authors have addressed most of the points raised in the first round, and the revised manuscript is clearly improved.

There is one typo: In Supplementary Figure 15b, the protein is labeled as bL4, whereas the main text consistently refers to the same ribosomal protein as uL4.

Reviewer #4

(Remarks to the Author)
I co-reviewed this manuscript with one of the reviewers who provided the listed reports. This is part of the Nature Communications initiative to facilitate training in peer review and to provide appropriate recognition for Early Career Researchers who co-review manuscripts.

Open Access This Peer Review File is licensed under a Creative Commons Attribution 4.0 International License, which permits use, sharing, adaptation, distribution and reproduction in any medium or format, as long as you give appropriate credit to the original author(s) and the source, provide a link to the Creative Commons license, and indicate if changes were made.
In cases where reviewers are anonymous, credit should be given to 'Anonymous Referee' and the source.
The images or other third party material in this Peer Review File are included in the article's Creative Commons license, unless indicated otherwise in a credit line to the material. If material is not included in the article's Creative Commons license and your intended use is not permitted by statutory regulation or exceeds the permitted use, you will need to obtain permission directly from the copyright holder.
To view a copy of this license, visit https://creativecommons.org/licenses/by/4.0/

Response to reviewers

Stuart et al., “Cryo-EM reveals multiple mechanisms of ribosome inhibition by doxycycline”

We thank the reviewers for their insightful and detailed comments on our manuscript. All the reviews provided excellent suggestions for improving our work. We have amended the manuscript as far as was practicable in the permitted time to address these suggestions. We detail our response below (reviewer comments in black, our response in green).

Reviewer #1 (Remarks to the Author)

This manuscript provides the structures of doxycycline in complex with ribosomes from C. burnetii and E. coli while providing new mechanisms for translation inhibition by doxycycline. In solving these structures, the authors also identified ribosomes in complex with HPFcold and a novel peptide CLaSP. These structures are informative about diversity in translation. The manuscript is topical due to the rise of antimicrobial resistant pathogens and the need for novel antibiotics. I have some comments about the manuscript below:
Major Comments:
1. It is unclear how extra density from the C. burnetii 70S was identified as CBU_0020. Was the structure high enough resolution that the sequence could be determined or was another method used.

The resolution of the cryo-EM reconstruction was sufficiently high to identify part of the sequence of of the hibernation factor directly, allowing unambiguous assignment of this protein as CBU_0020. Furthermore, there was additional evidence to support assignment of CBU_0020 to the density. Since the structure showed the presence of an HPF fold protein bound, this narrowed the choice down to only two options in the genome, CBU_0020 or CBU_0745. CBU_0745 is significantly shorter than CBU_0020, lacking the characteristic C-terminal CSD. The choice of CBU_0020 was confirmed by also attempting to model CBU_0745 into the density which showed sidechain disagreement and failed to match the density for the larger loop between strands ß2 and ß3 that CBU_0020 displays. We have clarified this in the methods.

2. HPFcold makes several interactions with the C. burnetii ribosome, are any of these amino acids conserved, indicating that they are essential for HPFcold function?

This is an excellent question. We have carried out further analysis of the conservation of HPF_{cold}. Briefly, the CSD displays a higher average conservation than the sigma-54 like domain. Most of the well conserved residues are structural (as might be expected). However, the CSD shows a striking patch of conserved surface exposed residues at the interface with the anti-Shine-Dalgarno rRNA sequence. This

is significantly more conserved than any other surface face of HPF_{cold}. This includes other patches of rRNA interacting residues, where conservation is largely limited to residues making charge-charge interactions with backbone phosphates or magnesium ions. This suggests that the aSD interaction is the major functional role of HPF_{cold}. Stabilisation of the ribosome appears to be secondary and variation may arise due to changes in ribosome structure between species. We have added this analysis as Figures S6 and S7 and further detail in the results section.

3. The minimal inhibitory concentrations data explained on page 9 line 173 to 175 is missing. This should be included to be interpreted by the audience.

We apologise for this oversight and have added these data in Supplementary Figure 14, which is noted at the appropriate point in the text. We followed DSTL's protocols for determining *Coxiella* MIC values to maintain compatibility with previous studies. These require that samples are determined as growing or not by eye (as plate readers are not usually available at high containment). We have highlighted which antimicrobial concentrations showed visible growth for all our replicates.

4. Are there any bacteria that lack of the HPFcold protein and do they have a different MIC to tetracycline antibiotics.

The majority of bacteria do not have an HPF_{cold} orthologue (as is discussed in detail in the helpful recent article that reviewer 2 notes). However, many have one of the other types of HPF that are noted in the recent publication on HPFs that reviewer 2 highlighted. While the 30S binding site of tetracyclines overlaps with HPFs and binding is mutually exclusive, to the best of our knowledge the effect of HPF mutants on tetracycline efficacy has not been tested. The aminoglycoside binding site also overlaps with HPF and it has been shown that *E. coli* strains with HPF mutants display higher sensitivity (doi: 10.1128/AAC.01532-15), not due to direct competition for binding but by general ribosome hibernation. *Legionella pneumophila* utilises HPF_{cold} but does not show such particular sensitivity towards doxycycline (doi: 10.1016/j.diagmicrobio.2011.09.016). Therefore, we suspect HPF_{cold} may not be a major determinant of doxycycline sensitivity. We have incorporated this into the supplementary discussion. There is also no evidence for variation between strains of *C. burnetii*, given the virulent NMI strain reporting consistent MIC values with NMII.

5. Why is it important that HPFcold would protect the anti-Shine Dalgarno from RNase access. Wouldn't the majority of the ribosome be sensitive to RNase?

This is a good question that we have elaborated further on in our revision in the discussion (lines 237-240). The most salient point is that all other HPFs deploy at least one strategy to protect the anti-Shine-Dalgarno sequence, and that disruption of this function has been shown to reduce bacterial viability. This is not unexpected

as the aSD sequence is a particularly sensitive part of the ribosome: it is a terminal sequence (so at risk from both endo and exo-nucleases), is surface exposed (especially in the free 30S ribosome), and is critical to function of the bacterial ribosome. Many other HPFs protect the aSD sequence by dimerising ribosomes. As HPFcold does not support dimerisation, another mechanism must be invoked to explain a protective effect on the aSD sequence.

6. Page 12 Line 227 to 229 “bL27 has been hypothesized to function in communication within the ribosome, a function CLaSP may also provide” communication of what information and how does it do this?

We have altered this language and removed reference to communication within the ribosome. We suspect CLaSP is a case of convergent evolution to the bL37 gene from *Mycobacteria*, given the similar interactions made. bL37 is thought to contribute to general stability (doi: 10.1016/j.celrep.2017.06.029). Given CLaSP’s proximity within the genome to *nusB* we hypothesise that CLaSP is likely supportive of ribosome biogenesis through stabilisation, but this will be a topic for future investigation.

7. Are the nucleotides that doxycycline interacts with in the peptide exit tunnel conserved for both C. burnetii and E. coli. This is important because if these nucleotides can change their sequence then natural resistance may be found in nature.

This is a good question that poses two interesting directions. We have aligned the sequences of all currently available 23S rRNA sequences from *C. burnetii* genomes. These confirm that all nucleotides within 4 Å of the three 50S doxycycline molecules are conserved in all such *C. burnetii* sequences. We also aligned the *Coxiella* 23S rRNA consensus to example sequences from other Hydrobacteria with solved ribosome structures. All nucleotides within 4 Å of the 50S doxycycline molecules are conserved within the proteobacteria (e.g. *E. coli*) with solved sequences; whilst there are a couple of changes in the more distantly related organisms, the modified bases are conserved in space and the changes would not affect doxycycline binding to the ribosome. We have included this analysis in a new supplementary figure (Fig. S12). and noted this at the appropriate point in the results section.

8. Your structures were solved at a antibiotic concentration of 50 μM which for doxycycline is roughly 22 mg/mL. Since the MIC for doxycycline for C. brunetii is 0.015 μg/mL is there evidence that this binding would be observed at concentrations closer to the MIC?

This is an important question, also raised by Reviewer 3 – what is the most appropriate concentration of doxycycline for this study?

Briefly, we have modified the text to address the question. We have softened our language to make clear that we cannot prove that this is the dominant inhibitory mechanism for inhibition and may not directly explain *Coxiella's* sensitivity to doxycycline. Nevertheless, our results are clearly suggestive that this is important for the effect on MIC.

We chose 50 μM as this reflects the higher end of physiological serum conditions. Observed plasma levels of doxycycline in humans under preferred treatment reach 8 mg/L (doi:10.1128/aac.02401-16), whilst diagnostic firms consider 30 mg/L to be a dangerous level indicating overdosing (e.g. https://testdirectory.questdiagnostics.com/test/test-detail/94093/doxycyline?cc=MASTER). 30 mg/L doxycycline is 67.5 μM, a little higher than the concentration that we chose. Performing cryo-EM at the MIC would have been unlikely to result in a high quality structure of the ribosome-doxycycline complex, as to get good cryo-EM results we required ribosome preparations at high nanomolar concentrations, so doxycycline at the MIC would have been significantly less than stoichiometric.

A significant challenge is that it is not clear what doxycycline concentration the ribosome experiences on antimicrobial treatment in the cell. The best test would be to perform cryo-electron tomography on *Coxiella* treated with doxycycline at the MIC. And within the last year we had made a significant effort to carry out such an experiment. Unfortunately, this was not possible as there are no tomography facilities in the UK that have sufficient security to handle *C. burnetii* within the requirements of UK anti-terror laws, thus the experiment would be highly challenging and an entirely separate study in its own right.

Minor Comments:

1. The use of A-site and A site is incorrect throughout the manuscript, please go over where A-site is hyphenated and ensure that it needs to be. Same is true for the P and E sites.

We have amended this throughout the manuscript.

2. Page 4 line 59 – "At the A-site the tRNA anti-codon pairs to the mRNA". Anti-codon pairs is unclear.

We have clarified this sentence to be clearer about our meaning. The revised sentence reads: "In a translating ribosome, at the A site the aminoacylated tRNA is accepted and codon-anticodon base pairing occurs with the mRNA."

3. Page 4 line 59-60 – "Amino acids are moved from the tRNA to the nascent chain at the P-site" The nascent chain is added to the amino acid of the A site. Clarification would help.

We thank the reviewer for this suggestion. We have clarified this paragraph detailed in response to the next comment (4).

4. There are several areas of the manuscript where the details on translation are vague although they are well defined. For example Page 4 Line 61 “tRNAs move to the E-site and leave the ribosome”. This could be clarified and elaborated on.

We have checked through the manuscript carefully and been more precise on the details of translation that are relevant to the results of the paper. Specifically we have updated the paragraph at the start of Page 4 (Lines 56-66) to clarify details as follows: “Ribosomes have three binding sites for tRNA molecules: the A, P and E sites. In prokaryotes, the translation initiation site is determined by the mRNA Shine-Dalgarno sequence pairing with the 16S rRNA anti-Shine Dalgarno sequence in the 30S subunit. After formation of the translationally competent 70S ribosome, at the A site decoding centre, aminoacylated tRNA is decoded by codon-anticodon base pairing with the mRNA. Peptide bond formation is catalysed by the peptidyl transferase centre (50S subunit), where the growing nascent polypeptide chain is transferred from the P-site tRNA to the amino group of the aminoacylated A-site tRNA. The extending polypeptide proceeds through the nascent chain exit tunnel in the large subunit. Translocation occurs, moving the mRNA and tRNAs to the next site (from A-site to P-site, or P-site to E-site).”

5. Page 5 line 78 – “Focused classification was used to fully elucidate antibiotic behavior”. It is unclear what behavior is being discussed.

We have replaced this sentence with the following (“Both datasets showed doxycycline bound in the expected (…)”) to improve the logical flow of this paragraph and altered this sentence to: “Focused classification was used to further interrogate doxycycline binding behaviour beyond the canonical site.”

6. Page 5 line 92 – post translation modifications are listed but this should be post-transcriptional modifications. This is in the supplemental as well.

We have corrected this throughout the main and supplementary text.

7. Page 7 line 125 – “CLaSP appears to stabilize the ribosome by inserting between the r23S and r5S, anchoring the two (Fig. 3c-d). What is meant by stabilize, is this region not stable in other species?

We have re-worded this to focus on the factual point: “CLaSP connects the shorter *C. burnetii* 5S rRNA to the 23S rRNA, providing a direct contact between the rRNA molecules (Fig. 3c-d, Fig. S8).” (Page 7, Line 140-1). In discussion, we have added

to the points made in response to major point 6 that we *speculate* that CLaSP may be stabilising the ribosome by bridging these two rRNA molecules.

8. Coxiellaceae spelling varies throughout the manuscript.

We thank the reviewer for pointing this out. We have carefully checked the manuscript and ensured the correct spelling throughout.

9. Sometimes the 5S and 23S rRNA are referred to r5S and r23S.

We have altered all reference to r5S and r23S to 5S and 23S throughout the manuscript, also following notes from reviewer 2.

10. Page 12 Line 224-226: “The role of CLaSP remains unclear, since the r5S rRNA of C. burnetii is truncated compared to other bacteria (Fig. S5a), suggesting a role in ribosome assembly and r23S and r5S stability. Figure S5a does not show the r5S rRNA, Fig S6a does but it just shows the helix as being shorter, not necessarily as truncated.

We apologise for the error and have corrected the figure call out. Aligning the 5S rRNA structures, there is a reduction of two bases in the D-loop region of the *C. burnetii* ribosome compared to E. coli (Fig. S8). This results in reduced protrusion of the 5S into the cleft. We have updated this sentence (Page 13, Lines 248-9) to the following: “The 5S rRNA of *C. burnetii* shows a more compact conformation and therefore reduced protrusion into this cleft compared to *E. coli* (Fig. S8a).”

11. Throughout the discussion the supplemental figures are called incorrectly.

We apologise to the reviewers for these errors. We have carefully checked the manuscript in revision to ensure that all reference to supplemental figures are correct.

Reviewer #2 (Remarks to the Author):

The study titled "Cryo-EM reveals multiple mechanisms of ribosome inhibition by doxycycline," by Stuart et al. reports high-resolution cryo-EM structures of the E. coli and Coxiella burnetii 70S ribosomes complexed with doxycycline. Beyond the canonical 30S A-site binding, doxycycline binds the 50S subunit exit tunnel in both species. In C. burnetii, a stack of three doxycycline molecules blocks the exit tunnel. In E. coli, doxycycline binding triggers a major, previously uncharacterized rearrangement of the peptidyl transferase center (PTC), essentially "zippering" the RNA into an inactive state. The work further reports a new hibernation factor, HPFcold (containing a cold shock domain), and a Coxiellaceae-specific peptide, CLaSP, which is proposed to stabilize the large subunit.

This work is significant as it challenges the long-standing view that tetracyclines primarily inhibit translation by blocking the A site (together with a recent bioRxiv manuscript, see below). The discovery of a 50S-targeted mechanism explains the "exquisite" sensitivity of C. burnetii to doxycycline (MIC 0.01-0.04ug/ml) compared to E. coli (~8 ug/ml). Furthermore, the observation of a large-scale PTC rearrangement in E. coli suggests that this highly conserved region is more dynamic than previously thought. The quality of the EM maps presented is excellent, achieving resolutions as high as 1.77 Å for the E. coli 70S and 2.16 Å for the rearranged state. The use of focused classification was essential to resolve the relatively small subpopulation (~12%) of ribosomes in the rearranged state, which might have been overlooked in previous studies. The authors effectively used MIC experiments with various tetracycline derivatives (tetracycline, doxycycline, tigecycline, eravacycline) to support their structural findings. They demonstrate that bulkier later-generation tetracyclines, which cannot fit into the 50S stack, are less potent against C. burnetii, validating the importance of the exit tunnel site.

We thank the reviewer for their supportive comments.

It would be advisable to perform mass-spectrometry on the samples, such that the identity of the newly identified CLaSP peptide could be confirmed – not only relying on the EM maps.

Before the initial submission, we attempted mass spectrometry (of chymotryptic digests of the lower part of an SDS-PAGE gel run on purified *C. burnetii* ribosomes, as recommended by our local MS facility). We were specifically looking for the CLaSP peptide (with a view to confirming whether there are additional amino acids beyond those that can be resolved in the cryo-EM density). Unfortunately, we were not able to detect any peptides consistent with CLaSP. This likely reflects the comparatively low yields of ribosomes that could be recovered from *C. burnetii.* The CLaSP sequence contains many positively charged residues, making the usual workflow with trypsin impossible. Although we attempted analysis using chymotrypsin, there is only one potential chymotrypsin target in CLaSP, and the sequence may obviate chymotrypsin activity at this site. These features would explain the failure to observe CLaSP in MS analysis. We have amended the methods section to include our attempt to identify the full length expressed CLaSP peptide sequence. Given the high quality of the cryo-EM reconstruction and the conservation of this ORF throughout *Coxiellaceae,* we are confident in the

identification of CLaSP is secure. We have inserted example images of the CLaSP map quality below:

In the section “Doxycycline binds to both large and small ribosomal subunits” (line 75), the authors should cite a new study deposited in bioRxiv (https://www.biorxiv.org/content/10.1101/2025.09.25.678686v1), which also reports binding of DOX (stacked conformation) in the peptide exit tunnel. This should also be presented in the discussion section, as it is relevant to the current study. This paper

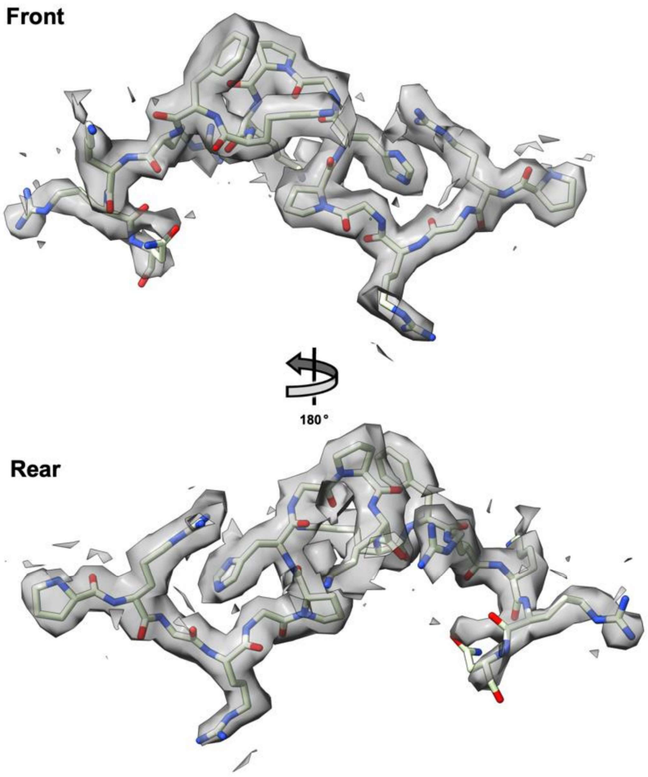

should again be cited around line 146.

We thank the reviewer for pointing out this study. We have noted its findings in lines 90, 160 and discussed in lines 276-280.

Around line 107, the authors should refer to a review paper deposited in bioRxiv (https://www.biorxiv.org/content/10.1101/2025.11.04.686544v2), in which HPFcold was presented (e.g. see figure 6 in this paper).

We thank the reviewer for highlighting this very useful study. We have referred to this on lines 108-9 and 226-8.

Overall, this study advances our understanding of antibiotic action and ribosome biology. By combining high-resolution structural biology with microbiological data, the

authors provide a compelling explanation for species-specific drug sensitivity and reveal unexpected flexibility in the ribosome's catalytic core. The paper is well-written, and the findings provide a roadmap for designing more potent, potentially bactericidal, tetracycline derivatives that target the 50S subunit.

Minor

In models 9T2Y (Dox70S_Cb) and 9T1N (Dox50S_triple_Cb), U2629 proximal to DOX is not properly modeled into the density. Density indicates it should base pair with A759. Furthermore, nucleotide numbering in 9T1N (Dox50S_triple_Cb) is not the same as in the 50S subunit of 9T2Y (Dox70S_Cb). rRNA nucleotide numbering in 9T1N should be fixed.

We thank the referee for their thorough analysis of our maps and models. We agree with the comment and have updated the doxycycline bound models to reflect the multiple occupancies present for U2629. We have also addressed the numbering in the 70S *C. burnetii* model.

In the 9T1N map, there is additional density that has not been modeled, located adjacent to helix 21 (e.g., residue G385) and helix 9 (e.g., residue C205) of the 23S rRNA.

We apologise for this error, at some point late in the refinement process several ribosomal proteins were lost (L23, L27 and L28). We have reinstated these in all large subunit models. The updated model PDB codes are as follows:

- 9TZE: *C. burnetii* 70S with 50S doxycycline and 30S HPF_{cold}
- 9TZ5: *C. burnetii* 50S with doxycycline
- 9TZ9: *C. burnetii* 50S empty

Supplementary Table 2 (Data collection and refinement parameters) is difficult to read due to the very small font size and confusing column headings. The table should be generated in, for instance, Word, and not as an image. Several fields could be removed to improve clarity and conciseness, and PDB and EMDB accession codes should be provided at the top of each column. More importantly, there are substantial inconsistencies in the naming and annotation of deposited maps and models. Multiple files labeled as 30S or 50S correspond to full 70S maps (e.g., 9SLG is labeled Dox50S_Ec, although it corresponds to a 70S map; 9T0Z is a 70S map but is named as 9T0Z_HPFcold_30S_Cb_map.mrc; 9SLG_Dox50S_map.mrc, 9SX2_Dox30S_Ec_map.mrc, 9T61_Dox30S_Cb_map.mrc also corresponds to 70S maps), making it difficult to interpret the deposited data. In addition, models such as C. burnetii HPFcold appear to originate from the same 70S dataset but are presented as separate entries without clear justification. If local refinements were performed, the corresponding local and

global maps should be deposited (and even composite map, if used to refine the model) together with the models and mentioned separately in the table. Finally, several model names (e.g., "E. coli Rearrange" and other mentioned above) are unclear and non-standard. Sharpening B-factors for the maps should also be indicated in the table.

We apologise for the unclear naming and have updated the files to reflect this. We have also clarified this in the data availability section (lines 530-7). In line with this suggestion and Reviewer 3, we have reformatted Table S2 into a Nature format. The *C. burnetii* 30S HPF_{cold} and doxycycline models utilised classified particles and locally refined maps and these data have been deposited separately.

Proposed new names:

- *E. coli* 70S Ribosome (50S Bound Doxycycline) -> **Ec_70S_50S_doxycycline**
- *E. coli* 30S Ribosome Subunit (30S Bound Doxycycline) -> **Ec_30S_30S_doxycycline**
- *C. burnetii* 30S Ribosome Subunit (HPFcold Bound) -> **Cb_30S_30S_HPFcold**
- *C. burnetii* 30S Ribosome Subunit (30S Bound Doxycycline) -> **Cb_30S_30S_doxycycline**
- *C. burnetii* 70S Ribosome (50S Doxycycline & 30S HPFcold) -> **Cb_70S_50S_doxycycline_30S_HPFcold**
- *C. burnetii* 50S Ribosome Subunit (50S Bound Doxycycline) -> **Cb_50S_doxycycline**
- *C. burnetii* 50S Ribosome Subunit (Empty) -> **Cb_50S_empty**

Line 157, The identity of nucleotide 2082 is not U, but A

Thank you, we have corrected this.

Line 192, The identity of nucleotide 2506 is not C, but U. Similarly, 2063 is a C, not U.

Thank you, we have corrected this.

Line 198, Wrong nomenclature, this is uL4, not bL4. The authors should pay close attention to the r-protein nomenclature, nucleotide identity and numbering. Same for their Supplementary Table 1. For the now accepted nomenclature, please see Ban N et al. Curr. Opin. Struct. Biol. (2014), 165-169
or https://bangroup.ethz.ch/research/nomenclature-of-ribosomal-proteins.html

We thank the reviewer for noting the errors and for highlighting the useful paper providing the best nomenclature. We have amended the nomenclature throughout to follow this.

Line 215, Here and throughout the text, r16S is not the usual nomenclature. It should be 16S rRNA, not r16S rRNA. Same comment at line 224, 23S and 5S rRNAs.

We thank the reviewer for noting these points, which we have corrected.

Lines 226-227, "The position of CLaSP overlaps that of the…" overlaps with that…

We have corrected this line as recommended.

Line 235, The authors mean reduced PTMs, but this term can bring confusion as it could also refer to reduced (shorter) rRNA forms.

We thank the reviewer for this comment, and propose to change this line to: "Finally, it is possible that CLaSP may mitigate the general reduction in modified ribosomal nucleotides we observe in the *C. burnetii* ribosome (…)".

Line 243, U2506 is E. coli numbering, but the authors discuss C. burnetii ribosome here. E. coli number should instead be in parentheses.

This is a good point that we have addressed in the revision.

Lines 276 and 278, bL4 should be uS4.

Figure 5e: The label should read "A-site" instead of "a-site."

Line 59, Page 4: anti-codon (should be "anticodon")

Line 352: 1.07m (should be typed as "million" particles)

Lines 355-365: 655k…306k, 372 k (should be typed as 655,000 particles)

We have amended these five points as recommended by the reviewer; for the fourth point, we have given the exact number of particles for consistency with the final point.

Reviewer #3 (Remarks to the Author):

Stuart et al. shown a comprehensive cryo-EM analysis of doxycycline bound ribosomes from C. burnetii and E. coli, revealing unexpected antibiotic binding modes in the large ribosomal subunit. The identification of a stacked triple-Dox conformation blocking the nascent peptide exit tunnel in C. burnetii, as well as a striking Dox induced rearrangement of PTC and PET in E. coli, is a beautiful

conceptual advance in our understanding of tetracyclines action. In addition, the author also discovered a new ribosomal protein (CLaSP) and a new hibernation factor (HPFcold) in C. burnetii ribosome.
In general, this work is technically sound, with exceptionally high resolution cryo-EM maps, and careful model building. Overall, the conclusions are largely supported by the data, however, I have few suggestions which may improve this manuscript.

Major concern:
1. According to the authors, the daily clinical dose of Dox is 200 mg, which should correspond to a peak plasma concentration below ~10 μM. However, the structures were solved using 50 μM doxycycline (this is understandable for achieving high occupancy). This raises the possibility that the additional binding site, including the stacked binding mode, may be induced by unphysiological concentrations that are not achieved during standard treatment. While I understand that higher local concentrations may occur in certain tissues or under in vitro conditions, I would be more suggest to use physiologically relevant concentrations. Additionally, please clearly state the Dox concentration used in the main text and in the relevant figure legends.

We have explained in detail in our answer to reviewer 1 (point 8) why we chose the concentration that we did, and that this lies within normal serum concentrations in treated humans. Consequently, we do not feel that repeating at a lower concentration is likely to add significantly to the value of the study and given the difficulty of working with this organism, this would introduce a major delay in the publication of our findings.

The reviewer's suggestion to clearly state the doxycycline concentrations used in the main text and figure legends is a very good one. We have done this (line 84 and figure legend 1).

2. Several statements in the manuscript used a direct causal relationship between the observed structural states and Dox MIC values, which is not sufficiently supported by the data. MIC is influenced by many factors, including uptake, efflux, solubility, metabolism, and ribosome binding at both the 30S and 50S subunits. For example, the statement in Line 255 that "binding in the large subunit … is the major determinant of the Dox MIC in this pathogen" is not supported experimentally and is likely incorrect. Eravacycline can also bind the exit tunnel, and despite the dramatic PTC rearrangements induced by Dox, it does not strongly inhibit E. coli. The triple-stacked binding mode in C. burnetii may therefore not necessarily correlate with higher inhibitory efficiency and could even represent a less efficient or non-physiological state induced by high Dox concentrations.

This is a very fair comment. We have amended the language used through the manuscript appropriately to reflect that whilst the doxycycline triple stack is

suggestive of an effect, it remains to be determined what this effect is. Reviewer 1 made similar points and we have altered the manuscript to reflect both reviewers' opinions.

Throughout the manuscript, strong statements such as "explains," "major determinant," or "accounts for" should be softened to more appropriate language.

Again this is reasonable and we have softened the language at lines 70, 286, 324-5 and 843.

Suggestions:
1. In my view, the central advance of this study is the identification of a new mechanism by which Dox inhibits the ribosome. I would therefore strongly recommend restructuring the manuscript such that the Dox inhibition mechanism is presented first and more prominently, followed by the description of the new hibernation factor and ribosomal protein. At present, the flow of the story is difficult to follow, and the most conceptually important findings appear too late in the manuscript.

We agree with the reviewer about the central advance of the study. Before submission of the manuscript, we tried alternative structures, as suggested, focusing on the Dox inhibition mechanisms at the start of the results section. However, we found this challenging to structure as a coherent narrative and that the present structure worked best. Whilst we appreciate the reviewer's perspective, we fear from our experience that a rearrangement would have a worse flow and be harder to follow.

2. In the Abstract, the statement "challenging the concept that this region is largely static" is misleading. It is well known that antibiotics, nascent polypeptide chains, and even peptide bond formation itself can remodel the peptidyl transferase center (PTC) to some extent. This sentence should therefore be changed.

The reviewer makes a good point. However we are not aware of a conformational change as fundamental as the one we report here, with nucleotide movements of up to 30 Å. Nevertheless, we have reworded this sentence to state: "This significant rearrangement of the peptidyl transferase centre blocks tRNA binding and nascent chain accommodation, abolishing interactions that are fundamental to ribosome function." This focuses the sentence on the salient points of this structural rearrangement.

3. Line 92: "There are notably fewer rRNA post-translational protein modifications (PTMs)" is confusing. Please clarify whether this refers to rRNA modifications or ribosomal protein modifications.

We have amended this to state “post-transcriptional modifications”, as reviewer 1 also pointed out.

4. Line 101: “mRNA entrance tunnel” should be “exit tunnel.”

The reviewer is entirely correct and we have amended this.

5. The abbreviation “r16S” is used repeatedly in this study. However, this is not a standard abbreviation for 16S rRNA and should be changed.

We have amended this throughout the manuscript, following also the suggestions from reviewer 2.

6. Line 144: The term “incomplete binding” is not appropriate and should be changed to a more accurate description. Moreover, this sentence disrupts the logic of the paragraph, as E. coli structures have not yet been introduced at this point. I would suggest to remove it.

We thank the reviewer for this suggestion. We have removed the indicated line, and updated the corresponding line in the *E. coli* section (lines 199-201) to be consistent with this removal.

7. Line 168 states “To test this hypothesis”, referring to the idea that lack of additional chemical elaboration may facilitate this stacked binding mode. However, the following MIC experiments do not directly support this hypothesis, as MIC values are influenced by many non-ribosomal reasons.

We agree with the reviewer’s comment and have removed the phrase “to test this hypothesis. At the conclusion to this paragraph, we have added the line “As MIC results are influenced by a complex network of factors, and as such we caution that a direct causation cannot be drawn between the structural and inhibitory data.” to highlight that there remain multiple potential causes for the effect on MIC.

8. Line 242: “To date, no antibiotic has been observed to block the exit tunnel so extensively” does not add any meaningful information and should be removed.

We agree with the reviewer’s suggestion and have removed this sentence.

9. Cryo-EM particle numbers are consistently reported as approximate values (such as 417k particles). The exact numbers must be used.

This is a fair comment, as reviewer 2 also noted. We have corrected this in the revision.

10. Supplementary Figure 9. HPFcold is not present in the E. coli sample.

We thank the reviewer for noting this error and have corrected it.

11. Figure citations should be checked carefully. For example, the main text (Line 244) cites Fig. S8 in a context where it does not appear relevant, the Supplementary Discussion uses Fig. S8e as support for the claim about A2503 C8 methylation and C. burnetii doxycycline binding. Since Fig. S8 is a processing pipeline, it does not seem to contain the needed structural evidence. Moreover, Line 225/227 should likely cite Fig. S6a/S6b (rather than Fig. S5a/5b); Line 246 "Fig. S9" should be Fig. S10 and Line 276 "Fig. S10b" should be Fig. S11b. Maybe even more?

We apologise for these errors. We have carefully checked the revision to make sure that all supplementary figures are correctly called (as reviewer 1 also noted).

12. Please consider converting Supplementary Table 2 into a standard Nature format table.

We thank the reviewer for suggesting this. We have converted Supplementary Table 2 into the standard Nature format, suggested also by reviewer 2.

Reviewer #4 (Remarks to the Author):

I co-reviewed this manuscript with one of the reviewers who provided the listed reports. This is part of the Nature Communications initiative to facilitate training in peer review and to provide appropriate recognition for Early Career Researchers who co-review manuscripts.

REVIEWERS' COMMENTS

Reviewer #1 (Remarks to the Author):

All comments and concerns that I had about the manuscript have been addressed. The authors provided additional figures that have strengthened their arguments that the doxycycline trimer in the peptide exit tunnel is functionally relevant by justifying the concentration used and characterizing the conservation of the peptide exit tunnel nucleotides. Furthermore, they have characterize the conservation of HPFcold residues that interact with the anti-Shine-Dalgarno sequence, strengthening the article.
I do have a few minor comments that have arisen since submission of the manuscript. These include:
1. In the abstract "cryo electron-microscopy" is hyphenated incorrectly, it should be cryo-electron microscopy.

- We thank the reviewer for noticing this mistake, we have corrected this.

2. Page 2 line 59 – "anti-Shine Dalgarno" should be "anti-Shine-Dalgarno"

- We have corrected this.

3. Page 11 line 213-215 has a sentence "Other key rearrangements include A2062 'flipping in', away from the PTC to base pair with dihydrouridine 2449 and, via a non-canonical base pair, with A2450." This is interesting however, I can not see this interaction in the figures.

- We apologise for this oversight and have added panel (**g**) to figure S16 to show the discussed interaction, added a callout and moved this and the following sentence after the supplementary discussion sentence, to maintain the correct figure callout order. The content of the text remains identical.

4. Supplemental discussion. "Gram-negatives" should be "Gram-negative bacteria"

- We have corrected this.

Reviewer #2 (Remarks to the Author):

The authors have appropriately addressed my initial concerns in the revised manuscript. Despite the absence of mass spectrometry-based identification of the CLaSP peptide, the electron density map is indeed of high-enough resolution to confidently confirm its identity.

In Supplementary Table 2, the standard practice is to report model resolution at a FSC cutoff of 0.5, not 0.143. The sharpening B factor should be provided for each deposited map. Finally, for Rms deviations, what are the values reported in parentheses?

- We thank the reviewer for spotting this and have updated the FSC cutoff values to call out to 0.5 instead. We did not apply a sharpening B factor to the deposited maps and have updated the table to make this clear. We apologise for the lack of detail with the Rms reporting and have clarified the values in parentheses were outliers beyond the 4 sigma threshold.

Reviewer #3 (Remarks to the Author):

The authors have addressed most of the points raised in the first round, and the revised manuscript is clearly improved.

There is one typo: In Supplementary Figure 15b, the protein is labeled as bL4, whereas the main text consistently refers to the same ribosomal protein as uL4.

- We thank the reviewer for spotting this – we have corrected the figure to uL4 in line with the remainder of the manuscript. We have also corrected an incorrect callout from bL4 to uL4 in the supplementary discussion.

Reviewer #4 (Remarks to the Author):

I co-reviewed this manuscript with one of the reviewers who provided the listed reports. This is part of the Nature Communications initiative to facilitate training in peer review and to provide appropriate recognition for Early Career Researchers who co-review manuscripts.
